# Gene Expression Profiling and Clinical Validation Reveal CEBPB for Early Diagnosis of Esophageal Squamous Cell Carcinoma

**DOI:** 10.1002/cnr2.70753

**Published:** 2026-11-08

**Authors:** Jihan Qi, Ning Li, Hongliang Chen, Xinyu Geng, Xueyu Cang, Ranyan Gao, Xinyu Jiang, Lingyi Xu, Fengchun Li, Jiaxin Wang, Shizhu Jin

**Affiliations:** ^1^ Department of Gastroenterology and Hepatology The Second Affiliated Hospital of Harbin Medical University Harbin China

**Keywords:** bioinformatics, biomarker, early diagnosis, esophageal squamous cell carcinoma, molecular diagnosis

## Abstract

**Background:**

Esophageal squamous cell carcinoma (ESCC) accounts for over 90% of esophageal carcinoma cases in Asia, with early‐stage lack of reliable biomarkers and obvious symptoms leading to delayed diagnosis and poor prognosis.

**Aims:**

This study aimed to screen and validate potential diagnostic biomarkers for early ESCC, and explore their regulatory roles in the tumor immune microenvironment.

**Methods and Results:**

This study used weighted gene co‐expression network analysis to identify 1,204 genes strongly correlated with T1 stage tumors. Differential analysis showed 448 upregulated differentially expressed genes, mainly enriched in immune‐ and tumor‐related pathways. Through cross‐analysis, a protein‐protein interaction network was constructed and seven key genes were identified. External dataset validation and receiver operating characteristic curve analysis showed two key genes (CEBPB and CXCL8) had good diagnostic efficiency (AUC > 0.8). Although CXCL8 shows superior diagnostic performance in the GSE53625 transcriptomic dataset, we prioritize CEBPB based on consistent immunofluorescence validation in our tissue samples. Western blotting, immunohistochemistry and immunofluorescence preliminarily verified the expression feature of CEBPB in a small set of three paired clinical samples; larger independent cohorts are required before clinical application of CEBPB as a potential biomarker. Immune cell infiltration analysis revealed significant changes in macrophage populations in ESCC tissues. Five distinct cell types were identified in ESCC, with CEBPB expressed in all. Tissue stem cells and epithelial cells showed strong intercellular communication, while TGFB1‐(TGFBR1+TGFBR2) was widely expressed.

**Conclusion:**

This study highlights CEBPB as a potential biomarker for ESCC, revealing its role in tumor progression and metastasis by modulating the immune microenvironment, providing insights for timely identification and precise management of ESCC.

## 1 Introduction

The sixth leading cause of global mortality in 2020 was esophageal cancer (ESCA), which includes esophageal squamous cell carcinoma (ESCC) and esophageal adenocarcinoma, with approximately 544 000 fatalities [1, 2]. ESCC is a commonly observed cancer in particular areas of East Asia and Africa and constitutes more than 80% of all ESCA cases in China [3, 4]. Owing to the lack of noticeable symptoms such as weight loss and dysphagia, detection of carcinoma in situ is frequently delayed, thus increasing the likelihood of disease progression into invasive carcinoma [5, 6]. The survival rate for patients with ESCC after 5 years is approximately 15%–20%; however, timely identification can significantly increase the likelihood of survival by up to 85% [7]. The current deficiencies in the early identification of ESCC necessitate the development of novel and reliable molecular biomarkers to complement and enhance diagnostic capabilities.

The timely identification and endoscopic resection of early‐stage T1a or T1b ESCC can significantly improve patient prognosis [8]. Early diagnosis of ESCC can be achieved through various methods; the combination of endoscopy and biopsy is widely recognized as the most reliable method for detecting early ESCC (EEC, defined as T1‐stage ESCC with tumor invasion confined to mucosa or submucosa) [9]. EEC can be identified through endoscopic observations of characteristics such as localized mucosal redness, erosion, plaques, and nodules. However, accurate identification of subtle changes in the gastrointestinal mucosa often poses challenges [10, 11]. The diagnostic precision of conventional serological tumor markers for ESCC is relatively limited [12, 13]. As omics technology has advanced, scientists have investigated a wide range of novel biomarkers for detecting ESCC, including free DNA markers [14], metabolites [15], salivary extracellular microRNAs [16], serum long noncoding RNAs (lncRNAs) [17], autoantibodies [18], and others, as well as artificial intelligence‐based methods [19]. However, these strategies are limited by factors such as reliance on advanced technological platforms, methodological inconsistencies, or high costs that impede their extensive implementation in clinical settings. The development of sensitive, specific, reliable, and cost‐effective diagnostic methods is urgently needed to meet the demands of early detection and therapy for ESCC.

Our present research focused on profiling transcriptomic variations during EEC progression, with the goal of uncovering promising biomarkers for early‐stage ESCC recognition. We further characterized how the candidate biomarker participates in reshaping the immune microenvironment, which may facilitate the rational selection of tumor microenvironment (TME)‐targeted antitumor therapies.

## 2 Methods

### 2.1 Transcriptome Data Preparation

The Cancer Genome Atlas (TCGA) and Genotype‐Tissue Expression (GTEx) databases were merged to create a combined TCGA‐GTEx dataset including RNA transcriptome data obtained from the UCSC Xena website (https://xenabrowser.net/). The transcriptome data for the GSE17351, GSE44021, GSE77861, GSE53625, and GSE196756 datasets were obtained from the official website of the Gene Expression Omnibus (GEO) (https://www.ncbi.nlm.nih.gov/geo/) [20]. All downloaded transcriptome datasets were log2‐transformed and quantile‐normalized prior to integration and subsequent analyses. We compiled complete clinical profiles covering tumor stage and pathological classification. Readers can refer to Table 1 and Document S1 for the complete clinical dataset.

**TABLE 1:** Summary of the datasets utilized in this research and their features.

Dataset	Database	Platform	Number	Feature
TCGA‐ESCC	TCGA	TCGA	79	ESCC
GTEx	GTEx	GTEx	273	Esophageal mucosa from HC
GSE17351	GEO	GPL570	10	5 cases of ESCC and 5 controls
GSE44021	GEO	GPL570	12	6 cases of ESCC and 6 controls
GSE77861	GEO	GPL570	14	7 cases of ESCC and 7 controls
GSE53625	GEO	GPL18109	358	12 cases of T1 ESCC; 27 cases of T2 ESCC; 110 cases of T3 ESCC; 30 cases of T4 ESCC; additionally contains 179 independent normal esophageal control samples
GSE196756	GEO	GPL24676	3	ESCC

### 2.2 Weighted Gene Coexpression Network Analysis (WGCNA)

The WGCNA in this research was exclusively based on the transcriptome data of the TCGA‐ESCC cohort (containing 79 ESCC cases) retrieved from the UCSC Xena website (https://xenabrowser.net/); the GTEx database data were not involved in WGCNA module construction. The R package “WGCNA” helped identify gene modules associated with ESCC tumor stage [21]. Genes exhibiting a median deviation greater than 25 were included, whereas those lacking significant expression were excluded. The gene network was constructed by carefully selecting an appropriate soft threshold and calculating coexpression similarity and adjacency. The values were converted into a matrix of topological overlap to perform hierarchical clustering, enabling the identification of modules that exhibit strong correlations with clinical characteristics. Tumor stage was encoded as ordinal numeric variable: T1 = 1, T2 = 2, T3 = 3, T4 = 4 for module‐trait correlation analysis. Significant genes within these modules were determined based on high gene significance (GS) and module membership (MM) values.

### 2.3 Identification of DEGs

The R package “Limma” was utilized to identify DEGs between ESCC and healthy samples based on |Log_2_ fold change (FC)| > 2 and false discovery rate (FDR) < 0.05 thresholds. We utilized “Pheatmap” to construct heatmaps and employed “ggplot2” for volcano plot generation. The intersection set derived from upregulated DEGs and pivotal module genes was visualized by means of the “VennDiagram” R package.

### 2.4 Functional Enrichment Analysis

The R package “clusterProfiler” in R Studio was utilized for functional enrichment analysis of Gene Ontology (GO) and Kyoto Encyclopedia of Genes and Genomes (KEGG) terms, with screening criteria of *p* < 0.05 and FDR < 0.05 [22].

### 2.5 Construction and Modular Examination of a Protein–Protein Interaction (PPI) Network

A PPI network was built with the STRING database (https://string‐db.org/) at a confidence threshold of 0.400 and visualized with Cytoscape software [23, 24]. The MCODE plug‐in with default settings was utilized to detect nodes with high levels of interaction within the subnetwork. The application of the CytoHubba plug‐in and MCC algorithm identified 10 hub genes.

### 2.6 Validation of Key Gene Expression in External Datasets

Key gene expression was validated by integrating three datasets (GSE17351, GSE44021, and GSE77861). ComBat batch correction was performed when integrating GSE17351, GSE44021, and GSE77861 to mitigate cross‐dataset batch effect. Notably, this integrated validation dataset contains only 18 ESCC cases and 18 controls; the analysis is limited by a relatively small sample size. The GSE53625 dataset was utilized to analyze gene expression patterns in normal and malignant tissues at different developmental stages. Gene expression levels were visualized with GraphPad Prism 9.

### 2.7 Receiver Operating Characteristic (ROC) Curves

mRNA expression data from ESCC tissues and matched healthy tissues obtained from multiple datasets were utilized. The R packages “pROC” and “ggplot2” were used to generate the ROC curves. An area under the ROC curve (AUC) approaching 1 indicated greater diagnostic value.

### 2.8 Gene Set Enrichment Analysis (GSEA)

A comparative investigation was performed to examine potential biological roles in ESCC through GSEA utilizing the R package “clusterProfiler,” employing a significance threshold of *p* < 0.05.

### 2.9 Immunoinfiltration Analysis

The CIBERSORT algorithm analyzed the transcriptome data of 22 immune cell types to assess their infiltration levels in the TME. We adopted the canonical LM22 signature matrix built into the CIBERSORT algorithm for human immune cell deconvolution (Document S2). TIMER2.0 (https://cistrome.shinyapps.io/timer/) was utilized to evaluate gene expression in infiltrating immune cells and explore the associated correlations [25].

### 2.10 Clinical Samples

Our research was approved by the Medical Ethics Committee of the Second Affiliated Hospital of Harbin Medical University (Ethics number: YJSKY2022‐269), and three clinical samples of ESCC with their corresponding healthy tissues were included. All patients were diagnosed with ESCC via endoscopic evaluation and pathological staining.

### 2.11 Hematoxylin–Eosin (H&E) Staining

Esophageal tissues were immersed in a 4% paraformaldehyde solution for 12 h for fixation. The tissues were subsequently dehydrated, embedded, and sectioned. After deparaffinization, the sections were subjected to xylene I and II staining for 10 min each. Paraffin removal was performed via gradient ethanol solution prior to hematoxylin staining. The samples were dehydrated by rinsing with tap water, treating with ethanol and xylene, and finally sealing the sections with neutral glue.

### 2.12 Western Blotting

The samples were finely ground, rapidly frozen with liquid nitrogen, and treated with RIPA lysis buffer containing a protease inhibitor. After incubation at 4°C, the resulting supernatant was combined with sample buffer, and total protein was extracted and subjected to electrophoresis. The membrane was transferred and subjected to an overnight incubation with primary antibodies at ambient temperature. The membranes were washed the next day and then exposed to the corresponding secondary antibody (diluted 1:5000) for 60 min before imaging.

### 2.13 qRT‐PCR

The total RNA was subjected to reverse transcription using an All‐in‐One First‐Strand SuperMix to obtain cDNA. Real‐time qPCR was performed using SYBR Green dye on Pangaea Q3 real‐time quantitative PCR system. The specific primer sequences used are as follows. CEBPB, (forward) 5′‐TTTGTCCAAACCAACCGCAC‐3′, (reverse) 5′‐GCATCAACTTCGAAACCGGC‐3′; CXCL8, (forward) 5′‐GAA CTCCAAGCTGGCTGTGG‐3′, (reverse) 5′‐TATGACTCTTGCTGCTCAGC‐3′; ACTIN, (forward) 5′‐CTCTTCCAGCCTTCCTTCCT‐3′, (reverse) 5′‐AGCACTGTGTTGGCGTACAG‐3′.

### 2.14 Immunohistochemical Staining

The paraffin sections were deparaffinized and transferred to an alkaline pressure cooker with 20 mL of EDTA antigen retrieval solution for a 2‐min boil. Following inactivation and blocking, the samples were incubated with antibodies against CEBPB (1:50) and CXCL8 (1:50) overnight at 4°C. The subsequent day, the sections were treated with secondary antibodies suitable for the experiment at ambient temperature, stained, dehydrated, and ultimately sealed for observation.

### 2.15 Immunofluorescence Staining

The ESCC samples were subjected to a 20‐min treatment with a solution containing 4% paraformaldehyde, followed by incubation with the CEBPB antibody (rabbit, ab19379) overnight at 4°C. After the completion of the washing process, the samples were exposed to species‐specific secondary antibodies at room temperature for 2 h. The samples were subsequently sealed with DAPI and examined with a confocal microscope.

### 2.16 Single‐Cell Analysis

The single‐cell transcriptome data (GSE196756) were filtered, preprocessed, and normalized according to the criteria of nFeature_RNA > 50 and percent.mt < 5. Clusters were then reorganized using the JackStraw test algorithm to determine optimal principal components (ScoreJackStraw). The cell grouping was performed with an adjacency distance calculation at a resolution of 0.5 t‐distributed stochastic neighbor embedding cluster analysis and visualization were performed with the RunTSNE function, whereas the R package SingleR was utilized for cell type annotation. Cell clustering relies on the built‐in human reference dataset of SingleR, where tissue stem cell clusters are automatically distinguished from differentiated epithelial cells by the software using predefined epithelial progenitor cell markers for scoring. The FeaturePlot and VlnPlot functions were used to visualize key genes in different cell subtypes.

The CellChatDB database was used to analyze ligand–receptor pairs in humans. Computational methods, such as the computeCommunProb and computeCommunProbPathway functions, were used to determine cellular communication and infer pathway‐level interaction networks. AggregateNet was used to integrate and calculate the communication results between different cell types, and netVisual_circle was employed for visualizing the outcomes. The NetVisual_bubble function was used to generate a bubble map of receptor–ligand pairs.

### 2.17 Statistical Analysis

The study utilized R Studio software and GraphPad Prism 9 for statistical analysis and image generation. Descriptive statistics were employed to determine the means ± standard deviations of normally distributed continuous variables. Gene expression was assessed for statistical significance via independent *t*‐tests and one‐way analyses of variance (ANOVAs), employing a predetermined level of significance at *p* < 0.05.

## 3 Results

### 3.1 WGCNA Explores Potential Biomarkers Associated With EEC

WGCNA was performed to identify crucial genes linked to ESCC progression, using a soft threshold of *β* = 6, which resulted in the development of a scale‐free network (*R* ^2^ = 0.9) (Figure 1a). The gene modules with differences less than 0.4 were merged to construct a dynamic cutting tree (Figure 1b). The analysis of the relationship between tumor progression and each module demonstrated that T1 tumors presented the highest correlation with the green module (Figure 1c). The green module also exhibited a significant positive correlation between GS and MM (cor = 0.5, *p* = 3.7e‐77) (Figure 1d). The subsequent analysis was performed with 1204 genes from the green module.

**FIGURE 1:**
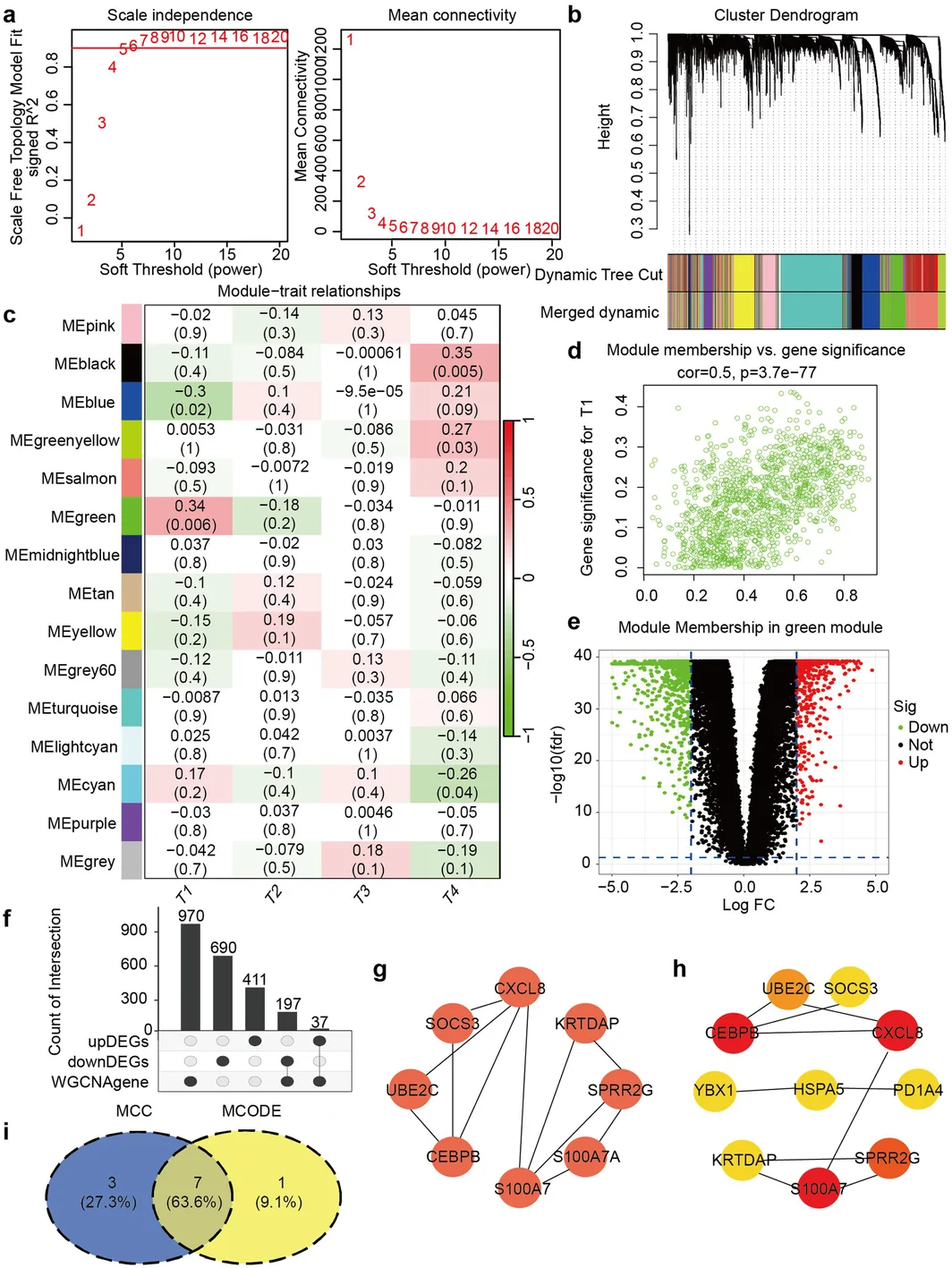
Identification of key genes. (a) WGCNA scale‐free fitting index and average connectivity. (b) Dendrogram of the hierarchical clustering of the coexpression network modules. (c) Correlation analysis between modules and clinical traits, where each row corresponds to a feature gene module and each column corresponds to a clinical parameter. The cells are colored as shown in the color legend based on the correlation. The strength and direction of the correlation are displayed on the right side of the heatmap (red, positive correlation; green, negative correlation). (d) Scatter plot of green module gene correlations. (e) Volcano plot of DEGs in the TCGA‐GTEx cohort. (f) Illustration of the intersection of upregulated DEGs, downregulated DEGs, and green module genes identified via WGCNA. (g) MCODE subnetwork. (h) CytoHubba‐MCC identified hub genes in the network. (i) Venn diagram of the overlapping genes between MCC and MCODE.

### 3.2 Identification of DEGs in ESCC

The analysis of gene expression in ESCC and healthy esophageal tissues revealed 448 upregulated DEGs and 887 downregulated DEGs. These findings were visually represented via a heatmap and volcano plot (Figures 1e and S1). In the present study, we focused on upregulated DEGs for subsequent screening, as elevated gene expression is more amenable to antibody‐based immunohistochemical detection in clinical tissue samples. We acknowledge that downregulated DEGs can also serve as potential biomarkers and warrant independent investigation in future studies.

### 3.3 Functional Enrichment Analysis and Detection of Key Genes

GO analysis revealed significant enrichment of upregulated DEGs in ESCC, particularly in biological processes associated with extracellular matrix (ECM) organization and structure (Figure S2A, Document S3). Furthermore, KEGG analysis demonstrated significant enrichment of upregulated DEGs in the immune response and carcinogenesis pathways (Figure S2B, Document S4). The results of the analysis of downregulated DEGs are illustrated in Figure S2C,D (Documents S5 and S6).

To identify pivotal genes, we cross‐analyzed the 448 upregulated DEGs with the 1204 key module genes, resulting in the identification of 37 overlapping genes (Figure 1f). The cluster module (score: 3.143, 8 nodes and 11 edges) was obtained with the MCODE plugin after the construction of the PPI network (Figure 1g). The CytoHubba‐MCC plugin was utilized to identify the Top 10 core genes (Figure 1h). Seven key genes were selected via these two methods (Figure 1i).

The GO enrichment analysis was conducted on these seven key genes, revealing that their molecular functions are associated primarily with RNA polymerase II core promoter sequence‐specific DNA binding (Figure 2a, Document S7). KEGG enrichment analysis demonstrated significant enrichment of pathways related to TNF signaling and IL‐17 signaling (Figure 2b, Document S8).

**FIGURE 2:**
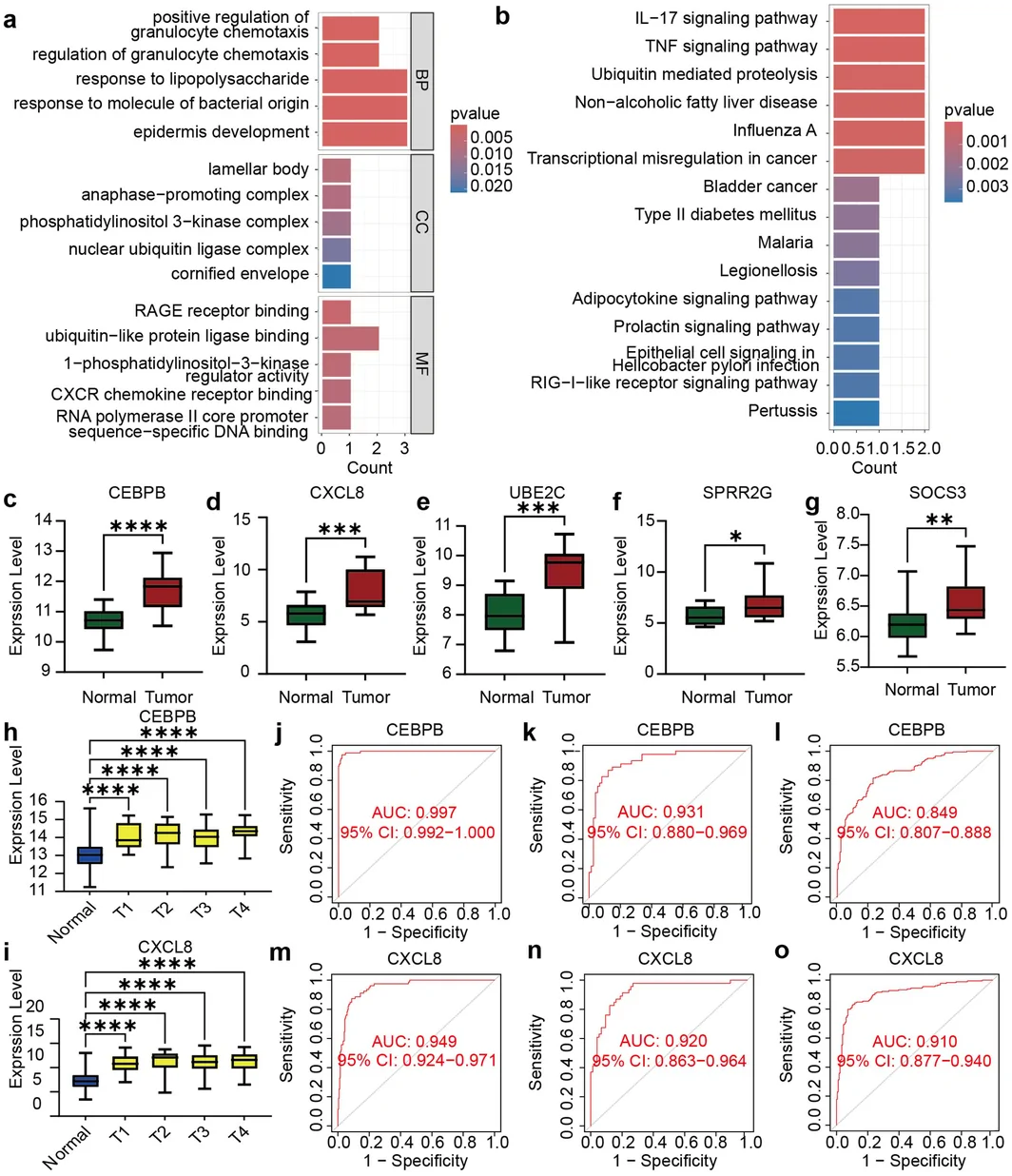
Functional enrichment analysis and Verification of key gene expression in the validation set. (a) Results of the GO enrichment analysis of key genes. (b) Results of KEGG key gene enrichment analysis. Key gene expression in the GSE17351, GSE44021, and GSE77861 datasets: (c) CEBPB; (d) CXCL8; (e) UBE2C; (f) SPRR2G; (g) SOCS3. Key gene expression in the GSE53625 dataset: (h) CEBPB; (i) CXCL8. ROC curves of key genes in the TCGA‐GTEx cohort: (j) CEBPB; (m) CXCL8. ROC curves for the key genes in the GSE17351, GSE44021, and GSE77861 datasets: (k) CEBPB; (n) CXCL8. ROC curves for the key genes in the GSE53625 dataset: (l) CEBPB; (o) CXCL8. *p < 0.05, **p < 0.01, ***p < 0.001, ****p < 0.0001, ns: nonsignificant.

### 3.4 External Dataset Validation of Key Genes

The datasets GSE17351, GSE44021, and GSE77861 were integrated for batch analysis to validate key gene expression. These three datasets were selected for external validation as they share the same GPL570 microarray platform (reducing technical variation), adopt a case–control design (each containing ESCC cases and healthy esophageal tissue samples for expression comparison), and their integration (18 ESCC cases and 18 controls in total) enhances statistical robustness. Of note, this merged GPL570 dataset has limited sample size, and the diagnostic results should be interpreted cautiously. The tumor group exhibited a significant upregulation of CXCL8, SOCS3, CEBPB, and SPRR2G compared to the control group (Figures 2c–g and S3). The relationships between tumor stage and these genes were assessed using the GSE53625 dataset, excluding UBE2C from further analysis. The findings revealed a significant upregulation in the levels of CEBPB and CXCL8 expression in both tumor tissues and T1 tumors (Figures 2h,i and S3). ROC curve analysis revealed that CEBPB and CXCL8 had AUC values of 0.997 and 0.949, respectively, in the TCGA‐GTEx dataset (Figure 2j,m). Given potential cross‐platform bias between TCGA and GTEx, the AUC values derived from TCGA‐GTEx should be interpreted cautiously; we place higher weight on diagnostic results from GEO same‐platform datasets. In the integrated dataset, CEBPB had an AUC value of 0.931, whereas CXCL8 had an AUC value of 0.920 (Figure 2k,n). In the GSE53625 dataset, CEBPB and CXCL8 achieved AUC values of 0.849 and 0.910, respectively (Figure 2l,o). Therefore, the key genes of EEC were identified as CEBPB and CXCL8.

### 3.5 Immune Landscape Analysis in ESCC

GSEA was used to improve the accuracy of identifying pivotal gene functions, revealing their significant enrichment in tumor‐ and immune‐related pathways (Figure 3a,b). The immune cells in the TME are considered a general component of tumors. We utilized the CIBERSORT algorithm to evaluate immune cell infiltration within the TCGA‐GTEx cohort and determine the proportions of 22 distinct categories of immune cells (Figure 3c). The distribution of immune cells was visualized with heatmaps and violin plots (Figure 3d,e). The results demonstrated a pronounced alteration in macrophage distribution within ESCC tissues. The proportions of M0 and M1 macrophages were significantly greater in ESCC tissues than in healthy tissues, while healthy tissues presented a higher abundance of M2 macrophages. This distinct macrophage distribution pattern is a characteristic feature of early‐stage ESCC TME, differing from the M2‐dominant immune landscape widely reported in advanced ESCC.

**FIGURE 3:**
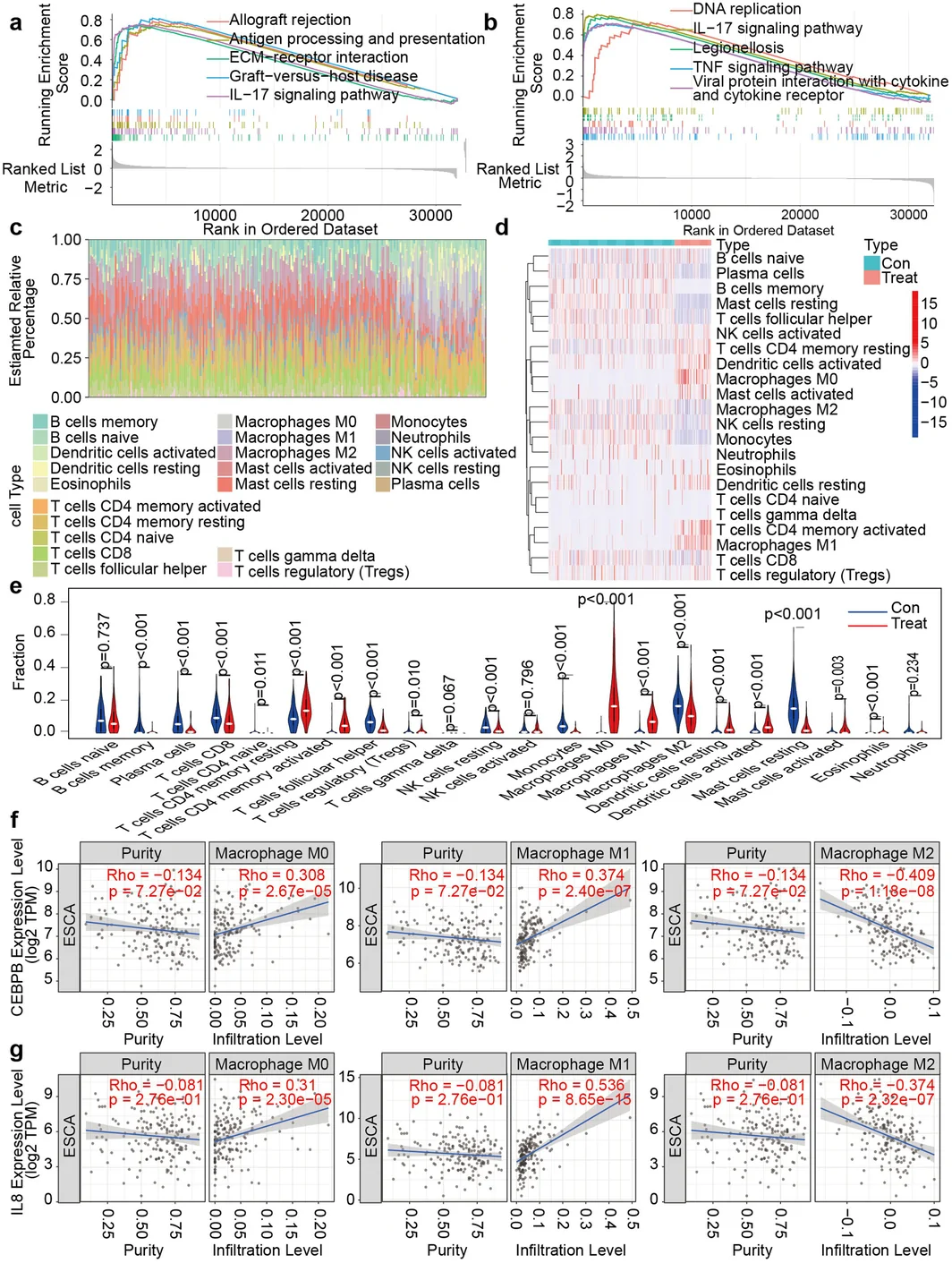
GSEA and immune‐related analysis. (a) GSEA of CEBPB. (b) GSEA of CXCL8. (c) Comparison of the degree of infiltration of different immune cells between the tumor group and the healthy group. (d) Heatmap showing the differences in infiltrating immune cells between the tumor group and the healthy group. (e) Histogram showing the difference in infiltrating immune cells between the tumor group and the healthy group. (f) The expression level of CEBPB is correlated with the infiltration levels of M0, M1, and M2 macrophages, as well as the purity of tumor cells. (g) The expression level of CXCL8 is correlated with the infiltration levels of M0, M1, and M2 macrophages, as well as the purity of tumor cells.

The TIMER2.0 tool was employed to evaluate the correlation between mRNA expression of crucial genes and immune cell infiltration levels in ESCA. The results revealed a strong positive correlation between CEBPB and CXCL8 expression levels and the infiltration of M0 and M1 macrophages (Cor = 0.308, Cor = 0.310; Cor = 0.374, Cor = 0.536), but a negative correlation with M2 macrophage infiltration (Cor = −0.409, Cor = −0.374) (Figure 3f,g).

### 3.6 Experimental Verification of Key Gene Expression

The expression of pivotal genes in EEC patients was further validated through preliminary experimental demonstration based on three paired ESCC and matched healthy tissues. The left and anterior walls of the esophagus exhibited a rough texture under white light gastroscope examination, characterized by slightly elevated white plaque. The pigmented endoscopic mode enhanced lesion visibility. Confocal microendoscopy revealed a loss of polarity in the squamous epithelium arrangement, along with an increase in disordered and distorted capillary loops. Moreover, increased intercellular effusion of sodium fluorescein was detected (Figure 4a). H&E staining of esophageal tissue revealed that the healthy control (HC) group had intact structural integrity, mild hyperplasia, slightly enlarged nuclei in the mucosal epithelium, and a slightly disordered polarity arrangement. In contrast, the EEC group exhibited severe dysplasia in the squamous epithelium of the mucosa with enlarged nuclei, deep staining, disrupted polarity, and irregular arrangement (Figure 4b). Western blotting revealed statistically significant upregulation of CEBPB and CXCL8 expression in EEC patients (*n* = 3 paired samples, *p* = 0.0004 for CEBPB, *p* = 0.0002 for CXCL8) (Figure 4c, Documents S9 and S10). To further confirm the specific expression of CEBPB and CXCL8 in ESCC, we performed supplementary qRT‐PCR and Western blotting analyses using the ESCC cell line Eca109 and normal esophageal epithelial cell line Het‐1A (control). Results showed significant upregulation of both genes in Eca109 versus Het‐1A cells at both the mRNA and protein levels (*p* < 0.05), with consistent trends between the two assays (Figure 4d,e, Documents S11 and S12).

**FIGURE 4:**
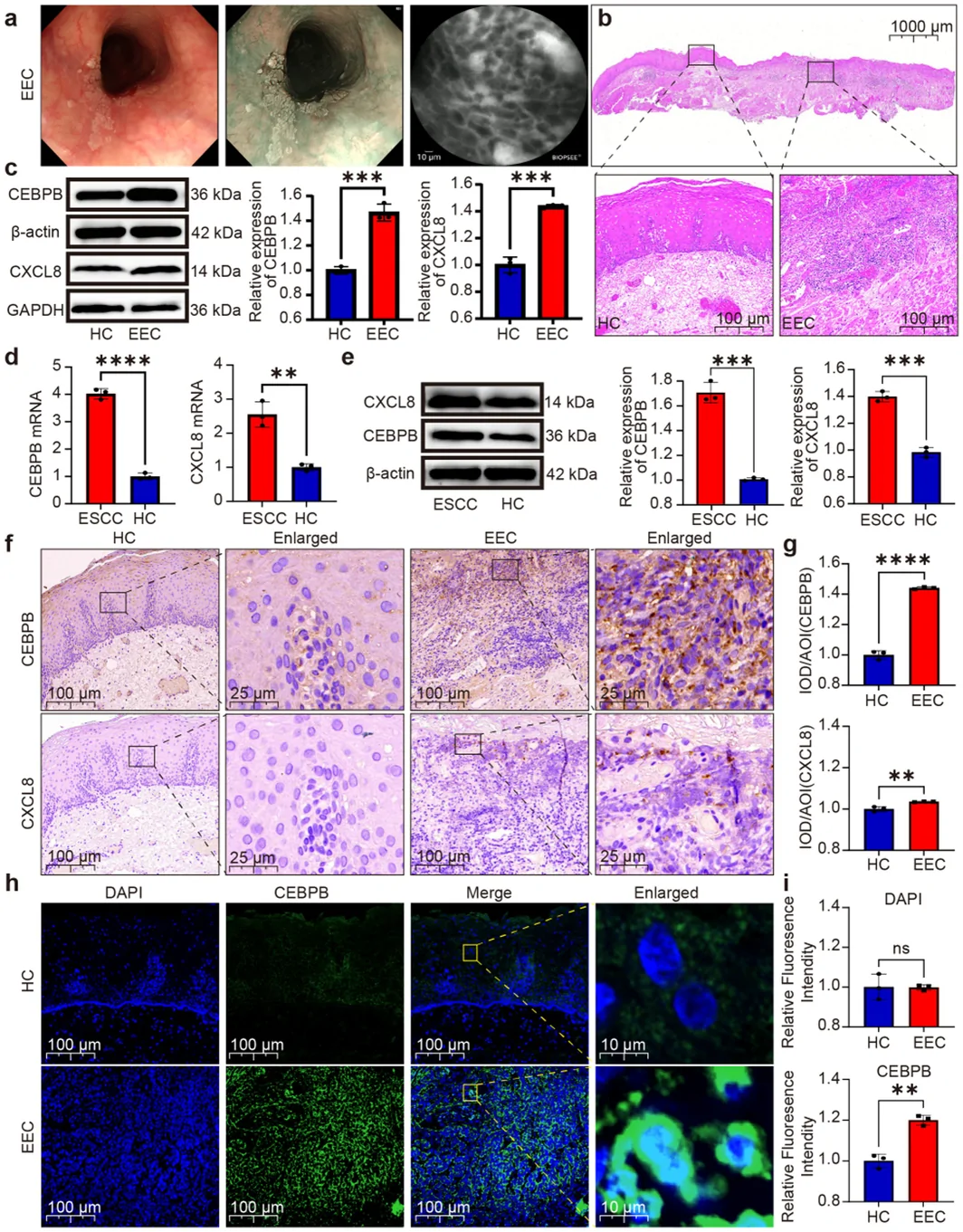
Validation of key gene expression. (a) Endoscopic examination of the esophagus of ESCC patients under white‐light gastroscopy. Endoscopic examination of the esophagus of ESCC patients under NBI gastroscopy. Esophageal cancer in ESCC patients observed via CLE1000e confocal endoscopic microscopy. (b) Representative image of histological staining of esophageal tissue. (c) Western blot representation and statistical analysis of CEBPB and CXCL8 in esophageal tissues (*n* = 3, *p* = 0.0004 for CEBPB, *p* = 0.0002 for CXCL8). (d) CEBPB and CXCL8 mRNA expression in ESCC and normal esophageal epithelial cell lines. (e) CEBPB and CXCL8 protein expression in ESCC and normal esophageal epithelial cell lines (*n* = 3, *p* = 0.0001 for CEBPB, *p* = 0.0002 for CXCL8). (f) Immunohistochemical staining of normal esophageal tissue and EEC tissue following treatment with the anti‐CEBPB and the anti‐CXCL8 antibody. (g) Histogram of the results of the quantitative analysis of immunohistochemical staining. (h) Immunofluorescence images of normal esophageal tissue and EEC tissue stained with an anti‐CEBPB antibody. (i) Histogram of the results of the quantitative analysis of immunofluorescence staining. *P < 0.05, **P < 0.01, ***P < 0.001, ****P < 0.0001.

CEBPB and CXCL8 antibodies were used to analyze esophageal tissues through immunohistochemistry. In the HC group, intact tissue structure was observed without abnormal squamous epithelial cells or malignant tumor cells, and no histochemical staining was detected. In contrast, the EEC group showed infiltration of malignant tumors into the submucosa with a significant presence of heterotypic carcinomatous cells exhibiting intense nuclear and cytoplasmic staining. The expression levels of CEBPB and CXCL8 significantly differed between the EEC group and the HC group, as quantitatively analyzed (*p* < 0.05) (Figure 4f,g).

Immunofluorescence analysis was used for quantitative and spatial assessment of CEBPB because of its superior recognition efficiency in immunohistochemical staining analysis. The results revealed widespread cytoplasmic expression of CEBPB, with significant expression observed in EEC patients, which was consistent with the trends observed via Western blotting and immunohistochemical analysis (Figure 4h). Quantitative analysis did not yield any noteworthy variation in DAPI fluorescence intensity between the EEC group and the HC group. However, the expression of CEBPB showed a significant contrast between the mentioned groups (Figure 4i). However, repeated attempts did not produce interpretable immunofluorescence signals for CXCL8 in paraffin sections, which limited further protein localization analysis of CXCL8 at the single‐cell level.

### 3.7 Single‐Cell Analysis of ESCC

Quality control of the ESCC single‐cell analysis revealed that 20 clusters were optimal, with a *p*‐value less than 0.05 for each principal component (PC) (Figure 5a–c). The data were subjected to t‐SNE cluster analysis, which revealed 11 distinct cell clusters. Five specific cell subtypes were subsequently distinguished: T cells, B cells, epithelial cells, monocytes, and tissue stem cells (Figure 5d). The representative marker differentially expressed genes corresponding to each cluster are listed in Document S13 and Figure S4. The spatial distribution of CEBPB in ESCC was visualized by generating a scatter plot (Figure 5e), and a violin plot was generated to provide a visual representation of its expression levels across different cell subtypes (Figure 5f). The receptor–ligand pair types in the human single‐cell transcriptome database were visualized (Figure 5g). Intercellular communication was observed at the signaling pathway level, indicating cell communication among all cell subtypes (Figure 5h,i). In particular, epithelial cells and tissue stem cells exhibited strong intercellular communication with other cells (Figure 5j). The investigation of cell communication interactions revealed the widespread presence of TGFB1‐(TGFBR1+TGFBR2) in all cells (Figure 5k). We emphasize that these are computationally inferred interactions from transcriptome data; direct functional validation is required to confirm biological causality.

**FIGURE 5:**
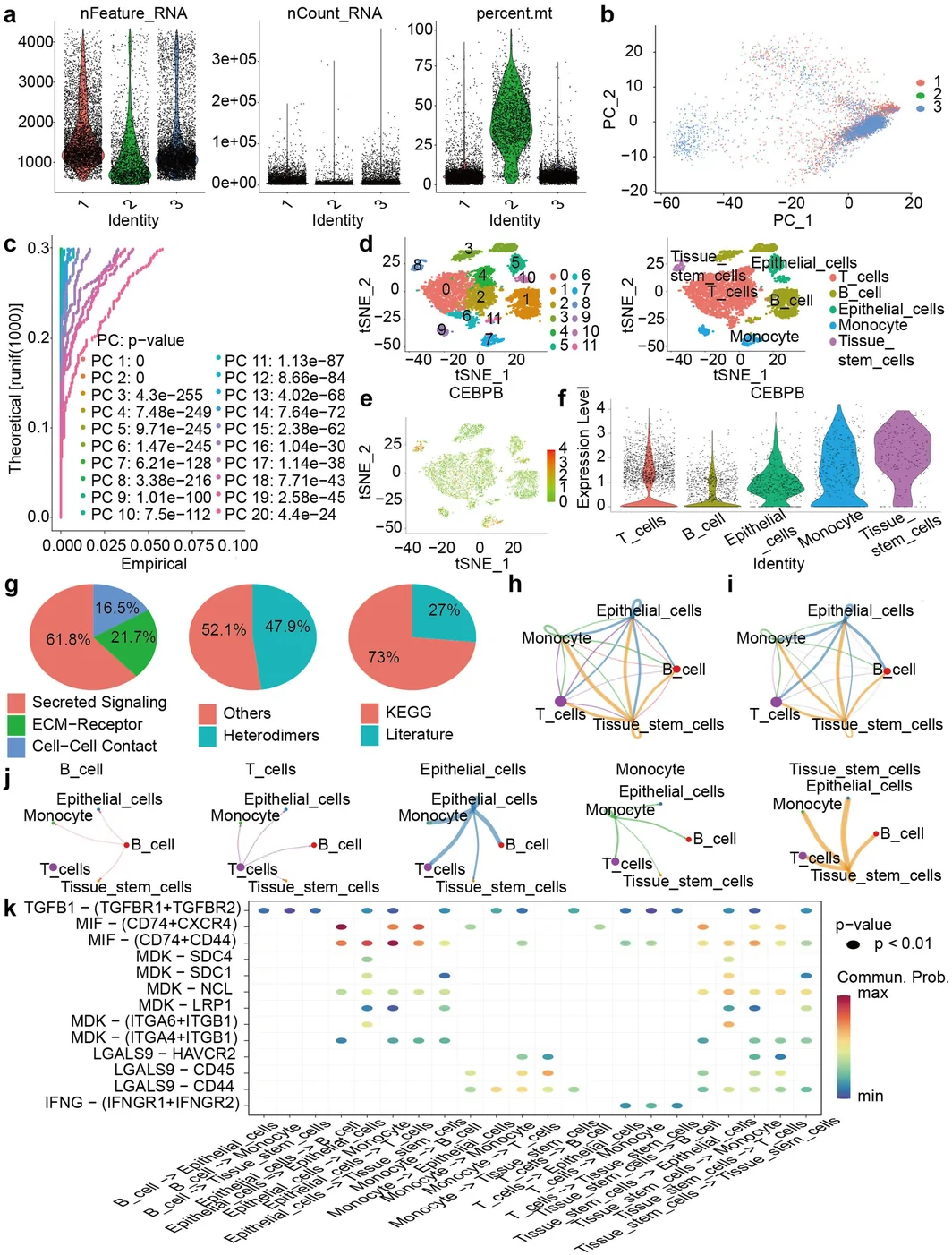
Single‐cell analysis of CEBPB in ESCC. (a) Violin plot of nFeature, nCount, and percent.mt after quality control. (b) PCA determines the optimal dimension. (c) Distribution of *p* values according to PC. (d) Composition of single cells in ESCC. (e) Expression distribution of CEBPB in single cells. (f) Violin plot of the expression level of CEBPB in single cells. (g) Pie chart illustrating the types of receptor–ligand pairs. (h) Diagram illustrating the number of cellular communication interactions. (i) Diagram illustrating the strength of the cellular communication interactions. (j) Communication between individual cells and other cells. (k) Bubble plot of receptor–ligand pairs.

## 4 Discussion

ESCA has consistently been a major global public health concern, with ESCC being the predominant subtype in China. In its early stages, ESCC often presents asymptomatically, leading to rapid local tumor progression and metastasis and delayed diagnosis. Additionally, the inherent treatment resistance and frequent recurrence of ESCC contribute to a poor prognosis for patients [26, 27]. The exploration of molecular mechanisms in the TME is crucial for identifying potential targets for diagnosing and treating ESCC.

The TNM system is primarily used for the clinical staging of ESCC, but current imaging techniques have limited accuracy in distinguishing lymph node metastasis and T stage, achieving only an approximately 50% success rate. Importantly, pathological evaluation and tumor heterogeneity can impact staging outcomes [28, 29, 30]. The combination of biomarkers and clinicopathological parameters is crucial for facilitating the endoscopic diagnosis of EEC. In the present work, we identified CEBPB upregulation in ESCC and verified its favorable diagnostic performance via WGCNA and complementary analytical approaches. Notably, our WGCNA network was built exclusively on 79 TCGA‐ESCC samples; such a moderate sample size may introduce potential instability in the co‐expression network. The observed association between the green module and T1‐stage ESCC still requires validation in larger independent cohorts. It is worth noting that CXCL8 achieved a higher mRNA‐level AUC (0.910) than CEBPB (0.849) within the GSE53625 cohort. Nevertheless, the strong transcriptomic diagnostic performance of CXCL8 was not fully reproduced at the protein level in our patient tissue specimens. Immunohistochemistry revealed only a weak increase in CXCL8 protein abundance in EEC tissues, and we failed to obtain reliable immunofluorescence signals for CXCL8. In comparison, the differential expression of CEBPB was validated by both IHC and immunofluorescence assays. Accordingly, CEBPB was chosen as the core molecule emphasized in the title and abstract. CXCL8 remains presented herein as a promising mRNA biomarker for EEC.

The TME is crucial for the progression of cancer and its response to treatment and includes factors such as the vasculature surrounding the tumor, immune cell infiltration, fibroblasts, signaling molecules, and the ECM [31, 32]. Tumor‐associated macrophages constitute the primary constituents of the TME. The communication between cancer cells and tumor‐associated macrophages enhances tissue restructuring and promotes cancer cell invasiveness [33]. The complex relationship between cancer cell attributes and the TME necessitates a deeper exploration of the dynamic interaction between ESCC and the TME. Most previous studies observed abundant M2‐type TAMs in advanced ESCC, which promote tumor progression via immune suppression and ECM remodeling. However, our CIBERSORT results based on the TCGA‐GTEx cohort revealed enriched M0 and M1 macrophages in early T1‐stage ESCC, while normal esophageal mucosa harbored more M2 macrophages. This discrepancy arises from the different disease stages investigated. Early precancerous lesions trigger acute anti‐tumor inflammation dominated by undifferentiated M0 and anti‐tumor M1 macrophages, without large‐scale M2 polarization. Combined with our TIMER2.0 correlation results, CEBPB and CXCL8 are positively correlated with M0/M1 infiltration in early ESCC, indicating immature monocyte/macrophage precursors as the primary source of CXCL8 at this stage. With tumor progression to late stages, sustained tumor signaling drives macrophage M2 polarization, and M2 TAMs will become an additional cellular source of CXCL8, consistent with prior literature [34, 35, 36].

The functional examination demonstrated that CEBPB is significantly enriched in the ECM and its associated structures, as well as the “ECM‐receptor interaction” pathway. The ECM is a vital cellular component found on the cell surface or within the intercellular space, playing a crucial role in inhibiting tumor cell metastasis. However, an imbalance in ECM composition can drive cancer progression by promoting tumor proliferation and metastasis, angiogenesis induction, immune evasion, and genomic instability [37, 38, 39]. Numerous studies have provided evidence that diverse ECM molecules exert regulatory functions in the progression and metastasis of ESCC [40, 41, 42]. The present study extracted information about cell communication interactions, revealing the ubiquitous presence of TGFB1‐(TGFBR1+TGFBR2) across all cellular subtypes. This observation only reflects transcriptomic intercellular communication, and the direct regulatory effect of TGF‐β signaling on CEBPB‐related pathways cannot be confirmed by current sequencing data. The surrounding ECM within the microenvironment stimulates transforming growth factor (TGF)‐β and other signaling pathways, thereby inducing transcriptional programs involved in epithelial–mesenchymal transition (EMT). EMT involves the disruption of apicobasal polarity and intercellular adhesion in epithelial cells, leading to their transformation into aggressive mesenchymal cells [43, 44]. The pivotal role of key genes in shaping the TME suggests their potential contribution to immune evasion, making them promising targets for novel TME therapy strategies.

The CEBPB protein is characterized by an alkaline leucine zipper pattern; it acts as a transcription factor and has crucial functions in controlling cellular growth and development and tumor formation [45, 46, 47, 48, 49]. The upregulation of CEBPB in ESCC has been demonstrated to be concomitant with copy number amplification [50]. KDM6B expression is increased in patients with ESCC and lymph node metastasis, activating the transcription of C/EBP‐β through an inhibitory H3K27me3 marker on a demethylated promoter bound by KDM6B. EMT is consistently induced by Snail via the TNFα/NF‐κB signaling pathway, which significantly promotes tumor cell migration and invasion [51, 52]. The interaction between ATAD2 and CEBPB facilitates its translocation into the nucleus, enabling direct binding to the promoter region of TGF‐β1 and enhancing its transcriptional activity. This activation is Smad3 dependent, leading to Snail induction and subsequent EMT, promoting ESCC metastasis [53]. Based on published literature, the CEBPB/TGF‐β/Snail/EMT regulatory pathway is summarized in Figure 6. This study has demonstrated through multi‐omics data and clinical samples only that CEBPB is highly expressed in early‐stage ESCC and holds diagnostic value; however, the causal relationship between the TGF‐β signaling pathway and CEBPB requires further functional validation. These results indicate that the involvement of CEBPB is significant in the development and advancement of ESCC, and the protein potentially serves as both a diagnostic indicator and therapeutic target in EEC.

**FIGURE 6:**
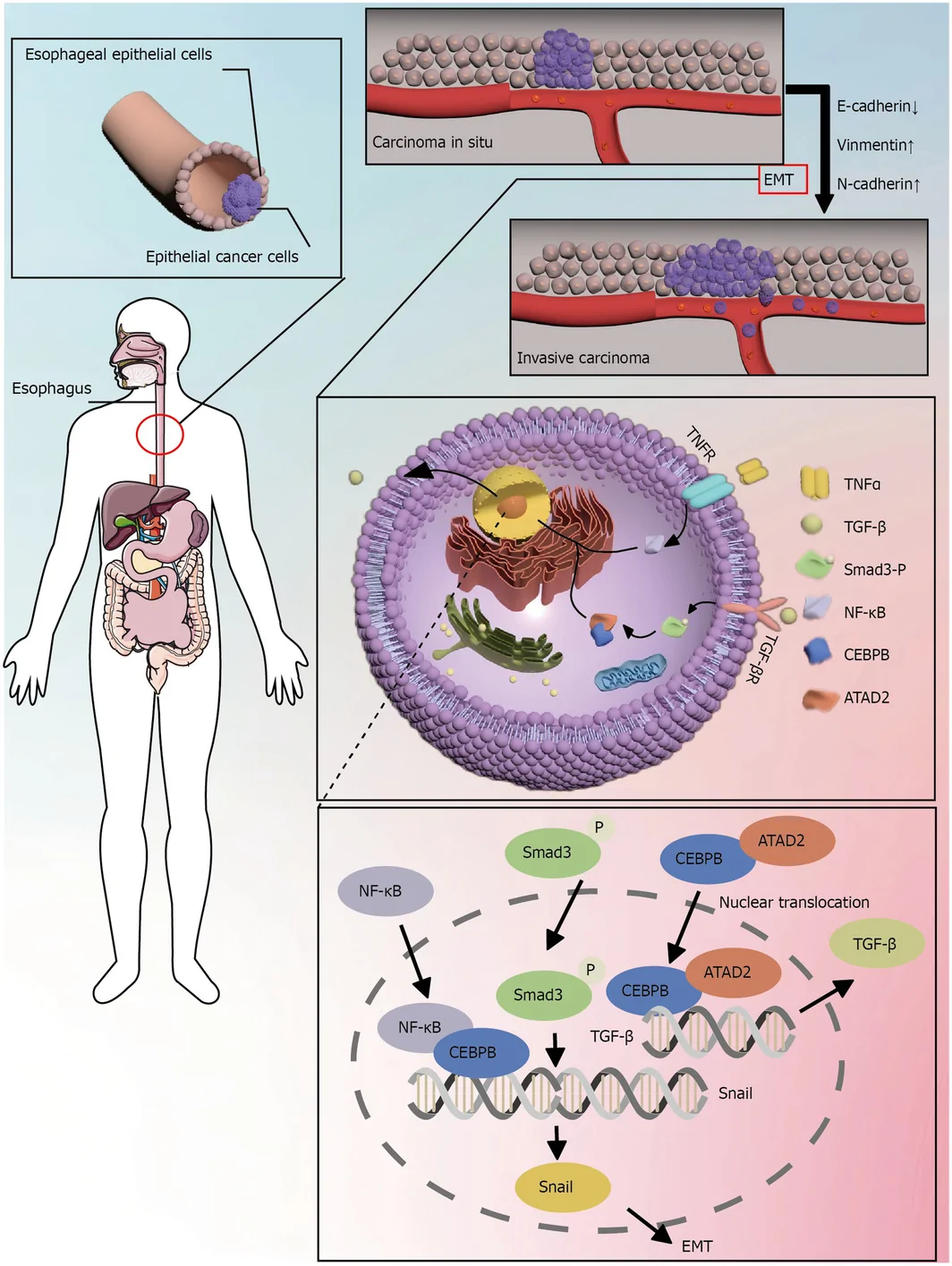
Proposed literature‐supported hypothetical model illustrating potential roles of CEBPB in ESCC initiation and metastasis. This schematic represents a hypothetical regulatory model compiled from previously published literature rather than mechanisms directly proven by the present study. CEBPB may promote ESCC metastasis through epithelial‐mesenchymal transition (EMT). Interaction with ATAD2 facilitates CEBPB nuclear translocation, where it may directly bind the TGF‐β1 promoter to enhance transcriptional activity in a Smad3‐dependent manner. This may activate TGF‐β/Smad3 and TNFα/NF‐κB signaling pathways, inducing Snail expression to drive EMT. CEBPB may orchestrate ESCC progression by modulating ECM‐receptor interactions and EMT via multi‐pathway crosstalk. Further wet‐lab functional experiments are needed to verify these causal relationships.

The potential biomarker CEBPB shows promise in aiding timely diagnosis and personalized therapy of ESCC, enhancing the feasibility of early detection. With advancements in molecular biology technology, integrating multiomics approaches is increasingly emphasized; these methods have substantial value in comprehending the role of CEBPB within the TME and its clinical applications. The limitations of public databases and infrequent experimental verification in human esophageal tissue necessitate future studies to broaden sample diversity, incorporate standardized detection methods, and conduct clinical validation. We note that high AUC values obtained from retrospective tumor‐versus‐normal comparisons in public datasets may be influenced by dataset‐specific technical effects and cannot directly represent prospective real‐world diagnostic accuracy for population‐level early screening. Future studies will enroll larger independent clinical cohorts to calculate sensitivity, specificity, AUC with confidence intervals and implement blinded evaluation for validating the real‐world diagnostic performance of CEBPB. Our single‐cell transcriptome analysis only characterized broad cell lineages to map the general expression of CEBPB in early ESCC, without further subclustering of monocytes and epithelial progenitor subsets. High‐resolution subtyping of myeloid cells will be implemented in our subsequent mechanistic investigation to pinpoint the exact monocyte subpopulation producing CEBPB. This approach will facilitate the translation of potential biomarkers into clinical applications and improve early screening and diagnosis for high‐risk ESCC patients. Moreover, identifying key gene targets will contribute to achieving precise targeted therapy for EEC. The complex immune microenvironment during tumor progression necessitates further investigation to comprehensively comprehend the role of CEBPB in EEC growth and dissemination.

The marked effectiveness of CEBPB in detecting and recognizing ESCC in a timely manner suggests its potential as a valuable asset for promoting cancer screening and diagnosis. Furthermore, CEBPB may exert a significant regulatory role in modulating the immune microenvironment during the early stages of ESCC progression and dissemination.

## Author Contributions

**Jihan Qi:** writing – original draft, validation, visualization, software, formal analysis, data curation, conceptualization. **Ning Li:** writing – original draft, visualization, methodology, validation, formal analysis, data curation. **Hongliang Chen:** validation, data curation, visualization, methodology. **Xinyu Geng:** validation, visualization, software, formal analysis. **Xueyu Cang:** data curation, visualization, validation. **Ranyan Gao:** data curation, validation, formal analysis. **Xinyu Jiang:** validation, visualization. **Lingyi Xu:** validation, visualization. **Fengchun Li:** data curation. **Jiaxin Wang:** data curation. **Shizhu Jin:** conceptualization, investigation, funding acquisition, writing – review and editing, project administration, resources, supervision.

## Funding

The authors have nothing to report.

## Ethics Statement

Informed consent was obtained from all patients, and this study was approved by the Ethics Committee of the Second Affiliated Hospital of Harbin Medical University (Ethics No. YJSKY2022‐269).

## Conflicts of Interest

The authors declare no conflicts of interest.

## Supporting information


**Figure S1:** Heatmap of DEGs in the TCGA‐GTEx cohort. Rows represent upregulated DEGs (*n* = 448) and downregulated DEGs (*n* = 887); columns represent tumor and normal samples. Red and green indicate high and low expression, respectively.

**Figure S2:** Functional enrichment analysis of DEGs. (A) GO enrichment results for the upregulated DEGs. (B) KEGG enrichment results for the upregulated DEGs. (C) GO enrichment results for the downregulated DEGs. (D) KEGG enrichment results for the downregulated DEGs.

**Figure S3:** Verification of key gene expression in the validation set. Key gene expression in the GSE17351, GSE44021, and GSE77861 datasets: (A) CEBPB; (B) CXCL8; (C) UBE2C; (D) SPRR2G; (E) SOCS3; (F) S100A7; (G) KRTDAP. Key gene expression in the GSE53625 dataset: (H) CEBPB; (I) CXCL8; (J) SOCS3; (K) SPRR2G. *p < 0.05, **p < 0.01, ***p < 0.001, ****p < 0.0001, ns: nonsignificant.

**Figure S4:** Marker gene heatmap of major cell clusters. Rows represent marker genes and columns represent cell clusters.

**Document S1:** Clinical characteristics of the study samples and datasets. Detailed clinicopathological information, including tumor stage and pathological classification, of the samples used in this study.

**Document S2:** LM22 immune cell signature matrix. The canonical LM22 gene signature matrix used for CIBERSORT deconvolution of 22 human immune cell subtypes.

**Document S3:** GO enrichment analysis of the upregulated DEGs. Full list of enriched Gene Ontology terms, including biological processes associated with extracellular matrix organization and structure.

**Document S4:** KEGG pathway enrichment analysis of the upregulated DEGs. Full list of enriched KEGG pathways, including immune response and carcinogenesis pathways.

**Document S5:** GO enrichment analysis of the downregulated DEGs. Full list of enriched Gene Ontology terms.

**Document S6:** KEGG pathway enrichment analysis of the downregulated DEGs. Full list of enriched KEGG pathways.

**Document S7:** GO enrichment analysis of the seven key genes. Full list of enriched Gene Ontology terms.

**Document S8:** KEGG pathway enrichment analysis of the seven key genes. Full list of enriched KEGG pathways, including TNF signaling and IL‐17 signaling pathways.

**Document S9:** Quantitative analysis of CEBPB protein expression by Western blotting in paired EEC and adjacent normal esophageal tissues (*n* = 3).

**Document S10:** Quantitative analysis of CXCL8 protein expression by Western blotting in paired EEC and adjacent normal esophageal tissues (*n* = 3).

**Document S11:** qRT‐PCR analysis of CEBPB and CXCL8 mRNA expression in the ESCC cell line Eca109 and the normal esophageal epithelial cell line Het‐1A (*n* = 3).

**Document S12:** Western blotting analysis of CEBPB and CXCL8 protein expression in Eca109 and Het‐1A cells (*n* = 3).

**Document S13:** Cluster‐specific marker genes of the five major cell subtypes. The complete list of marker differentially expressed genes for T cells, B cells, epithelial cells, monocytes, and tissue stem cells.

## Data Availability

This paper analyzes existing, publicly available data, accessible at (GEO database: GSE17351, GSE44021, GSE77861, GSE53625, GSE196756) and (UCSC Xena website https://xenabrowser.net/). Original images have been deposited at Mendeley at (https://doi.org/10.17632/f8ctdwzf29.1) and are publicly available as of the date of publication. Microscopy data reported in this paper will be shared by the lead contact upon request. Any additional information required to reanalyze the data reported in this paper is available from the lead contact upon request.
