# Supplementary material for: Gene Expression Profiling and Clinical Validation Reveal CEBPB for Early Diagnosis of Esophageal Squamous Cell Carcinoma
Source: Cancer Rep (Hoboken). 2026 Nov 8;9(11):e70753. doi: 10.1002/cnr2.70753 (PMC13745642; doi:10.1002/cnr2.70753)
Supplement: Supplementary file 1 [file CNR2-9-e70753-s001.pdf]

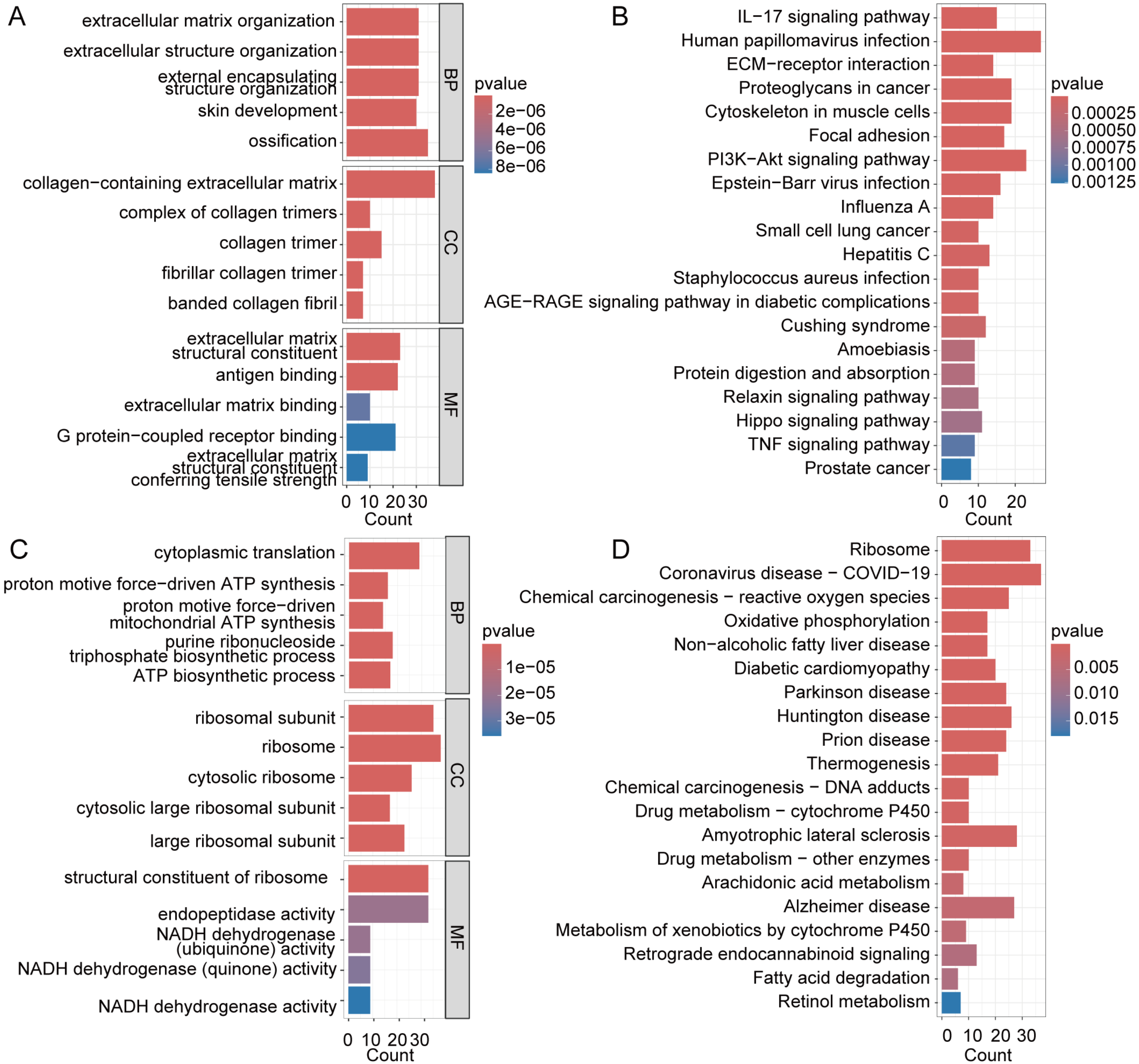

Fig S2. Functional enrichment analysis of DEGs

A GO enrichment results for the upregulated DEGs. **B** KEGG enrichment results for the upregulated DEGs. **C** GO enrichment results for the downregulated DEGs. **D** KEGG enrichment results for the downregulated DEGs.
