# Supplementary material for: Gene Expression Profiling and Clinical Validation Reveal CEBPB for Early Diagnosis of Esophageal Squamous Cell Carcinoma
Source: Cancer Rep (Hoboken). 2026 Nov 8;9(11):e70753. doi: 10.1002/cnr2.70753 (PMC13745642; doi:10.1002/cnr2.70753)
Supplement: Supplementary file 2 [file CNR2-9-e70753-s002.pdf]

Doc.S11 qRT-PCR analysis of CEBPB and CXCL8 mRNA expression in the ESCC cell line Eca109 and the normal esophageal epithelial cell line Het-1A (n=3).

样本ID	CEBPB	CXCL8	ACTIN	ΔCT (CEBPB-内参CT)
treat	23.72	24.92	18.95	4.77
treat	23.81	25.45	19.07	4.74
treat	23.97	25.32	19.1	4.87
NORMAL	25.37	26.46	18.75	6.62
NORMAL	25.22	25.77	18.29	6.93
NORMAL	25.28	25.83	18.41	6.87

归一化	treat	NORMAL	treat	NORMAL
	4.102964486	1.138131035	2.962195105	0.886791389
	4.189176491	0.91806402	2.229417273	1.040059934
	3.828201228	0.957050307	2.490899245	1.08422687
		1.004415121		1.003692731
	4.084929032	1.133128137	2.951296759	0.883528755
	4.170762074	0.914028474	2.221214924	1.036233403
	3.811373555	0.952843389	2.481734865	1.080237842

ΔCT (CXCL8-内参CT)	对照组ΔCT均值(CEBPB)	对照组ΔCT均值(CXCL8)
5.97	6.806666667	7.536666667
6.38	6.806666667	7.536666667
6.22	6.806666667	7.536666667
7.71	6.806666667	7.536666667
7.48	6.806666667	7.536666667
7.42	6.806666667	7.536666667

ΔΔCT (CEBPB)	ΔΔCT (CXCL8)	2-ΔΔCT (CEBPB)	2-ΔΔCT (CXCL8)
-2.036666667	-1.566666667 -	4.	2.
2.066666667	-1.156666667 -1.	1029644864.	9621951052.
936666667	-1.316666667	1891764913.	2294172732.
-0.186666667	0.173333333	8282012281.	4908992450.
0.123333333	-0.056666667	138131035	8867913891.
0.063333333	-0.116666667	0.91806402	040059934
		0.957050307	1.08422687
