# Supplementary material for: Gene Expression Profiling and Clinical Validation Reveal CEBPB for Early Diagnosis of Esophageal Squamous Cell Carcinoma
Source: Cancer Rep (Hoboken). 2026 Nov 8;9(11):e70753. doi: 10.1002/cnr2.70753 (PMC13745642; doi:10.1002/cnr2.70753)
Supplement: Supplementary file 3 [file CNR2-9-e70753-s005.pdf]

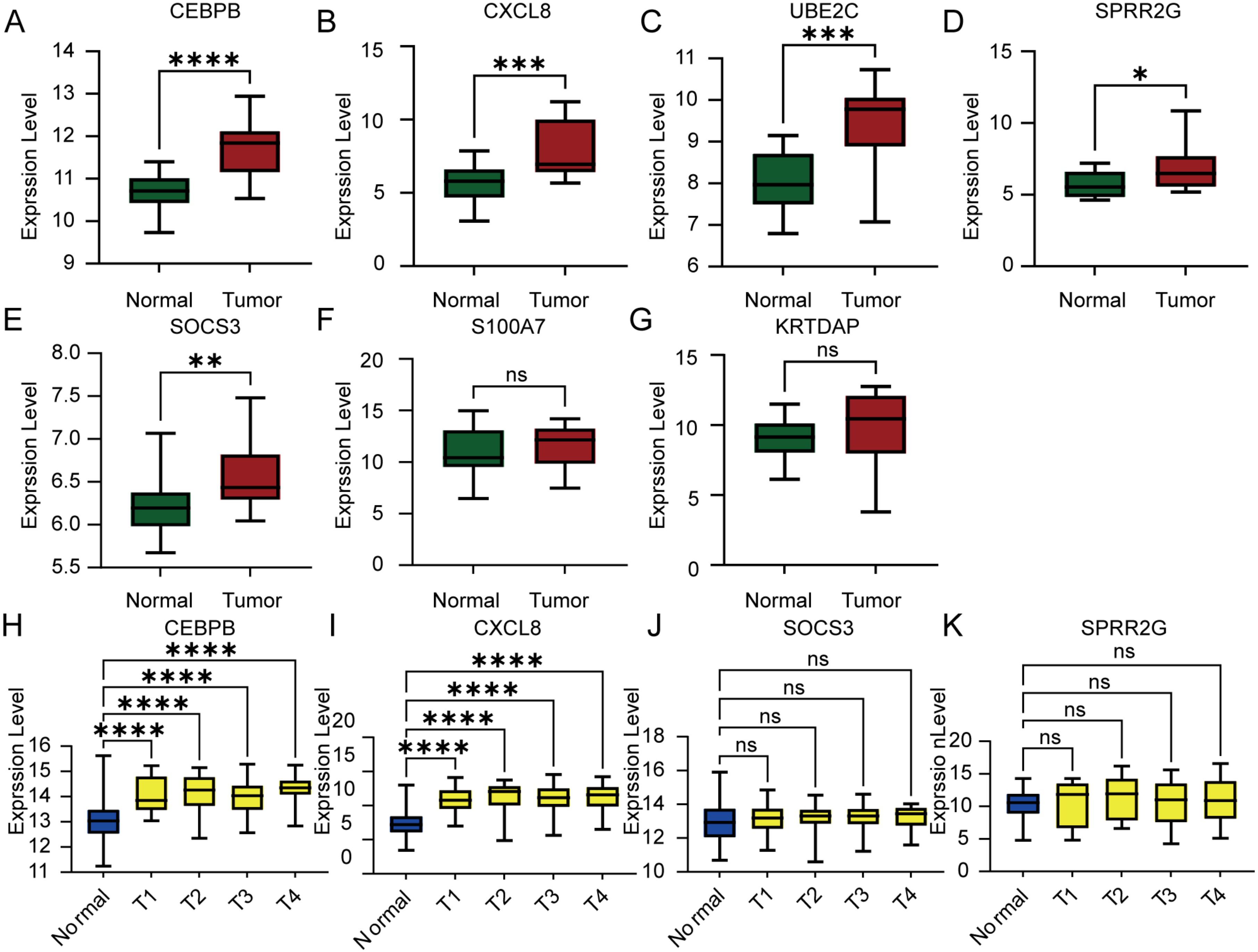

Fig S3. Verification of key gene expression in the validation set
Key gene expression in the GSE17351, GSE44021, and GSE77861 datasets: **A** CEBPB; **B** CXCL8; **C** UBE2C; **D** SPRR2G; **E** SOCS3; **F** S100A7; **G** KRTDAP. Key gene expression in the GSE53625 dataset: **H** CEBPB; **I** CXCL8; **J** SOCS3; **K** SPRR2G. *P < 0.05, **P < 0.01, ***P < 0.001, ****P < 0.0001, ns: nonsignificant.
