# Supplementary material for: Gene Expression Profiling and Clinical Validation Reveal CEBPB for Early Diagnosis of Esophageal Squamous Cell Carcinoma
Source: Cancer Rep (Hoboken). 2026 Nov 8;9(11):e70753. doi: 10.1002/cnr2.70753 (PMC13745642; doi:10.1002/cnr2.70753)
Supplement: Supplementary file 4 [file CNR2-9-e70753-s006.pdf]

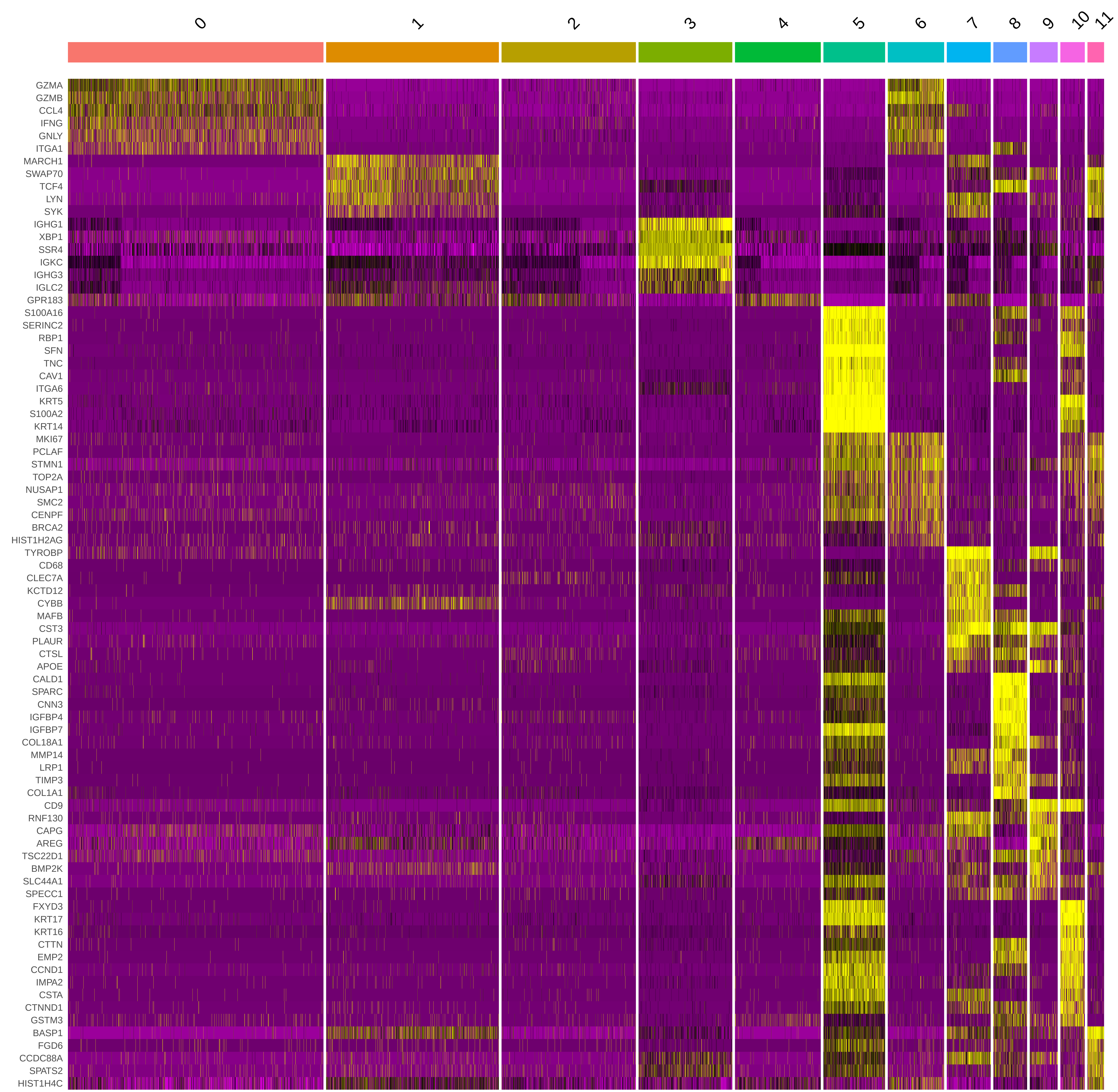
0
1
2
3
4
5
6
7
8
9
10
11
GZMA
GZMB
CCL4
IFNG
GNLY
ITGA1
MARCH1
SWAP70
TCF4
LYN
SYK
IGHG1
XBP1
SSR4
IGKC
IGHG3
IGLC2
GPR183
S100A16
SERINC2
RBP1
SFN
TNC
CAV1
ITGA6
KRT5
S100A2
KRT14
MKI67
PCLAF
STMN1
TOP2A
NUSAP1
SMC2
CENPF
BRCA2
HIST1H2AG
TYROBP
CD68
CLEC7A
KCTD12
CYBB
MAFB
CST3
PLAUR
CTSL
APOE
CALD1
SPARC
CNN3
IGFBP4
IGFBP7
COL18A1
MMP14
LRP1
TIMP3
COL1A1
CD9
RNF130
CAPG
AREG
TSC22D1
BMP2K
SLC44A1
SPECC1
FXYD3
KRT17
KRT16
CTTN
EMP2
CCND1
IMPA2
CSTA
CTNND1
GSTM3
BASP1
FGD6
CCDC88A
SPATS2
HIST1H4C
