# Supplementary material for: Gene Expression Profiling and Clinical Validation Reveal CEBPB for Early Diagnosis of Esophageal Squamous Cell Carcinoma
Source: Cancer Rep (Hoboken). 2026 Nov 8;9(11):e70753. doi: 10.1002/cnr2.70753 (PMC13745642; doi:10.1002/cnr2.70753)
Supplement: Supplementary file 5 [file CNR2-9-e70753-s009.pdf]

**Doc. S1. Clinical characteristics of the study samples and datasets.**

| submitter | additiona | age_at_in | age_began | amount_of | barretts_ | batch_num | bcr | bcr_follo |
|---|---|---|---|---|---|---|---|---|
| TCGA-Z6-A | Complete | 57 | | | | 419.47.0 | Nationwic | TCGA-Z6-A |
| TCGA-L5-A | Progressi | 54 | | 1 | | 272.66.0 | Nationwic | TCGA-L5-A |
| TCGA-IG-A | Complete | 62 | 19 | 1 | No | 374.52.0 | Nationwic | TCGA-IG-A |
| TCGA-LN-A | Progressi | 57 | | 1 | No | 298.64.0 | Nationwic | TCGA-LN-A |
| TCGA-IG-A5B8-01A | | 72 | 23 | 5 | No | 298.64.0 | Nationwide Childre | |
| TCGA-LN-A | Complete | 70 | | 2 | No | 395.50.0 | Nationwic | TCGA-LN-A |
| TCGA-IG-A | Complete | 51 | | | No | 254.66.0 | Nationwic | TCGA-IG-A |
| TCGA-LN-A | Complete | 44 | | | | 272.66.0 | Nationwic | TCGA-LN-A |
| TCGA-VR-A8EW-01A | | 57 | 27 | 1 | No | 374.52.0 | Nationwide Childre | |
| TCGA-LN-A | Complete | 59 | 20 | 2 | No | 362.55.0 | Nationwic | TCGA-LN-A |
| TCGA-L5-A8NK-01A | | 84 | 19 | | No | 382.51.0 | Nationwic | TCGA-L5-A |
| TCGA-JY-A6FE-01A | | 49 | 20 | 1 | No | 339.57.0 | Nationwide Childre | |
| TCGA-VR-A | Complete | 49 | | 5 | No | 374.52.0 | Nationwic | TCGA-VR-A |
| TCGA-Z6-A | Complete | 53 | | | | 382.51.0 | Nationwic | TCGA-Z6-A |
| TCGA-L5-A8NQ-01A | | 71 | 19 | 8 | No | 374.52.0 | Nationwide Childre | |
| TCGA-VR-A | Complete | 60 | 16 | 2 | No | 382.51.0 | Nationwic | TCGA-VR-A |
| TCGA-LN-A9FO-01A | | 42 | | | No | 395.50.0 | Nationwide Childre | |
| TCGA-LN-A | Complete | 46 | | | | 298.64.0 | Nationwic | TCGA-LN-A |
| TCGA-IG-A | Complete | 54 | 24 | 1 | No | 254.66.0 | Nationwic | TCGA-IG-A |
| TCGA-LN-A | Complete | 62 | | 2 | No | 395.50.0 | Nationwic | TCGA-LN-A |
| TCGA-VR-A | Complete | 44 | | | No | 374.52.0 | Nationwic | TCGA-VR-A |
| TCGA-LN-A | Complete | 50 | | 2 | No | 362.55.0 | Nationwic | TCGA-LN-A |
| TCGA-JY-A6FG-01A | | 50 | 25 | 4 | No | 339.57.0 | Nationwide Childre | |
| TCGA-VR-A8ER-01A | | 54 | 10 | 1 | No | 374.52.0 | Nationwide Childre | |
| TCGA-VR-A8EU-01A | | 51 | 21 | 1 | No | 374.52.0 | Nationwide Childre | |
| TCGA-IG-A | Progressi | 48 | 27 | | No | 272.66.0 | Nationwic | TCGA-IG-A |
| TCGA-VR-A8ET-01A | | 64 | | | | 419.47.0 | Nationwide Childre | |
| TCGA-LN-A | Complete | 62 | 22 | 1 | | 272.66.0 | Nationwic | TCGA-LN-A |
| TCGA-IG-A | Complete | 63 | | | | 272.66.0 | Nationwic | TCGA-IG-A |
| TCGA-L5-A88W-01A | | 67 | | 2 | No | 362.55.0 | Nationwide Childre | |
| TCGA-LN-A | Progressi | 49 | | 2 | | 272.66.0 | Nationwic | TCGA-LN-A |
| TCGA-LN-A | Complete | 52 | 23 | 1 | No | 374.52.0 | Nationwic | TCGA-LN-A |
| TCGA-LN-A | Progressi | 60 | | 2 | | 272.66.0 | Nationwic | TCGA-LN-A |
| TCGA-IG-A | Complete | 36 | 16 | 1 | | 395.50.0 | Nationwic | TCGA-IG-A |
| TCGA-Z6-A | Complete | 53 | | | No | 374.52.0 | Nationwic | TCGA-Z6-A |
| TCGA-VR-AA7I-01A | | 70 | 11 | 5 | No | 419.47.0 | Nationwide Childre | |
| TCGA-IG-A625-01A | | 60 | | 1 | No | 323.59.0 | Nationwic | TCGA-IG-A |
| TCGA-JY-A | Complete | 58 | | 0 | No | 382.51.0 | Nationwic | TCGA-JY-A |
| TCGA-LN-A | Complete | 49 | 24 | 1 | No | 362.55.0 | Nationwic | TCGA-LN-A |
| TCGA-LN-A | Progressi | 71 | 31 | | | 254.66.0 | Nationwic | TCGA-LN-A |
| TCGA-S8-A | Complete | 51 | | | No | 323.59.0 | Nationwic | TCGA-S8-A |
| TCGA-IG-A | Complete | 69 | | 1 | | 298.64.0 | Nationwic | TCGA-IG-A |
| TCGA-L5-A | Complete | 84 | 25 | | No | 374.52.0 | Nationwic | TCGA-L5-A |
| TCGA-VR-A8EZ-01A | | 47 | 17 | | No | 374.52.0 | Nationwide Childre | |
| TCGA-LN-A | Complete | 36 | | 1 | | 272.66.0 | Nationwic | TCGA-LN-A |
| TCGA-VR-A8EX-01A | | 63 | 23 | | No | 374.52.0 | Nationwide Childre | |
| TCGA-LN-A | Progressi | 58 | 38 | 2 | | 298.64.0 | Nationwic | TCGA-LN-A |
| TCGA-LN-A | Complete | 62 | | | | 272.66.0 | Nationwic | TCGA-LN-A |
| TCGA-LN-A | Complete | 61 | 26 | | | 272.66.0 | Nationwic | TCGA-LN-A |
| TCGA-LN-A | Progressi | 77 | | 2 | | 272.66.0 | Nationwic | TCGA-LN-A |

| | | | | | | | |
|---|---|---|---|---|---|---|---|
| TCGA-Z6-AComplete | 57 | | | No | 382.51.0 | Nationwic | TCGA-Z6-A |
| TCGA-IG-AComplete | 53 | 24 | 1 | No | 254.66.0 | Nationwic | TCGA-IG-A |
| TCGA-LN-AComplete | 52 | | 2 | | 272.66.0 | Nationwic | TCGA-LN-A |
| TCGA-LN-AComplete | 72 | 23 | 1 | No | 339.57.0 | Nationwic | TCGA-LN-A |
| TCGA-LN-AComplete | 46 | 23 | 1 | No | 323.59.0 | Nationwic | TCGA-LN-A |
| TCGA-LN-AComplete | 54 | 30 | 1 | No | 298.64.0 | Nationwic | TCGA-LN-A |
| TCGA-L5-AProgressi | 90 | 21 | 3 | No | 254.66.0 | Nationwic | TCGA-L5-A |
| TCGA-LN-AComplete | 58 | 28 | 2 | No | 362.55.0 | Nationwic | TCGA-LN-A |
| TCGA-IG-A6QS-01A | 54 | 21 | 1 | No | 339.57.0 | Nationwic | TCGA-IG-A |
| TCGA-L5-A88Z-01A | 70 | | 0 | No | 374.52.0 | Nationwic | TCGA-L5-A |
| TCGA-JY-A6FA-01A | 51 | 16 | 14 | No | 339.57.0 | Nationwide Childre | |
| TCGA-LN-AProgressi | 59 | 19 | | | 254.66.0 | Nationwic | TCGA-LN-A |
| TCGA-IG-AComplete | 61 | | | No | 254.66.0 | Nationwic | TCGA-IG-A |
| TCGA-LN-AComplete | 67 | | 0 | No | 374.52.0 | Nationwic | TCGA-LN-A |
| TCGA-IG-AProgressi | 62 | 31 | 1 | No | 254.66.0 | Nationwic | TCGA-IG-A |
| TCGA-V5-AProgressi | 67 | | 0 | No | 395.50.0 | Nationwic | TCGA-V5-A |
| TCGA-LN-AProgressi | 47 | 27 | 1 | | 254.66.0 | Nationwic | TCGA-LN-A |
| TCGA-XP-AComplete | 54 | 29 | 10 | No | 374.52.0 | Nationwic | TCGA-XP-A |
| TCGA-LN-AProgressi | 60 | | 0 | No | 395.50.0 | Nationwic | TCGA-LN-A |
| TCGA-IC-AComplete | 69 | 25 | 1 | | 339.57.0 | Nationwic | TCGA-IC-A |
| TCGA-IG-AComplete | 58 | | | No | 395.50.0 | Nationwic | TCGA-IG-A |
| TCGA-XP-AComplete | 49 | 12 | 7 | No | 374.52.0 | Nationwic | TCGA-XP-A |
| TCGA-LN-AComplete | 73 | 33 | | | 272.66.0 | Nationwic | TCGA-LN-A |
| TCGA-VR-AComplete | 51 | 12 | | No | 382.51.0 | Nationwic | TCGA-VR-A |
| TCGA-JY-AComplete | 51 | | 0 | No | 339.57.0 | Nationwic | TCGA-JY-A |
| TCGA-IG-A50L-01A | 58 | | | | 272.66.0 | Nationwic | TCGA-IG-A |
| TCGA-KH-AComplete | 82 | | | No | 339.57.0 | Nationwic | TCGA-KH-A |
| TCGA-LN-AComplete | 56 | 26 | 1 | No | 374.52.0 | Nationwic | TCGA-LN-A |
| TCGA-VR-AComplete | 51 | 15 | | No | 419.47.0 | Nationwic | TCGA-VR-A |

| bcr_follc | submitter | city_of_p | clinical_ | clinical_ | clinical_ | clinical_ | columnar_ | columnar_ |
|---|---|---|---|---|---|---|---|---|
| 5CD13BF5- | TCGA-Z6-A | Saint Petersburg | | | | | | |
| B2BD11E4- | TCGA-L5-A | Ann arbor | | | | | N0 | High grad |
| 2EF41295- | TCGA-IG-A | Hanoi | | | | | N0 | Negative/ |
| 8A4D992D- | TCGA-LN-A5U5 | | | | | | | |
| n's Hospi | TCGA-IG-A | Lviv | | | | | N0 | |
| FE4C4142- | TCGA-LN-A9FR | | | | | | | |
| 031734F5- | TCGA-IG-A | Tver | | | | | N0 | |
| 6300106D- | TCGA-LN-A49X | | M0 | N0 | T3 | Stage IIA | | |
| n's Hospi | TCGA-VR-A | Barretos | M0 | N2 | T3 | Stage III | N0 | |
| 8B2DC84D- | TCGA-LN-A7HW | | | | | | | |
| DE93FE27- | TCGA-L5-A | ann arbor | | | | | | |
| n's Hospi | TCGA-JY-A | Toronto | | | | | N0 | Negative/ |
| 9FCE5741- | TCGA-VR-A | Barretos | M0 | N0 | T3 | Stage IIA | N0 | Negative/ |
| D0919D08- | TCGA-Z6-A | Saint Petersburg | | | | | | |
| n's Hospi | TCGA-L5-A | ann arbor | | | | | | |
| DCA6B8EF- | TCGA-VR-A | Barretos | M0 | N1 | T3 | Stage III | N0 | Negative/ |
| n's Hospi | TCGA-LN-A9FO | | | | | | | |
| B5BB323F- | TCGA-LN-A4MQ | | M0 | N1 | T3 | Stage III | | |
| 178E1467- | TCGA-IG-A | Tver | | | | | N0 | |
| 6DE2616E- | TCGA-LN-A9FQ | | | | | | | |
| ED6801CA- | TCGA-VR-A | Barretos | M0 | NX | T3 | Stage IIA | N0 | Negative/ |
| D0EA5792- | TCGA-LN-A7HY | | | | | | | |
| n's Hospi | TCGA-JY-A | Toronto | | | | | N0 | Negative/ |
| n's Hospi | TCGA-VR-A | Barretos | M0 | N1 | T4 | Stage III | N0 | |
| n's Hospi | TCGA-VR-A | Barretos | M1 | N1 | T1 | Stage IV | N0 | Negative/ |
| 835809EF- | TCGA-IG-A | Lviv | | | | | | |
| n's Hospi | TCGA-VR-A | Barretos | M0 | NX | T2 | Stage IIA | N0 | |
| 97B87582- | TCGA-LN-A49U | | M0 | N0 | T3 | Stage IIA | | |
| 3B73A366- | TCGA-IG-A | Tver | M1 | NX | T2 | Stage IV | N0 | |
| n's Hospi | TCGA-L5-A | ann arbor | | | | | N0 | |
| AF2E9B39- | TCGA-LN-A4A5 | | M0 | N0 | T2 | Stage IIA | | |
| 858C5174- | TCGA-LN-A8I0 | | | | | | | |
| 8732F857- | TCGA-LN-A4A1 | | M0 | N0 | T3 | Stage IIA | | |
| 48F9B93F- | TCGA-IG-A | Hanoi | | | | | N0 | Negative/ |
| F2B99F09- | TCGA-Z6-A | Saint petersburg | | | | | | |
| n's Hospi | TCGA-VR-A | Barretos | M0 | N0 | T4 | Stage III | N0 | Negative/ |
| 1EF8D7FA- | TCGA-IG-A | Hanoi | | | | | N0 | Negative/ |
| BC867851- | TCGA-JY-A | Toronto | | | | | N0 | Negative/ |
| DDAAAA80- | TCGA-LN-A7HZ | | | | | | | |
| 29B726A3- | TCGA-LN-A49P | | M0 | N0 | T3 | Stage IIA | | |
| FBEBF1A4- | TCGA-S8-A | Indianapolis | | | | | N0 | Negative/ |
| 19381990- | TCGA-IG-A | Tver | | | | | N0 | |
| E17AB354- | TCGA-L5-A | ann arbor | | | | | N0 | Negative/ |
| n's Hospi | TCGA-VR-A | Barretos | M0 | NX | T3 | Stage IIA | N0 | Negative/ |
| 1203CF76- | TCGA-LN-A4A4 | | M0 | N1 | T3 | Stage III | | |
| n's Hospi | TCGA-VR-A | Barretos | M1a | N1 | T1 | Stage IVA | N0 | Negative/ |
| 294028A9- | TCGA-LN-A4A9 | | M0 | N0 | T2 | Stage IIA | | |
| 28BE31BF- | TCGA-LN-A49M | | M0 | N0 | T2 | Stage IIA | | |
| 60A7D692- | TCGA-LN-A4A3 | | M0 | N1 | T3 | Stage III | | |
| B2C45760- | TCGA-LN-A49Y | | M0 | N0 | T3 | Stage IIA | | |

| | | | | | | | |
|---|---|---|---|---|---|---|---|
| 8DC79E31-TCGA-Z6-ASaint pet | M0 | N1 | T3 | Stage IIIA | | |
| BBFAEBD2-TCGA-IG-ATver | | | | | NO | |
| 898A9BCE-TCGA-LN-A4A8 | M0 | N0 | T2 | Stage IIA | | |
| 20C2CD50-TCGA-LN-A7HX | | | | | | |
| EB1763EB-TCGA-LN-A5U7 | | | | | | |
| 81049F76-TCGA-LN-A5U6 | | | | | | |
| A032BC6D-TCGA-L5-AAnn Arbor | | | | | NO | Negative/ |
| C645A6FC-TCGA-LN-A7HV | | | | | | |
| 6E54C66A-TCGA-IG-ALVIV | | | | | NO | |
| B5254507-TCGA-L5-Aann arbor | | | | | NO | Negative/ |
| n's HospiTCGA-JY-AToronto | | | | | YES | High grad |
| 6BAAA38E-TCGA-LN-A49S | M0 | N0 | T3 | Stage IIA | | |
| F10FA528-TCGA-IG-ATver | | | | | NO | |
| DABA8495-TCGA-LN-A8I1 | | | | | | |
| 9DD01E15-TCGA-IG-ATver | | | | | NO | |
| FF49687E-TCGA-V5-ADurham | | | | | NO | |
| A5B84371-TCGA-LN-A49O | M0 | N0 | T3 | Stage IIA | | |
| E1882817-TCGA-XP-A8T6 | | | | | NO | High grad |
| B30422F6-TCGA-LN-A9FP | | | | | | |
| A06DBA78-TCGA-IC-APittsburg | M0 | N1 | T1 | Stage IIB | | |
| 65148602-TCGA-IG-ALviv | | | | | NO | |
| DD0AAC68-TCGA-XP-A8T8 | M0 | N1 | T1 | Stage IIE | NO | High grad |
| E8F98A3A-TCGA-LN-A49W | M0 | N1 | T3 | Stage III | | |
| 10B4A3E4-TCGA-VR-ABarretos | M0 | N2 | T2 | Stage III | NO | Negative/ |
| 74319F68-TCGA-JY-AToronto | | | | | NO | Negative/ |
| 8B31477E-TCGA-IG-AHanoi | M0 | N1 | T3 | Stage III | NO | |
| 86A3A5C9-TCGA-KH-ANew York | M0 | N0 | T1 | Stage IA | NO | |
| 5C653A16-TCGA-LN-A8HZ | | | | | | |
| 7FBAEAB7-TCGA-VR-ABarretos | M0 | NX | T3 | Stage IIE | NO | |

| columnar_ | country_c | country_c | day_of_dc | day_of_fc | days_to_i | esophagea | file_uuid | frequency |
|---|---|---|---|---|---|---|---|---|
| | Russia | Russia | 22 | 24 | 0 | Distal | 2558374A-AD81-420F | |
| NO | | United St | 22 | 18 | 0 | Distal | AC3740F7- | 7 |
| NO | Vietnam | Vietnam | 22 | 12 | 0 | Distal | E05F0F59- | 7 |
| | Vietnam | Vietnam | 22 | 22 | 0 | Distal | 63776EAC- | 2 |
| NO | Ukraine | Ukraine | 22 | | 0 | Distal | 65034689- | 5 |
| | Vietnam | Vietnam | 22 | 22 | 0 | Proximal | 6D8A4D99- | 7 |
| NO | Russia | Russia | 22 | 31 | 0 | Distal | F036DB74-06C5-48B6 | |
| | Vietnam | Vietnam | 22 | 15 | 0 | Mid | 885EB1D5-BEDF-4F5C | |
| NO | Brazil | Brazil | 22 | | 0 | Mid | 22679454- | 1 |
| | Vietnam | Vietnam | 22 | 15 | 0 | Mid | 849703F5- | 7 |
| | | United St | 22 | 19 | 0 | Mid | 5F08410B-0A0D-47A1 | |
| NO | | Canada | 22 | | 0 | Distal | 9CAF3B78- | 7 |
| NO | Brazil | Brazil | 22 | 21 | 0 | Distal | A2F9566B- | 5 |
| | Russia | Russia | 22 | 24 | 0 | Distal | 103C7DB0-AF17-41E9 | |
| | | United St | 22 | | 0 | Mid | 8F7C7B0F- | 7 |
| NO | Brazil | Brazil | 22 | 10 | 0 | Distal | D765A887- | 7 |
| | Vietnam | Vietnam | 22 | | 0 | Distal | 74878085-F1E2-468E | |
| | Vietnam | Vietnam | 22 | 15 | 0 | Distal | 49832AD5-21D0-4E6E | |
| NO | Russia | Russia | 22 | 26 | 0 | Distal | 353979EE- | 2 |
| | Vietnam | Vietnam | 22 | 21 | 0 | Mid | 579C2395- | 7 |
| NO | Brazil | Brazil | 22 | 30 | 0 | Distal | EC9AB418-167B-4038 | |
| | Vietnam | Vietnam | 22 | 15 | 0 | Proximal | 6928B65E- | 7 |
| 'no dysplasia | | Canada | 22 | | 0 | Distal | 58620E57- | 7 |
| NO | Brazil | Brazil | 22 | | 0 | Distal | 95F31D29- | 1 |
| NO | Brazil | Brazil | 22 | | 0 | Mid | 51B8796D- | 7 |
| | Ukraine | Ukraine | 22 | 19 | 0 | Mid | 77F27762-43C2-4CF6 | |
| | Brazil | Brazil | 22 | | 0 | Distal | 5F0E9E81- | 3 |
| | Vietnam | Vietnam | 22 | 22 | 0 | Mid | 87F54FBF- | 7 |
| NO | Russia | Russia | 22 | 11 | 0 | Proximal | 67CE2037-2B6C-4AFE | |
| NO | | United St | 22 | | 0 | Distal | 02A2FD9F- | 7 |
| | Vietnam | Vietnam | 22 | 15 | 0 | Mid | 4CB799C6- | 7 |
| | Vietnam | Vietnam | 22 | 21 | 0 | Distal | FF41E92C- | 7 |
| | Vietnam | Vietnam | 22 | 15 | 0 | Mid | EC9C437D- | 7 |
| NO | Vietnam | Vietnam | 22 | 12 | 0 | Distal | FA184235- | 4 |
| | Russia | Russia | 22 | 24 | 0 | Mid | D20A4E4F-6843-4776 | |
| NO | Brazil | Brazil | 22 | | 0 | Mid | B564EDDE- | 7 |
| NO | Vietnam | Vietnam | 22 | 3 | 0 | Distal | 5534C8E4- | 7 |
| 'no dysplasia | | Canada | 22 | 16 | 0 | Distal | 695C942B- | 0 |
| | Vietnam | Vietnam | 22 | 15 | 0 | Proximal | 4311E87E- | 7 |
| | Vietnam | Vietnam | 22 | 20 | 0 | Distal | 2DD89B26-B7FE-4B5E | |
| NO | United St | United St | 22 | 21 | 0 | | ED1BBE01-8326-4BB0 | |
| | Russia | Russia | 22 | 11 | 0 | Proximal | F337A28F- | 7 |
| NO | | United St | 22 | 19 | 0 | Distal | F98FEBB8-DF64-4A37 | |
| NO | Brazil | Brazil | 22 | | 0 | Distal | 3EF70A3A-856E-4084 | |
| | Vietnam | Vietnam | 22 | 15 | 0 | Distal | D833214E- | 7 |
| NO | Brazil | Brazil | 22 | | 0 | Mid | 2B8284E6-FAD0-4EE5 | |
| | Vietnam | Vietnam | 22 | 15 | 0 | Mid | C941057C- | 7 |
| | Vietnam | Vietnam | 22 | 19 | 0 | Distal | 2227DB13-839F-4E66 | |
| | Vietnam | Vietnam | 22 | 15 | 0 | Mid | 6FFCED30-96C0-4183 | |
| | Vietnam | Vietnam | 22 | 15 | 0 | Mid | 3EAA856E- | 7 |

| | | | | | | | | |
|---|---|---|---|---|---|---|---|---|
| | Russia | Russia | 22 | 24 | 0 | Proximal | A222F6D2-13C5-4EDB | |
| NO | Russia | Russia | 22 | 11 | 0 | Distal | 75DF2D53- | 1 |
| | Vietnam | Vietnam | 22 | 30 | 0 | Mid | 37898EBF- | 7 |
| | Vietnam | Vietnam | 22 | 19 | 0 | Mid | 375710FC- | 7 |
| | Vietnam | Vietnam | 22 | 15 | 0 | Mid | 106E6F7F- | 7 |
| | Vietnam | Vietnam | 22 | 22 | 0 | Distal | 6F51BCCE- | 3 |
| NO | | United St | 22 | 24 | 0 | Mid | 848FB277- | 7 |
| | Vietnam | Vietnam | 22 | 22 | 0 | Mid | 0691CE37- | 7 |
| | Ukraine | Ukraine | 22 | 1 | 0 | Distal | 57FA9EB5- | 3 |
| NO | | United St | 22 | 19 | 0 | Mid | EB9468CF- | 0 |
| NO | | Canada | 22 | | 0 | Distal | A865BB9C- | 7 |
| | Vietnam | Vietnam | 22 | 15 | 0 | Distal | 7127F2D2-F7D6-42F3 | |
| NO | Russia | Russia | 22 | 11 | 0 | Distal | 6325DD0B-2620-423D | |
| | Vietnam | Vietnam | 22 | 16 | 0 | Mid | DCC53611- | 0 |
| NO | Russia | Russia | 22 | 11 | 0 | Distal | E3DDA0F1- | 2 |
| NO | United St | United St | 22 | 4 | 0 | Mid | 2F37B5EA- | 0 |
| | Vietnam | Vietnam | 22 | 15 | 0 | Mid | 79B5A5FA- | 7 |
| NO | Brazil | | 22 | 26 | 0 | Distal | 79F522DE- | 7 |
| | Vietnam | Vietnam | 22 | 19 | 0 | Mid | EDF38024- | 0 |
| | | United St | 22 | 13 | 0 | Mid | 12269AAB- | 1 |
| | Ukraine | Ukraine | 22 | 29 | 0 | Distal | B6ECBBFA-6057-431B | |
| NO | Brazil | | 22 | 28 | 0 | Mid | C453D8BF- | 7 |
| | Vietnam | Vietnam | 22 | 15 | 0 | Mid | 2A064650-A9C9-48FE | |
| NO | Brazil | Brazil | 22 | 21 | 0 | Mid | 7AB3844A-2713-4A1B | |
| NO | | Canada | 22 | 23 | 0 | Distal | 8CE178E3- | 0 |
| NO | Vietnam | Vietnam | 22 | 6 | 0 | Distal | 9C910CB9-8D4F-421E | |
| NO | United St | United St | 22 | 16 | 0 | Mid | CE3CC895- | 3 |
| | Vietnam | Vietnam | 22 | 16 | 0 | Mid | 4819E0BA- | 7 |
| | Brazil | Brazil | 22 | 30 | 0 | Mid | D8A9D96D-3B81-42D9 | |

| goblet_ce | h_pylori_ | history_c | history_c | informed_ | init_path | initial_d | initial_p | lost_foll |
|---|---|---|---|---|---|---|---|---|
| -BCA5-F2E61B4F7927 | | | No | YES | | | | NO |
| | | NO | No | YES | | Symptomat | Endoscopi | NO |
| | | NO | No | YES | | | Endoscopi | NO |
| | Never | NO | No | YES | | Symptomat | Endoscopi | NO |
| | | NO | No | YES | | Symptomat | Endoscopic Biopsy | |
| | Never | NO | No | YES | Tumor Res | Symptomat | Other met | NO |
| -B5F2-70B50C3C00EF | | NO | No | YES | Surgical | Symptomat | Other met | NO |
| -9416-2116247A0DF6 | | NO | No | YES | | | Surgical | NO |
| NO | Never | NO | No | YES | | Symptomat | Endoscopic Biopsy | |
| | Never | NO | No | YES | Surgical | Symptomat | Other met | NO |
| -8E1B-92074F7A3D85 | | NO | No | YES | cytology | Symptomat | Other met | YES |
| | Never | NO | No | YES | | Symptomat | Endoscopic Biopsy | |
| NO | Never | NO | No | YES | | Symptomat | Endoscopi | NO |
| -9B2B-E818CB85F583 | | | No | YES | | | | NO |
| | | | No | YES | | Symptomat | Endoscopic Biopsy | |
| | Never | NO | No | YES | | Symptomat | Endoscopi | NO |
| -8E29-708 | Never | NO | No | YES | | Symptomat | Endoscopic Biopsy | |
| -9BC9-0C5EF6140A79 | | NO | No | YES | | | Surgical | NO |
| | | NO | No | YES | Surgical | Symptomat | Other met | NO |
| | Never | NO | No | YES | Tumor Res | Symptomat | Other met | NO |
| NO | Never | NO | No | YES | | Symptomat | Endoscopi | NO |
| | Never | NO | No | YES | Surgical | Symptomat | Other met | NO |
| | Never | NO | No | YES | | Symptomat | Endoscopic Biopsy | |
| | Never | NO | No | YES | | Symptomat | Endoscopic Biopsy | |
| | | YES | No | YES | | Symptomat | Endoscopic Biopsy | |
| -B760-11EB3BA27BA6 | | NO | No | YES | | | Surgical | NO |
| | | YES | No | YES | | | Endoscopic Biopsy | |
| | | NO | No | YES | | | Surgical | NO |
| -8729-60442B840C46 | | NO | No | YES | | | Surgical | NO |
| | | NO | No | YES | | Symptomat | Endoscopic Biopsy | |
| | | NO | No | YES | | | Surgical | NO |
| | Never | NO | No | YES | Tumor Res | Symptomat | Other met | NO |
| | | NO | No | YES | | | Surgical | NO |
| | | NO | No | YES | | | Endoscopi | NO |
| NO | | | No | YES | Surgical | Symptomat | Other met | NO |
| NO | Never | | No | YES | | Symptomat | Endoscopic Biopsy | |
| | | NO | No | YES | | | Endoscopi | NO |
| | Never | NO | No | YES | | Symptomat | Endoscopi | NO |
| | Never | NO | No | YES | Surgical | Symptomat | Other met | NO |
| -BB49-92DF1BCA2406 | | NO | No | YES | | | Surgical | NO |
| -B5FF-C17 | Never | NO | No | YES | | Symptomat | Endoscopi | NO |
| | | NO | No | YES | Surgical | resection | Other met | NO |
| -BEF4-CED49C656180 | | NO | No | YES | | Symptomat | Endoscopi | NO |
| -8AC1-ACA | Never | NO | No | YES | | Symptomat | Endoscopic Biopsy | |
| | | NO | No | YES | | | Surgical | NO |
| -AA62-19A | Never | NO | No | YES | | Symptomat | Endoscopic Biopsy | |
| | | NO | No | YES | | | Surgical | NO |
| -997C-9205286FF20A | | NO | No | YES | | | Surgical | NO |
| -94A6-E5A5D686E4AB | | NO | No | YES | | | Surgical | NO |
| | | NO | No | YES | | | Surgical | NO |

| | | | | | | | | |
|---|---|---|---|---|---|---|---|---|
| -ABBD-58135C9D5D23 | | | No | YES | Surgical | Symptomat | Other met | NO |
| | | NO | No | YES | | Symptomat | Surgical | NO |
| | | NO | No | YES | | | Surgical | NO |
| | Never | NO | No | YES | Surgical | Symptomat | Other met | NO |
| | Never | NO | No | YES | | Symptomat | Endoscopi | NO |
| | Never | NO | No | YES | | Symptomat | Endoscopi | NO |
| | | YES | No | YES | | Symptomat | Surgical | NO |
| | | NO | No | YES | Surgical | Symptomat | Other met | NO |
| | | NO | No | YES | | Symptomat | Endoscopi | NO |
| | | NO | No | YES | | Symptomat | Endoscopi | YES |
| | Never | NO | No | YES | | Symptomat | Endoscopic Biopsy | |
| -9318-B1B64F194BD4 | | NO | No | YES | | | Surgical | NO |
| -B991-338D93482E7E | | NO | No | YES | | Symptomat | Surgical | YES |
| | Never | NO | No | YES | Tumor Res | Symptomat | Other met | NO |
| | | NO | No | YES | Surgical | Symptomat | Other met | NO |
| | Never | NO | No | YES | | Symptomat | Endoscopi | NO |
| | | NO | No | YES | | | Surgical | NO |
| | Never | NO | No | YES | | Symptomat | Endoscopi | NO |
| | Never | NO | No | YES | Tumor Res | Symptomat | Other met | NO |
| | | | No | YES | | | Endoscopi | NO |
| -8902-0DB | Never | NO | No | YES | Surgery | Symptomat | Other met | NO |
| | Never | NO | No | YES | | Symptomat | Endoscopi | YES |
| -BF0F-B1840B43B51A | | NO | No | YES | | | Surgical | NO |
| -B381-550 | Never | NO | No | YES | | Symptomat | Endoscopi | NO |
| NO | Never | NO | No | YES | | Symptomat | Endoscopi | NO |
| -88C9-4F82894B21B2 | | NO | No | YES | | | Endoscopi | YES |
| | Never | NO | No | YES | | Symptomat | Endoscopi | NO |
| | | NO | No | YES | Tumor Res | Symptomat | Other met | NO |
| -B95A-800 | Current | YES | No | YES | | Symptomat | Endoscopi | NO |

| lymph_noc | lymph_nod | month_of_ | month_of_ | neoplasm_ | number_of | number_of | number_of | number_pa |
|---|---|---|---|---|---|---|---|---|
| 11 | | 12 | 12 | G1 | 0 | | | 1 |
| 3 | NO | 12 | 12 | GX | 0 | | | 40 |
| 20 | YES | 12 | 12 | G3 | 6 | | | 15 |
| 8 | | 12 | 1 | G2 | 5 | | | |
| 1 | NO | 12 | | G1 | 0 | | | 75 |
| 2 | NO | 12 | 1 | G2 | 1 | | | |
| 5 | YES | 12 | 10 | G2 | 0 | 0 | | |
| | | 12 | 1 | G2 | | | 0 | |
| 13 | YES | 12 | | G2 | 4 | 4 | | 10 |
| | NO | 12 | 1 | G2 | | | | 40 |
| 14 | NO | 12 | 12 | G1 | 0 | 0 | | 25 |
| 35 | YES | 12 | | G3 | 8 | | | 29 |
| 8 | NO | 12 | 9 | G3 | 0 | | | |
| | | 12 | 12 | G2 | | | | |
| 3 | NO | 12 | | G3 | 0 | 0 | | 60 |
| 87 | YES | 12 | 9 | G3 | 3 | 3 | | 13.5 |
| | NO | 12 | | G2 | | | | |
| 2 | | 12 | 1 | G3 | 2 | | | |
| 7 | NO | 12 | 2 | G2 | 0 | 0 | | 30 |
| | NO | 12 | 1 | G3 | | | | |
| 32 | NO | 12 | 10 | G1 | 0 | | | |
| 1 | | 12 | 1 | G2 | 1 | | | |
| 18 | YES | 12 | | G2 | 5 | | | 6 |
| 14 | YES | 12 | | GX | 3 | 3 | | 12.25 |
| 14 | NO | 12 | | G2 | 2 | 2 | | 10 |
| 15 | | 12 | 3 | G2 | 2 | | | 20 |
| 17 | YES | 12 | | G2 | 0 | | 1 | |
| | | 12 | 1 | G1 | | | 0 | 40 |
| 1 | | 12 | 12 | G2 | 1 | | | |
| 8 | YES | 12 | | GX | 0 | 0 | | |
| | | 12 | 1 | G2 | | | 0 | |
| 2 | NO | 12 | 1 | G2 | 0 | | | 15 |
| | | 12 | 1 | G3 | | | 0 | |
| | YES | 12 | 12 | G3 | | | | 10 |
| 19 | NO | 12 | 12 | G2 | 0 | 0 | | |
| 15 | NO | 12 | | G1 | 0 | | | 20 |
| 8 | YES | 12 | 3 | G2 | 5 | | | |
| 39 | NO | 12 | 3 | G1 | 0 | 0 | | |
| | NO | 12 | 1 | G2 | | | | 25 |
| | | 12 | 1 | G2 | | | 0 | 40 |
| 22 | | 12 | 8 | G1 | 0 | 0 | | |
| 2 | | 12 | 8 | G2 | 0 | | | |
| 8 | NO | 12 | 12 | GX | 0 | 0 | | 30 |
| 28 | YES | 12 | | G2 | 7 | | | 45 |
| 2 | | 12 | 1 | GX | 2 | | 0 | |
| 34 | NO | 12 | | G2 | 9 | 9 | | 10 |
| | | 12 | 1 | G1 | | | 0 | 20 |
| | | 12 | 1 | G1 | | | 0 | |
| 2 | | 12 | 1 | G2 | 2 | | 0 | 35 |
| | | 12 | 1 | G2 | | | 0 | |

| | | | | | | | | |
|---|---|---|---|---|---|---|---|---|
| 9 | NO | 12 | 12 | G3 | 1 | 0 | | |
| 4 | YES | 12 | 12 | G2 | 0 | 0 | | 37.5 |
| | | 12 | 1 | G2 | | | 0 | |
| | NO | 12 | 1 | G2 | | | | 50 |
| | NO | 12 | 1 | G1 | | | | 23 |
| 2 | | 12 | 1 | G2 | 1 | | | 24 |
| 3 | NO | 12 | 3 | G3 | 0 | 0 | 1 | 54 |
| | NO | 12 | 1 | G1 | | | | 15 |
| 10 | NO | 12 | 5 | G1 | 2 | | | 34 |
| 8 | NO | 12 | 12 | GX | 2 | 0 | | |
| 19 | NO | 12 | | G2 | 4 | | | 35 |
| | | 12 | 1 | G2 | | | 0 | 40 |
| 3 | YES | 12 | 12 | G2 | 1 | | | |
| | NO | 12 | 1 | G3 | | | | |
| 5 | YES | 12 | 12 | G2 | 1 | 0 | | 40 |
| | NO | 12 | 12 | G3 | | | 0 | 30 |
| | | 12 | 1 | G3 | | | 0 | 20 |
| 14 | NO | 12 | 1 | G2 | 3 | | | 25 |
| | NO | 12 | 1 | G3 | | | | |
| 2 | | 12 | 1 | GX | 0 | | | 22 |
| 4 | | 12 | 9 | G2 | 0 | | | |
| 10 | YES | 12 | 1 | G1 | 1 | | | 19 |
| 2 | | 12 | 1 | G3 | 2 | | 0 | 40 |
| 25 | YES | 12 | 9 | G2 | 1 | | | 10 |
| 26 | NO | 12 | 5 | G1 | 0 | | | |
| 1 | YES | 12 | 11 | G2 | 1 | | | 31 |
| | NO | 12 | 9 | GX | | | 0 | 50 |
| | NO | 12 | 1 | G3 | | | | 30 |
| 27 | NO | 12 | 10 | G3 | 4 | | 1 | 54 |

| other_dx | pathologi | pathologi | pathologi | patient_i | person_ne | planned_s | postopera | primary_l |
|---|---|---|---|---|---|---|---|---|
| No | M0 | N0 | T3 | AAPN | TUMOR FREE | | NO | YES |
| No | | N0 | T1 | A40M | | | NO | YES |
| No | M0 | N2 | T3 | A802 | | Surgery already pe | | YES |
| No | M1 | N1 | T3 | A5U5 | WITH TUMOR | | NO | YES |
| No | M0 | N0 | T3 | A5B8 | | | | YES |
| No | M0 | N1 | T2 | A9FR | TUMOR FREE | | YES | YES |
| No | M0 | N0 | T3 | A3I8 | TUMOR FREE | | YES | YES |
| No | M0 | N0 | T3 | A49X | TUMOR FRE | Surgery a | NO | NO |
| No | M0 | N2 | T3 | A8EW | | Surgery already pe | | YES |
| No | M0 | N0 | T2 | A7HW | TUMOR FREE | | NO | NO |
| No | M0 | N0 | T3 | A8NK | | | | YES |
| No | M0 | N1 | T3 | A6FE | | | | YES |
| No | M0 | N0 | T3 | A8E0 | TUMOR FRE | Surgery a | NO | YES |
| No | M0 | N1 | T3 | A9VB | TUMOR FREE | | NO | |
| No | M0 | N0 | T2 | A8NQ | | | | YES |
| No | M0 | N1 | T3 | A8Q7 | TUMOR FRE | Surgery a | NO | YES |
| No | M0 | N0 | T2 | A9F0 | | | | NO |
| No | M0 | N1 | T3 | A4MQ | TUMOR FRE | Surgery a | NO | YES |
| No | M0 | N0 | T2 | A3QL | TUMOR FREE | | NO | YES |
| No | M0 | N0 | T3 | A9FQ | TUMOR FREE | | NO | NO |
| No | M0 | N0 | T3 | A8EY | TUMOR FRE | Surgery a | NO | YES |
| No | M0 | N1 | T3 | A7HY | TUMOR FREE | | NO | YES |
| No | M0 | N1 | T3 | A6FG | | | | YES |
| No | M0 | N1 | T4 | A8ER | | Surgery already pe | | YES |
| No | M1 | N1 | T1 | A8EU | | Surgery already pe | | YES |
| No | M0 | N1 | T2 | A4P3 | | Surgery a | NO | YES |
| No | M0 | N0 | T2 | A8ET | | Surgery already pe | | YES |
| No | M0 | N0 | T3 | A49U | TUMOR FRE | Surgery a | YES | NO |
| No | M0 | N1 | T1 | A51D | TUMOR FRE | Yes | NO | YES |
| No | MX | N0 | T3 | A88W | | | | YES |
| No | M0 | N0 | T2 | A4A5 | WITH TUMO | Surgery a | NO | NO |
| No | M0 | N0 | T2 | A8I0 | TUMOR FREE | | NO | YES |
| No | M0 | N0 | T3 | A4A1 | WITH TUMO | Surgery a | NO | NO |
| No | M0 | NX | T3 | A97H | TUMOR FREE | | NO | NO |
| No | M0 | N0 | T3 | A8JD | TUMOR FREE | | NO | YES |
| No | M0 | N0 | T4 | AA7I | | Surgery already pe | | YES |
| No | M0 | N2 | T3 | A625 | | Surgery a | NO | YES |
| No | M0 | N0 | T2 | A93F | TUMOR FREE | | NO | YES |
| No | M0 | N0 | T2 | A7HZ | TUMOR FREE | | NO | NO |
| No | M0 | N0 | T3 | A49P | WITH TUMO | Surgery a | NO | NO |
| No | MX | N0 | T2 | A6BW | TUMOR FRE | Surgery a | NO | YES |
| No | M0 | N0 | T3 | A5S3 | TUMOR FREE | | NO | YES |
| No | MX | N0 | T3 | A88S | TUMOR FREE | | NO | YES |
| No | M0 | N3 | T3 | A8EZ | | Surgery already pe | | YES |
| No | M0 | N1 | T3 | A4A4 | TUMOR FRE | Surgery a | NO | YES |
| No | M1a | N1 | T1 | A8EX | | Surgery already pe | | YES |
| No | M0 | N0 | T2 | A4A9 | WITH TUMO | Surgery a | NO | NO |
| No | M0 | N0 | T2 | A49M | TUMOR FRE | Surgery a | YES | NO |
| No | M0 | N1 | T3 | A4A3 | TUMOR FRE | Surgery a | YES | YES |
| No | M0 | N0 | T3 | A49Y | WITH TUMO | Surgery a | NO | NO |

| | | | | | | | | |
|---|---|---|---|---|---|---|---|---|
| No | M0 | N1 | T3 | A8JE | TUMOR FRE | Surgery a | NO | YES |
| No | M0 | N0 | T4 | A3YA | TUMOR FREE | | NO | YES |
| No | M0 | N0 | T2 | A4A8 | TUMOR FRE | Surgery a | NO | NO |
| No | M0 | N0 | T2 | A7HX | TUMOR FREE | | YES | NO |
| No | M0 | N0 | T2 | A5U7 | TUMOR FREE | | NO | NO |
| No | M0 | N1 | T2 | A5U6 | TUMOR FREE | | YES | YES |
| Yes | MX | N0 | T3 | A43J | WITH TUMOR | | | YES |
| No | M0 | N0 | T2 | A7HV | TUMOR FREE | | YES | NO |
| No | M0 | N1 | T2 | A6QS | | Surgery a | NO | YES |
| No | M0 | N1 | T1 | A88Z | | | | YES |
| No | M0 | N1 | T2 | A6FA | | | | YES |
| No | M0 | N0 | T3 | A49S | WITH TUMC | Surgery a | NO | NO |
| No | M0 | N1 | T3 | A3YB | TUMOR FREE | | NO | YES |
| No | M0 | N0 | T2 | A8I1 | TUMOR FREE | | NO | NO |
| No | M0 | N1 | T3 | A3YC | WITH TUMOR | | NO | YES |
| No | MX | N0 | T3 | AASV | WITH TUMOR | | YES | YES |
| No | M0 | N0 | T3 | A49O | WITH TUMC | Surgery a | YES | NO |
| No | M0 | N1 | T2 | A8T6 | TUMOR FRE | Yes | NO | YES |
| No | M0 | N0 | T2 | A9FP | WITH TUMOR | | YES | NO |
| Yes | M0 | N0 | T1 | A6RF | TUMOR FRE | Surgery a | NO | YES |
| No | M0 | N0 | T2 | A97I | TUMOR FREE | | NO | YES |
| No | M0 | N1 | T1 | A8T8 | TUMOR FRE | Surgery a | YES | YES |
| No | M0 | N1 | T3 | A49W | TUMOR FRE | Surgery a | YES | YES |
| No | M0 | N2 | T2 | AA4G | TUMOR FRE | Surgery a | NO | YES |
| No | M0 | N0 | T3 | A6FD | TUMOR FREE | | NO | YES |
| No | M0 | N1 | T3 | A50L | | Surgery already pe | | YES |
| No | M0 | N0 | T1 | A6WC | TUMOR FRE | No | YES | NO |
| No | M0 | N0 | T2 | A8HZ | TUMOR FREE | | YES | NO |
| No | M0 | N2 | T3 | A8EP | TUMOR FRE | Surgery a | NO | YES |

| primary_t | radiation | reflux_hi | state_prc | stopped_s | system_ve | tissue_pr | tissue_re | tissue_sc |
|---|---|---|---|---|---|---|---|---|
| Complete | NO | | | | 7th | YES | NO | Z6 |
| Complete | NO | NO | Mi | | 7th | NO | YES | L5 |
| Complete Remission | | NO | Hanoi | 2000 | 7th | YES | NO | IG |
| Progressi | NO | NO | | | 6th | YES | NO | LN |
| | | NO | | | 7th | YES | NO | IG |
| Complete | YES | NO | | | 6th | YES | NO | LN |
| Complete | NO | | | | 7th | YES | NO | IG |
| Complete | YES | NO | | | 6th | YES | NO | LN |
| | | NO | Sao Paulo | | 7th | NO | YES | VR |
| Complete | NO | NO | | 2012 | 6th | YES | NO | LN |
| | | YES | michigan | 1989 | 6th | NO | YES | L5 |
| | | | Ontario | 2009 | 6th | NO | YES | JY |
| Complete | YES | YES | Sao Paulo | | 7th | NO | YES | VR |
| Complete | NO | | | | 7th | YES | NO | Z6 |
| | | YES | michigan | 1979 | 5th | NO | YES | L5 |
| Complete | NO | NO | Sao Paulo | | 7th | NO | YES | VR |
| | | NO | | | 6th | YES | NO | LN |
| Complete | NO | NO | | | 6th | YES | NO | LN |
| Complete | NO | | | | 7th | YES | NO | IG |
| Complete | NO | NO | | | 6th | YES | NO | LN |
| Complete | NO | NO | Sao Paulo | | 7th | NO | YES | VR |
| Complete | NO | NO | | | 6th | YES | NO | LN |
| | | | Ontario | 2008 | 6th | NO | YES | JY |
| | | NO | Sao Paulo | | 6th | NO | YES | VR |
| | | YES | Sao Paulo | | 7th | NO | YES | VR |
| Progressi | YES | NO | | 2012 | 7th | YES | NO | IG |
| | | | Sao Paulo | | 7th | NO | YES | VR |
| Complete | YES | NO | | 2010 | 6th | YES | NO | LN |
| Complete | NO | | | | 7th | YES | NO | IG |
| | | NO | michigan | | 6th | NO | YES | L5 |
| Complete | NO | NO | | | 6th | YES | NO | LN |
| Complete | NO | NO | | | 6th | YES | NO | LN |
| Complete | NO | NO | | | 6th | YES | NO | LN |
| Complete Remission | | NO | | 2013 | 7th | YES | NO | IG |
| Complete | NO | | | | 7th | YES | NO | Z6 |
| | | NO | Sao Paulo | | 7th | NO | YES | VR |
| | | NO | | | 7th | YES | NO | IG |
| Complete | NO | NO | Ontario | | 7th | NO | YES | JY |
| Complete | NO | NO | | | 6th | YES | NO | LN |
| Complete | YES | NO | | 2010 | 6th | YES | NO | LN |
| Complete | NO | YES | Indiana | 1990 | 7th | YES | NO | S8 |
| Complete | NO | | | | 7th | YES | NO | IG |
| Complete | NO | YES | michigan | 1968 | 7th | NO | YES | L5 |
| | | NO | Sao Paulo | | 7th | NO | YES | VR |
| Complete | YES | NO | | | 6th | YES | NO | LN |
| | | NO | Sao Paulo | | 6th | NO | YES | VR |
| Complete | NO | NO | | 2012 | 7th | YES | NO | LN |
| Complete | YES | NO | | | 6th | YES | NO | LN |
| Complete | YES | NO | | | 6th | YES | NO | LN |
| Complete | NO | NO | | | 6th | YES | NO | LN |

| | | | | | | | | |
|---|---|---|---|---|---|---|---|---|
| Complete | NO | | | | 7th | YES | NO | Z6 |
| Complete | NO | | | | 7th | YES | NO | IG |
| Complete | NO | NO | | | 6th | YES | NO | LN |
| Complete | YES | NO | | | 6th | YES | NO | LN |
| Complete | NO | NO | | | 6th | YES | NO | LN |
| Complete | YES | NO | | | 6th | YES | NO | LN |
| Progressive Diseas | | NO | MI | 1958 | 7th | NO | YES | L5 |
| Complete | YES | NO | | | 6th | YES | NO | LN |
| Complete | NO | NO | | | 7th | YES | NO | IG |
| | | YES | michigan | | 6th | NO | YES | L5 |
| | | YES | Ontario | 2006 | 6th | NO | YES | JY |
| Complete | YES | NO | | 2010 | 6th | YES | NO | LN |
| Complete | YES | | | | 7th | YES | NO | IG |
| Complete | YES | NO | | | 6th | YES | NO | LN |
| Stable Di | NO | | | | 7th | YES | NO | IG |
| Partial R | YES | YES | NC | 2003 | 7th | YES | NO | V5 |
| Complete | YES | NO | | 2010 | 6th | YES | NO | LN |
| Complete | NO | NO | | | 6th | | | XP |
| Complete | YES | NO | | | 6th | YES | NO | LN |
| Complete | NO | YES | Pennsylva | 1992 | 7th | YES | NO | IC |
| Complete | YES | NO | | | 7th | YES | NO | IG |
| Complete | YES | NO | | | 7th | NO | YES | XP |
| Complete | YES | NO | | 2010 | 6th | YES | NO | LN |
| Complete | NO | NO | Sao Paulo | | 7th | NO | YES | VR |
| Complete | NO | | Ontario | | 6th | NO | YES | JY |
| | | NO | | 1981 | 7th | YES | NO | IG |
| Complete | YES | NO | New York | 1973 | 7th | YES | NO | KH |
| Complete | YES | NO | | 2013 | 6th | YES | NO | LN |
| Progressi | NO | NO | Sao Paulo | | 7th | NO | YES | VR |

| tobacco_s | treatment | weight | withdrawn | year_of_d | year_of_f | year_of_i | days_to_idb | gap_reg |
|---|---|---|---|---|---|---|---|---|
| 4 | | 74 | FALSE | 2016 | 2014 | 2013 | 0 | |
| 2 | | 69 | FALSE | 2016 | 2014 | 2010 | 0 | |
| 4 | No Treatm | 53 | FALSE | 2016 | 2014 | 2013 | 0 | |
| 1 | | 47 | FALSE | 2016 | 2015 | 2012 | 0 | |
| 2 | | 64 | FALSE | 2016 | | 2012 | 0 | 178 |
| 1 | | 61 | FALSE | 2016 | 2015 | 2013 | 0 | |
| 1 | | 48 | FALSE | 2016 | 2012 | 2011 | 0 | |
| 1 | No Treatment | | FALSE | 2016 | 2015 | 2011 | 0 | |
| 2 | No Treatm | 48 | FALSE | 2016 | | 2012 | 0 | |
| 2 | | 69 | FALSE | 2016 | 2015 | 2013 | 0 | |
| 4 | | 67 | FALSE | 2016 | 2014 | 2004 | 0 | |
| 4 | | 62 | FALSE | 2016 | | 2009 | 0 | |
| | No Treatm | 63 | FALSE | 2016 | 2014 | 2013 | 0 | |
| 1 | | 62 | FALSE | 2016 | 2014 | 2013 | 0 | |
| 3 | | 87 | FALSE | 2016 | | 2001 | 0 | |
| 2 | No Treatm | 57 | FALSE | 2016 | 2014 | 2010 | 0 | |
| 2 | | 65 | FALSE | 2016 | | 2013 | 0 | |
| 2 | No Treatm | 56 | FALSE | 2016 | 2015 | 2010 | 0 | |
| 2 | | 73 | FALSE | 2016 | 2013 | 2011 | 0 | |
| 1 | | 63 | FALSE | 2016 | 2015 | 2013 | 0 | 178 |
| 3 | No Treatm | 70 | FALSE | 2016 | 2014 | 2012 | 0 | |
| 1 | | 65 | FALSE | 2016 | 2015 | 2013 | 0 | |
| 4 | | 110 | FALSE | 2016 | | 2009 | 0 | |
| 2 | No Treatm | 46 | FALSE | 2016 | | 2009 | 0 | |
| 2 | No Treatm | 51 | FALSE | 2016 | | 2011 | 0 | |
| 4 | No Treatm | 86 | FALSE | 2016 | 2014 | 2012 | 0 | 178 |
| 1 | No Treatm | 66 | FALSE | 2016 | | 2010 | 0 | |
| 4 | No Treatm | 64 | FALSE | 2016 | 2015 | 2011 | 0 | |
| 1 | | 63 | FALSE | 2016 | 2013 | 2012 | 0 | 178 |
| | | 80 | FALSE | 2016 | | 2007 | 0 | |
| 1 | No Treatm | 68 | FALSE | 2016 | 2015 | 2011 | 0 | 178 |
| 2 | | 65 | FALSE | 2016 | 2015 | 2013 | 0 | |
| 1 | No Treatm | 65 | FALSE | 2016 | 2015 | 2011 | 0 | |
| 4 | | 59 | FALSE | 2016 | 2014 | 2013 | 0 | |
| 1 | | 69 | FALSE | 2016 | 2014 | 2013 | 0 | |
| 3 | No Treatm | 56 | FALSE | 2016 | | 2010 | 0 | |
| 1 | No Treatm | 50 | FALSE | 2016 | 2014 | 2012 | 0 | 178 |
| 1 | | 73 | FALSE | 2016 | 2015 | 2012 | 0 | |
| 2 | | 62 | FALSE | 2016 | 2015 | 2013 | 0 | |
| 4 | No Treatm | 62 | FALSE | 2016 | 2015 | 2011 | 0 | |
| 3 | No Treatm | 76 | FALSE | 2016 | 2014 | 2012 | 0 | |
| 1 | | 69 | FALSE | 2016 | 2014 | 2012 | 0 | |
| 3 | | 77 | FALSE | 2016 | 2014 | 2013 | 0 | |
| 2 | No Treatm | 69 | FALSE | 2016 | | 2013 | 0 | |
| 1 | No Treatm | 62 | FALSE | 2016 | 2015 | 2011 | 0 | |
| 2 | No Treatm | 60 | FALSE | 2016 | | 2009 | 0 | |
| 4 | No Treatm | 72 | FALSE | 2016 | 2015 | 2012 | 0 | |
| 1 | No Treatm | 57 | FALSE | 2016 | 2015 | 2010 | 0 | |
| 2 | No Treatm | 63 | FALSE | 2016 | 2015 | 2011 | 0 | |
| 1 | No Treatm | 57 | FALSE | 2016 | 2015 | 2011 | 0 | |

| | | | | | | | | |
|---|---|---|---|---|---|---|---|---|
| 1 | No Treatm | 61 | FALSE | 2016 | 2014 | 2013 | 0 | |
| 2 | | 76 | FALSE | 2016 | 2013 | 2012 | 0 | 178 |
| 1 | No Treatm | 68 | FALSE | 2016 | 2015 | 2012 | 0 | |
| 2 | | 67 | FALSE | 2016 | 2015 | 2013 | 0 | 178 |
| 2 | | 53 | FALSE | 2016 | 2015 | 2012 | 0 | 178 |
| 2 | | 60 | FALSE | 2016 | 2015 | 2012 | 0 | |
| 3 | | 61 | FALSE | 2016 | 2015 | 2011 | 0 | |
| 2 | | 72 | FALSE | 2016 | 2015 | 2012 | 0 | |
| 2 | No Treatm | 70 | FALSE | 2016 | 2014 | 2013 | 0 | |
| 1 | | 66 | FALSE | 2016 | 2014 | 2005 | 0 | |
| 4 | | 64 | FALSE | 2016 | | 2006 | 0 | |
| 4 | No Treatm | 63 | FALSE | 2016 | 2015 | 2011 | 0 | |
| 1 | | 80 | FALSE | 2016 | 2013 | 2012 | 0 | |
| 1 | | 61 | FALSE | 2016 | 2015 | 2013 | 0 | |
| 2 | | 66 | FALSE | 2016 | 2013 | 2012 | 0 | |
| 4 | | 55 | FALSE | 2016 | 2014 | | | |
| 4 | No Treatm | 57 | FALSE | 2016 | 2015 | 2011 | 0 | 178 |
| 2 | No Treatm | 70 | FALSE | 2016 | 2015 | 2009 | 0 | |
| 1 | | 49 | FALSE | 2016 | 2015 | 2013 | 0 | |
| 3 | No Treatm | 100 | FALSE | 2016 | 2015 | 2013 | 0 | |
| 1 | | 62 | FALSE | 2016 | 2014 | 2013 | 0 | |
| 2 | No Treatm | 62 | FALSE | 2016 | 2015 | 2011 | 0 | |
| 4 | No Treatm | 61 | FALSE | 2016 | 2015 | 2011 | 0 | 178 |
| 4 | No Treatm | 69 | FALSE | 2016 | 2014 | 2013 | 0 | |
| 1 | | 65 | FALSE | 2016 | 2014 | 2008 | 0 | |
| 3 | No Treatm | 61 | FALSE | 2016 | 2013 | 2012 | 0 | |
| 3 | | 72 | FALSE | 2016 | 2014 | 2013 | 0 | |
| | | 63 | FALSE | 2016 | 2015 | 2013 | 0 | 178 |
| 2 | No Treatm | 59 | FALSE | 2016 | 2014 | 2013 | 0 | |

| disease_c | pathology | program | project_c | vial_numb | age_at_ir | days_to_b | days_to_c | ethnicity |
|---|---|---|---|---|---|---|---|---|
| ESCA | TCGA-Z6-AAPN.75067 | TCGA | | A | 57 | -20926 | | not hispa |
| ESCA | TCGA-L5-A40M.675BD | TCGA | | A | 54 | -19845 | 1458 | not repor |
| ESCA | TCGA-IG-A802.54F24 | TCGA | | A | 62 | -22869 | 142 | not hispa |
| ESCA | TCGA-LN-A5U5.F791C | TCGA | | A | 57 | -20993 | 136 | not hispa |
| ESCA | TCGA-IG-ATCGA | TCGA | | A | 72 | -26574 | 24 | not hispa |
| ESCA | TCGA-LN-A9FR.17F05 | TCGA | | A | 70 | -25657 | | not hispa |
| ESCA | TCGA-IG-A3I8.713A7 | TCGA | | A | 51 | -18786 | | not hispa |
| ESCA | TCGA-LN-A49X.77C11 | TCGA | | A | 44 | -16154 | | not hispa |
| ESCA | TCGA-VR-A8EW.0E37F | TCGA | | A | 57 | -20902 | 247 | not repor |
| ESCA | TCGA-LN-A7HW.EDF0A | TCGA | | A | 59 | -21840 | | not hispa |
| ESCA | TCGA-L5-A8NK.2249D | TCGA | | A | 84 | -30719 | | not repor |
| ESCA | TCGA-JY-A6FE.B3CA2 | TCGA | | A | 49 | -18105 | 112 | not repor |
| ESCA | TCGA-VR-A8E0.1063C | TCGA | | A | 49 | -18123 | | not repor |
| ESCA | TCGA-Z6-A9VB.C5F85 | TCGA | | A | 53 | -19470 | | not hispa |
| ESCA | TCGA-L5-A8NQ.26722 | TCGA | | A | 71 | -26250 | 650 | not repor |
| ESCA | TCGA-VR-A8Q7.86C5C | TCGA | | A | 60 | -22149 | | not repor |
| ESCA | TCGA-LN-A9F0.5FBD6 | TCGA | | A | 42 | -15551 | | not hispa |
| ESCA | TCGA-LN-A4MQ.C4286 | TCGA | | A | 46 | -16990 | | not hispa |
| ESCA | TCGA-IG-A3QL.DF339 | TCGA | | A | 54 | -19755 | | not hispa |
| ESCA | TCGA-LN-ATCGA | TCGA | | A | 62 | -22717 | | not hispa |
| ESCA | TCGA-VR-A8EY.6A2DC | TCGA | | A | 44 | -16255 | | not repor |
| ESCA | TCGA-LN-A7HY.CF4E8 | TCGA | | A | 50 | -18296 | | not hispa |
| ESCA | TCGA-JY-A6FG.C6926 | TCGA | | A | 50 | -18348 | 1263 | not repor |
| ESCA | TCGA-VR-A8ER.E984A | TCGA | | A | 54 | -19951 | 378 | not repor |
| ESCA | TCGA-VR-A8EU.37CB2 | TCGA | | A | 51 | -18711 | 557 | not repor |
| ESCA | TCGA-IG-ATCGA | TCGA | | A | 48 | -17860 | 567 | not hispa |
| ESCA | TCGA-VR-A8ET.BD6C2 | TCGA | | A | 64 | -23517 | 47 | not repor |
| ESCA | TCGA-LN-A49U.0FAC8 | TCGA | | A | 62 | -22969 | | not hispa |
| ESCA | TCGA-IG-ATCGA | TCGA | | A | 63 | -23259 | | not hispa |
| ESCA | TCGA-L5-A88W.8BF17 | TCGA | | A | 67 | -24716 | 764 | not repor |
| ESCA | TCGA-LN-ATCGA | TCGA | | A | 49 | -18160 | 681 | not hispa |
| ESCA | TCGA-LN-A8I0.9FBEE | TCGA | | A | 52 | -19328 | | not hispa |
| ESCA | TCGA-LN-A4A1.DCFBF | TCGA | | A | 60 | -21954 | | not hispa |
| ESCA | TCGA-IG-A97H.2C604 | TCGA | | A | 36 | -13166 | | not hispa |
| ESCA | TCGA-Z6-A8JD.E402E | TCGA | | A | 53 | -19523 | | not hispa |
| ESCA | TCGA-VR-AA7I.D93BC | TCGA | | A | 70 | -25855 | 484 | not repor |
| ESCA | TCGA-IG-ATCGA | TCGA | | A | 60 | -22070 | 390 | not hispa |
| ESCA | TCGA-JY-A93F.666D9 | TCGA | | A | 58 | -21257 | | not repor |
| ESCA | TCGA-LN-A7HZ.09349 | TCGA | | A | 49 | -17931 | | not hispa |
| ESCA | TCGA-LN-A49P.0EA71 | TCGA | | A | 71 | -26189 | | not hispa |
| ESCA | TCGA-S8-A6BW.820EE | TCGA | | A | 51 | -18944 | | not hispa |
| ESCA | TCGA-IG-A5S3.FDFE8 | TCGA | | A | 69 | -25414 | | not hispa |
| ESCA | TCGA-L5-A88S.DA04E | TCGA | | A | 84 | -30954 | | not repor |
| ESCA | TCGA-VR-A8EZ.5CD32 | TCGA | | A | 47 | -17449 | 553 | not repor |
| ESCA | TCGA-LN-A4A4.4EADC | TCGA | | A | 36 | -13419 | | not hispa |
| ESCA | TCGA-VR-A8EX.8773A | TCGA | | A | 63 | -23226 | 855 | not repor |
| ESCA | TCGA-LN-A4A9.CBB2F | TCGA | | A | 58 | -21217 | 351 | not hispa |
| ESCA | TCGA-LN-A49M.DA5E3 | TCGA | | A | 62 | -22933 | | not hispa |
| ESCA | TCGA-LN-A4A3.0EA87 | TCGA | | A | 61 | -22313 | | not hispa |
| ESCA | TCGA-LN-A49Y.32800 | TCGA | | A | 77 | -28125 | | not hispa |

| | | | | | | | |
|---|---|---|---|---|---|---|---|
| ESCA | TCGA-Z6-A8JE.EA4E0 | TCGA | A | 57 | -20855 | | not hispa |
| ESCA | TCGA-IG-A | TCGA | A | 53 | -19567 | | not hispa |
| ESCA | TCGA-LN-A4A8.60FF0 | TCGA | A | 52 | -19046 | | not hispa |
| ESCA | TCGA-LN-A | TCGA | A | 72 | -26517 | | not hispa |
| ESCA | TCGA-LN-A | TCGA | A | 46 | -16928 | | not hispa |
| ESCA | TCGA-LN-A5U6.E52D8 | TCGA | A | 54 | -19840 | | not hispa |
| ESCA | TCGA-L5-A43J.3C212 | TCGA | A | 90 | -32872 | 0 | not repor |
| ESCA | TCGA-LN-A7HV.CF1C8 | TCGA | A | 58 | -21462 | | not hispa |
| ESCA | TCGA-IG-A6QS.02D2F | TCGA | A | 54 | -19900 | 303 | not hispa |
| ESCA | TCGA-L5-A88Z.9B690 | TCGA | A | 70 | -25890 | | not repor |
| ESCA | TCGA-JY-A6FA.B1CD2 | TCGA | A | 51 | -18978 | 1361 | not repor |
| ESCA | TCGA-LN-A49S.CD1FE | TCGA | A | 59 | -21865 | | not hispa |
| ESCA | TCGA-IG-A3YB.3886C | TCGA | A | 61 | -22316 | | not hispa |
| ESCA | TCGA-LN-A8I1.01727 | TCGA | A | 67 | -24551 | | not hispa |
| ESCA | TCGA-IG-A3YC.D21EC | TCGA | A | 62 | -22774 | | not hispa |
| ESCA | TCGA-V5-AASV.3E53E | TCGA | A | 67 | -24559 | | not hispa |
| ESCA | TCGA-LN-A | TCGA | A | 47 | -17237 | | not hispa |
| ESCA | TCGA-XP-A8T6.3C4F0 | TCGA | A | 54 | -19886 | 763 | hispanic |
| ESCA | TCGA-LN-A9FP.E4974 | TCGA | A | 60 | -22104 | | not hispa |
| ESCA | TCGA-IC-A6RF.8E288 | TCGA | A | 69 | -25417 | | not hispa |
| ESCA | TCGA-IG-A97I.2AA80 | TCGA | A | 58 | -21271 | | not hispa |
| ESCA | TCGA-XP-A8T8.3CA9E | TCGA | A | 49 | -18048 | | hispanic |
| ESCA | TCGA-LN-A | TCGA | A | 73 | -26910 | | not repor |
| ESCA | TCGA-VR-AA4G.AB01C | TCGA | A | 51 | -18909 | | not repor |
| ESCA | TCGA-JY-A6FD.764C0 | TCGA | A | 51 | -18808 | | not repor |
| ESCA | TCGA-IG-A50L.FFA1C | TCGA | A | 58 | -21214 | | not hispa |
| ESCA | TCGA-KH-A6WC.2316F | TCGA | A | 82 | -30167 | | not hispa |
| ESCA | TCGA-LN-A | TCGA | A | 56 | -20510 | | not hispa |
| ESCA | TCGA-VR-A8EP.3732C | TCGA | A | 51 | -18942 | | not repor |

| gender.de | race.demc | vital_sta | year_of_b | year_of_c | age_at_di | classific | days_to_c | days_to_l |
|---|---|---|---|---|---|---|---|---|
| male | white | Alive | 1956 | | 20926 | not repor | 0 | 81 |
| female | white | Dead | 1956 | | 19845 | not repor | 0 | 124 |
| male | asian | Dead | 1951 | | 22869 | not repor | 0 | 14 |
| male | asian | Dead | 1955 | | 20993 | not repor | 0 | 5 |
| male | white | Dead | 1940 | 2012 | 26574 | not repor | 0 | |
| male | asian | Alive | 1943 | | 25657 | not repor | 0 | 373 |
| female | white | Alive | 1960 | | 18786 | not repor | 0 | 1012 |
| male | asian | Alive | 1967 | | 16154 | not repor | 0 | 384 |
| male | asian | Dead | 1955 | 2012 | 20902 | not repor | 0 | |
| male | asian | Alive | 1954 | | 21840 | not repor | 0 | 365 |
| female | white | Alive | 1920 | | 30719 | not repor | 0 | 412 |
| male | white | Dead | 1960 | 2009 | 18105 | not repor | 0 | |
| male | white | Alive | 1964 | | 18123 | not repor | 0 | 785 |
| male | white | Alive | 1960 | | 19470 | not repor | 0 | 40 |
| male | white | Dead | 1930 | 2002 | 26250 | not repor | 0 | |
| male | white | Alive | 1950 | | 22149 | not repor | 0 | 1590 |
| male | asian | Alive | 1971 | | 15551 | not repor | 0 | 4 |
| male | asian | Alive | 1964 | | 16990 | not repor | 0 | 375 |
| male | white | Alive | 1957 | | 19755 | not repor | 0 | 1071 |
| male | asian | Alive | 1951 | | 22717 | not repor | 0 | 391 |
| female | white | Alive | 1968 | | 16255 | not repor | 0 | 1025 |
| male | asian | Alive | 1963 | | 18296 | not repor | 0 | 366 |
| male | white | Dead | 1959 | 2012 | 18348 | not repor | 0 | |
| male | not repor | Dead | 1955 | 2010 | 19951 | not repor | 0 | |
| male | white | Dead | 1960 | 2012 | 18711 | not repor | 0 | |
| male | white | Dead | 1964 | | 17860 | not repor | 0 | 1 |
| male | white | Dead | 1946 | 2010 | 23517 | not repor | 0 | |
| male | asian | Alive | 1949 | | 22969 | not repor | 0 | 467 |
| male | white | Alive | 1949 | | 23259 | not repor | 0 | 518 |
| male | white | Dead | 1940 | 2009 | 24716 | not repor | 0 | |
| male | asian | Dead | 1962 | | 18160 | not repor | 0 | 3 |
| male | asian | Alive | 1961 | | 19328 | not repor | 0 | 407 |
| male | asian | Alive | 1951 | | 21954 | not repor | 0 | 383 |
| male | asian | Alive | 1977 | | 13166 | not repor | 0 | 441 |
| male | white | Alive | 1960 | | 19523 | not repor | 0 | 104 |
| male | white | Dead | 1940 | 2011 | 25855 | not repor | 0 | |
| male | asian | Dead | 1952 | | 22070 | not repor | 0 | 11 |
| female | white | Alive | 1954 | | 21257 | not repor | 0 | 731 |
| male | asian | Alive | 1964 | | 17931 | not repor | 0 | 401 |
| male | asian | Alive | 1940 | | 26189 | not repor | 0 | 375 |
| male | white | Alive | 1961 | | 18944 | not repor | 0 | 620 |
| female | white | Alive | 1943 | | 25414 | not repor | 0 | 712 |
| male | white | Alive | 1929 | | 30954 | not repor | 0 | 471 |
| male | white | Dead | 1966 | 2014 | 17449 | not repor | 0 | |
| male | asian | Alive | 1975 | | 13419 | not repor | 0 | 383 |
| male | not repor | Dead | 1946 | 2011 | 23226 | not repor | 0 | |
| male | asian | Dead | 1954 | | 21217 | not repor | 0 | 2 |
| male | asian | Alive | 1948 | | 22933 | not repor | 0 | 385 |
| male | asian | Alive | 1950 | | 22313 | not repor | 0 | 388 |
| male | asian | Alive | 1934 | | 28125 | not repor | 0 | 379 |

| | | | | | | | | |
|---|---|---|---|---|---|---|---|---|
| male | white | Alive | 1956 | | 20855 | not repor | 0 | 64 |
| male | white | Alive | 1959 | | 19567 | not repor | 0 | 632 |
| male | asian | Alive | 1960 | | 19046 | not repor | 0 | 472 |
| male | asian | Alive | 1941 | | 26517 | not repor | 0 | 372 |
| male | asian | Alive | 1966 | | 16928 | not repor | 0 | 768 |
| male | asian | Alive | 1958 | | 19840 | not repor | 0 | 375 |
| male | white | Dead | 1921 | 2011 | 32872 | not repor | 0 | |
| male | asian | Alive | 1954 | | 21462 | not repor | 0 | 320 |
| male | white | Dead | 1959 | | 19900 | not repor | 0 | 4 |
| female | white | Alive | 1935 | | 25890 | not repor | 0 | 225 |
| male | asian | Dead | 1955 | 2009 | 18978 | not repor | 0 | |
| male | asian | Alive | 1952 | | 21865 | not repor | 0 | 400 |
| male | white | Alive | 1951 | | 22316 | not repor | 0 | 80 |
| female | asian | Alive | 1946 | | 24551 | not repor | 0 | 401 |
| male | white | Alive | 1950 | | 22774 | not repor | 0 | 612 |
| male | black or | Alive | | | 24559 | not repor | 0 | 467 |
| male | asian | Alive | 1964 | | 17237 | not repor | 0 | 408 |
| male | black or | Dead | 1955 | 2011 | 19886 | not repor | 0 | |
| female | asian | Alive | 1953 | | 22104 | not repor | 0 | 366 |
| female | white | Alive | 1944 | | 25417 | not repor | 0 | 477 |
| male | white | Alive | 1955 | | 21271 | not repor | 0 | 370 |
| male | black or | Alive | 1962 | | 18048 | not repor | 0 | 437 |
| male | asian | Alive | 1938 | | 26910 | not repor | 0 | 403 |
| female | white | Alive | 1962 | | 18909 | not repor | 0 | 549 |
| female | asian | Alive | 1957 | | 18808 | not repor | 0 | 2069 |
| male | asian | Alive | 1954 | | 21214 | not repor | 0 | 16 |
| male | white | Alive | 1931 | | 30167 | not repor | 0 | 191 |
| male | asian | Alive | 1957 | | 20510 | not repor | 0 | 375 |
| male | white | Alive | 1962 | | 18942 | not repor | 0 | 824 |

| icd_10_cc | last_know | morpholog | primary_d | prior_mal | prior_tre | progressi | site_of_r | synchronc |
|---|---|---|---|---|---|---|---|---|
| C15.5 | not repor | Mar-70 | Squamous | no | No | not repor | Lower thi | No |
| C15.5 | not repor | Mar-83 | Basaloid | no | No | not repor | Lower thi | No |
| C15.5 | not repor | Mar-70 | Squamous | no | No | not repor | Lower thi | No |
| C15.5 | not repor | Mar-70 | Squamous | no | No | not repor | Lower thi | No |
| C15.9 | not repor | Mar-71 | Squamous | no | No | not repor | Esophagus | No |
| C15.3 | not repor | Mar-70 | Squamous | no | No | not repor | Upper thi | No |
| C15.9 | not repor | Mar-71 | Squamous | no | No | not repor | Esophagus | No |
| C15.4 | not repor | Mar-70 | Squamous | no | No | not repor | Middle th | No |
| C15.4 | not repor | Mar-70 | Squamous | no | No | not repor | Middle th | No |
| C15.4 | not repor | Mar-70 | Squamous | no | No | not repor | Middle th | No |
| C15.4 | not repor | Mar-70 | Squamous | no | No | not repor | Middle th | No |
| C15.5 | not repor | Mar-70 | Squamous | no | No | not repor | Lower thi | No |
| C15.5 | not repor | Mar-70 | Squamous | no | No | not repor | Lower thi | No |
| C15.5 | not repor | Mar-70 | Squamous | no | No | not repor | Lower thi | No |
| C15.4 | not repor | Mar-70 | Squamous | no | No | not repor | Middle th | No |
| C15.5 | not repor | Mar-70 | Squamous | no | No | not repor | Lower thi | No |
| C15.5 | not repor | Mar-70 | Squamous | no | No | not repor | Lower thi | No |
| C15.5 | not repor | Mar-70 | Squamous | no | No | not repor | Lower thi | No |
| C15.5 | not repor | Mar-71 | Squamous | no | No | not repor | Lower thi | No |
| C15.4 | not repor | Mar-70 | Squamous | no | No | not repor | Middle th | No |
| C15.5 | not repor | Mar-70 | Squamous | no | No | not repor | Lower thi | No |
| C15.3 | not repor | Mar-70 | Squamous | no | No | not repor | Upper thi | No |
| C16.0 | not repor | Mar-70 | Squamous | no | No | not repor | Cardia, N | No |
| C15.5 | not repor | Mar-70 | Squamous | no | No | not repor | Lower thi | No |
| C15.4 | not repor | Mar-70 | Squamous | no | No | not repor | Middle th | No |
| C15.4 | not repor | Mar-70 | Squamous | no | No | not repor | Middle th | No |
| C15.5 | not repor | Mar-70 | Squamous | no | No | not repor | Lower thi | No |
| C15.4 | not repor | Mar-70 | Squamous | no | No | not repor | Middle th | No |
| C15.3 | not repor | Mar-70 | Squamous | no | No | not repor | Upper thi | No |
| C15.5 | not repor | Mar-70 | Squamous | no | No | not repor | Lower thi | No |
| C15.4 | not repor | Mar-70 | Squamous | no | No | not repor | Middle th | No |
| C15.5 | not repor | Mar-70 | Squamous | no | No | not repor | Lower thi | No |
| C15.4 | not repor | Mar-70 | Squamous | no | No | not repor | Middle th | No |
| C15.5 | not repor | Mar-70 | Squamous | no | No | not repor | Lower thi | No |
| C15.9 | not repor | Mar-70 | Squamous | no | No | not repor | Esophagus | No |
| C15.4 | not repor | Mar-70 | Squamous | no | No | not repor | Middle th | No |
| C15.5 | not repor | Mar-70 | Squamous | no | No | not repor | Lower thi | No |
| C15.5 | not repor | Mar-70 | Squamous | no | No | not repor | Lower thi | No |
| C15.3 | not repor | Mar-70 | Squamous | no | No | not repor | Upper thi | No |
| C15.5 | not repor | Mar-70 | Squamous | no | No | not repor | Lower thi | No |
| C15.9 | not repor | Mar-70 | Squamous | no | No | not repor | Esophagus | No |
| C15.9 | not repor | Mar-70 | Squamous | no | No | not repor | Esophagus | No |
| C15.5 | not repor | Mar-70 | Squamous | no | No | not repor | Lower thi | No |
| C15.5 | not repor | Mar-70 | Squamous | no | No | not repor | Lower thi | No |
| C15.5 | not repor | Mar-70 | Squamous | no | No | not repor | Lower thi | No |
| C15.4 | not repor | Mar-70 | Squamous | no | No | not repor | Middle th | No |
| C15.4 | not repor | Mar-70 | Squamous | no | No | not repor | Middle th | No |
| C15.5 | not repor | Mar-70 | Squamous | no | No | not repor | Lower thi | No |
| C15.4 | not repor | Mar-70 | Squamous | no | No | not repor | Middle th | No |
| C15.4 | not repor | Mar-70 | Squamous | no | No | not repor | Middle th | No |

| | | | | | | | | |
|---|---|---|---|---|---|---|---|---|
| C15.3 | not repor | Mar-70 | Squamous | no | No | not repor | Upper thi | No |
| C15.5 | not repor | Mar-70 | Squamous | no | No | not repor | Lower thi | No |
| C15.4 | not repor | Mar-70 | Squamous | no | No | not repor | Middle th | No |
| C15.4 | not repor | Mar-70 | Squamous | no | No | not repor | Middle th | No |
| C15.4 | not repor | Mar-70 | Squamous | no | No | not repor | Middle th | No |
| C15.5 | not repor | Mar-70 | Squamous | no | No | not repor | Lower thi | No |
| C15.4 | not repor | Mar-70 | Squamous | yes | No | not repor | Middle th | Not Repor |
| C15.4 | not repor | Mar-70 | Squamous | no | No | not repor | Middle th | No |
| C15.5 | not repor | Mar-71 | Squamous | no | No | not repor | Lower thi | No |
| C15.4 | not repor | Mar-70 | Squamous | no | No | not repor | Middle th | No |
| C15.5 | not repor | Mar-70 | Squamous | no | No | not repor | Lower thi | No |
| C15.5 | not repor | Mar-70 | Squamous | no | No | not repor | Lower thi | No |
| C15.5 | not repor | Mar-70 | Squamous | no | No | not repor | Lower thi | No |
| C15.9 | not repor | Mar-70 | Squamous | no | No | not repor | Esophagus | No |
| C15.5 | not repor | Mar-70 | Squamous | no | No | not repor | Lower thi | No |
| C15.9 | not repor | Mar-70 | Squamous | no | No | not repor | Esophagus | No |
| C15.4 | not repor | Mar-70 | Squamous | no | No | not repor | Middle th | No |
| C15.5 | not repor | Mar-70 | Squamous | no | No | not repor | Lower thi | No |
| C15.4 | not repor | Mar-70 | Squamous | no | No | not repor | Middle th | No |
| C15.5 | not repor | Mar-70 | Squamous | yes | No | not repor | Middle th | Not Repor |
| C15.5 | not repor | Mar-70 | Squamous | no | No | not repor | Lower thi | No |
| C15.5 | not repor | Mar-70 | Squamous | no | No | not repor | Middle th | No |
| C15.4 | not repor | Mar-70 | Squamous | no | No | not repor | Middle th | No |
| C15.4 | not repor | Mar-70 | Squamous | no | No | not repor | Middle th | No |
| C15.5 | not repor | Mar-70 | Squamous | no | No | not repor | Lower thi | No |
| C15.5 | not repor | Mar-70 | Squamous | no | No | not repor | Lower thi | No |
| C15.4 | not repor | Mar-70 | Squamous | no | No | not repor | Middle th | No |
| C15.4 | not repor | Mar-70 | Squamous | no | No | not repor | Middle th | No |
| C15.4 | not repor | Mar-70 | Squamous | no | No | not repor | Middle th | No |

| tissue_or | tumor_gra | tumor_sta | year_of_d | disease_t | alcohol_h | bmi.expos | cigarette | height.ex |
|---|---|---|---|---|---|---|---|---|
| Lower thi | not repor | stage iia | 2013 | Squamous | Not Repor | 27.1809 | 0.054795 | 165 |
| Lower thi | not repor | stage ia | 2010 | Squamous | Yes | | 2.191781 | |
| Lower thi | not repor | stage iii | 2013 | Squamous | Yes | 19.23356 | 0.821918 | 166 |
| Lower thi | not repor | stage iv | 2012 | Squamous | Yes | 17.47472 | | 164 |
| Esophagus | not repor | stage ib | 2012 | Squamous | Yes | 22.14533 | 4.109589 | 170 |
| Upper thi | not repor | stage iib | 2013 | Squamous | Yes | 20.61925 | | 172 |
| Esophagus | not repor | stage iia | 2011 | Squamous | No | 17.63085 | | 165 |
| Middle th | not repor | stage iia | 2011 | Squamous | No | | | |
| Middle th | not repor | stage iii | 2012 | Squamous | Yes | 17.84652 | 0.547945 | 164 |
| Middle th | not repor | stage iia | 2013 | Squamous | Yes | 22.27531 | 2.191781 | 176 |
| Middle th | not repor | stage iia | 2004 | Squamous | Yes | 25.84777 | 1.369863 | 161 |
| Lower thi | not repor | stage iii | 2009 | Squamous | Yes | 20.0155 | 1.589041 | 176 |
| Lower thi | not repor | stage iia | 2013 | Squamous | Yes | 19.66231 | | 179 |
| Lower thi | not repor | stage iii | 2013 | Squamous | Not Repor | 24.52435 | | 159 |
| Middle th | not repor | stage iia | 2001 | Squamous | Yes | 27.45865 | 3.287671 | 178 |
| Lower thi | not repor | stage iii | 2010 | Squamous | Yes | 19.72318 | 0.739726 | 170 |
| Lower thi | not repor | stage iia | 2013 | Squamous | No | 22.49135 | | 170 |
| Lower thi | not repor | stage iii | 2010 | Squamous | No | 19.84127 | | 168 |
| Lower thi | not repor | stage iia | 2011 | Squamous | Yes | 25.25952 | 1.643836 | 170 |
| Middle th | not repor | stage iia | 2013 | Squamous | Yes | 21.29529 | | 172 |
| Lower thi | not repor | stage iia | 2012 | Squamous | No | 25.71166 | | 165 |
| Upper thi | not repor | stage iii | 2013 | Squamous | Yes | 20.98399 | | 176 |
| Cardia, N | not repor | stage iii | 2009 | Squamous | Yes | 36.75365 | 0.328767 | 173 |
| Lower thi | not repor | stage iii | 2009 | Squamous | Yes | 17.10291 | 0.671233 | 164 |
| Middle th | not repor | stage iv | 2011 | Squamous | Yes | 16.84503 | 0.547945 | 174 |
| Middle th | not repor | stage iib | 2012 | Squamous | Yes | 25.96305 | 1.09589 | 182 |
| Lower thi | not repor | stage iia | 2010 | Squamous | Yes | 25.78125 | | 160 |
| Middle th | not repor | stage iia | 2011 | Squamous | Yes | 20.19947 | 2.191781 | 178 |
| Upper thi | not repor | stage iib | 2012 | Squamous | No | 19.88385 | | 178 |
| Lower thi | not repor | stage iia | 2007 | Squamous | Yes | 24.15167 | | 182 |
| Middle th | not repor | stage iia | 2011 | Squamous | Yes | 22.20408 | | 175 |
| Lower thi | not repor | stage iia | 2013 | Squamous | Yes | 22.22906 | 0.821918 | 171 |
| Middle th | not repor | stage iia | 2011 | Squamous | Yes | 21.97134 | | 172 |
| Lower thi | not repor | stage iia | 2013 | Squamous | Yes | 20.41522 | 0.547945 | 170 |
| Esophagus | not repor | stage iib | 2013 | Squamous | No | 23.32342 | | 172 |
| Middle th | not repor | stage iii | 2010 | Squamous | Yes | 19.15119 | 1.09589 | 171 |
| Lower thi | not repor | stage iii | 2012 | Squamous | Yes | 17.71542 | | 168 |
| Lower thi | not repor | stage ib | 2012 | Squamous | Yes | 26.81359 | | 165 |
| Upper thi | not repor | stage iia | 2013 | Squamous | Yes | 20.0155 | 1.369863 | 176 |
| Lower thi | not repor | stage iia | 2011 | Squamous | Yes | 20.2449 | 2.191781 | 175 |
| Esophagus | not repor | stage ib | 2012 | Squamous | Yes | 24.25867 | | 177 |
| Esophagus | not repor | stage iib | 2012 | Squamous | Yes | 21.53491 | | 179 |
| Lower thi | not repor | stage ib | 2013 | Squamous | No | 22.99262 | 1.643836 | 183 |
| Lower thi | not repor | stage iii | 2013 | Squamous | No | 24.74094 | 2.465753 | 167 |
| Lower thi | not repor | stage iii | 2011 | Squamous | Yes | 20.2449 | | 175 |
| Middle th | not repor | stage iva | 2009 | Squamous | No | 20.51913 | 0.547945 | 171 |
| Middle th | not repor | stage iia | 2012 | Squamous | Yes | 22.7244 | 1.09589 | 178 |
| Lower thi | not repor | stage iia | 2010 | Squamous | No | 20.19558 | | 168 |
| Middle th | not repor | stage iii | 2011 | Squamous | No | 21.29529 | 1.917808 | 172 |
| Middle th | not repor | stage iia | 2011 | Squamous | Yes | 20.19558 | | 168 |

| | | | | | | | | |
|---|---|---|---|---|---|---|---|---|
| Upper thi | not repor | stage iii | 2013 | Squamous | No | 21.61281 | | 168 |
| Lower thi | not repor | stage iii | 2012 | Squamous | Yes | 23.71961 | 2.054795 | 179 |
| Middle th | not repor | stage iia | 2012 | Squamous | Yes | 22.20408 | | 175 |
| Middle th | not repor | stage iia | 2013 | Squamous | Yes | 22.64738 | 2.739726 | 172 |
| Middle th | not repor | stage iia | 2012 | Squamous | Yes | 18.55677 | 1.260274 | 169 |
| Lower thi | not repor | stage iib | 2012 | Squamous | Yes | 20.28123 | 1.315068 | 172 |
| Middle th | not repor | stage iib | 2011 | Squamous | Yes | 21.3578 | 2.958904 | 169 |
| Middle th | not repor | stage iia | 2012 | Squamous | Yes | 23.78121 | 0.821918 | 174 |
| Lower thi | not repor | stage iib | 2013 | Squamous | Yes | 22.59814 | 1.863014 | 176 |
| Middle th | not repor | stage iia | 2005 | Squamous | Yes | 24.84098 | | 163 |
| Lower thi | not repor | stage iib | 2006 | Squamous | Yes | 21.13886 | 1.917808 | 174 |
| Lower thi | not repor | stage iia | 2011 | Squamous | No | 21.04982 | 2.191781 | 173 |
| Lower thi | not repor | stage iii | 2012 | Squamous | No | 25.24934 | | 178 |
| Esophagus | not repor | stage iia | 2013 | Squamous | Yes | 26.40235 | | 152 |
| Lower thi | not repor | stage iii | 2012 | Squamous | Yes | 23.10843 | 2.191781 | 169 |
| Esophagus | not repor | stage iib | | Squamous | Yes | 20.20202 | 1.643836 | 165 |
| Middle th | not repor | stage iia | 2011 | Squamous | Yes | 22.83288 | 1.09589 | 158 |
| Lower thi | not repor | stage iib | 2009 | Squamous | Yes | | 1.369863 | |
| Middle th | not repor | stage iia | 2013 | Squamous | Yes | 21.49029 | | 151 |
| Middle th | not repor | stage ia | 2013 | Squamous | Yes | 34.60208 | 1.205479 | 170 |
| Lower thi | not repor | stage iia | 2013 | Squamous | No | 20.47827 | | 174 |
| Middle th | not repor | stage iib | 2011 | Squamous | Yes | | 1.041096 | |
| Middle th | not repor | stage iii | 2011 | Squamous | Yes | 23.24341 | 2.191781 | 162 |
| Middle th | not repor | stage iii | 2013 | Squamous | No | 27.29322 | 0.547945 | 159 |
| Lower thi | not repor | stage iia | 2008 | Squamous | Yes | 28.13366 | | 152 |
| Lower thi | not repor | stage iii | 2012 | Squamous | No | 23.24341 | 1.69863 | 162 |
| Middle th | not repor | stage ia | 2013 | Squamous | Yes | 26.44628 | 2.739726 | 165 |
| Middle th | not repor | stage iia | 2013 | Squamous | Yes | 20.57143 | 1.643836 | 175 |
| Middle th | not repor | stage iii | 2013 | Squamous | No | 19.71332 | 2.958904 | 173 |

| pack_year | weight.exp | primary_s | name.proj | project_i | releasabl | bcr_id.ti | code.tiss | name.tiss |
|---|---|---|---|---|---|---|---|---|
| 1 | 74 | Esophagus | Esophagea | TCGA-ESCA | FALSE | NCH | Z6 | Cureline |
| 40 | 69 | Esophagus | Esophagea | TCGA-ESCA | FALSE | NCH | L5 | Universit |
| 15 | 53 | Esophagus | Esophagea | TCGA-ESCA | FALSE | NCH | IG | Asterand |
| | 47 | Esophagus | Esophagea | TCGA-ESCA | FALSE | NCH | LN | ILSBIO |
| 75 | 64 | Esophagus | Esophagea | TCGA-ESCA | FALSE | NCH | IG | Asterand |
| | 61 | Esophagus | Esophagea | TCGA-ESCA | FALSE | NCH | LN | ILSBIO |
| | 48 | Esophagus | Esophagea | TCGA-ESCA | FALSE | NCH | IG | Asterand |
| | | Esophagus | Esophagea | TCGA-ESCA | FALSE | NCH | LN | ILSBIO |
| 10 | 48 | Esophagus | Esophagea | TCGA-ESCA | FALSE | NCH | VR | Barretos |
| 40 | 69 | Esophagus | Esophagea | TCGA-ESCA | FALSE | NCH | LN | ILSBIO |
| 25 | 67 | Esophagus | Esophagea | TCGA-ESCA | FALSE | NCH | L5 | Universit |
| 29 | 62 | Esophagus | Esophagea | TCGA-ESCA | FALSE | NCH | JY | Universit |
| | 63 | Esophagus | Esophagea | TCGA-ESCA | FALSE | NCH | VR | Barretos |
| | 62 | Esophagus | Esophagea | TCGA-ESCA | FALSE | NCH | Z6 | Cureline |
| 60 | 87 | Esophagus | Esophagea | TCGA-ESCA | FALSE | NCH | L5 | Universit |
| 13.5 | 57 | Esophagus | Esophagea | TCGA-ESCA | FALSE | NCH | VR | Barretos |
| | 65 | Esophagus | Esophagea | TCGA-ESCA | FALSE | NCH | LN | ILSBIO |
| | 56 | Esophagus | Esophagea | TCGA-ESCA | FALSE | NCH | LN | ILSBIO |
| 30 | 73 | Esophagus | Esophagea | TCGA-ESCA | FALSE | NCH | IG | Asterand |
| | 63 | Esophagus | Esophagea | TCGA-ESCA | FALSE | NCH | LN | ILSBIO |
| | 70 | Esophagus | Esophagea | TCGA-ESCA | FALSE | NCH | VR | Barretos |
| | 65 | Esophagus | Esophagea | TCGA-ESCA | FALSE | NCH | LN | ILSBIO |
| 6 | 110 | Stomach | Esophagea | TCGA-ESCA | FALSE | NCH | JY | Universit |
| 12.25 | 46 | Esophagus | Esophagea | TCGA-ESCA | FALSE | NCH | VR | Barretos |
| 10 | 51 | Esophagus | Esophagea | TCGA-ESCA | FALSE | NCH | VR | Barretos |
| 20 | 86 | Esophagus | Esophagea | TCGA-ESCA | FALSE | NCH | IG | Asterand |
| | 66 | Esophagus | Esophagea | TCGA-ESCA | FALSE | NCH | VR | Barretos |
| 40 | 64 | Esophagus | Esophagea | TCGA-ESCA | FALSE | NCH | LN | ILSBIO |
| | 63 | Esophagus | Esophagea | TCGA-ESCA | FALSE | NCH | IG | Asterand |
| | 80 | Esophagus | Esophagea | TCGA-ESCA | FALSE | NCH | L5 | Universit |
| | 68 | Esophagus | Esophagea | TCGA-ESCA | FALSE | NCH | LN | ILSBIO |
| 15 | 65 | Esophagus | Esophagea | TCGA-ESCA | FALSE | NCH | LN | ILSBIO |
| | 65 | Esophagus | Esophagea | TCGA-ESCA | FALSE | NCH | LN | ILSBIO |
| 10 | 59 | Esophagus | Esophagea | TCGA-ESCA | FALSE | NCH | IG | Asterand |
| | 69 | Esophagus | Esophagea | TCGA-ESCA | FALSE | NCH | Z6 | Cureline |
| 20 | 56 | Esophagus | Esophagea | TCGA-ESCA | FALSE | NCH | VR | Barretos |
| | 50 | Esophagus | Esophagea | TCGA-ESCA | FALSE | NCH | IG | Asterand |
| | 73 | Esophagus | Esophagea | TCGA-ESCA | FALSE | NCH | JY | Universit |
| 25 | 62 | Esophagus | Esophagea | TCGA-ESCA | FALSE | NCH | LN | ILSBIO |
| 40 | 62 | Esophagus | Esophagea | TCGA-ESCA | FALSE | NCH | LN | ILSBIO |
| | 76 | Esophagus | Esophagea | TCGA-ESCA | FALSE | NCH | S8 | ABS - IUF |
| | 69 | Esophagus | Esophagea | TCGA-ESCA | FALSE | NCH | IG | Asterand |
| 30 | 77 | Esophagus | Esophagea | TCGA-ESCA | FALSE | NCH | L5 | Universit |
| 45 | 69 | Esophagus | Esophagea | TCGA-ESCA | FALSE | NCH | VR | Barretos |
| | 62 | Esophagus | Esophagea | TCGA-ESCA | FALSE | NCH | LN | ILSBIO |
| 10 | 60 | Esophagus | Esophagea | TCGA-ESCA | FALSE | NCH | VR | Barretos |
| 20 | 72 | Esophagus | Esophagea | TCGA-ESCA | FALSE | NCH | LN | ILSBIO |
| | 57 | Esophagus | Esophagea | TCGA-ESCA | FALSE | NCH | LN | ILSBIO |
| 35 | 63 | Esophagus | Esophagea | TCGA-ESCA | FALSE | NCH | LN | ILSBIO |
| | 57 | Esophagus | Esophagea | TCGA-ESCA | FALSE | NCH | LN | ILSBIO |

| | | | | | | | | |
|---|---|---|---|---|---|---|---|---|
| | 61 | Esophagus | Esophagea | TCGA-ESCA | FALSE | NCH | Z6 | Cureline |
| 37.5 | 76 | Esophagus | Esophagea | TCGA-ESCA | FALSE | NCH | IG | Asterand |
| | 68 | Esophagus | Esophagea | TCGA-ESCA | FALSE | NCH | LN | ILSBIO |
| 50 | 67 | Esophagus | Esophagea | TCGA-ESCA | FALSE | NCH | LN | ILSBIO |
| 23 | 53 | Esophagus | Esophagea | TCGA-ESCA | FALSE | NCH | LN | ILSBIO |
| 24 | 60 | Esophagus | Esophagea | TCGA-ESCA | FALSE | NCH | LN | ILSBIO |
| 54 | 61 | Esophagus | Esophagea | TCGA-ESCA | FALSE | NCH | L5 | Universit |
| 15 | 72 | Esophagus | Esophagea | TCGA-ESCA | FALSE | NCH | LN | ILSBIO |
| 34 | 70 | Esophagus | Esophagea | TCGA-ESCA | FALSE | NCH | IG | Asterand |
| | 66 | Esophagus | Esophagea | TCGA-ESCA | FALSE | NCH | L5 | Universit |
| 35 | 64 | Esophagus | Esophagea | TCGA-ESCA | FALSE | NCH | JY | Universit |
| 40 | 63 | Esophagus | Esophagea | TCGA-ESCA | FALSE | NCH | LN | ILSBIO |
| | 80 | Esophagus | Esophagea | TCGA-ESCA | FALSE | NCH | IG | Asterand |
| | 61 | Esophagus | Esophagea | TCGA-ESCA | FALSE | NCH | LN | ILSBIO |
| 40 | 66 | Esophagus | Esophagea | TCGA-ESCA | FALSE | NCH | IG | Asterand |
| 30 | 55 | Esophagus | Esophagea | TCGA-ESCA | FALSE | NCH | V5 | Duke Univ |
| 20 | 57 | Esophagus | Esophagea | TCGA-ESCA | FALSE | NCH | LN | ILSBIO |
| 25 | 70 | Esophagus | Esophagea | TCGA-ESCA | FALSE | NCH | XP | Universit |
| | 49 | Esophagus | Esophagea | TCGA-ESCA | FALSE | NCH | LN | ILSBIO |
| 22 | 100 | Esophagus | Esophagea | TCGA-ESCA | FALSE | NCH | IC | Universit |
| | 62 | Esophagus | Esophagea | TCGA-ESCA | FALSE | NCH | IG | Asterand |
| 19 | 62 | Esophagus | Esophagea | TCGA-ESCA | FALSE | NCH | XP | Universit |
| 40 | 61 | Esophagus | Esophagea | TCGA-ESCA | FALSE | NCH | LN | ILSBIO |
| 10 | 69 | Esophagus | Esophagea | TCGA-ESCA | FALSE | NCH | VR | Barretos |
| | 65 | Esophagus | Esophagea | TCGA-ESCA | FALSE | NCH | JY | Universit |
| 31 | 61 | Esophagus | Esophagea | TCGA-ESCA | FALSE | NCH | IG | Asterand |
| 50 | 72 | Esophagus | Esophagea | TCGA-ESCA | FALSE | NCH | KH | Memorial |
| 30 | 63 | Esophagus | Esophagea | TCGA-ESCA | FALSE | NCH | LN | ILSBIO |
| 54 | 59 | Esophagus | Esophagea | TCGA-ESCA | FALSE | NCH | VR | Barretos |

| project.t | days_to_c | days_to_s | initial_w | is_ffpe.s | oct_embec | preservat | sample_ty | sample_ty |
|---|---|---|---|---|---|---|---|---|
| Esophagea | 181 | | 280 | FALSE | TRUE | | Primary T | 1 |
| Esophagea | 936 | | 80 | FALSE | FALSE | | Primary T | 1 |
| Esophagea | 91 | | 650 | FALSE | TRUE | | Primary T | 1 |
| Esophagea | 126 | | 320 | FALSE | TRUE | | Primary T | 1 |
| Esophagea | 86 | | 1100 | FALSE | TRUE | | Primary T | 1 |
| Esophagea | 97 | | 210 | FALSE | TRUE | | Primary T | 1 |
| Esophagea | 241 | | 140 | FALSE | TRUE | | Primary T | 1 |
| Esophagea | 480 | | 220 | FALSE | TRUE | | Primary T | 1 |
| Esophagea | 584 | | 70 | FALSE | FALSE | | Primary T | 1 |
| Esophagea | 232 | | 280 | FALSE | TRUE | | Primary T | 1 |
| Esophagea | 3539 | | 150 | FALSE | TRUE | | Primary T | 1 |
| Esophagea | 1479 | | 120 | FALSE | FALSE | | Primary T | 1 |
| Esophagea | 284 | | 160 | FALSE | FALSE | | Primary T | 1 |
| Esophagea | 126 | | 140 | FALSE | TRUE | | Primary T | 1 |
| Esophagea | 4627 | | 110 | FALSE | TRUE | | Primary T | 1 |
| Esophagea | 1205 | | 200 | FALSE | FALSE | | Primary T | 1 |
| Esophagea | 233 | | 290 | FALSE | TRUE | | Primary T | 1 |
| Esophagea | 647 | | 440 | FALSE | TRUE | | Primary T | 1 |
| Esophagea | 262 | | 150 | FALSE | TRUE | | Primary T | 1 |
| Esophagea | 115 | | 290 | FALSE | TRUE | | Primary T | 1 |
| Esophagea | 563 | | 80 | FALSE | FALSE | | Primary T | 1 |
| Esophagea | 162 | | 360 | FALSE | TRUE | | Primary T | 1 |
| Esophagea | 1316 | | 120 | FALSE | FALSE | | Primary T | 1 |
| Esophagea | 1475 | | 80 | FALSE | FALSE | | Primary T | 1 |
| Esophagea | 704 | | 30 | FALSE | FALSE | | Primary T | 1 |
| Esophagea | 198 | | 1200 | FALSE | TRUE | | Primary T | 1 |
| Esophagea | 1369 | | 80 | FALSE | FALSE | | Primary T | 1 |
| Esophagea | 513 | | 240 | FALSE | TRUE | | Primary T | 1 |
| Esophagea | 135 | | 570 | FALSE | TRUE | | Primary T | 1 |
| Esophagea | 2319 | | 140 | FALSE | FALSE | | Primary T | 1 |
| Esophagea | 270 | | 350 | FALSE | TRUE | | Primary T | 1 |
| Esophagea | 171 | | 240 | FALSE | TRUE | | Primary T | 1 |
| Esophagea | 464 | | 470 | FALSE | TRUE | | Primary T | 1 |
| Esophagea | 91 | | 1000 | FALSE | TRUE | | Primary T | 1 |
| Esophagea | 148 | | 140 | FALSE | TRUE | | Primary T | 1 |
| Esophagea | 1141 | | 80 | FALSE | TRUE | | Primary T | 1 |
| Esophagea | 102 | | 410 | FALSE | TRUE | | Primary T | 1 |
| Esophagea | 394 | | 120 | FALSE | FALSE | | Primary T | 1 |
| Esophagea | 161 | | 220 | FALSE | TRUE | | Primary T | 1 |
| Esophagea | 548 | | 390 | FALSE | TRUE | | Primary T | 1 |
| Esophagea | 222 | | 190 | FALSE | FALSE | | Primary T | 1 |
| Esophagea | 174 | | 230 | FALSE | TRUE | | Primary T | 1 |
| Esophagea | 155 | | 90 | FALSE | FALSE | | Primary T | 1 |
| Esophagea | 293 | | 40 | FALSE | FALSE | | Primary T | 1 |
| Esophagea | 291 | | 310 | FALSE | TRUE | | Primary T | 1 |
| Esophagea | 1480 | | 40 | FALSE | FALSE | | Primary T | 1 |
| Esophagea | 136 | | 170 | FALSE | TRUE | | Primary T | 1 |
| Esophagea | 634 | | 290 | FALSE | TRUE | | Primary T | 1 |
| Esophagea | 403 | | 190 | FALSE | TRUE | | Primary T | 1 |
| Esophagea | 471 | | 280 | FALSE | TRUE | | Primary T | 1 |

| | | | | | | |
|---|---|---|---|---|---|---|
| Esophagea | 108 | 150 | FALSE | TRUE | Primary T | 1 |
| Esophagea | 115 | 840 | FALSE | TRUE | Primary T | 1 |
| Esophagea | 143 | 320 | FALSE | TRUE | Primary T | 1 |
| Esophagea | 217 | 340 | FALSE | TRUE | Primary T | 1 |
| Esophagea | 141 | 510 | FALSE | TRUE | Primary T | 1 |
| Esophagea | 127 | 410 | FALSE | TRUE | Primary T | 1 |
| Esophagea | 391 | 210 | FALSE | FALSE | Primary T | 1 |
| Esophagea | 246 | 400 | FALSE | TRUE | Primary T | 1 |
| Esophagea | 173 | 770 | FALSE | TRUE | Primary T | 1 |
| Esophagea | 2968 | 70 | FALSE | FALSE | Primary T | 1 |
| Esophagea | 2444 | 80 | FALSE | FALSE | Primary T | 1 |
| Esophagea | 522 | 140 | FALSE | TRUE | Primary T | 1 |
| Esophagea | 100 | 400 | FALSE | TRUE | Primary T | 1 |
| Esophagea | 145 | 280 | FALSE | TRUE | Primary T | 1 |
| Esophagea | 93 | 780 | FALSE | TRUE | Primary T | 1 |
| Esophageal carcinoma | | 10 | FALSE | TRUE | Primary T | 1 |
| Esophagea | 605 | 330 | FALSE | TRUE | Primary T | 1 |
| Esophagea | 1546 | 180 | FALSE | TRUE | Primary T | 1 |
| Esophagea | 111 | 270 | FALSE | TRUE | Primary T | 1 |
| Esophagea | 113 | 170 | FALSE | TRUE | Primary T | 1 |
| Esophagea | 90 | 280 | FALSE | TRUE | Primary T | 1 |
| Esophagea | 930 | 70 | FALSE | TRUE | Primary T | 1 |
| Esophagea | 500 | 630 | FALSE | TRUE | Primary T | 1 |
| Esophagea | 190 | 90 | FALSE | FALSE | Primary T | 1 |
| Esophagea | 1805 | 80 | FALSE | FALSE | Primary T | 1 |
| Esophagea | 81 | 1210 | FALSE | TRUE | Primary T | 1 |
| Esophagea | 78 | 50 | FALSE | TRUE | Primary T | 1 |
| Esophagea | 200 | 330 | FALSE | TRUE | Primary T | 1 |
| Esophagea | 248 | 210 | FALSE | FALSE | Primary T | 1 |

| state.sam | tissue_type.samples |
| --- | --- |
| released | Not Reported |
| released | Not Reported |
| released | Not Reported |
| released | Not Reported |
| released | Not Reported |
| released | Not Reported |
| released | Not Reported |
| released | Not Reported |
| released | Not Reported |
| released | Not Reported |
| released | Not Reported |
| released | Not Reported |
| released | Not Reported |
| released | Not Reported |
| released | Not Reported |
| released | Not Reported |
| released | Not Reported |
| released | Not Reported |
| released | Not Reported |
| released | Not Reported |
| released | Not Reported |
| released | Not Reported |
| released | Not Reported |
| released | Not Reported |
| released | Not Reported |
| released | Not Reported |
| released | Not Reported |
| released | Not Reported |
| released | Not Reported |
| released | Not Reported |
| released | Not Reported |
| released | Not Reported |
| released | Not Reported |
| released | Not Reported |
| released | Not Reported |
| released | Not Reported |
| released | Not Reported |
| released | Not Reported |
| released | Not Reported |
| released | Not Reported |
| released | Not Reported |
| released | Not Reported |
| released | Not Reported |
| released | Not Reported |
| released | Not Reported |
| released | Not Reported |
| released | Not Reported |
| released | Not Reported |
| released | Not Reported |
| released | Not Reported |

released Not Reported
released Not Reported
released Not Reported
released Not Reported
released Not Reported
released Not Reported
released Not Reported
released Not Reported
released Not Reported
released Not Reported
released Not Reported
released Not Reported
released Not Reported
released Not Reported
released Not Reported
released Not Reported
released Not Reported
released Not Reported
released Not Reported
released Not Reported
released Not Reported
released Not Reported
released Not Reported
released Not Reported
released Not Reported
released Not Reported
released Not Reported
released Not Reported
released Not Reported
