# Supplementary material for: Gene Expression Profiling and Clinical Validation Reveal CEBPB for Early Diagnosis of Esophageal Squamous Cell Carcinoma
Source: Cancer Rep (Hoboken). 2026 Nov 8;9(11):e70753. doi: 10.1002/cnr2.70753 (PMC13745642; doi:10.1002/cnr2.70753)
Supplement: Supplementary file 6 [file CNR2-9-e70753-s010.pdf]

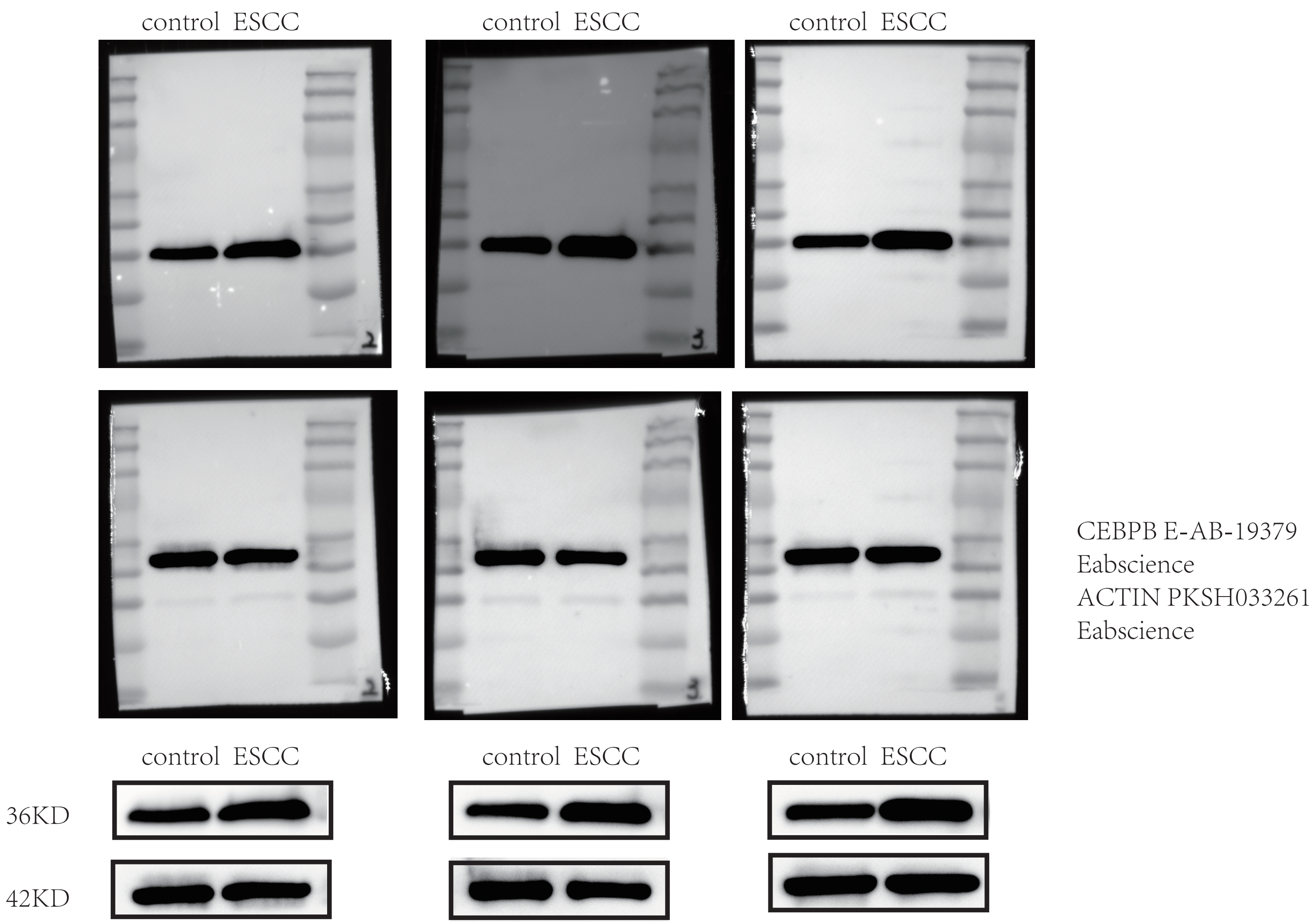

Doc.S9 Quantitative analysis of CEBPB protein expression by Western blotting in paired EEC and adjacent normal esophageal tissues (n=3)
