# Supplementary material for: Gene Expression Profiling and Clinical Validation Reveal CEBPB for Early Diagnosis of Esophageal Squamous Cell Carcinoma
Source: Cancer Rep (Hoboken). 2026 Nov 8;9(11):e70753. doi: 10.1002/cnr2.70753 (PMC13745642; doi:10.1002/cnr2.70753)
Supplement: Supplementary file 7 [file CNR2-9-e70753-s014.pdf]

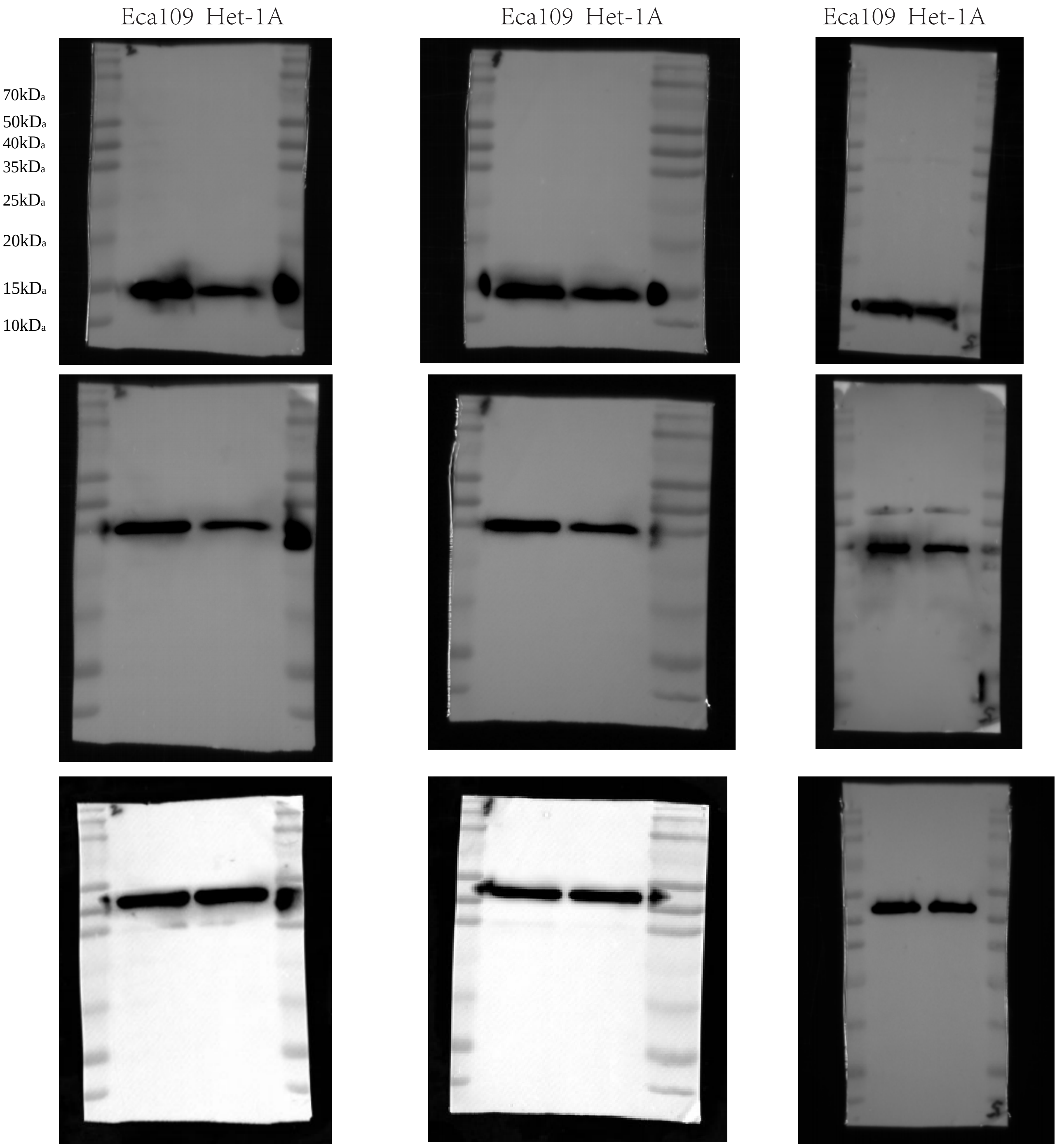

Doc. S12. Western blotting analysis of CEBPB and CXCL8 protein expression in Eca109 and Het-1A cells (n=3) .
