# Supplementary material for: Gene Expression Profiling and Clinical Validation Reveal CEBPB for Early Diagnosis of Esophageal Squamous Cell Carcinoma
Source: Cancer Rep (Hoboken). 2026 Nov 8;9(11):e70753. doi: 10.1002/cnr2.70753 (PMC13745642; doi:10.1002/cnr2.70753)

Doc.S10 Quantitative analysis of CXCL8 protein expression by Western blotting in paired EEC and adjacent normal esophageal tissues (n=3).

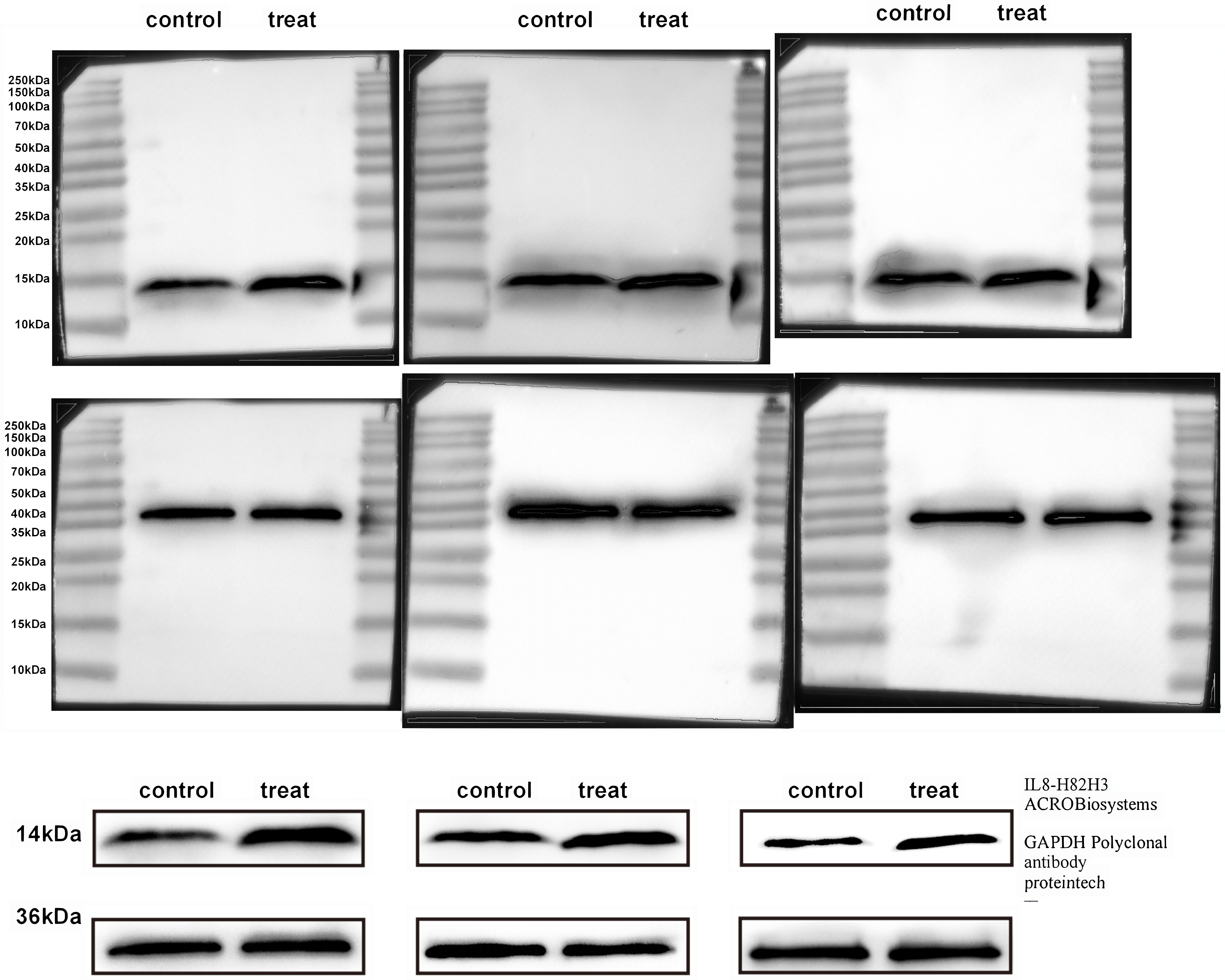

Supplement: Supplementary file 8 [file CNR2-9-e70753-s016.pdf]
