# Supplementary material for: Gene Expression Profiling and Clinical Validation Reveal CEBPB for Early Diagnosis of Esophageal Squamous Cell Carcinoma
Source: Cancer Rep (Hoboken). 2026 Nov 8;9(11):e70753. doi: 10.1002/cnr2.70753 (PMC13745642; doi:10.1002/cnr2.70753)
Supplement: Supplementary file 10 [file CNR2-9-e70753-s003.doc]

Doc. S6. KEGG pathway enrichment analysis of the downregulated DEGs.

category	subcategory	ID	Description	GeneRatio	BgRatio	pvalue	p.adjust	qvalue	geneID	Count

Genetic Information Processing	Translation	hsa03010	Ribosome	33/350	172/8846	2.05190519751294e-14	6.17623464451394e-12	5.81013155927348e-12	MRPL23/MRPS11/MRPS18C/RNA5-8SN1/RNA5-8SN2/RNA5-8SN3/RPL13/RPL14/RPL15/RPL17/RPL17-C18orf32/RPL18/RPL22/RPL23/RPL26/RPL27A/RPL30/RPL31/RPL32/RPL34/RPL36A/RPL37/RPL37A/RPL39/RPS10-NUDT3/RPS15A/RPS19/RPS23/RPS24/RPS27L/RPS29/RPS3/RPS9	33

Human Diseases	Infectious disease: viral	hsa05171	Coronavirus disease - COVID-19	37/350	238/8846	4.89119172382451e-13	7.36124354435589e-11	6.92489775636207e-11	C4A/C4B/C7/CFB/CFD/IKBKB/IKBKG/IRF9/PIK3R2/RPL13/RPL14/RPL15/RPL17/RPL17-C18orf32/RPL18/RPL22/RPL23/RPL26/RPL27A/RPL30/RPL31/RPL32/RPL34/RPL36A/RPL37/RPL37A/RPL39/RPS10-NUDT3/RPS15A/RPS19/RPS23/RPS24/RPS27L/RPS29/RPS3/RPS9/TMPRSS2	37

Human Diseases	Cancer: overview	hsa05208	Chemical carcinogenesis - reactive oxygen species	25/350	226/8846	2.93704502557642e-06	0.000294683517566167	0.000277215828729844	ATP5MC3/ATP5PD/ATP5PO/CYP2E1/EPHX2/GSTM2/IKBKB/IKBKG/MGST1/MGST2/MGST3/NDUFA10/NDUFA11/NDUFA13/NDUFA7/NDUFA9/NDUFB1/NDUFB2/NDUFB8/NDUFC2/NDUFS7/NDUFV2/PIK3R2/SDHC/UQCRB	25

Metabolism	Energy metabolism	hsa00190	Oxidative phosphorylation	17/350	138/8846	2.93509665221911e-05	0.00220866023079488	0.00207773947222879	ATP5MC3/ATP5PD/ATP5PO/ATP6V0C/NDUFA10/NDUFA11/NDUFA13/NDUFA7/NDUFA9/NDUFB1/NDUFB2/NDUFB8/NDUFC2/NDUFS7/NDUFV2/SDHC/UQCRB	17

Human Diseases	Endocrine and metabolic disease	hsa04932	Non-alcoholic fatty liver disease	17/350	157/8846	0.000150816960695127	0.00768203384331031	0.0072266728583685	CYP2E1/IKBKB/NDUFA10/NDUFA11/NDUFA13/NDUFA7/NDUFA9/NDUFB1/NDUFB2/NDUFB8/NDUFC2/NDUFS7/NDUFV2/PIK3R2/SDHC/UQCRB/XBP1	17

Human Diseases	Cardiovascular disease	hsa05415	Diabetic cardiomyopathy	20/350	205/8846	0.000171725482931494	0.00768203384331031	0.0072266728583685	ATP5MC3/ATP5PD/ATP5PO/CPT1B/NDUFA10/NDUFA11/NDUFA13/NDUFA7/NDUFA9/NDUFB1/NDUFB2/NDUFB8/NDUFC2/NDUFS7/NDUFV2/PDK4/PIK3R2/SDHC/SMAD2/UQCRB	20

Human Diseases	Neurodegenerative disease	hsa05012	Parkinson disease	24/350	271/8846	0.000178651949844426	0.00768203384331031	0.0072266728583685	ATP5MC3/ATP5PD/ATP5PO/KLC1/NDUFA10/NDUFA11/NDUFA13/NDUFA7/NDUFA9/NDUFB1/NDUFB2/NDUFB8/NDUFC2/NDUFS7/NDUFV2/PINK1/PSMA2/PSMA6/PSMC1/PSMD9/SDHC/SEM1/UQCRB/XBP1	24

Human Diseases	Neurodegenerative disease	hsa05016	Huntington disease	26/350	311/8846	0.000244298463176468	0.00878317164145009	0.00826253950533336	ATP5MC3/ATP5PD/ATP5PO/GPX3/KLC1/NDUFA10/NDUFA11/NDUFA13/NDUFA7/NDUFA9/NDUFB1/NDUFB2/NDUFB8/NDUFC2/NDUFS7/NDUFV2/PIK3C3/POLR2F/POLR2J3/PSMA2/PSMA6/PSMC1/PSMD9/SDHC/SEM1/UQCRB	26

Human Diseases	Neurodegenerative disease	hsa05020	Prion disease	24/350	278/8846	0.000262619750076581	0.00878317164145009	0.00826253950533336	ATP5MC3/ATP5PD/ATP5PO/C7/KLC1/NDUFA10/NDUFA11/NDUFA13/NDUFA7/NDUFA9/NDUFB1/NDUFB2/NDUFB8/NDUFC2/NDUFS7/NDUFV2/PIK3R2/PSMA2/PSMA6/PSMC1/PSMD9/SDHC/SEM1/UQCRB	24

Organismal Systems	Environmental adaptation	hsa04714	Thermogenesis	21/350	235/8846	0.000399645146803881	0.0120293189187968	0.0113162678410783	ADCY4/ATP5MC3/ATP5PD/ATP5PO/COX20/CPT1B/MGLL/NDUFA10/NDUFA11/NDUFA13/NDUFA7/NDUFA9/NDUFB1/NDUFB2/NDUFB8/NDUFC2/NDUFS7/NDUFV2/SDHC/SMARCE1/UQCRB	21

Human Diseases	Cancer: overview	hsa05204	Chemical carcinogenesis - DNA adducts	10/350	71/8846	0.000443526732734595	0.0121365042321012	0.0114170996273307	CYP2C18/CYP2E1/CYP3A5/GSTM2/MGST1/MGST2/MGST3/SULT1A3/UGT1A6/UGT1A7	10

Metabolism	Xenobiotics biodegradation and metabolism	hsa00982	Drug metabolism - cytochrome P450	10/350	73/8846	0.000556430092637499	0.0139571214903239	0.013129797799955	ADH1B/CYP2E1/CYP3A5/FMO2/GSTM2/MGST1/MGST2/MGST3/UGT1A6/UGT1A7	10

Human Diseases	Neurodegenerative disease	hsa05014	Amyotrophic lateral sclerosis	28/350	371/8846	0.000749184545546233	0.0173465037084166	0.016318270668173	ATP5MC3/ATP5PD/ATP5PO/GPX3/HDAC6/KLC1/MATR3/NDUFA10/NDUFA11/NDUFA13/NDUFA7/NDUFA9/NDUFB1/NDUFB2/NDUFB8/NDUFC2/NDUFS7/NDUFV2/PIK3C3/PINK1/PSMA2/PSMA6/PSMC1/PSMD9/SDHC/SEM1/UQCRB/XBP1	28

Metabolism	Xenobiotics biodegradation and metabolism	hsa00983	Drug metabolism - other enzymes	10/350	81/8846	0.00127161756413643	0.0273397776289332	0.025719182312233	CYP2E1/GSTM2/MGST1/MGST2/MGST3/NME1-NME2/NME2/UGT1A6/UGT1A7/UPP1	10

Metabolism	Lipid metabolism	hsa00590	Arachidonic acid metabolism	8/350	61/8846	0.00261064496498128	0.0523869422972911	0.0492816488126291	ALOX15B/CYP2E1/EPHX2/HPGD/LTC4S/PLA2G2A/PLA2G4B/PTGDS	8

Human Diseases	Neurodegenerative disease	hsa05010	Alzheimer disease	27/350	391/8846	0.00334335080614216	0.0628967870405493	0.0591685109771211	ATP5MC3/ATP5PD/ATP5PO/IKBKB/IKBKG/KLC1/NDUFA10/NDUFA11/NDUFA13/NDUFA7/NDUFA9/NDUFB1/NDUFB2/NDUFB8/NDUFC2/NDUFS7/NDUFV2/PIK3C3/PIK3R2/PSMA2/PSMA6/PSMC1/PSMD9/SDHC/SEM1/UQCRB/XBP1	27

Metabolism	Xenobiotics biodegradation and metabolism	hsa00980	Metabolism of xenobiotics by cytochrome P450	9/350	79/8846	0.00383186783452567	0.067846601070131	0.0638249193490653	ADH1B/CYP2E1/CYP3A5/GSTM2/MGST1/MGST2/MGST3/UGT1A6/UGT1A7	9

Organismal Systems	Nervous system	hsa04723	Retrograde endocannabinoid signaling	13/350	149/8846	0.00606824531619373	0.101474546676351	0.0954595315822289	ADCY4/MGLL/NDUFA10/NDUFA11/NDUFA13/NDUFA7/NDUFA9/NDUFB1/NDUFB2/NDUFB8/NDUFC2/NDUFS7/NDUFV2	13

Metabolism	Lipid metabolism	hsa00071	Fatty acid degradation	6/350	43/8846	0.00649691530878941	0.102924816207664	0.096823834795809	ACAA1/ACADVL/ADH1B/ALDH2/ALDH7A1/CPT1B	6

Metabolism	Metabolism of cofactors and vitamins	hsa00830	Retinol metabolism	7/350	68/8846	0.0174747254347011	0.262994617792251	0.247405323259715	ADH1B/CYP2C18/CYP3A5/RDH13/RDH5/UGT1A6/UGT1A7	7

Organismal Systems	Digestive system	hsa04972	Pancreatic secretion	9/350	106/8846	0.0242480870529101	0.347555914425046	0.326954156252272	ADCY4/AMY2B/CLCA4/PLA2G2A/PRSS1/PRSS2/PRSS3/RAB11A/RAP1B	9

Metabolism	Metabolism of other amino acids	hsa00480	Glutathione metabolism	6/350	58/8846	0.026370419647275	0.360795286992263	0.339408750484066	GGT6/GPX3/GSTM2/MGST1/MGST2/MGST3	6

Metabolism	Lipid metabolism	hsa00120	Primary bile acid biosynthesis	3/350	17/8846	0.0276469320517497	0.361814197720724	0.340367264161129	ACOX2/AMACR/HSD17B4	3

Metabolism	Carbohydrate metabolism	hsa00053	Ascorbate and aldarate metabolism	4/350	30/8846	0.0293009959801676	0.367483324584602	0.345700347309872	ALDH2/ALDH7A1/UGT1A6/UGT1A7	4

Environmental Information Processing	Membrane transport	hsa02010	ABC transporters	5/350	45/8846	0.0314917493371745	0.379160662019581	0.356685497755787	ABCA5/ABCA6/ABCA8/ABCB6/ABCD4	5

Genetic Information Processing	Folding, sorting and degradation	hsa03050	Proteasome	5/350	46/8846	0.034229558517481	0.396272965913914	0.372783451060825	PSMA2/PSMA6/PSMC1/PSMD9/SEM1	5

Genetic Information Processing	Replication and repair	hsa03420	Nucleotide excision repair	6/350	63/8846	0.0376287687804917	0.41612888890725	0.39146239243242	ERCC5/GTF2H2C/GTF2H4/POLD4/POLR2F/POLR2J3	6

Human Diseases	Infectious disease: viral	hsa05164	Influenza A	12/350	172/8846	0.0400857606578991	0.41612888890725	0.39146239243242	IKBKB/IKBKG/IRF9/PIK3R2/PRSS1/PRSS2/PRSS3/RAB11A/TMPRSS2/TMPRSS4/TPSAB1/TPSB2	12

Metabolism	Global and overview maps	hsa01240	Biosynthesis of cofactors	11/350	153/8846	0.0400921520874095	0.41612888890725	0.39146239243242	AK1/ALDH2/COQ6/MMAB/NADSYN1/NME1-NME2/NME2/PMM1/RDH13/UGT1A6/UGT1A7	11

NA	NA	hsa04820	Cytoskeleton in muscle cells	15/350	232/8846	0.0419486368676745	0.420884656572334	0.395936256751033	ACTG2/CAPN3/CSRP2/DCN/DES/ELN/FHL1/ITGA7/MYH11/MYL5/PDLIM2/SYNE1/TNNI2/TPM1/TPM2	15

Cellular Processes	Transport and catabolism	hsa04146	Peroxisome	7/350	83/8846	0.0455430013932902	0.43856486027745	0.412568446982459	ABCD4/ACAA1/ACOX2/AMACR/EPHX2/HSD17B4/MVK	7

Metabolism	Amino acid metabolism	hsa00330	Arginine and proline metabolism	5/350	50/8846	0.0466248356441143	0.43856486027745	0.412568446982459	ALDH2/ALDH7A1/CKMT1A/CKMT1B/PRODH	5

Environmental Information Processing	Signal transduction	hsa04014	Ras signaling pathway	15/350	238/8846	0.0504548514198284	0.460209402344495	0.43292998506966	AFDN/FLT3LG/IKBKB/IKBKG/KSR1/LAT/PAK6/PIK3R2/PLA2G2A/PLA2G4B/RAP1B/RASA4/RASA4B/RASAL1/RASGRP2	15

Metabolism	Global and overview maps	hsa01250	Biosynthesis of nucleotide sugars	4/350	37/8846	0.0570398376072545	0.495307366929861	0.465947479294047	GALE/GMDS/NAGK/PMM1	4

Organismal Systems	Immune system	hsa04610	Complement and coagulation cascades	7/350	88/8846	0.059091558468871	0.495307366929861	0.465947479294047	C4A/C4B/C7/CFB/CFD/CLU/SERPINB2	7

Human Diseases	Endocrine and metabolic disease	hsa04936	Alcoholic liver disease	10/350	144/8846	0.0592394193005814	0.495307366929861	0.465947479294047	ACADVL/ADH1B/ALDH2/ALDH7A1/C4A/C4B/CPT1B/CYP2E1/IKBKB/IKBKG	10

Human Diseases	Neurodegenerative disease	hsa05022	Pathways of neurodegeneration - multiple diseases	26/350	483/8846	0.0671887221044234	0.546589333876525	0.514189651382358	ATP5MC3/ATP5PD/ATP5PO/GPX3/KLC1/NDUFA10/NDUFA11/NDUFA13/NDUFA7/NDUFA9/NDUFB1/NDUFB2/NDUFB8/NDUFC2/NDUFS7/NDUFV2/PIK3C3/PINK1/PSMA2/PSMA6/PSMC1/PSMD9/SDHC/SEM1/UQCRB/XBP1	26

Metabolism	Lipid metabolism	hsa00592	alpha-Linolenic acid metabolism	3/350	26/8846	0.08160878192636	0.646427456837746	0.608109760060688	ACAA1/PLA2G2A/PLA2G4B	3

Organismal Systems	Circulatory system	hsa04270	Vascular smooth muscle contraction	9/350	134/8846	0.083797749529123	0.646746733545283	0.608410111291069	ACTG2/ADCY4/EDN3/MYH11/MYL6/PLA2G2A/PLA2G4B/PPP1R12C/PPP1R14A	9

Organismal Systems	Immune system	hsa04670	Leukocyte transendothelial migration	8/350	116/8846	0.0885592470316324	0.666408333913034	0.626906248723924	AFDN/CLDN5/CLDN7/CXCL12/MYL5/PIK3R2/RAP1B/RAPGEF3	8

Metabolism	Lipid metabolism	hsa00140	Steroid hormone biosynthesis	5/350	62/8846	0.0978368477285488	0.718265638202273	0.675689654402558	CYP2E1/CYP3A5/SULT2B1/UGT1A6/UGT1A7	5

Human Diseases	Infectious disease: bacterial	hsa05150	Staphylococcus aureus infection	7/350	100/8846	0.100777763825547	0.722240640749754	0.679429034312586	C4A/C4B/CFB/CFD/KRT13/KRT15/KRT24	7

Metabolism	Lipid metabolism	hsa00591	Linoleic acid metabolism	3/350	30/8846	0.113790249734372	0.724029540889308	0.681111895433551	CYP2E1/PLA2G2A/PLA2G4B	3

Metabolism	Lipid metabolism	hsa00561	Glycerolipid metabolism	5/350	65/8846	0.113808126341697	0.724029540889308	0.681111895433551	ABHD16A/ALDH2/ALDH7A1/DGKA/MGLL	5

Metabolism	Metabolism of cofactors and vitamins	hsa00730	Thiamine metabolism	2/350	15/8846	0.116845967288839	0.724029540889308	0.681111895433551	AK1/NTPCR	2

Metabolism	Global and overview maps	hsa01232	Nucleotide metabolism	6/350	85/8846	0.119693978092827	0.724029540889308	0.681111895433551	AK1/GUK1/NME1-NME2/NME2/NTPCR/UPP1	6

Metabolism	Amino acid metabolism	hsa00280	Valine, leucine and isoleucine degradation	4/350	48/8846	0.120771215876713	0.724029540889308	0.681111895433551	ACAA1/ALDH2/ALDH7A1/BCKDHA	4

Environmental Information Processing	Signal transduction	hsa04064	NF-kappa B signaling pathway	7/350	105/8846	0.121909893096938	0.724029540889308	0.681111895433551	BLNK/CCL19/CFLAR/CXCL12/IKBKB/IKBKG/LAT	7

Cellular Processes	Transport and catabolism	hsa04137	Mitophagy - animal	7/350	105/8846	0.121909893096938	0.724029540889308	0.681111895433551	ATG9B/CCZ1/CCZ1B/GABARAP/PINK1/RABGEF1/USP15	7

Genetic Information Processing	Folding, sorting and degradation	hsa03060	Protein export	3/350	31/8846	0.12248636422642	0.724029540889308	0.681111895433551	GET4/SEC11C/SRP19	3

Metabolism	Metabolism of other amino acids	hsa00430	Taurine and hypotaurine metabolism	2/350	16/8846	0.130189705321969	0.724029540889308	0.681111895433551	FMO2/GGT6	2

Cellular Processes	Transport and catabolism	hsa04136	Autophagy - other	3/350	32/8846	0.131416282363915	0.724029540889308	0.681111895433551	ATG9B/GABARAP/PIK3C3	3

Metabolism	Nucleotide metabolism	hsa00230	Purine metabolism	8/350	128/8846	0.134425989445257	0.724029540889308	0.681111895433551	ADCY4/AK1/GUK1/NME1-NME2/NME2/NTPCR/PDE2A/PDE4C	8

Metabolism	Carbohydrate metabolism	hsa00520	Amino sugar and nucleotide sugar metabolism	4/350	50/8846	0.134703170398011	0.724029540889308	0.681111895433551	GALE/GMDS/NAGK/PMM1	4

Metabolism	Lipid metabolism	hsa00565	Ether lipid metabolism	4/350	50/8846	0.134703170398011	0.724029540889308	0.681111895433551	GDPD3/LPCAT4/PLA2G2A/PLA2G4B	4

Human Diseases	Infectious disease: parasitic	hsa05144	Malaria	4/350	50/8846	0.134703170398011	0.724029540889308	0.681111895433551	ACKR1/HBA1/HBA2/HBB	4

Metabolism	Carbohydrate metabolism	hsa00051	Fructose and mannose metabolism	3/350	34/8846	0.149923588075676	0.79170175457506	0.744772764401788	ENOSF1/GMDS/PMM1	3

NA	NA	hsa04980	Cobalamin transport and metabolism	2/350	18/8846	0.157816382319198	0.819012604794461	0.770464734008428	ABCD4/MMAB	2

Human Diseases	Drug resistance: antineoplastic	hsa01524	Platinum drug resistance	5/350	75/8846	0.175010694152586	0.867660743868759	0.816229201261396	GSTM2/MGST1/MGST2/MGST3/PIK3R2	5

Human Diseases	Infectious disease: parasitic	hsa05143	African trypanosomiasis	3/350	37/8846	0.179101115119331	0.867660743868759	0.816229201261396	HBA1/HBA2/HBB	3

Organismal Systems	Endocrine system	hsa03320	PPAR signaling pathway	5/350	76/8846	0.181725273111057	0.867660743868759	0.816229201261396	ACAA1/ACOX2/CPT1B/DBI/ILK	5

Metabolism	Lipid metabolism	hsa00100	Steroid biosynthesis	2/350	20/8846	0.186399664384657	0.867660743868759	0.816229201261396	CYP51A1/TM7SF2	2

Metabolism	Metabolism of cofactors and vitamins	hsa00785	Lipoic acid metabolism	2/350	20/8846	0.186399664384657	0.867660743868759	0.816229201261396	AMT/BCKDHA	2

Metabolism	Global and overview maps	hsa01212	Fatty acid metabolism	4/350	57/8846	0.188029923792112	0.867660743868759	0.816229201261396	ACAA1/ACADVL/CPT1B/HSD17B4	4

Organismal Systems	Excretory system	hsa04960	Aldosterone-regulated sodium reabsorption	3/350	38/8846	0.18915138056513	0.867660743868759	0.816229201261396	PIK3R2/SCNN1A/SCNN1B	3

Environmental Information Processing	Signal transduction	hsa04070	Phosphatidylinositol signaling system	6/350	98/8846	0.191553000179152	0.867660743868759	0.816229201261396	DGKA/IP6K2/IPPK/MTMR3/PIK3C3/PIK3R2	6

Organismal Systems	Development and regeneration	hsa04360	Axon guidance	10/350	184/8846	0.193133786841219	0.867660743868759	0.816229201261396	ABLIM3/BOC/CXCL12/ILK/MYL5/NFATC4/PAK6/PIK3R2/ROBO3/SEMA4D	10

Environmental Information Processing	Signaling molecules and interaction	hsa04061	Viral protein interaction with cytokine and cytokine receptor	6/350	100/8846	0.203849676047841	0.902334595447061	0.848847722242558	CCL14/CCL19/CXCL12/CXCR2/IL34/TNFRSF14	6

Human Diseases	Neurodegenerative disease	hsa05017	Spinocerebellar ataxia	8/350	144/8846	0.210617692929192	0.918781530024446	0.864319746726966	PIK3C3/PIK3R2/PSMA2/PSMA6/PSMC1/PSMD9/SEM1/XBP1	8

Metabolism	Amino acid metabolism	hsa00340	Histidine metabolism	2/350	22/8846	0.215626125779191	0.921613564328635	0.866983909090411	ALDH2/ALDH7A1	2

Genetic Information Processing	Replication and repair	hsa03440	Homologous recombination	3/350	41/8846	0.22009373940331	0.921613564328635	0.866983909090411	POLD4/SEM1/TOP3B	3

Organismal Systems	Digestive system	hsa04978	Mineral absorption	4/350	61/8846	0.221104553648681	0.921613564328635	0.866983909090411	ATOX1/MT1G/MT1X/TF	4

Metabolism	Metabolism of terpenoids and polyketides	hsa00900	Terpenoid backbone biosynthesis	2/350	23/8846	0.230394391000736	0.921613564328635	0.866983909090411	MVD/MVK	2

Organismal Systems	Immune system	hsa04623	Cytosolic DNA-sensing pathway	5/350	83/8846	0.231155806884167	0.921613564328635	0.866983909090411	IKBKB/IKBKG/POLR2F/RIPK3/TREX1	5

Organismal Systems	Immune system	hsa04062	Chemokine signaling pathway	10/350	193/8846	0.23425821453013	0.921613564328635	0.866983909090411	ADCY4/CCL14/CCL19/CXCL12/CXCR2/IKBKB/IKBKG/PIK3R2/RAP1B/RASGRP2	10

Organismal Systems	Immune system	hsa04625	C-type lectin receptor signaling pathway	6/350	105/8846	0.235761609479418	0.921613564328635	0.866983909090411	IKBKB/IKBKG/IRF9/KSR1/NFATC4/PIK3R2	6

Organismal Systems	Digestive system	hsa04974	Protein digestion and absorption	6/350	105/8846	0.235761609479418	0.921613564328635	0.866983909090411	ELN/PGA3/PGA4/PRSS1/PRSS2/PRSS3	6

Genetic Information Processing	Transcription	hsa03022	Basal transcription factors	3/350	44/8846	0.251962838475786	0.961721754403041	0.904714642190656	GTF2H2C/GTF2H4/TAF10	3

Cellular Processes	Cell motility	hsa04814	Motor proteins	10/350	197/8846	0.253521026819931	0.961721754403041	0.904714642190656	ACTG2/BICDL2/KLC1/MYH11/MYL5/MYL6/MYO15B/TNNI2/TPM1/TPM2	10

Environmental Information Processing	Signal transduction	hsa04350	TGF-beta signaling pathway	6/350	108/8846	0.255607110804795	0.961721754403041	0.904714642190656	DCN/NBL1/SKP1/SMAD2/TF/THSD4	6

Metabolism	Metabolism of cofactors and vitamins	hsa00860	Porphyrin metabolism	3/350	46/8846	0.273566695845139	0.999999993029775	0.940723896223149	MMAB/UGT1A6/UGT1A7	3

Human Diseases	Infectious disease: bacterial	hsa05131	Shigellosis	12/350	249/8846	0.281930369712937	0.999999993029775	0.940723896223149	CYTH2/IKBKB/IKBKG/ILK/MYL5/NAIP/PIK3C3/PIK3R2/SKP1/U2AF1/U2AF1L4/UBE2V1	12

Metabolism	Carbohydrate metabolism	hsa00620	Pyruvate metabolism	3/350	47/8846	0.284442723653713	0.999999993029775	0.940723896223149	ADH1B/ALDH2/ALDH7A1	3

Metabolism	Lipid metabolism	hsa01040	Biosynthesis of unsaturated fatty acids	2/350	27/8846	0.289821372451345	0.999999993029775	0.940723896223149	ACAA1/HSD17B4	2

Human Diseases	Cancer: specific types	hsa05211	Renal cell carcinoma	4/350	70/8846	0.299990286471493	0.999999993029775	0.940723896223149	EGLN2/PAK6/PIK3R2/RAP1B	4

Metabolism	Glycan biosynthesis and metabolism	hsa00601	Glycosphingolipid biosynthesis - lacto and neolacto series	2/350	28/8846	0.304642030457566	0.999999993029775	0.940723896223149	FUT3/FUT6	2

Human Diseases	Infectious disease: bacterial	hsa05120	Epithelial cell signaling in Helicobacter pylori infection	4/350	71/8846	0.308994711170902	0.999999993029775	0.940723896223149	ATP6V0C/CXCR2/IKBKB/IKBKG	4

Human Diseases	Cardiovascular disease	hsa05418	Fluid shear stress and atherosclerosis	7/350	141/8846	0.324402506036383	0.999999993029775	0.940723896223149	GSTM2/IKBKB/IKBKG/MGST1/MGST2/MGST3/PIK3R2	7

Metabolism	Carbohydrate metabolism	hsa00562	Inositol phosphate metabolism	4/350	73/8846	0.327077681763432	0.999999993029775	0.940723896223149	IPPK/MTMR3/PIK3C3/PLCH2	4

Metabolism	Energy metabolism	hsa00920	Sulfur metabolism	1/350	10/8846	0.332295032146552	0.999999993029775	0.940723896223149	SELENBP1	1

Human Diseases	Drug resistance: antineoplastic	hsa01523	Antifolate resistance	2/350	30/8846	0.334091816866255	0.999999993029775	0.940723896223149	IKBKB/IKBKG	2

Metabolism	Metabolism of other amino acids	hsa00410	beta-Alanine metabolism	2/350	31/8846	0.348685296358658	0.999999993029775	0.940723896223149	ALDH2/ALDH7A1	2

Metabolism	Metabolism of cofactors and vitamins	hsa00130	Ubiquinone and other terpenoid-quinone biosynthesis	1/350	11/8846	0.358743282344459	0.999999993029775	0.940723896223149	COQ6	1

Cellular Processes	Cellular community - eukaryotes	hsa04530	Tight junction	8/350	170/8846	0.359249703114874	0.999999993029775	0.940723896223149	AFDN/ARHGEF18/CLDN5/CLDN7/LLGL2/MYH11/MYL6/TJP3	8

Human Diseases	Cancer: specific types	hsa05212	Pancreatic cancer	4/350	77/8846	0.363389979529238	0.999999993029775	0.940723896223149	IKBKB/IKBKG/PIK3R2/SMAD2	4

Genetic Information Processing	Replication and repair	hsa03460	Fanconi anemia pathway	3/350	55/8846	0.371924946891117	0.999999993029775	0.940723896223149	SLX1A/SLX1B/TOP3B	3

Environmental Information Processing	Signal transduction	hsa04072	Phospholipase D signaling pathway	7/350	149/8846	0.376318540013583	0.999999993029775	0.940723896223149	ADCY4/CXCR2/CYTH2/DGKA/PIK3R2/PLA2G4B/RAPGEF3	7

NA	NA	hsa01210	2-Oxocarboxylic acid metabolism	2/350	33/8846	0.377531405039418	0.999999993029775	0.940723896223149	ACY1/BCKDHA	2

Organismal Systems	Immune system	hsa04611	Platelet activation	6/350	126/8846	0.381311466378216	0.999999993029775	0.940723896223149	ADCY4/P2RX1/PIK3R2/PLA2G4B/RAP1B/RASGRP2	6

Metabolism	Lipid metabolism	hsa00564	Glycerophospholipid metabolism	5/350	103/8846	0.386181866165427	0.999999993029775	0.940723896223149	CHKB/DGKA/LPCAT4/PLA2G2A/PLA2G4B	5

Human Diseases	Infectious disease: parasitic	hsa05142	Chagas disease	5/350	103/8846	0.386181866165427	0.999999993029775	0.940723896223149	CFLAR/IKBKB/IKBKG/PIK3R2/SMAD2	5

Genetic Information Processing	Transcription	hsa03020	RNA polymerase	2/350	34/8846	0.391756893599652	0.999999993029775	0.940723896223149	POLR2F/POLR2J3	2

Organismal Systems	Environmental adaptation	hsa04710	Circadian rhythm	2/350	34/8846	0.391756893599652	0.999999993029775	0.940723896223149	NPAS2/SKP1	2

Human Diseases	Cardiovascular disease	hsa05414	Dilated cardiomyopathy	5/350	105/8846	0.402024715994842	0.999999993029775	0.940723896223149	ADCY4/DES/ITGA7/TPM1/TPM2	5

Metabolism	Nucleotide metabolism	hsa00240	Pyrimidine metabolism	3/350	58/8846	0.404377605430547	0.999999993029775	0.940723896223149	NME1-NME2/NME2/UPP1	3

Cellular Processes	Cellular community - eukaryotes	hsa04510	Focal adhesion	9/350	203/8846	0.411955613647074	0.999999993029775	0.940723896223149	EMP1/ILK/ITGA7/MYL5/PAK6/PIK3R2/PPP1R12C/RAP1B/TNXB	9

Organismal Systems	Endocrine system	hsa04923	Regulation of lipolysis in adipocytes	3/350	59/8846	0.415092379084348	0.999999993029775	0.940723896223149	ADCY4/MGLL/PIK3R2	3

Metabolism	Carbohydrate metabolism	hsa00040	Pentose and glucuronate interconversions	2/350	36/8846	0.419754081174128	0.999999993029775	0.940723896223149	UGT1A6/UGT1A7	2

Human Diseases	Cancer: overview	hsa05203	Viral carcinogenesis	9/350	205/8846	0.423355320870029	0.999999993029775	0.940723896223149	GTF2H2C/GTF2H4/HDAC10/HDAC6/IKBKG/IRF9/PIK3R2/PSMC1/SCIN	9

Human Diseases	Infectious disease: viral	hsa05160	Hepatitis C	7/350	159/8846	0.441622739821404	0.999999993029775	0.940723896223149	CFLAR/CLDN5/CLDN7/IKBKB/IKBKG/IRF9/PIK3R2	7

Organismal Systems	Aging	hsa04213	Longevity regulating pathway - multiple species	3/350	62/8846	0.446841393596519	0.999999993029775	0.940723896223149	ADCY4/CRYAB/PIK3R2	3

Metabolism	Metabolism of cofactors and vitamins	hsa00760	Nicotinate and nicotinamide metabolism	2/350	38/8846	0.447080918049362	0.999999993029775	0.940723896223149	NADSYN1/SIRT7	2

Human Diseases	Immune disease	hsa05340	Primary immunodeficiency	2/350	38/8846	0.447080918049362	0.999999993029775	0.940723896223149	BLNK/IKBKG	2

Metabolism	Amino acid metabolism	hsa00310	Lysine degradation	3/350	63/8846	0.457274094622456	0.999999993029775	0.940723896223149	ALDH2/ALDH7A1/PHYKPL	3

Human Diseases	Cancer: overview	hsa05235	PD-L1 expression and PD-1 checkpoint pathway in cancer	4/350	90/8846	0.479456324352208	0.999999993029775	0.940723896223149	IKBKB/IKBKG/LAT/PIK3R2	4

Human Diseases	Cancer: overview	hsa05207	Chemical carcinogenesis - receptor activation	9/350	215/8846	0.480018747817591	0.999999993029775	0.940723896223149	ADCY4/EPHX2/GSTM2/MGST1/MGST2/MGST3/PIK3R2/UGT1A6/UGT1A7	9

Metabolism	Amino acid metabolism	hsa00260	Glycine, serine and threonine metabolism	2/350	41/8846	0.486676887406128	0.999999993029775	0.940723896223149	ALDH7A1/AMT	2

Organismal Systems	Immune system	hsa04662	B cell receptor signaling pathway	4/350	91/8846	0.488092214347527	0.999999993029775	0.940723896223149	BLNK/IKBKB/IKBKG/PIK3R2	4

Metabolism	Carbohydrate metabolism	hsa00010	Glycolysis / Gluconeogenesis	3/350	67/8846	0.498123666424879	0.999999993029775	0.940723896223149	ADH1B/ALDH2/ALDH7A1	3

Metabolism	Amino acid metabolism	hsa00380	Tryptophan metabolism	2/350	42/8846	0.49947923654105	0.999999993029775	0.940723896223149	ALDH2/ALDH7A1	2

Cellular Processes	Cell growth and death	hsa04216	Ferroptosis	2/350	42/8846	0.49947923654105	0.999999993029775	0.940723896223149	PCBP2/TF	2

Cellular Processes	Cellular community - eukaryotes	hsa04520	Adherens junction	4/350	93/8846	0.505185628094256	0.999999993029775	0.940723896223149	AFDN/LMO7/MYL5/RAP1B	4

Cellular Processes	Transport and catabolism	hsa04140	Autophagy - animal	7/350	169/8846	0.505769125086324	0.999999993029775	0.940723896223149	ATG16L2/ATG9B/CFLAR/GABARAP/MTMR3/PIK3C3/PIK3R2	7

Human Diseases	Cancer: specific types	hsa05221	Acute myeloid leukemia	3/350	68/8846	0.508094743442556	0.999999993029775	0.940723896223149	IKBKB/IKBKG/PIK3R2	3

Environmental Information Processing	Signal transduction	hsa04668	TNF signaling pathway	5/350	119/8846	0.510429553834568	0.999999993029775	0.940723896223149	CFLAR/IKBKB/IKBKG/PIK3R2/RIPK3	5

Human Diseases	Cancer: specific types	hsa05225	Hepatocellular carcinoma	7/350	170/8846	0.512061370201662	0.999999993029775	0.940723896223149	GSTM2/MGST1/MGST2/MGST3/PIK3R2/SMAD2/SMARCE1	7

Metabolism	Glycan biosynthesis and metabolism	hsa00511	Other glycan degradation	1/350	18/8846	0.516822863907198	0.999999993029775	0.940723896223149	MAN2C1	1

Organismal Systems	Immune system	hsa04664	Fc epsilon RI signaling pathway	3/350	69/8846	0.517961866868976	0.999999993029775	0.940723896223149	LAT/PIK3R2/PLA2G4B	3

Genetic Information Processing	Replication and repair	hsa03410	Base excision repair	2/350	44/8846	0.524461765719594	0.999999993029775	0.940723896223149	NEIL1/POLD4	2

Organismal Systems	Excretory system	hsa04962	Vasopressin-regulated water reabsorption	2/350	44/8846	0.524461765719594	0.999999993029775	0.940723896223149	AQP3/RAB11A	2

Organismal Systems	Endocrine system	hsa04920	Adipocytokine signaling pathway	3/350	70/8846	0.527721788379311	0.999999993029775	0.940723896223149	CPT1B/IKBKB/IKBKG	3

Organismal Systems	Immune system	hsa04660	T cell receptor signaling pathway	5/350	122/8846	0.532693483787155	0.999999993029775	0.940723896223149	IKBKB/IKBKG/LAT/PAK6/PIK3R2	5

Human Diseases	Infectious disease: bacterial	hsa05132	Salmonella infection	10/350	251/8846	0.537438668342183	0.999999993029775	0.940723896223149	CYTH2/IKBKB/IKBKG/KLC1/MYL5/NAIP/PIK3C3/RIPK3/RPS3/SKP1	10

Organismal Systems	Immune system	hsa04666	Fc gamma R-mediated phagocytosis	4/350	98/8846	0.546757365630688	0.999999993029775	0.940723896223149	LAT/PIK3R2/PLA2G4B/SCIN	4

Human Diseases	Cancer: specific types	hsa05215	Prostate cancer	4/350	98/8846	0.546757365630688	0.999999993029775	0.940723896223149	IKBKB/IKBKG/PIK3R2/TMPRSS2	4

Organismal Systems	Immune system	hsa04622	RIG-I-like receptor signaling pathway	3/350	72/8846	0.546908287701624	0.999999993029775	0.940723896223149	AZI2/IKBKB/IKBKG	3

Metabolism	Metabolism of cofactors and vitamins	hsa00670	One carbon pool by folate	1/350	20/8846	0.554378106544469	0.999999993029775	0.940723896223149	AMT	1

Human Diseases	Cancer: overview	hsa05231	Choline metabolism in cancer	4/350	99/8846	0.554854090636525	0.999999993029775	0.940723896223149	CHKB/DGKA/PIK3R2/PLA2G4B	4

Human Diseases	Cardiovascular disease	hsa05410	Hypertrophic cardiomyopathy	4/350	99/8846	0.554854090636525	0.999999993029775	0.940723896223149	DES/ITGA7/TPM1/TPM2	4

Human Diseases	Infectious disease: bacterial	hsa05130	Pathogenic Escherichia coli infection	8/350	203/8846	0.556020115343354	0.999999993029775	0.940723896223149	CLDN5/CLDN7/CYTH2/IKBKB/IKBKG/MYH11/NAIP/RPS3	8

Human Diseases	Endocrine and metabolic disease	hsa04930	Type II diabetes mellitus	2/350	47/8846	0.560332684751381	0.999999993029775	0.940723896223149	IKBKB/PIK3R2	2

Cellular Processes	Cell motility	hsa04810	Regulation of actin cytoskeleton	9/350	230/8846	0.562229210646604	0.999999993029775	0.940723896223149	C7/CXCL12/ITGA7/MYH11/MYL5/PAK6/PIK3R2/PPP1R12C/SCIN	9

Metabolism	Metabolism of cofactors and vitamins	hsa00770	Pantothenate and CoA biosynthesis	1/350	21/8846	0.572049493663145	0.999999993029775	0.940723896223149	ALDH2	1

Human Diseases	Cancer: specific types	hsa05220	Chronic myeloid leukemia	3/350	77/8846	0.592817715331332	0.999999993029775	0.940723896223149	IKBKB/IKBKG/PIK3R2	3

Human Diseases	Infectious disease: bacterial	hsa05100	Bacterial invasion of epithelial cells	3/350	78/8846	0.601631675376606	0.999999993029775	0.940723896223149	ARHGAP10/ILK/PIK3R2	3

Organismal Systems	Endocrine system	hsa04913	Ovarian steroidogenesis	2/350	51/8846	0.605092068942155	0.999999993029775	0.940723896223149	ADCY4/PLA2G4B	2

Metabolism	Amino acid metabolism	hsa00220	Arginine biosynthesis	1/350	23/8846	0.605323291535962	0.999999993029775	0.940723896223149	ACY1	1

Genetic Information Processing	Replication and repair	hsa03430	Mismatch repair	1/350	23/8846	0.605323291535962	0.999999993029775	0.940723896223149	POLD4	1

Environmental Information Processing	Signal transduction	hsa04015	Rap1 signaling pathway	8/350	212/8846	0.605673887752053	0.999999993029775	0.940723896223149	ADCY4/AFDN/EVL/LAT/PIK3R2/RAP1B/RAPGEF3/RASGRP2	8

Organismal Systems	Digestive system	hsa04973	Carbohydrate digestion and absorption	2/350	52/8846	0.615729431663953	0.999999993029775	0.940723896223149	AMY2B/PIK3R2	2

Metabolism	Glycan biosynthesis and metabolism	hsa00534	Glycosaminoglycan biosynthesis - heparan sulfate / heparin	1/350	24/8846	0.620979740358631	0.999999993029775	0.940723896223149	NDST2	1

Organismal Systems	Immune system	hsa04659	Th17 cell differentiation	4/350	108/8846	0.624057849804899	0.999999993029775	0.940723896223149	IKBKB/IKBKG/LAT/SMAD2	4

Cellular Processes	Cell growth and death	hsa04210	Apoptosis	5/350	136/8846	0.629830131440975	0.999999993029775	0.940723896223149	CFLAR/DIABLO/IKBKB/IKBKG/PIK3R2	5

Environmental Information Processing	Signal transduction	hsa04066	HIF-1 signaling pathway	4/350	109/8846	0.631315236068943	0.999999993029775	0.940723896223149	EGLN2/EIF4E/PIK3R2/TF	4

Organismal Systems	Immune system	hsa04620	Toll-like receptor signaling pathway	4/350	109/8846	0.631315236068943	0.999999993029775	0.940723896223149	IKBKB/IKBKG/IRF9/PIK3R2	4

Human Diseases	Endocrine and metabolic disease	hsa04931	Insulin resistance	4/350	109/8846	0.631315236068943	0.999999993029775	0.940723896223149	CPT1B/IKBKB/PIK3R2/PPP1R3C	4

Environmental Information Processing	Signaling molecules and interaction	hsa04060	Cytokine-cytokine receptor interaction	11/350	298/8846	0.637699004758043	0.999999993029775	0.940723896223149	CCL14/CCL19/CXCL12/CXCL17/CXCR2/IL11RA/IL1RN/IL34/IL36A/TNFRSF14/TNFRSF6B	11

Organismal Systems	Endocrine system	hsa04910	Insulin signaling pathway	5/350	138/8846	0.64267276333919	0.999999993029775	0.940723896223149	EIF4E/IKBKB/PIK3R2/PPP1R3C/TRIP10	5

Genetic Information Processing	Chromosome	hsa03083	Polycomb repressive complex	3/350	83/8846	0.643791450393539	0.999999993029775	0.940723896223149	COMMD3-BMI1/PHC1/SKP1	3

Organismal Systems	Endocrine system	hsa04915	Estrogen signaling pathway	5/350	139/8846	0.64898950353662	0.999999993029775	0.940723896223149	ADCY4/KRT13/KRT15/KRT24/PIK3R2	5

Metabolism	Glycan biosynthesis and metabolism	hsa00563	Glycosylphosphatidylinositol (GPI)-anchor biosynthesis	1/350	26/8846	0.650458956753396	0.999999993029775	0.940723896223149	MPPE1	1

Organismal Systems	Digestive system	hsa04977	Vitamin digestion and absorption	1/350	26/8846	0.650458956753396	0.999999993029775	0.940723896223149	ABCD4	1

Environmental Information Processing	Signal transduction	hsa04340	Hedgehog signaling pathway	2/350	56/8846	0.656081595574836	0.999999993029775	0.940723896223149	BOC/CUL3	2

Human Diseases	Infectious disease: bacterial	hsa05134	Legionellosis	2/350	56/8846	0.656081595574836	0.999999993029775	0.940723896223149	EEF1G/NAIP	2

Human Diseases	Infectious disease: viral	hsa05166	Human T-cell leukemia virus 1 infection	8/350	223/8846	0.662303987889921	0.999999993029775	0.940723896223149	ADCY4/ANAPC15/GPS2/IKBKB/IKBKG/NFATC4/PIK3R2/SMAD2	8

Organismal Systems	Development and regeneration	hsa04380	Osteoclast differentiation	5/350	142/8846	0.66751432587158	0.999999993029775	0.940723896223149	BLNK/IKBKB/IKBKG/IRF9/PIK3R2	5

Organismal Systems	Sensory system	hsa04742	Taste transduction	3/350	86/8846	0.667534766910971	0.999999993029775	0.940723896223149	ADCY4/SCNN1A/SCNN1B	3

Organismal Systems	Nervous system	hsa04726	Serotonergic synapse	4/350	115/8846	0.672943953881439	0.999999993029775	0.940723896223149	ALOX15B/CYP2C18/PLA2G4B/RAPGEF3	4

Organismal Systems	Endocrine system	hsa04928	Parathyroid hormone synthesis, secretion and action	4/350	115/8846	0.672943953881439	0.999999993029775	0.940723896223149	ADCY4/NACA/PDE4C/WNK4	4

Organismal Systems	Circulatory system	hsa04260	Cardiac muscle contraction	3/350	87/8846	0.675188595773342	0.999999993029775	0.940723896223149	TPM1/TPM2/UQCRB	3

Environmental Information Processing	Signal transduction	hsa04024	cAMP signaling pathway	8/350	226/8846	0.676886628335117	0.999999993029775	0.940723896223149	ADCY4/AFDN/EDN3/FXYD1/PDE4C/PIK3R2/RAP1B/RAPGEF3	8

Organismal Systems	Excretory system	hsa04966	Collecting duct acid secretion	1/350	28/8846	0.677651396132526	0.999999993029775	0.940723896223149	ATP6V0C	1

Human Diseases	Cancer: specific types	hsa05213	Endometrial cancer	2/350	59/8846	0.684074466634592	0.999999993029775	0.940723896223149	ILK/PIK3R2	2

Organismal Systems	Nervous system	hsa04727	GABAergic synapse	3/350	89/8846	0.690105674128509	0.999999993029775	0.940723896223149	ADCY4/GABARAP/SLC6A1	3

Environmental Information Processing	Signal transduction	hsa04370	VEGF signaling pathway	2/350	60/8846	0.692982374621063	0.999999993029775	0.940723896223149	PIK3R2/PLA2G4B	2

Organismal Systems	Aging	hsa04211	Longevity regulating pathway	3/350	90/8846	0.697369444810451	0.999999993029775	0.940723896223149	ADCY4/EIF4E/PIK3R2	3

Organismal Systems	Digestive system	hsa04976	Bile secretion	3/350	90/8846	0.697369444810451	0.999999993029775	0.940723896223149	ADCY4/UGT1A6/UGT1A7	3

Human Diseases	Infectious disease: viral	hsa05169	Epstein-Barr virus infection	7/350	203/8846	0.698327419273957	0.999999993029775	0.940723896223149	BLNK/IKBKB/IKBKG/IRF9/PIK3R2/PSMC1/SEM1	7

Metabolism	Carbohydrate metabolism	hsa00020	Citrate cycle (TCA cycle)	1/350	30/8846	0.702733980777486	0.999999993029775	0.940723896223149	SDHC	1

Human Diseases	Substance dependence	hsa05032	Morphine addiction	3/350	91/8846	0.70450389584896	0.999999993029775	0.940723896223149	ADCY4/PDE2A/PDE4C	3

Organismal Systems	Immune system	hsa04658	Th1 and Th2 cell differentiation	3/350	92/8846	0.711509546878795	0.999999993029775	0.940723896223149	IKBKB/IKBKG/LAT	3

Metabolism	Carbohydrate metabolism	hsa00630	Glyoxylate and dicarboxylate metabolism	1/350	31/8846	0.714535603591447	0.999999993029775	0.940723896223149	AMT	1

Human Diseases	Cancer: specific types	hsa05222	Small cell lung cancer	3/350	93/8846	0.718387010838279	0.999999993029775	0.940723896223149	IKBKB/IKBKG/PIK3R2	3

Metabolism	Carbohydrate metabolism	hsa00052	Galactose metabolism	1/350	32/8846	0.725869981214022	0.999999993029775	0.940723896223149	GALE	1

Metabolism	Carbohydrate metabolism	hsa00640	Propanoate metabolism	1/350	32/8846	0.725869981214022	0.999999993029775	0.940723896223149	BCKDHA	1

Cellular Processes	Cell growth and death	hsa04215	Apoptosis - multiple species	1/350	32/8846	0.725869981214022	0.999999993029775	0.940723896223149	DIABLO	1

Genetic Information Processing	Information processing in viruses	hsa03250	Viral life cycle - HIV-1	2/350	64/8846	0.726560864357825	0.999999993029775	0.940723896223149	CHMP4A/ELL3	2

Organismal Systems	Immune system	hsa04657	IL-17 signaling pathway	3/350	95/8846	0.731760263275971	0.999999993029775	0.940723896223149	IKBKB/IKBKG/S100A8	3

Organismal Systems	Endocrine system	hsa04921	Oxytocin signaling pathway	5/350	154/8846	0.735063158017951	0.999999993029775	0.940723896223149	ADCY4/MYL6/NFATC4/PLA2G4B/PPP1R12C	5

Genetic Information Processing	Translation	hsa00970	Aminoacyl-tRNA biosynthesis	2/350	66/8846	0.742155470078239	0.999999993029775	0.940723896223149	CARS2/SARS2	2

Human Diseases	Infectious disease: viral	hsa05170	Human immunodeficiency virus 1 infection	7/350	213/8846	0.744556045524181	0.999999993029775	0.940723896223149	AP1G2/IKBKB/IKBKG/NFATC4/PAK6/PIK3R2/SKP1	7

Organismal Systems	Nervous system	hsa04720	Long-term potentiation	2/350	67/8846	0.749664140959881	0.999999993029775	0.940723896223149	RAP1B/RAPGEF3	2

Environmental Information Processing	Signal transduction	hsa04150	mTOR signaling pathway	5/350	158/8846	0.755226329950196	0.999999993029775	0.940723896223149	CASTOR1/EIF4E/IKBKB/PIK3R2/STRADA	5

Organismal Systems	Sensory system	hsa04750	Inflammatory mediator regulation of TRP channels	3/350	99/8846	0.757004609687614	0.999999993029775	0.940723896223149	ADCY4/PIK3R2/PLA2G4B	3

Cellular Processes	Cell growth and death	hsa04217	Necroptosis	5/350	159/8846	0.760084519968947	0.999999993029775	0.940723896223149	CFLAR/CHMP4A/IRF9/PLA2G4B/RIPK3	5

Organismal Systems	Endocrine system	hsa04924	Renin secretion	2/350	69/8846	0.764119427490272	0.999999993029775	0.940723896223149	CLCA4/EDN3	2

Organismal Systems	Immune system	hsa04621	NOD-like receptor signaling pathway	6/350	189/8846	0.764576778829729	0.999999993029775	0.940723896223149	GABARAP/IKBKB/IKBKG/IRF9/NAIP/RIPK3	6

Genetic Information Processing	Transcription	hsa03040	Spliceosome	7/350	218/8846	0.76571660784534	0.999999993029775	0.940723896223149	CCDC12/DDX39B/ISY1-RAB43/PRPF40B/SRSF5/U2AF1/U2AF1L4	7

Metabolism	Amino acid metabolism	hsa00350	Tyrosine metabolism	1/350	36/8846	0.766893275761564	0.999999993029775	0.940723896223149	ADH1B	1

Metabolism	Glycan biosynthesis and metabolism	hsa00512	Mucin type O-glycan biosynthesis	1/350	36/8846	0.766893275761564	0.999999993029775	0.940723896223149	ST6GALNAC1	1

Genetic Information Processing	Replication and repair	hsa03030	DNA replication	1/350	36/8846	0.766893275761564	0.999999993029775	0.940723896223149	POLD4	1

Environmental Information Processing	Signal transduction	hsa04068	FoxO signaling pathway	4/350	133/8846	0.777746625972978	0.999999993029775	0.940723896223149	CCNG2/GABARAP/IKBKB/PIK3R2	4

Human Diseases	Infectious disease: parasitic	hsa05146	Amoebiasis	3/350	103/8846	0.780302528433372	0.999999993029775	0.940723896223149	PIK3R2/SERPINB13/SERPINB3	3

Organismal Systems	Immune system	hsa04650	Natural killer cell mediated cytotoxicity	4/350	135/8846	0.787568545455318	0.999999993029775	0.940723896223149	ICAM2/LAT/PIK3R2/RAET1E	4

Human Diseases	Infectious disease: viral	hsa05167	Kaposi sarcoma-associated herpesvirus infection	6/350	196/8846	0.793890356589891	0.999999993029775	0.940723896223149	IKBKB/IKBKG/IRF9/NFATC4/PIK3C3/PIK3R2	6

Human Diseases	Infectious disease: viral	hsa05163	Human cytomegalovirus infection	7/350	226/8846	0.796882702887519	0.999999993029775	0.940723896223149	ADCY4/CXCL12/CXCR2/IKBKB/IKBKG/NFATC4/PIK3R2	7

Genetic Information Processing	Translation	hsa03008	Ribosome biogenesis in eukaryotes	5/350	168/8846	0.800585413439556	0.999999993029775	0.940723896223149	POP4/RNA5-8SN1/RNA5-8SN2/RNA5-8SN3/SNORD3A	5

Human Diseases	Infectious disease: bacterial	hsa05135	Yersinia infection	4/350	138/8846	0.801650143799343	0.999999993029775	0.940723896223149	IKBKB/IKBKG/LAT/PIK3R2	4

Metabolism	Carbohydrate metabolism	hsa00500	Starch and sucrose metabolism	1/350	40/8846	0.801792364750432	0.999999993029775	0.940723896223149	AMY2B	1

Cellular Processes	Cell growth and death	hsa04115	p53 signaling pathway	2/350	75/8846	0.803217500881142	0.999999993029775	0.940723896223149	CCNG2/TP53I3	2

Organismal Systems	Endocrine system	hsa04918	Thyroid hormone synthesis	2/350	75/8846	0.803217500881142	0.999999993029775	0.940723896223149	ADCY4/GPX3	2

Human Diseases	Infectious disease: viral	hsa05162	Measles	4/350	139/8846	0.80617342202935	0.999999993029775	0.940723896223149	IKBKB/IKBKG/IRF9/PIK3R2	4

Genetic Information Processing	Folding, sorting and degradation	hsa04141	Protein processing in endoplasmic reticulum	5/350	170/8846	0.808815908853592	0.999999993029775	0.940723896223149	CRYAB/SEC31B/SKP1/TXNDC5/XBP1	5

Environmental Information Processing	Signal transduction	hsa04371	Apelin signaling pathway	4/350	140/8846	0.810612766784138	0.999999993029775	0.940723896223149	ADCY4/BORCS8-MEF2B/PIK3C3/SMAD2	4

Metabolism	Glycan biosynthesis and metabolism	hsa00513	Various types of N-glycan biosynthesis	1/350	42/8846	0.817235886199723	0.999999993029775	0.940723896223149	ALG9	1

Human Diseases	Infectious disease: bacterial	hsa05133	Pertussis	2/350	78/8846	0.820527012952899	0.999999993029775	0.940723896223149	C4A/C4B	2

Organismal Systems	Endocrine system	hsa04914	Progesterone-mediated oocyte maturation	3/350	111/8846	0.821387824788703	0.999999993029775	0.940723896223149	ADCY4/ANAPC15/PIK3R2	3

Human Diseases	Cancer: overview	hsa05205	Proteoglycans in cancer	6/350	204/8846	0.823882687781006	0.999999993029775	0.940723896223149	DCN/PDCD4/PIK3R2/PPP1R12C/SMAD2/TIMP3	6

Human Diseases	Endocrine and metabolic disease	hsa04940	Type I diabetes mellitus	1/350	43/8846	0.824501610851029	0.999999993029775	0.940723896223149	ICA1	1

Organismal Systems	Digestive system	hsa04975	Fat digestion and absorption	1/350	43/8846	0.824501610851029	0.999999993029775	0.940723896223149	PLA2G2A	1

Organismal Systems	Nervous system	hsa04721	Synaptic vesicle cycle	2/350	79/8846	0.825986946188254	0.999999993029775	0.940723896223149	ATP6V0C/SLC6A1	2

Cellular Processes	Cellular community - eukaryotes	hsa04550	Signaling pathways regulating pluripotency of stem cells	4/350	144/8846	0.827548115827041	0.999999993029775	0.940723896223149	COMMD3-BMI1/MEIS1/PIK3R2/SMAD2	4

Human Diseases	Drug resistance: antineoplastic	hsa01521	EGFR tyrosine kinase inhibitor resistance	2/350	80/8846	0.831297371727453	0.999999993029775	0.940723896223149	EIF4E/PIK3R2	2

Genetic Information Processing	Chromosome	hsa03082	ATP-dependent chromatin remodeling	3/350	117/8846	0.84774948556316	0.999999993029775	0.940723896223149	INO80B/INO80C/SMARCE1	3

Organismal Systems	Nervous system	hsa04722	Neurotrophin signaling pathway	3/350	120/8846	0.85961874854121	0.999999993029775	0.940723896223149	IKBKB/PIK3R2/RAP1B	3

Environmental Information Processing	Signal transduction	hsa04012	ErbB signaling pathway	2/350	86/8846	0.860200114136181	0.999999993029775	0.940723896223149	PAK6/PIK3R2	2

Human Diseases	Cardiovascular disease	hsa05412	Arrhythmogenic right ventricular cardiomyopathy	2/350	86/8846	0.860200114136181	0.999999993029775	0.940723896223149	DES/ITGA7	2

Organismal Systems	Immune system	hsa04672	Intestinal immune network for IgA production	1/350	49/8846	0.862430080452874	0.999999993029775	0.940723896223149	CXCL12	1

Organismal Systems	Circulatory system	hsa04261	Adrenergic signaling in cardiomyocytes	4/350	154/8846	0.864457606162087	0.999999993029775	0.940723896223149	ADCY4/RAPGEF3/TPM1/TPM2	4

Human Diseases	Cancer: specific types	hsa05210	Colorectal cancer	2/350	87/8846	0.864553326332953	0.999999993029775	0.940723896223149	PIK3R2/SMAD2	2

Environmental Information Processing	Signal transduction	hsa04152	AMPK signaling pathway	3/350	122/8846	0.867075127177676	0.999999993029775	0.940723896223149	CPT1B/PIK3R2/STRADA	3

Environmental Information Processing	Signaling molecules and interaction	hsa04512	ECM-receptor interaction	2/350	89/8846	0.872889696719777	0.999999993029775	0.940723896223149	ITGA7/TNXB	2

Organismal Systems	Digestive system	hsa04979	Cholesterol metabolism	1/350	51/8846	0.873159705441339	0.999999993029775	0.940723896223149	CIDEB	1

Human Diseases	Infectious disease: bacterial	hsa05110	Vibrio cholerae infection	1/350	51/8846	0.873159705441339	0.999999993029775	0.940723896223149	ATP6V0C	1

Environmental Information Processing	Signaling molecules and interaction	hsa04514	Cell adhesion molecules	4/350	157/8846	0.874126327242363	0.999999993029775	0.940723896223149	CLDN5/CLDN7/ICAM2/ICAM3	4

Metabolism	Glycan biosynthesis and metabolism	hsa00510	N-Glycan biosynthesis	1/350	53/8846	0.883054688405767	0.999999993029775	0.940723896223149	ALG9	1

Organismal Systems	Excretory system	hsa04961	Endocrine and other factor-regulated calcium reabsorption	1/350	53/8846	0.883054688405767	0.999999993029775	0.940723896223149	RAB11A	1

Human Diseases	Cancer: overview	hsa05202	Transcriptional misregulation in cancer	5/350	193/8846	0.885210658134626	0.999999993029775	0.940723896223149	COMMD3-BMI1/HPGD/MEIS1/NUPR1/TMPRSS2	5

Metabolism	Lipid metabolism	hsa00600	Sphingolipid metabolism	1/350	54/8846	0.887709625180245	0.999999993029775	0.940723896223149	DEGS2	1

Organismal Systems	Endocrine system	hsa04912	GnRH signaling pathway	2/350	93/8846	0.888164466630839	0.999999993029775	0.940723896223149	ADCY4/PLA2G4B	2

Human Diseases	Infectious disease: viral	hsa05161	Hepatitis B	4/350	163/8846	0.891692575443491	0.999999993029775	0.940723896223149	IKBKB/IKBKG/NFATC4/PIK3R2	4

Human Diseases	Immune disease	hsa05323	Rheumatoid arthritis	2/350	94/8846	0.891708542242024	0.999999993029775	0.940723896223149	ATP6V0C/CXCL12	2

Organismal Systems	Endocrine system	hsa04926	Relaxin signaling pathway	3/350	130/8846	0.893524169771454	0.999999993029775	0.940723896223149	ADCY4/PIK3R2/SMAD2	3

Cellular Processes	Transport and catabolism	hsa04142	Lysosome	3/350	132/8846	0.89935464544327	0.999999993029775	0.940723896223149	AP1G2/ATP6V0C/CD68	3

Environmental Information Processing	Signal transduction	hsa04022	cGMP-PKG signaling pathway	4/350	166/8846	0.899644946948114	0.999999993029775	0.940723896223149	ADCY4/BORCS8-MEF2B/NFATC4/PDE2A	4

Organismal Systems	Digestive system	hsa04970	Salivary secretion	2/350	97/8846	0.901725465147151	0.999999993029775	0.940723896223149	ADCY4/AMY2B	2

Environmental Information Processing	Signal transduction	hsa04630	JAK-STAT signaling pathway	4/350	168/8846	0.904657275002003	0.999999993029775	0.940723896223149	FHL1/IL11RA/IRF9/PIK3R2	4

Organismal Systems	Endocrine system	hsa04925	Aldosterone synthesis and secretion	2/350	98/8846	0.904868103246404	0.999999993029775	0.940723896223149	ADCY4/PDE2A	2

Human Diseases	Drug resistance: antineoplastic	hsa01522	Endocrine resistance	2/350	99/8846	0.907916835072358	0.999999993029775	0.940723896223149	ADCY4/PIK3R2	2

Organismal Systems	Immune system	hsa04640	Hematopoietic cell lineage	2/350	99/8846	0.907916835072358	0.999999993029775	0.940723896223149	FLT3LG/IL11RA	2

Organismal Systems	Nervous system	hsa04730	Long-term depression	1/350	60/8846	0.912005037696705	0.999999993029775	0.940723896223149	PLA2G4B	1

Human Diseases	Endocrine and metabolic disease	hsa04933	AGE-RAGE signaling pathway in diabetic complications	2/350	101/8846	0.913742467345527	0.999999993029775	0.940723896223149	PIK3R2/SMAD2	2

Human Diseases	Infectious disease: viral	hsa05165	Human papillomavirus infection	9/350	333/8846	0.916505573547533	0.999999993029775	0.940723896223149	ATP6V0C/IKBKB/IKBKG/IRF9/ITGA7/LLGL2/PIK3R2/PSMC1/TNXB	9

Cellular Processes	Cell growth and death	hsa04114	Oocyte meiosis	3/350	139/8846	0.917566432922528	0.999999993029775	0.940723896223149	ADCY4/ANAPC15/SKP1	3

Human Diseases	Immune disease	hsa05322	Systemic lupus erythematosus	3/350	139/8846	0.917566432922528	0.999999993029775	0.940723896223149	C4A/C4B/C7	3

Genetic Information Processing	Translation	hsa03015	mRNA surveillance pathway	2/350	103/8846	0.919221647903307	0.999999993029775	0.940723896223149	DDX39B/PCF11	2

Genetic Information Processing	Folding, sorting and degradation	hsa04120	Ubiquitin mediated proteolysis	3/350	142/8846	0.924411647714238	0.999999993029775	0.940723896223149	ANAPC15/CUL3/SKP1	3

Organismal Systems	Endocrine system	hsa04927	Cortisol synthesis and secretion	1/350	65/8846	0.928193434485332	0.999999993029775	0.940723896223149	ADCY4	1

Organismal Systems	Endocrine system	hsa04929	GnRH secretion	1/350	65/8846	0.928193434485332	0.999999993029775	0.940723896223149	PIK3R2	1

Human Diseases	Immune disease	hsa05321	Inflammatory bowel disease	1/350	65/8846	0.928193434485332	0.999999993029775	0.940723896223149	SMAD2	1

Genetic Information Processing	Translation	hsa03013	Nucleocytoplasmic transport	2/350	108/8846	0.931523400981453	0.999999993029775	0.940723896223149	DDX39B/IPO4	2

Human Diseases	Cardiovascular disease	hsa05417	Lipid and atherosclerosis	5/350	216/8846	0.933889727652496	0.999999993029775	0.940723896223149	IKBKB/IKBKG/PIK3R2/RAP1B/XBP1	5

Human Diseases	Infectious disease: parasitic	hsa05145	Toxoplasmosis	2/350	111/8846	0.938032354625732	0.999999993029775	0.940723896223149	IKBKB/IKBKG	2

Cellular Processes	Transport and catabolism	hsa04144	Endocytosis	6/350	252/8846	0.938225542653575	0.999999993029775	0.940723896223149	CHMP4A/CXCR2/CYTH2/RAB11A/SH3GLB2/SMAD2	6

Organismal Systems	Endocrine system	hsa04917	Prolactin signaling pathway	1/350	71/8846	0.943747216515854	0.999999993029775	0.940723896223149	PIK3R2	1

Human Diseases	Cancer: overview	hsa05230	Central carbon metabolism in cancer	1/350	71/8846	0.943747216515854	0.999999993029775	0.940723896223149	PIK3R2	1

Organismal Systems	Nervous system	hsa04725	Cholinergic synapse	2/350	115/8846	0.945803731282279	0.999999993029775	0.940723896223149	ADCY4/PIK3R2	2

Metabolism	Global and overview maps	hsa01200	Carbon metabolism	2/350	116/8846	0.947596707712402	0.999999993029775	0.940723896223149	AMT/SDHC	2

Organismal Systems	Nervous system	hsa04724	Glutamatergic synapse	2/350	116/8846	0.947596707712402	0.999999993029775	0.940723896223149	ADCY4/PLA2G4B	2

Human Diseases	Cancer: specific types	hsa05218	Melanoma	1/350	73/8846	0.948145371230935	0.999999993029775	0.940723896223149	PIK3R2	1

Human Diseases	Cancer: specific types	hsa05223	Non-small cell lung cancer	1/350	73/8846	0.948145371230935	0.999999993029775	0.940723896223149	PIK3R2	1

Cellular Processes	Cell growth and death	hsa04218	Cellular senescence	3/350	157/8846	0.951458718160559	0.999999993029775	0.940723896223149	NFATC4/PIK3R2/SMAD2	3

Metabolism	Global and overview maps	hsa01230	Biosynthesis of amino acids	1/350	75/8846	0.952200559310711	0.999999993029775	0.940723896223149	ACY1	1

Cellular Processes	Cell growth and death	hsa04110	Cell cycle	3/350	158/8846	0.952895700340563	0.999999993029775	0.940723896223149	ANAPC15/SKP1/SMAD2	3

Organismal Systems	Digestive system	hsa04971	Gastric acid secretion	1/350	76/8846	0.9541079591786	0.999999993029775	0.940723896223149	ADCY4	1

Human Diseases	Cancer: specific types	hsa05214	Glioma	1/350	76/8846	0.9541079591786	0.999999993029775	0.940723896223149	PIK3R2	1

Environmental Information Processing	Signal transduction	hsa04071	Sphingolipid signaling pathway	2/350	122/8846	0.957224233987739	0.999999993029775	0.940723896223149	DEGS2/PIK3R2	2

Organismal Systems	Endocrine system	hsa04935	Growth hormone synthesis, secretion and action	2/350	122/8846	0.957224233987739	0.999999993029775	0.940723896223149	ADCY4/PIK3R2	2

Organismal Systems	Immune system	hsa04612	Antigen processing and presentation	1/350	80/8846	0.961008045084163	0.999999993029775	0.940723896223149	IFI30	1

Human Diseases	Cancer: overview	hsa05206	MicroRNAs in cancer	7/350	312/8846	0.967191152403174	0.999999993029775	0.940723896223149	COMMD3-BMI1/IKBKB/PDCD4/PIK3R2/TIMP3/TNXB/TPM1	7

Organismal Systems	Endocrine system	hsa04911	Insulin secretion	1/350	86/8846	0.969466987613913	0.999999993029775	0.940723896223149	ADCY4	1

Human Diseases	Infectious disease: bacterial	hsa05152	Tuberculosis	3/350	180/8846	0.976036214784077	0.999999993029775	0.940723896223149	ATP6V0C/KSR1/PIK3C3	3

Cellular Processes	Cellular community - eukaryotes	hsa04540	Gap junction	1/350	92/8846	0.976094929454665	0.999999993029775	0.940723896223149	ADCY4	1

Environmental Information Processing	Signal transduction	hsa04151	PI3K-Akt signaling pathway	8/350	362/8846	0.977760024332649	0.999999993029775	0.940723896223149	EIF4E/FLT3LG/IKBKB/IKBKG/ITGA7/MTCP1/PIK3R2/TNXB	8

Organismal Systems	Environmental adaptation	hsa04713	Circadian entrainment	1/350	97/8846	0.980507530277121	0.999999993029775	0.940723896223149	ADCY4	1

Environmental Information Processing	Signaling molecules and interaction	hsa04080	Neuroactive ligand-receptor interaction	8/350	368/8846	0.980715666411185	0.999999993029775	0.940723896223149	EDN3/LYNX1/NMU/P2RX1/PRSS1/PRSS2/PRSS3/SLURP1	8

Environmental Information Processing	Signal transduction	hsa04010	MAPK signaling pathway	6/350	300/8846	0.981277467776204	0.999999993029775	0.940723896223149	FLT3LG/IKBKB/IKBKG/PLA2G4B/RAP1B/RASGRP2	6

Organismal Systems	Endocrine system	hsa04916	Melanogenesis	1/350	101/8846	0.983444926526609	0.999999993029775	0.940723896223149	ADCY4	1

Organismal Systems	Immune system	hsa04613	Neutrophil extracellular trap formation	3/350	192/8846	0.983601203512207	0.999999993029775	0.940723896223149	HDAC10/HDAC6/PIK3R2	3

Human Diseases	Cancer: specific types	hsa05226	Gastric cancer	2/350	150/8846	0.983782675846769	0.999999993029775	0.940723896223149	PIK3R2/SMAD2	2

Human Diseases	Endocrine and metabolic disease	hsa04934	Cushing syndrome	2/350	155/8846	0.986407717915587	0.999999993029775	0.940723896223149	ADCY4/RAP1B	2

Organismal Systems	Endocrine system	hsa04922	Glucagon signaling pathway	1/350	107/8846	0.987044166385436	0.999999993029775	0.940723896223149	CPT1B	1

Cellular Processes	Transport and catabolism	hsa04145	Phagosome	2/350	157/8846	0.987337850810147	0.999999993029775	0.940723896223149	ATP6V0C/PIK3C3	2

NA	NA	hsa04148	Efferocytosis	2/350	157/8846	0.987337850810147	0.999999993029775	0.940723896223149	CPT1B/NFATC4	2

Environmental Information Processing	Signal transduction	hsa04390	Hippo signaling pathway	2/350	157/8846	0.987337850810147	0.999999993029775	0.940723896223149	LLGL2/SMAD2	2

Environmental Information Processing	Signal transduction	hsa04020	Calcium signaling pathway	4/350	254/8846	0.991706820003209	0.999999993029775	0.940723896223149	ADCY4/MST1/NFATC4/P2RX1	4

Organismal Systems	Endocrine system	hsa04919	Thyroid hormone signaling pathway	1/350	122/8846	0.992985863135532	0.999999993029775	0.940723896223149	PIK3R2	1

Environmental Information Processing	Signal transduction	hsa04310	Wnt signaling pathway	2/350	174/8846	0.993105953111751	0.999999993029775	0.940723896223149	NFATC4/SKP1	2

Human Diseases	Substance dependence	hsa05034	Alcoholism	2/350	188/8846	0.995849004846887	0.999999993029775	0.940723896223149	HDAC10/HDAC6	2

Human Diseases	Cancer: specific types	hsa05224	Breast cancer	1/350	148/8846	0.997584836926867	0.999999993029775	0.940723896223149	PIK3R2	1

Organismal Systems	Sensory system	hsa04740	Olfactory transduction	1/350	453/8846	0.999999993029775	0.999999993029775	0.940723896223149	PDE2A	1
