# Supplementary material for: Gene Expression Profiling and Clinical Validation Reveal CEBPB for Early Diagnosis of Esophageal Squamous Cell Carcinoma
Source: Cancer Rep (Hoboken). 2026 Nov 8;9(11):e70753. doi: 10.1002/cnr2.70753 (PMC13745642; doi:10.1002/cnr2.70753)
Supplement: Supplementary file 11 [file CNR2-9-e70753-s004.doc]

Doc. S5. GO enrichment analysis of the downregulated DEGs.

ONTOLOGY	ID	Description	GeneRatio	BgRatio	pvalue	p.adjust	qvalue	geneID	Count

BP	GO:0002181	cytoplasmic translation	29/695	156/18870	5.03036882572941e-13	1.99504427628428e-09	1.98090629232144e-09	EIF3C/EIF3CL/EIF3F/EIF4A1/RBM4/RPL13/RPL14/RPL15/RPL17/RPL18/RPL22/RPL23/RPL26/RPL27A/RPL30/RPL31/RPL32/RPL34/RPL36A/RPL37/RPL37A/RPL39/RPS15A/RPS19/RPS23/RPS24/RPS29/RPS3/RPS9	29

BP	GO:0015986	proton motive force-driven ATP synthesis	16/695	76/18870	1.33320137714779e-08	2.64373833088407e-05	2.62500334311047e-05	ATP5MC3/ATP5PD/ATP5PO/ATP6V0C/NDUFA10/NDUFA11/NDUFA13/NDUFA7/NDUFA9/NDUFB1/NDUFB2/NDUFB8/NDUFC2/NDUFS7/NDUFV2/SDHC	16

BP	GO:0042776	proton motive force-driven mitochondrial ATP synthesis	14/695	67/18870	1.1902887836018e-07	0.000114241461798952	0.000113431883799103	ATP5PD/ATP5PO/NDUFA10/NDUFA11/NDUFA13/NDUFA7/NDUFA9/NDUFB1/NDUFB2/NDUFB8/NDUFC2/NDUFS7/NDUFV2/SDHC	14

BP	GO:0009206	purine ribonucleoside triphosphate biosynthetic process	18/695	113/18870	1.59581284094098e-07	0.000114241461798952	0.000113431883799103	ATP5MC3/ATP5PD/ATP5PO/ATP6V0C/NDUFA10/NDUFA11/NDUFA13/NDUFA7/NDUFA9/NDUFB1/NDUFB2/NDUFB8/NDUFC2/NDUFS7/NDUFV2/NME2/PINK1/SDHC	18

BP	GO:0006754	ATP biosynthetic process	17/695	102/18870	1.79222466027245e-07	0.000114241461798952	0.000113431883799103	ATP5MC3/ATP5PD/ATP5PO/ATP6V0C/NDUFA10/NDUFA11/NDUFA13/NDUFA7/NDUFA9/NDUFB1/NDUFB2/NDUFB8/NDUFC2/NDUFS7/NDUFV2/PINK1/SDHC	17

BP	GO:0009145	purine nucleoside triphosphate biosynthetic process	18/695	114/18870	1.83095853416599e-07	0.000114241461798952	0.000113431883799103	ATP5MC3/ATP5PD/ATP5PO/ATP6V0C/NDUFA10/NDUFA11/NDUFA13/NDUFA7/NDUFA9/NDUFB1/NDUFB2/NDUFB8/NDUFC2/NDUFS7/NDUFV2/NME2/PINK1/SDHC	18

BP	GO:0009142	nucleoside triphosphate biosynthetic process	19/695	127/18870	2.01636468127247e-07	0.000114241461798952	0.000113431883799103	AK1/ATP5MC3/ATP5PD/ATP5PO/ATP6V0C/NDUFA10/NDUFA11/NDUFA13/NDUFA7/NDUFA9/NDUFB1/NDUFB2/NDUFB8/NDUFC2/NDUFS7/NDUFV2/NME2/PINK1/SDHC	19

BP	GO:0009201	ribonucleoside triphosphate biosynthetic process	18/695	119/18870	3.55491793960798e-07	0.000176235056856066	0.000174986158053598	ATP5MC3/ATP5PD/ATP5PO/ATP6V0C/NDUFA10/NDUFA11/NDUFA13/NDUFA7/NDUFA9/NDUFB1/NDUFB2/NDUFB8/NDUFC2/NDUFS7/NDUFV2/NME2/PINK1/SDHC	18

BP	GO:0046034	ATP metabolic process	18/695	137/18870	2.91282023939918e-06	0.00128358278549524	0.0012744866100108	AK1/ATP5MC3/ATP5PD/ATP5PO/ATP6V0C/NDUFA10/NDUFA11/NDUFA13/NDUFA7/NDUFA9/NDUFB1/NDUFB2/NDUFB8/NDUFC2/NDUFS7/NDUFV2/PINK1/SDHC	18

BP	GO:0009205	purine ribonucleoside triphosphate metabolic process	19/695	161/18870	7.75260907116219e-06	0.00307468475762292	0.00305289584581239	AK1/ATP5MC3/ATP5PD/ATP5PO/ATP6V0C/NDUFA10/NDUFA11/NDUFA13/NDUFA7/NDUFA9/NDUFB1/NDUFB2/NDUFB8/NDUFC2/NDUFS7/NDUFV2/NME2/PINK1/SDHC	19

BP	GO:0009144	purine nucleoside triphosphate metabolic process	19/695	167/18870	1.31775188042533e-05	0.00437985175602032	0.00434881371108953	AK1/ATP5MC3/ATP5PD/ATP5PO/ATP6V0C/NDUFA10/NDUFA11/NDUFA13/NDUFA7/NDUFA9/NDUFB1/NDUFB2/NDUFB8/NDUFC2/NDUFS7/NDUFV2/NME2/PINK1/SDHC	19

BP	GO:0006120	mitochondrial electron transport, NADH to ubiquinone	10/695	51/18870	1.40159527986621e-05	0.00437985175602032	0.00434881371108953	NDUFA10/NDUFA7/NDUFA9/NDUFB1/NDUFB2/NDUFB8/NDUFC2/NDUFS7/NDUFV2/PINK1	10

BP	GO:0009199	ribonucleoside triphosphate metabolic process	19/695	168/18870	1.43565488724821e-05	0.00437985175602032	0.00434881371108953	AK1/ATP5MC3/ATP5PD/ATP5PO/ATP6V0C/NDUFA10/NDUFA11/NDUFA13/NDUFA7/NDUFA9/NDUFB1/NDUFB2/NDUFB8/NDUFC2/NDUFS7/NDUFV2/NME2/PINK1/SDHC	19

BP	GO:0006119	oxidative phosphorylation	17/695	148/18870	3.28032483032593e-05	0.00929269162648045	0.00922683848890925	ATP5PD/ATP5PO/NDUFA10/NDUFA11/NDUFA13/NDUFA7/NDUFA9/NDUFB1/NDUFB2/NDUFB8/NDUFC2/NDUFS7/NDUFV2/NUPR1/PINK1/SDHC/UQCRB	17

BP	GO:0009260	ribonucleotide biosynthetic process	22/695	228/18870	3.86399882580023e-05	0.0102164128954158	0.0101440137595219	ADCY4/AK1/ATP5MC3/ATP5PD/ATP5PO/ATP6V0C/NDUFA10/NDUFA11/NDUFA13/NDUFA7/NDUFA9/NDUFB1/NDUFB2/NDUFB8/NDUFC2/NDUFS7/NDUFV2/NME2/PDK4/PINK1/SDHC/UPP1	22

BP	GO:0009152	purine ribonucleotide biosynthetic process	21/695	213/18870	4.19905237731234e-05	0.010408401080263	0.0103346414102141	ADCY4/AK1/ATP5MC3/ATP5PD/ATP5PO/ATP6V0C/NDUFA10/NDUFA11/NDUFA13/NDUFA7/NDUFA9/NDUFB1/NDUFB2/NDUFB8/NDUFC2/NDUFS7/NDUFV2/NME2/PDK4/PINK1/SDHC	21

BP	GO:0009141	nucleoside triphosphate metabolic process	19/695	184/18870	5.13793606173805e-05	0.0119865026005018	0.0119015596327938	AK1/ATP5MC3/ATP5PD/ATP5PO/ATP6V0C/NDUFA10/NDUFA11/NDUFA13/NDUFA7/NDUFA9/NDUFB1/NDUFB2/NDUFB8/NDUFC2/NDUFS7/NDUFV2/NME2/PINK1/SDHC	19

BP	GO:0046390	ribose phosphate biosynthetic process	22/695	235/18870	6.09929575435486e-05	0.0134387816454285	0.0133435470275097	ADCY4/AK1/ATP5MC3/ATP5PD/ATP5PO/ATP6V0C/NDUFA10/NDUFA11/NDUFA13/NDUFA7/NDUFA9/NDUFB1/NDUFB2/NDUFB8/NDUFC2/NDUFS7/NDUFV2/NME2/PDK4/PINK1/SDHC/UPP1	22

BP	GO:0034440	lipid oxidation	14/695	114/18870	7.71652175392511e-05	0.0161072238295089	0.0159930791586891	ABCD4/ACAA1/ACAD11/ACADVL/ACOX2/ALOX15B/AMACR/APOD/CPT1B/ECHDC2/ETFB/FMO2/HSD17B4/PDK4	14

BP	GO:0006164	purine nucleotide biosynthetic process	23/695	258/18870	8.87726403905736e-05	0.0164343435022721	0.016317880681052	ADCY4/AK1/ATP5MC3/ATP5PD/ATP5PO/ATP6V0C/GUK1/NADSYN1/NDUFA10/NDUFA11/NDUFA13/NDUFA7/NDUFA9/NDUFB1/NDUFB2/NDUFB8/NDUFC2/NDUFS7/NDUFV2/NME2/PDK4/PINK1/SDHC	23

BP	GO:0030258	lipid modification	19/695	192/18870	9.14094707965468e-05	0.0164343435022721	0.016317880681052	ABCD4/ACAA1/ACAD11/ACADVL/ACOX2/ALOX15B/AMACR/APOD/CPT1B/CYP2E1/CYP3A5/DGKA/ECHDC2/EPHX2/ETFB/FMO2/HSD17B4/MTMR3/PDK4	19

BP	GO:0009150	purine ribonucleotide metabolic process	29/695	365/18870	9.21132284836155e-05	0.0164343435022721	0.016317880681052	ADCY4/AK1/ATP5MC3/ATP5PD/ATP5PO/ATP6V0C/GUK1/HSD17B4/MVD/MVK/NDUFA10/NDUFA11/NDUFA13/NDUFA7/NDUFA9/NDUFB1/NDUFB2/NDUFB8/NDUFC2/NDUFS7/NDUFV2/NME2/PDE2A/PDE4C/PDK4/PINK1/SDHC/SULT1A3/SULT2B1	29

BP	GO:0030216	keratinocyte differentiation	18/695	177/18870	9.78804995879e-05	0.0164343435022721	0.016317880681052	ALOX15B/ANXA1/AQP3/CFLAR/CNFN/CSTA/GRHL1/IVL/KAZN/KRT4/KRT7/KRT78/NME2/SCEL/SPRR3/TGM1/TGM3/TRIM16	18

BP	GO:0009259	ribonucleotide metabolic process	30/695	385/18870	9.94513978957462e-05	0.0164343435022721	0.016317880681052	ADCY4/AK1/ATP5MC3/ATP5PD/ATP5PO/ATP6V0C/GUK1/HSD17B4/MVD/MVK/NDUFA10/NDUFA11/NDUFA13/NDUFA7/NDUFA9/NDUFB1/NDUFB2/NDUFB8/NDUFC2/NDUFS7/NDUFV2/NME2/PDE2A/PDE4C/PDK4/PINK1/SDHC/SULT1A3/SULT2B1/UPP1	30

BP	GO:0019693	ribose phosphate metabolic process	30/695	393/18870	0.000143125615943027	0.0226368255538739	0.0224764085243099	ADCY4/AK1/ATP5MC3/ATP5PD/ATP5PO/ATP6V0C/GUK1/HSD17B4/MVD/MVK/NDUFA10/NDUFA11/NDUFA13/NDUFA7/NDUFA9/NDUFB1/NDUFB2/NDUFB8/NDUFC2/NDUFS7/NDUFV2/NME2/PDE2A/PDE4C/PDK4/PINK1/SDHC/SULT1A3/SULT2B1/UPP1	30

BP	GO:0072522	purine-containing compound biosynthetic process	23/695	267/18870	0.000148400772667857	0.0226368255538739	0.0224764085243099	ADCY4/AK1/ATP5MC3/ATP5PD/ATP5PO/ATP6V0C/GUK1/NADSYN1/NDUFA10/NDUFA11/NDUFA13/NDUFA7/NDUFA9/NDUFB1/NDUFB2/NDUFB8/NDUFC2/NDUFS7/NDUFV2/NME2/PDK4/PINK1/SDHC	23

BP	GO:0019646	aerobic electron transport chain	12/695	94/18870	0.000170553261749881	0.0250523791148158	0.0248748441405966	NDUFA10/NDUFA7/NDUFA9/NDUFB1/NDUFB2/NDUFB8/NDUFC2/NDUFS7/NDUFV2/PINK1/SDHC/UQCRB	12

BP	GO:0006631	fatty acid metabolic process	30/695	401/18870	0.00020325333834394	0.0285242060607686	0.0283220678061776	ABCD4/ACAA1/ACAD11/ACADVL/ACOX2/ALOX15B/AMACR/ANXA1/CEACAM1/CPT1B/CYP2C18/CYP2E1/CYP4B1/CYP4F12/DBI/ECHDC2/ETFB/FMO2/GSTM2/HPGD/HSD17B4/LTC4S/MGLL/MGST3/PDK4/PLA2G4B/PTGDS/TNXB/TREX1/XBP1	30

BP	GO:0006690	icosanoid metabolic process	14/695	125/18870	0.000208573367564874	0.0285242060607686	0.0283220678061776	ALOX15B/ANXA1/CYP2C18/CYP2E1/CYP4F12/GGT6/HPGD/LTC4S/MGLL/MGST2/MGST3/NAIP/PLA2G4B/PTGDS	14

BP	GO:0009165	nucleotide biosynthetic process	24/695	295/18870	0.000255193594066923	0.0328969868294354	0.0326638606388295	ADCY4/AK1/ATP5MC3/ATP5PD/ATP5PO/ATP6V0C/GUK1/NADSYN1/NDUFA10/NDUFA11/NDUFA13/NDUFA7/NDUFA9/NDUFB1/NDUFB2/NDUFB8/NDUFC2/NDUFS7/NDUFV2/NME2/PDK4/PINK1/SDHC/UPP1	24

BP	GO:0097237	cellular response to toxic substance	14/695	128/18870	0.000267623546356443	0.0328969868294354	0.0326638606388295	ABCB6/GPX3/GSTM2/HBA1/HBA2/HBB/LTC4S/MGST1/MGST2/MGST3/PINK1/SELENOW/SRXN1/TXNDC17	14

BP	GO:0045333	cellular respiration	21/695	243/18870	0.000271523450460221	0.0328969868294354	0.0326638606388295	ATP5PD/ATP5PO/BLOC1S1/ETFB/NDUFA10/NDUFA11/NDUFA13/NDUFA7/NDUFA9/NDUFB1/NDUFB2/NDUFB8/NDUFC2/NDUFS7/NDUFV2/NFATC4/NUPR1/PINK1/SDHC/TREX1/UQCRB	21

BP	GO:0098869	cellular oxidant detoxification	12/695	99/18870	0.00027933557986198	0.0328969868294354	0.0326638606388295	GPX3/GSTM2/HBA1/HBA2/HBB/LTC4S/MGST1/MGST2/MGST3/SELENOW/SRXN1/TXNDC17	12

BP	GO:1901293	nucleoside phosphate biosynthetic process	24/695	297/18870	0.000282021571407162	0.0328969868294354	0.0326638606388295	ADCY4/AK1/ATP5MC3/ATP5PD/ATP5PO/ATP6V0C/GUK1/NADSYN1/NDUFA10/NDUFA11/NDUFA13/NDUFA7/NDUFA9/NDUFB1/NDUFB2/NDUFB8/NDUFC2/NDUFS7/NDUFV2/NME2/PDK4/PINK1/SDHC/UPP1	24

BP	GO:0009913	epidermal cell differentiation	21/695	245/18870	0.000303412403466053	0.0332939535330414	0.0330580142174557	ALOX15B/ANXA1/AQP3/CFLAR/CNFN/CSTA/GRHL1/IVL/KAZN/KRT4/KRT7/KRT78/NME2/SCEL/SPINK5/SPRR3/SULT2B1/TGM1/TGM3/TRIM16/TRIOBP	21

BP	GO:2000627	positive regulation of miRNA catabolic process	4/695	10/18870	0.000320775157228593	0.0332939535330414	0.0330580142174557	GAS5/NEAT1/XIST/ZSWIM8	4

BP	GO:0010257	NADH dehydrogenase complex assembly	9/695	60/18870	0.000325728589481134	0.0332939535330414	0.0330580142174557	NDUFA10/NDUFA11/NDUFA13/NDUFA9/NDUFB1/NDUFB2/NDUFB8/NDUFC2/NDUFS7	9

BP	GO:0032981	mitochondrial respiratory chain complex I assembly	9/695	60/18870	0.000325728589481134	0.0332939535330414	0.0330580142174557	NDUFA10/NDUFA11/NDUFA13/NDUFA9/NDUFB1/NDUFB2/NDUFB8/NDUFC2/NDUFS7	9

BP	GO:0120254	olefinic compound metabolic process	16/695	162/18870	0.000327398937919469	0.0332939535330414	0.0330580142174557	ACAA1/ADH1B/ALOX15B/CYP2C18/CYP2E1/CYP3A5/CYP4F12/EPHX2/GSTM2/MGLL/MGST3/PLA2G4B/PTGDS/RDH13/RDH5/SCNN1B	16

BP	GO:0008630	intrinsic apoptotic signaling pathway in response to DNA damage	12/695	102/18870	0.000369350145897153	0.0344207534665886	0.0341768290252695	CIDEB/CLU/CRIP1/CXCL12/ELL3/MUC1/NFATC4/NUPR1/RPL26/RPS27L/RPS3/TPT1	12

BP	GO:0042773	ATP synthesis coupled electron transport	12/695	102/18870	0.000369350145897153	0.0344207534665886	0.0341768290252695	NDUFA10/NDUFA7/NDUFA9/NDUFB1/NDUFB2/NDUFB8/NDUFC2/NDUFS7/NDUFV2/PINK1/SDHC/UQCRB	12

BP	GO:0042775	mitochondrial ATP synthesis coupled electron transport	12/695	102/18870	0.000369350145897153	0.0344207534665886	0.0341768290252695	NDUFA10/NDUFA7/NDUFA9/NDUFB1/NDUFB2/NDUFB8/NDUFC2/NDUFS7/NDUFV2/PINK1/SDHC/UQCRB	12

BP	GO:0009060	aerobic respiration	18/695	197/18870	0.000373195259471333	0.0344207534665886	0.0341768290252695	ATP5PD/ATP5PO/BLOC1S1/NDUFA10/NDUFA11/NDUFA13/NDUFA7/NDUFA9/NDUFB1/NDUFB2/NDUFB8/NDUFC2/NDUFS7/NDUFV2/NUPR1/PINK1/SDHC/UQCRB	18

BP	GO:1902229	regulation of intrinsic apoptotic signaling pathway in response to DNA damage	7/695	38/18870	0.000415511286147261	0.0374526763831826	0.0371872660640407	CLU/CXCL12/ELL3/MUC1/RPL26/RPS3/TPT1	7

BP	GO:1990748	cellular detoxification	13/695	119/18870	0.000443555771312276	0.0390920486449886	0.0388150208299233	ABCB6/GPX3/GSTM2/HBA1/HBA2/HBB/LTC4S/MGST1/MGST2/MGST3/SELENOW/SRXN1/TXNDC17	13

BP	GO:0006635	fatty acid beta-oxidation	10/695	76/18870	0.000458002401056824	0.039487772230247	0.0392079400996242	ABCD4/ACAA1/ACAD11/ACADVL/ACOX2/AMACR/CPT1B/ECHDC2/ETFB/HSD17B4	10

BP	GO:0051346	negative regulation of hydrolase activity	19/695	218/18870	0.000470034152194618	0.0396628818639118	0.0393818088098559	ANXA1/ANXA8/ANXA8L1/CSTA/CSTB/DNAJB6/ECM1/GCHFR/IKBKB/NAIP/NEIL1/RASA4/SEMA4D/SERPINA3/SERPINB13/SERPINB2/SERPINB3/SPINK5/TIMP3	19

BP	GO:2000625	regulation of miRNA catabolic process	4/695	11/18870	0.000489414573767608	0.0404378791575486	0.0401513140452768	GAS5/NEAT1/XIST/ZSWIM8	4

BP	GO:0006163	purine nucleotide metabolic process	32/695	463/18870	0.000514745144388094	0.0416628416865955	0.0413675958143042	ADCY4/AK1/ATP5MC3/ATP5PD/ATP5PO/ATP6V0C/FMO2/GUK1/HSD17B4/MVD/MVK/NADSYN1/NDUFA10/NDUFA11/NDUFA13/NDUFA7/NDUFA9/NDUFB1/NDUFB2/NDUFB8/NDUFC2/NDUFS7/NDUFV2/NME2/PDE2A/PDE4C/PDK4/PINK1/SDHC/SULT1A3/SULT2B1/TP53I3	32

BP	GO:0019395	fatty acid oxidation	12/695	107/18870	0.000573604501950124	0.0447678900441263	0.0444506400868106	ABCD4/ACAA1/ACAD11/ACADVL/ACOX2/AMACR/CPT1B/ECHDC2/ETFB/FMO2/HSD17B4/PDK4	12

BP	GO:0052547	regulation of peptidase activity	24/695	312/18870	0.000575683911308734	0.0447678900441263	0.0444506400868106	A2ML1/ANXA8/ANXA8L1/CIDEB/CSTA/CSTB/DIABLO/DNAJB6/ECM1/NAIP/NDUFA13/PDCD6/PINK1/PRSS22/RPS27L/RPS3/S100A8/SERPINA3/SERPINB13/SERPINB2/SERPINB3/SPINK5/TBC1D10A/TIMP3	24

BP	GO:0022904	respiratory electron transport chain	13/695	124/18870	0.000659060556360178	0.0502660416639321	0.0499098287721342	ETFB/NDUFA10/NDUFA7/NDUFA9/NDUFB1/NDUFB2/NDUFB8/NDUFC2/NDUFS7/NDUFV2/PINK1/SDHC/UQCRB	13

CC	GO:0044391	ribosomal subunit	35/737	188/19886	2.26453535407482e-15	1.18208745482706e-12	1.05837231285181e-12	MRPL23/MRPL38/MRPL46/MRPL52/MRPL53/MRPS11/MRPS18C/MRPS24/MRPS33/MTERF4/RPL13/RPL14/RPL15/RPL17/RPL18/RPL22/RPL23/RPL26/RPL27A/RPL30/RPL31/RPL32/RPL34/RPL36A/RPL37/RPL37A/RPL39/RPS15A/RPS19/RPS23/RPS24/RPS27L/RPS29/RPS3/RPS9	35

CC	GO:0005840	ribosome	38/737	246/19886	7.56479641422284e-14	1.97441186411216e-11	1.76777347784997e-11	APOD/MRPL23/MRPL38/MRPL46/MRPL52/MRPL53/MRPS11/MRPS18C/MRPS24/MRPS33/MTERF4/MTG1/NDUFA7/RPL13/RPL14/RPL15/RPL17/RPL18/RPL22/RPL23/RPL26/RPL27A/RPL30/RPL31/RPL32/RPL34/RPL36A/RPL37/RPL37A/RPL39/RPS15A/RPS19/RPS23/RPS24/RPS27L/RPS29/RPS3/RPS9	38

CC	GO:0022626	cytosolic ribosome	26/737	118/19886	1.49314397987399e-13	2.59807052498074e-11	2.32616114759316e-11	APOD/RPL13/RPL14/RPL15/RPL17/RPL18/RPL22/RPL23/RPL26/RPL27A/RPL30/RPL31/RPL32/RPL34/RPL36A/RPL37/RPL37A/RPL39/RPS15A/RPS19/RPS23/RPS24/RPS27L/RPS29/RPS3/RPS9	26

CC	GO:0022625	cytosolic large ribosomal subunit	17/737	60/19886	3.40760336732961e-11	4.44692239436514e-09	3.98151551340618e-09	RPL13/RPL14/RPL15/RPL17/RPL18/RPL22/RPL23/RPL26/RPL27A/RPL30/RPL31/RPL32/RPL34/RPL36A/RPL37/RPL37A/RPL39	17

CC	GO:0015934	large ribosomal subunit	23/737	117/19886	4.57300262963651e-11	4.77421474534052e-09	4.2745540369655e-09	MRPL23/MRPL38/MRPL46/MRPL52/MRPL53/MTERF4/RPL13/RPL14/RPL15/RPL17/RPL18/RPL22/RPL23/RPL26/RPL27A/RPL30/RPL31/RPL32/RPL34/RPL36A/RPL37/RPL37A/RPL39	23

CC	GO:0005743	mitochondrial inner membrane	44/737	497/19886	9.34093044005135e-08	8.12660948284468e-06	7.2760931848821e-06	ACAD11/ACADVL/ATP5MC3/ATP5PD/ATP5PO/CKMT1A/CKMT1B/CLU/COQ6/COX20/CYP2E1/MICOS10/MRPL23/MRPL38/MRPL46/MRPL52/MRPL53/MRPS11/MRPS18C/MRPS24/MRPS33/MTG1/NDUFA10/NDUFA11/NDUFA13/NDUFA7/NDUFA9/NDUFB1/NDUFB2/NDUFB8/NDUFC2/NDUFS7/NDUFV2/PDE2A/PINK1/PLA2G4B/PLSCR3/PRODH/RDH13/RPS3/SDHC/SLC25A27/SPNS1/UQCRB	44

CC	GO:0098798	mitochondrial protein-containing complex	30/737	300/19886	8.76811819782154e-07	6.53851099894692e-05	5.85420222531243e-05	ATP5MC3/ATP5PD/ATP5PO/BCKDHA/MICOS10/MRPL23/MRPL38/MRPL46/MRPL52/MRPL53/MRPS11/MRPS18C/MRPS24/MRPS33/MTERF4/NDUFA10/NDUFA11/NDUFA13/NDUFA7/NDUFA9/NDUFB1/NDUFB2/NDUFB8/NDUFC2/NDUFS7/NDUFV2/SDHC/TOMM5/TOMM6/UQCRB	30

CC	GO:0005747	mitochondrial respiratory chain complex I	11/737	49/19886	1.35316327760917e-06	7.06351230911985e-05	6.32425784482599e-05	NDUFA10/NDUFA11/NDUFA13/NDUFA7/NDUFA9/NDUFB1/NDUFB2/NDUFB8/NDUFC2/NDUFS7/NDUFV2	11

CC	GO:0030964	NADH dehydrogenase complex	11/737	49/19886	1.35316327760917e-06	7.06351230911985e-05	6.32425784482599e-05	NDUFA10/NDUFA11/NDUFA13/NDUFA7/NDUFA9/NDUFB1/NDUFB2/NDUFB8/NDUFC2/NDUFS7/NDUFV2	11

CC	GO:0045271	respiratory chain complex I	11/737	49/19886	1.35316327760917e-06	7.06351230911985e-05	6.32425784482599e-05	NDUFA10/NDUFA11/NDUFA13/NDUFA7/NDUFA9/NDUFB1/NDUFB2/NDUFB8/NDUFC2/NDUFS7/NDUFV2	11

CC	GO:0001533	cornified envelope	11/737	60/19886	1.09828447560328e-05	0.000521185905695377	0.000466639528390295	ANXA1/CNFN/CSTA/CYSRT1/IVL/KAZN/SCEL/SERPINB2/SPRR2A/SPRR3/TGM1	11

CC	GO:0042788	polysomal ribosome	8/737	31/19886	1.26715548872495e-05	0.000551212637595351	0.000493523716661294	RPL18/RPL30/RPL31/RPL32/RPL36A/RPL39/RPS23/RPS29	8

CC	GO:0015935	small ribosomal subunit	12/737	75/19886	1.88185576377061e-05	0.000755637468221739	0.000676553813047897	MRPS11/MRPS18C/MRPS24/MRPS33/RPS15A/RPS19/RPS23/RPS24/RPS27L/RPS29/RPS3/RPS9	12

CC	GO:0098803	respiratory chain complex	13/737	91/19886	2.98797427931796e-05	0.00111408755271713	0.00099748915790765	NDUFA10/NDUFA11/NDUFA13/NDUFA7/NDUFA9/NDUFB1/NDUFB2/NDUFB8/NDUFC2/NDUFS7/NDUFV2/SDHC/UQCRB	13

CC	GO:0005746	mitochondrial respirasome	13/737	94/19886	4.23972474262789e-05	0.00147542421043451	0.00132100897243985	NDUFA10/NDUFA11/NDUFA13/NDUFA7/NDUFA9/NDUFB1/NDUFB2/NDUFB8/NDUFC2/NDUFS7/NDUFV2/SDHC/UQCRB	13

CC	GO:0005925	focal adhesion	33/737	421/19886	4.61435388668572e-05	0.00150543295553122	0.00134787705637399	AIF1L/ALKBH6/ALOX15B/ANXA1/ATP6V0C/CAPN5/CNN1/CSRP2/EVL/FAM107A/FHL1/GAK/ILK/LIMS2/LMO7/MPRIP/PCBP2/PGM5/PIK3R2/RPL18/RPL22/RPL23/RPL30/RPL31/RPL37A/RPS19/RPS29/RPS3/RPS9/SORBS2/TNS2/TRIOBP/ZFYVE21	33

CC	GO:0030055	cell-substrate junction	33/737	431/19886	7.27579349432365e-05	0.00223409659060997	0.00200028006902768	AIF1L/ALKBH6/ALOX15B/ANXA1/ATP6V0C/CAPN5/CNN1/CSRP2/EVL/FAM107A/FHL1/GAK/ILK/LIMS2/LMO7/MPRIP/PCBP2/PGM5/PIK3R2/RPL18/RPL22/RPL23/RPL30/RPL31/RPL37A/RPS19/RPS29/RPS3/RPS9/SORBS2/TNS2/TRIOBP/ZFYVE21	33

CC	GO:1990204	oxidoreductase complex	15/737	128/19886	8.03846181298564e-05	0.00224691100902634	0.00201175335351421	BCKDHA/ETFB/NDUFA10/NDUFA11/NDUFA13/NDUFA7/NDUFA9/NDUFB1/NDUFB2/NDUFB8/NDUFC2/NDUFS7/NDUFV2/SDHC/UQCRB	15

CC	GO:0098800	inner mitochondrial membrane protein complex	17/737	158/19886	8.17841171867825e-05	0.00224691100902634	0.00201175335351421	ATP5MC3/ATP5PD/ATP5PO/MICOS10/NDUFA10/NDUFA11/NDUFA13/NDUFA7/NDUFA9/NDUFB1/NDUFB2/NDUFB8/NDUFC2/NDUFS7/NDUFV2/SDHC/UQCRB	17

CC	GO:0070469	respirasome	13/737	102/19886	0.000100365789745043	0.00251016037521462	0.00224745151561866	NDUFA10/NDUFA11/NDUFA13/NDUFA7/NDUFA9/NDUFB1/NDUFB2/NDUFB8/NDUFC2/NDUFS7/NDUFV2/SDHC/UQCRB	13

CC	GO:0000313	organellar ribosome	12/737	89/19886	0.000106567763673085	0.00251016037521462	0.00224745151561866	MRPL23/MRPL38/MRPL46/MRPL52/MRPL53/MRPS11/MRPS18C/MRPS24/MRPS33/MTERF4/MTG1/NDUFA7	12

CC	GO:0005761	mitochondrial ribosome	12/737	89/19886	0.000106567763673085	0.00251016037521462	0.00224745151561866	MRPL23/MRPL38/MRPL46/MRPL52/MRPL53/MRPS11/MRPS18C/MRPS24/MRPS33/MTERF4/MTG1/NDUFA7	12

CC	GO:0022627	cytosolic small ribosomal subunit	8/737	41/19886	0.000110600936072675	0.00251016037521462	0.00224745151561866	RPS15A/RPS19/RPS23/RPS24/RPS27L/RPS29/RPS3/RPS9	8

CC	GO:0005844	polysome	10/737	67/19886	0.000168762024116412	0.00367057402453197	0.00328641836437224	PSMA6/RPL18/RPL30/RPL31/RPL32/RPL36A/RPL39/RPS23/RPS29/RPS3	10

CC	GO:0016607	nuclear speck	30/737	417/19886	0.000441150131888568	0.0092112147538333	0.00824718562351681	ADGRD1/AFDN/ARL6IP4/ATOH8/CCNL2/CLK3/DDX39B/EIF4E/ELL3/FAM107A/FAM193B/GTF2H2C/GTF2H4/HIF3A/HSF4/HSPB6/KAZN/NDUFB1/NFATC4/PRPF40B/RBM4/RTEL1/SARNP/SDCBP2/SERPINB13/SIRT7/SRSF5/STK19/U2AF1/U2AF1L4	30

CC	GO:0005759	mitochondrial matrix	32/737	487/19886	0.00133094283450567	0.0267212369081523	0.0239246404259318	ACADVL/ALDH2/ALDH7A1/AMT/BCKDHA/BLOC1S1/CARS2/ETFB/METTL17/MMAB/MRPL23/MRPL38/MRPL46/MRPL52/MRPL53/MRPS11/MRPS18C/MRPS24/MRPS33/MTERF4/MTG1/NDUFA10/NDUFA7/NDUFA9/NDUFB8/NDUFS7/PDE2A/PDK4/PHYKPL/PRODH/RPS3/SARS2	32

CC	GO:0031089	platelet dense granule lumen	4/737	14/19886	0.00139131980282731	0.026898849521328	0.0240836644232096	CLEC3B/ECM1/SELENOP/TIMP3	4

CC	GO:0043292	contractile fiber	19/737	245/19886	0.0020322597777323	0.0378871287134379	0.0339219301245542	CAPN3/CRYAB/CSRP2/DES/DNAJB6/ILK/MYH11/MYL5/MYZAP/PDLIM2/PGM5/PSMA6/SMN1/SMN2/SORBS2/SYNE1/TNNI2/TPM1/TPM2	19

CC	GO:0033177	proton-transporting two-sector ATPase complex, proton-transporting domain	5/737	26/19886	0.00237351005627563	0.0427231810129613	0.0382518499087614	ATP5MC3/ATP5PD/ATP5PO/ATP6V0C/RNASEK	5

CC	GO:0072562	blood microparticle	13/737	144/19886	0.00274074936332866	0.0476890389219186	0.0426979900813306	A1BG/ACTG2/C4A/C4B/CFB/CLU/HBA1/HBA2/HBB/IGLC1/PRSS1/SERPINA3/TF	13

CC	GO:0030017	sarcomere	17/737	216/19886	0.0029310885003433	0.0493557482961033	0.0441902646571282	CAPN3/CRYAB/CSRP2/DES/DNAJB6/ILK/MYZAP/PDLIM2/PGM5/PSMA6/SMN1/SMN2/SORBS2/SYNE1/TNNI2/TPM1/TPM2	17

MF	GO:0003735	structural constituent of ribosome	33/712	176/18496	3.27600395924099e-14	2.43407094171606e-11	2.29665119668895e-11	MRPL23/MRPL46/MRPL52/MRPS11/MRPS18C/MRPS24/MRPS33/NDUFA7/RPL13/RPL14/RPL15/RPL17/RPL18/RPL22/RPL23/RPL26/RPL27A/RPL30/RPL31/RPL32/RPL34/RPL36A/RPL37/RPL37A/RPL39/RPS15A/RPS19/RPS23/RPS24/RPS27L/RPS29/RPS3/RPS9	33

MF	GO:0004175	endopeptidase activity	33/712	389/18496	1.98400356691837e-05	0.00380091495573243	0.00358632763408344	ADAM33/ADAMTS10/CAPN10/CAPN14/CAPN3/CAPN5/CFB/CFD/CFLAR/CLCA4/HM13/JMJD7/KLK11/KLK12/KLK13/KLK8/PCSK7/PGA3/PGA4/PRSS1/PRSS2/PRSS22/PRSS27/PRSS3/PSMA6/SEC11C/TMPRSS11B/TMPRSS11E/TMPRSS2/TMPRSS4/TPSAB1/TPSB2/USP15	33

MF	GO:0008137	NADH dehydrogenase (ubiquinone) activity	9/712	41/18496	2.04395830457033e-05	0.00380091495573243	0.00358632763408344	NDUFA10/NDUFA7/NDUFA9/NDUFB1/NDUFB2/NDUFB8/NDUFC2/NDUFS7/NDUFV2	9

MF	GO:0050136	NADH dehydrogenase (quinone) activity	9/712	42/18496	2.51334361223259e-05	0.00380091495573243	0.00358632763408344	NDUFA10/NDUFA7/NDUFA9/NDUFB1/NDUFB2/NDUFB8/NDUFC2/NDUFS7/NDUFV2	9

MF	GO:0003954	NADH dehydrogenase activity	9/712	44/18496	3.73020329564322e-05	0.00380091495573243	0.00358632763408344	NDUFA10/NDUFA7/NDUFA9/NDUFB1/NDUFB2/NDUFB8/NDUFC2/NDUFS7/NDUFV2	9

MF	GO:0004601	peroxidase activity	10/712	55/18496	4.08503756623782e-05	0.00380091495573243	0.00358632763408344	GPX3/GSTM2/HBA1/HBA2/HBB/LTC4S/MGST1/MGST2/MGST3/TXNDC17	10

MF	GO:0004252	serine-type endopeptidase activity	19/712	174/18496	4.29839656269798e-05	0.00380091495573243	0.00358632763408344	CFB/CFD/KLK11/KLK12/KLK13/KLK8/PCSK7/PRSS1/PRSS2/PRSS22/PRSS27/PRSS3/SEC11C/TMPRSS11B/TMPRSS11E/TMPRSS2/TMPRSS4/TPSAB1/TPSB2	19

MF	GO:0003955	NAD(P)H dehydrogenase (quinone) activity	9/712	45/18496	4.50510136465983e-05	0.00380091495573243	0.00358632763408344	NDUFA10/NDUFA7/NDUFA9/NDUFB1/NDUFB2/NDUFB8/NDUFC2/NDUFS7/NDUFV2	9

MF	GO:0008236	serine-type peptidase activity	20/712	192/18496	5.36185276506951e-05	0.00380091495573243	0.00358632763408344	CFB/CFD/ENDOU/KLK11/KLK12/KLK13/KLK8/PCSK7/PRSS1/PRSS2/PRSS22/PRSS27/PRSS3/SEC11C/TMPRSS11B/TMPRSS11E/TMPRSS2/TMPRSS4/TPSAB1/TPSB2	20

MF	GO:0016655	oxidoreductase activity, acting on NAD(P)H, quinone or similar compound as acceptor	10/712	57/18496	5.62719576218798e-05	0.00380091495573243	0.00358632763408344	NDUFA10/NDUFA7/NDUFA9/NDUFB1/NDUFB2/NDUFB8/NDUFC2/NDUFS7/NDUFV2/TP53I3	10

MF	GO:0016684	oxidoreductase activity, acting on peroxide as acceptor	10/712	57/18496	5.62719576218798e-05	0.00380091495573243	0.00358632763408344	GPX3/GSTM2/HBA1/HBA2/HBB/LTC4S/MGST1/MGST2/MGST3/TXNDC17	10

MF	GO:0017171	serine hydrolase activity	20/712	196/18496	7.16857261841834e-05	0.00435833016584443	0.00411227299065296	CFB/CFD/ENDOU/KLK11/KLK12/KLK13/KLK8/PCSK7/PRSS1/PRSS2/PRSS22/PRSS27/PRSS3/SEC11C/TMPRSS11B/TMPRSS11E/TMPRSS2/TMPRSS4/TPSAB1/TPSB2	20

MF	GO:0016209	antioxidant activity	12/712	83/18496	7.62561132651112e-05	0.00435833016584443	0.00411227299065296	GPX3/GSTM2/HBA1/HBA2/HBB/LTC4S/MGST1/MGST2/MGST3/SELENOW/SRXN1/TXNDC17	12

MF	GO:0019825	oxygen binding	8/712	39/18496	9.8884301162674e-05	0.00524793112599048	0.00495164996799555	CYP2C18/CYP2E1/CYP3A5/CYP4B1/EGLN2/HBA1/HBA2/HBB	8

MF	GO:0004602	glutathione peroxidase activity	6/712	22/18496	0.000140081853120222	0.00693872112455498	0.00654698345109247	GPX3/GSTM2/LTC4S/MGST1/MGST2/MGST3	6

MF	GO:0008307	structural constituent of muscle	8/712	42/18496	0.000171334330609769	0.00795633797769113	0.00750714895961223	CAPN3/CSRP2/MYH11/MYL5/MYL6/SORBS2/TPM1/TPM2	8

MF	GO:0019843	rRNA binding	10/712	66/18496	0.000201362437218076	0.00834866207660219	0.00787732371327769	CIRBP/MRPS18C/MTERF4/NOL12/PPAN/RBM34/RPL23/RPL37/RPS3/RPS9	10

MF	GO:0015399	primary active transmembrane transporter activity	17/712	164/18496	0.000202255608854427	0.00834866207660219	0.00787732371327769	ABCA5/ABCA6/ABCA8/ABCB6/ABCD4/ATP13A4/ATP6V0C/CYB5A/NDUFA10/NDUFA7/NDUFA9/NDUFB1/NDUFB2/NDUFB8/NDUFC2/NDUFS7/NDUFV2	17

MF	GO:0015453	oxidoreduction-driven active transmembrane transporter activity	10/712	68/18496	0.000258978734125177	0.00979599398121568	0.00924294395620832	CYB5A/NDUFA10/NDUFA7/NDUFA9/NDUFB1/NDUFB2/NDUFB8/NDUFC2/NDUFS7/NDUFV2	10

MF	GO:0030332	cyclin binding	7/712	34/18496	0.000263687590342279	0.00979599398121568	0.00924294395620832	CDK3/CDK5RAP3/CRABP2/CUL3/GAK/GPS2/RBM4	7

MF	GO:0009055	electron transfer activity	13/712	117/18496	0.000569593420607823	0.0201527576910292	0.0190149984022461	ALDH2/CYB5A/ETFB/NDUFA10/NDUFA7/NDUFA9/NDUFB1/NDUFB2/NDUFB8/NDUFC2/NDUFS7/NDUFV2/SDHC	13

MF	GO:0051087	protein-folding chaperone binding	14/712	133/18496	0.000615426028989908	0.0207846154336137	0.0196111835075253	AHSA2P/CLU/CYP2E1/DNAJB6/DNLZ/GAK/GET4/HSPB6/MVD/PFDN6/RNF207/RPS3/TBCD/TTC4	14

MF	GO:0033130	acetylcholine receptor binding	4/712	12/18496	0.000841595103302854	0.027187180945827	0.0256522809519314	LYNX1/PSCA/RER1/RIC3	4

MF	GO:0031369	translation initiation factor binding	6/712	33/18496	0.00145000484988211	0.0448897334776004	0.0423554048255038	DAPL1/EIF3C/EIF3CL/EIF3F/EIF4E/RPS24	6
