# Supplementary material for: Gene Expression Profiling and Clinical Validation Reveal CEBPB for Early Diagnosis of Esophageal Squamous Cell Carcinoma
Source: Cancer Rep (Hoboken). 2026 Nov 8;9(11):e70753. doi: 10.1002/cnr2.70753 (PMC13745642; doi:10.1002/cnr2.70753)
Supplement: Supplementary file 12 [file CNR2-9-e70753-s007.doc]

Doc. S4. KEGG pathway enrichment analysis of the upregulated DEGs.

category	subcategory	ID	Description	GeneRatio	BgRatio	pvalue	p.adjust	qvalue	geneID	Count

Organismal Systems	Immune system	hsa04657	IL-17 signaling pathway	15/192	95/8846	1.55909001698e-09	3.18896514688913e-07	2.44508048949264e-07	CEBPB/CXCL10/CXCL8/FOSB/FOSL1/HSP90AA1/HSP90AB1/JUND/MMP1/MMP13/MMP3/MMP9/S100A7/S100A7A/TNFAIP3	15

Human Diseases	Infectious disease: viral	hsa05165	Human papillomavirus infection	27/192	333/8846	2.62466267233673e-09	3.18896514688913e-07	2.44508048949264e-07	CCNA1/CCNA2/CCND1/CDK6/COL1A1/COL1A2/COL4A1/COL4A2/CREB3L1/EGFR/EIF2AK2/FZD1/FZD2/FZD6/FZD7/ISG15/ITGA2/ITGA6/ITGAV/ITGB4/LAMB3/LAMC2/SPP1/STAT1/THBS2/TNC/WNT7B	27

Environmental Information Processing	Signaling molecules and interaction	hsa04512	ECM-receptor interaction	14/192	89/8846	5.87743636326008e-09	4.76072345424067e-07	3.65019732034047e-07	AGRN/COL1A1/COL1A2/COL4A1/COL4A2/ITGA2/ITGA6/ITGAV/ITGB4/LAMB3/LAMC2/SPP1/THBS2/TNC	14

Human Diseases	Cancer: overview	hsa05205	Proteoglycans in cancer	19/192	204/8846	8.08612107800548e-08	4.91231855488833e-06	3.76643008107097e-06	CCND1/COL1A1/COL1A2/EGFR/FZD1/FZD2/FZD6/FZD7/HIF1A/HOXD10/ITGA2/ITGAV/ITPR3/LUM/MET/MMP9/MSN/PLAU/WNT7B	19

NA	NA	hsa04820	Cytoskeleton in muscle cells	19/192	232/8846	6.0927542495685e-07	2.96107856529029e-05	2.270352636155e-05	AGRN/BGN/COL11A1/COL1A1/COL1A2/COL3A1/COL4A1/COL4A2/COL5A1/COL5A2/DSG2/ITGA2/ITGA6/ITGAV/ITGB4/MYH9/PLEC/THBS2/VCAN	19

Cellular Processes	Cellular community - eukaryotes	hsa04510	Focal adhesion	17/192	203/8846	1.80432868534734e-06	7.30753117565671e-05	5.60291539134173e-05	CCND1/COL1A1/COL1A2/COL4A1/COL4A2/EGFR/ITGA2/ITGA6/ITGAV/ITGB4/LAMB3/LAMC2/MET/PAK2/SPP1/THBS2/TNC	17

Environmental Information Processing	Signal transduction	hsa04151	PI3K-Akt signaling pathway	23/192	362/8846	3.34797876955625e-06	0.000116222691571738	8.91116153701439e-05	CCND1/CDK6/COL1A1/COL1A2/COL4A1/COL4A2/CREB3L1/EGFR/F2R/HSP90AA1/HSP90AB1/ITGA2/ITGA6/ITGAV/ITGB4/LAMB3/LAMC2/MET/SPP1/THBS2/TNC/YWHAG/YWHAQ	23

Human Diseases	Infectious disease: viral	hsa05169	Epstein-Barr virus infection	16/192	203/8846	8.0383947718862e-06	0.000244166241196043	0.000187209983503139	CCNA1/CCNA2/CCND1/CDK6/CXCL10/EIF2AK2/HLA-DRA/HLA-DRB1/ICAM1/ISG15/OAS2/OAS3/RUNX3/STAT1/TAPBP/TNFAIP3	16

Human Diseases	Infectious disease: viral	hsa05164	Influenza A	14/192	172/8846	2.10130862778386e-05	0.000567353329501643	0.000435007751014905	CCL5/CDK6/CXCL10/CXCL8/EIF2AK2/HLA-DRA/HLA-DRB1/ICAM1/KPNA2/OAS2/OAS3/RSAD2/SOCS3/STAT1	14

Human Diseases	Cancer: specific types	hsa05222	Small cell lung cancer	10/192	93/8846	3.10613117957059e-05	0.000754789876635652	0.00057872128293052	CCND1/CDK6/CKS2/COL4A1/COL4A2/ITGA2/ITGA6/ITGAV/LAMB3/LAMC2	10

Human Diseases	Infectious disease: viral	hsa05160	Hepatitis C	13/192	159/8846	3.9786175960918e-05	0.000878912796227553	0.000673890253117942	CCND1/CDK6/CLDN1/CXCL10/EGFR/EIF2AK2/OAS2/OAS3/RSAD2/SOCS3/STAT1/YWHAG/YWHAQ	13

Human Diseases	Infectious disease: bacterial	hsa05150	Staphylococcus aureus infection	10/192	100/8846	5.83541450441115e-05	0.00118167143714326	0.000906024883579626	C1QA/C1QB/C1QC/FCGR3A/HLA-DRA/HLA-DRB1/ICAM1/KRT10/KRT14/KRT17	10

Human Diseases	Endocrine and metabolic disease	hsa04933	AGE-RAGE signaling pathway in diabetic complications	10/192	101/8846	6.35626440564933e-05	0.0011881325004406	0.000910978785263102	CCND1/COL1A1/COL1A2/COL3A1/COL4A1/COL4A2/CXCL8/ICAM1/SERPINE1/STAT1	10

Human Diseases	Endocrine and metabolic disease	hsa04934	Cushing syndrome	12/192	155/8846	0.000133806865427427	0.00232250487849034	0.00178073798350786	AHR/CCND1/CDK6/CREB3L1/EGFR/FZD1/FZD2/FZD6/FZD7/ITPR3/NCEH1/WNT7B	12

Human Diseases	Infectious disease: parasitic	hsa05146	Amoebiasis	9/192	103/8846	0.000383406440230136	0.0062111843317282	0.00476231157338484	CD14/COL1A1/COL1A2/COL3A1/COL4A1/COL4A2/CXCL8/LAMB3/LAMC2	9

Organismal Systems	Digestive system	hsa04974	Protein digestion and absorption	9/192	105/8846	0.000442319952201894	0.00671773427406627	0.00515069944340364	COL10A1/COL11A1/COL1A1/COL1A2/COL3A1/COL4A1/COL4A2/COL5A1/COL5A2	9

Organismal Systems	Endocrine system	hsa04926	Relaxin signaling pathway	10/192	130/8846	0.00051194424473546	0.00731779126298334	0.00561078212496448	COL1A1/COL1A2/COL3A1/COL4A1/COL4A2/CREB3L1/EGFR/MMP1/MMP13/MMP9	10

Environmental Information Processing	Signal transduction	hsa04390	Hippo signaling pathway	11/192	157/8846	0.000600555957890096	0.0081075054315163	0.00621628096763433	CCND1/FZD1/FZD2/FZD6/FZD7/GLI2/SERPINE1/SNAI2/WNT7B/YWHAG/YWHAQ	11

Environmental Information Processing	Signal transduction	hsa04668	TNF signaling pathway	9/192	119/8846	0.00109777651744421	0.014039983880997	0.0107648999217521	CCL5/CEBPB/CREB3L1/CXCL10/ICAM1/MMP3/MMP9/SOCS3/TNFAIP3	9

Human Diseases	Cancer: specific types	hsa05215	Prostate cancer	8/192	98/8846	0.00126382823286881	0.0153555130293561	0.0117735577483042	CCND1/CREB3L1/EGFR/HSP90AA1/HSP90AB1/MMP3/MMP9/PLAU	8

Environmental Information Processing	Signaling molecules and interaction	hsa04061	Viral protein interaction with cytokine and cytokine receptor	8/192	100/8846	0.00144055154373224	0.0166692392917588	0.0127808332451432	ACKR3/CCL18/CCL5/CXCL10/CXCL13/CXCL14/CXCL8/CXCL9	8

Human Diseases	Cancer: specific types	hsa05219	Bladder cancer	5/192	41/8846	0.00181677528190663	0.020067108795605	0.0153860873156686	CCND1/CXCL8/EGFR/MMP1/MMP9	5

Human Diseases	Infectious disease: viral	hsa05171	Coronavirus disease - COVID-19	13/192	238/8846	0.00200469300622122	0.0205186058791338	0.0157322644167497	C1QA/C1QB/C1QC/CXCL10/CXCL8/EGFR/EIF2AK2/ISG15/MMP1/MMP3/OAS2/OAS3/STAT1	13

Cellular Processes	Cell growth and death	hsa04216	Ferroptosis	5/192	42/8846	0.00202652897571692	0.0205186058791338	0.0157322644167497	FTL/GCLM/PRNP/SLC7A11/TFRC	5

Cellular Processes	Cell growth and death	hsa04218	Cellular senescence	10/192	157/8846	0.0021726726758016	0.0211183784087915	0.016192128994395	CCNA1/CCNA2/CCND1/CDK6/CXCL8/FOXM1/IGFBP3/ITPR3/MYBL2/SERPINE1	10

Human Diseases	Cancer: specific types	hsa05217	Basal cell carcinoma	6/192	63/8846	0.00235189356939792	0.0211758166502342	0.0162361687116806	FZD1/FZD2/FZD6/FZD7/GLI2/WNT7B	6

Organismal Systems	Immune system	hsa04659	Th17 cell differentiation	8/192	108/8846	0.00235286851669269	0.0211758166502342	0.0162361687116806	AHR/HIF1A/HLA-DRA/HLA-DRB1/HSP90AA1/HSP90AB1/IL27RA/STAT1	8

Human Diseases	Cancer: overview	hsa05202	Transcriptional misregulation in cancer	11/192	193/8846	0.00317189846433503	0.0269102139042258	0.0206329125451504	CCNA1/CCNA2/CD14/CEBPB/CXCL8/IGFBP3/MET/MLF1/MMP3/MMP9/PLAU	11

Organismal Systems	Endocrine system	hsa04915	Estrogen signaling pathway	9/192	139/8846	0.00321150700914629	0.0269102139042258	0.0206329125451504	CREB3L1/EGFR/HSP90AA1/HSP90AB1/ITPR3/KRT10/KRT14/KRT17/MMP9	9

Organismal Systems	Endocrine system	hsa04928	Parathyroid hormone synthesis, secretion and action	8/192	115/8846	0.00347739043821248	0.028166862549521	0.0215964248267933	CREB3L1/EGFR/GNA12/GNA13/ITPR3/JUND/MMP13/PTHLH	8

Organismal Systems	Development and regeneration	hsa04380	Osteoclast differentiation	9/192	142/8846	0.00370323161274314	0.029028557480535	0.0222571135978111	FCGR3A/FOSB/FOSL1/ITPR3/JUND/SOCS1/SOCS3/STAT1/TREM2	9

Human Diseases	Immune disease	hsa05323	Rheumatoid arthritis	7/192	94/8846	0.00424191415086859	0.0316097331491006	0.0242361826614287	CCL5/CXCL8/HLA-DRA/HLA-DRB1/ICAM1/MMP1/MMP3	7

Cellular Processes	Cell motility	hsa04810	Regulation of actin cytoskeleton	12/192	230/8846	0.00429267981037169	0.0316097331491006	0.0242361826614287	EGFR/F2R/GNA12/GNA13/ITGA2/ITGA6/ITGAV/ITGB4/MSN/MYH9/PAK2/PFN2	12

Organismal Systems	Immune system	hsa04611	Platelet activation	8/192	126/8846	0.00603828569079578	0.0431559830253934	0.0330890578102432	COL1A1/COL1A2/COL3A1/F2R/FCER1G/GNA13/ITGA2/ITPR3	8

Cellular Processes	Transport and catabolism	hsa04145	Phagosome	9/192	157/8846	0.00711064229597334	0.04799683549782	0.0368006925844234	CD14/FCGR3A/HLA-DRA/HLA-DRB1/ITGA2/ITGAV/TFRC/THBS2/TUBB	9

NA	NA	hsa04148	Efferocytosis	9/192	157/8846	0.00711064229597334	0.04799683549782	0.0368006925844234	C1QA/C1QB/C1QC/CEBPB/HIF1A/ITGAV/ODC1/PANX1/SLC16A1	9

Cellular Processes	Cell growth and death	hsa04110	Cell cycle	9/192	158/8846	0.00740262709068223	0.0486172535955617	0.0372763867724255	CCNA1/CCNA2/CCND1/CDK6/MCM2/PCNA/TRIP13/YWHAG/YWHAQ	9

Organismal Systems	Immune system	hsa04612	Antigen processing and presentation	6/192	80/8846	0.0076858303298936	0.0489607972359101	0.0375397925525497	HLA-DRA/HLA-DRB1/HSP90AA1/HSP90AB1/HSPA5/TAPBP	6

Human Diseases	Cancer: overview	hsa05206	MicroRNAs in cancer	14/192	312/8846	0.00793238631614192	0.0489607972359101	0.0375397925525497	CCND1/CDK6/EGFR/FSCN1/HOXD10/IRS2/MARCKS/MET/MMP9/PLAU/SOCS1/SOX4/TNC/TP63	14

Organismal Systems	Immune system	hsa04621	NOD-like receptor signaling pathway	10/192	189/8846	0.00805939049150784	0.0489607972359101	0.0375397925525497	CCL5/CXCL8/HSP90AA1/HSP90AB1/ITPR3/OAS2/OAS3/PANX1/STAT1/TNFAIP3	10

Human Diseases	Infectious disease: viral	hsa05161	Hepatitis B	9/192	163/8846	0.0090025401157402	0.053356518246948	0.0409101309495768	CCNA1/CCNA2/CREB3L1/CXCL8/EGR2/MMP9/PCNA/STAT1/YWHAQ	9

Organismal Systems	Immune system	hsa04620	Toll-like receptor signaling pathway	7/192	109/8846	0.00942072305095054	0.0545056119376424	0.0417911774440663	CCL5/CD14/CXCL10/CXCL8/CXCL9/SPP1/STAT1	7

Human Diseases	Infectious disease: viral	hsa05163	Human cytomegalovirus infection	11/192	226/8846	0.0101547053687422	0.0571183293007412	0.0437944305229855	CCL5/CCND1/CDK6/CREB3L1/CXCL8/EGFR/GNA12/GNA13/ITGAV/ITPR3/TAPBP	11

Human Diseases	Infectious disease: parasitic	hsa05145	Toxoplasmosis	7/192	111/8846	0.0103586698778911	0.0571183293007412	0.0437944305229855	HLA-DRA/HLA-DRB1/ITGA6/LAMB3/LAMC2/SOCS1/STAT1	7

Human Diseases	Infectious disease: viral	hsa05162	Measles	8/192	139/8846	0.0106791543615641	0.0571183293007412	0.0437944305229855	CCND1/CDK6/EIF2AK2/MSN/OAS2/OAS3/STAT1/TNFAIP3	8

Human Diseases	Cardiovascular disease	hsa05412	Arrhythmogenic right ventricular cardiomyopathy	6/192	86/8846	0.0108125232421156	0.0571183293007412	0.0437944305229855	DSG2/GJA1/ITGA2/ITGA6/ITGAV/ITGB4	6

Human Diseases	Cancer: specific types	hsa05225	Hepatocellular carcinoma	9/192	170/8846	0.0116670880360746	0.0603213275056625	0.0462502706021324	CCND1/CDK6/EGFR/FZD1/FZD2/FZD6/FZD7/MET/WNT7B	9

Organismal Systems	Immune system	hsa04610	Complement and coagulation cascades	6/192	88/8846	0.0120301194380168	0.06090247965496	0.0466958583449336	C1QA/C1QB/C1QC/F2R/PLAU/SERPINE1	6

Environmental Information Processing	Signal transduction	hsa04310	Wnt signaling pathway	9/192	174/8846	0.013433274214718	0.066618074166867	0.0510781855210546	CCND1/FOSL1/FZD1/FZD2/FZD6/FZD7/MMP7/SFRP4/WNT7B	9

Human Diseases	Cancer: specific types	hsa05224	Breast cancer	8/192	148/8846	0.0151703234033106	0.0737277717400893	0.0565294156291783	CCND1/CDK6/EGFR/FZD1/FZD2/FZD6/FZD7/WNT7B	8

Human Diseases	Cancer: specific types	hsa05226	Gastric cancer	8/192	150/8846	0.0163307251077068	0.0778111019837796	0.0596602341396101	CCND1/EGFR/FZD1/FZD2/FZD6/FZD7/MET/WNT7B	8

Organismal Systems	Endocrine system	hsa04935	Growth hormone synthesis, secretion and action	7/192	122/8846	0.0167692492529869	0.0783639917014582	0.0600841521817548	CREB3L1/IGFBP3/IRS2/ITPR3/SOCS1/SOCS3/STAT1	7

Human Diseases	Cardiovascular disease	hsa05417	Lipid and atherosclerosis	10/192	216/8846	0.019181186864594	0.087943932228233	0.0674293957305491	CCL5/CD14/CXCL8/HSP90AA1/HSP90AB1/HSPA5/ICAM1/MMP1/MMP3/MMP9	10

Organismal Systems	Immune system	hsa04640	Hematopoietic cell lineage	6/192	99/8846	0.0204885947755964	0.092198676490184	0.0706916427929936	CD14/HLA-DRA/HLA-DRB1/ITGA2/ITGA6/TFRC	6

Environmental Information Processing	Signaling molecules and interaction	hsa04514	Cell adhesion molecules	8/192	157/8846	0.0208989894765477	0.0923355353236563	0.0707965767913674	ALCAM/CLDN1/HLA-DRA/HLA-DRB1/ICAM1/ITGA6/ITGAV/VCAN	8

Organismal Systems	Endocrine system	hsa04916	Melanogenesis	6/192	101/8846	0.0223723559086755	0.0961790924450985	0.073743553661609	CREB3L1/FZD1/FZD2/FZD6/FZD7/WNT7B	6

Human Diseases	Infectious disease: parasitic	hsa05144	Malaria	4/192	50/8846	0.0227846680183814	0.0961790924450985	0.073743553661609	CXCL8/ICAM1/MET/THBS2	4

Cellular Processes	Cell growth and death	hsa04115	p53 signaling pathway	5/192	75/8846	0.0232074007247113	0.0961790924450985	0.073743553661609	CCND1/CDK6/IGFBP3/SERPINE1/SESN3	5

Human Diseases	Infectious disease: viral	hsa05166	Human T-cell leukemia virus 1 infection	10/192	223/8846	0.0233521253261762	0.0961790924450985	0.073743553661609	CCNA1/CCNA2/CCND1/CREB3L1/EGR2/FOSL1/HLA-DRA/HLA-DRB1/ICAM1/MMP7	10

Human Diseases	Infectious disease: parasitic	hsa05142	Chagas disease	6/192	103/8846	0.0243696937003974	0.0986972594866095	0.0756743120170235	C1QA/C1QB/C1QC/CCL5/CXCL8/SERPINE1	6

Human Diseases	Infectious disease: parasitic	hsa05140	Leishmaniasis	5/192	77/8846	0.0256705884104484	0.10226152432359	0.078407146654864	FCGR3A/HLA-DRA/HLA-DRB1/MARCKSL1/STAT1	5

Human Diseases	Infectious disease: bacterial	hsa05133	Pertussis	5/192	78/8846	0.0269626307178807	0.105676117168468	0.0810252230401507	C1QA/C1QB/C1QC/CD14/CXCL8	5

Human Diseases	Immune disease	hsa05310	Asthma	3/192	31/8846	0.0289725121212768	0.111751118182068	0.0856831185541521	FCER1G/HLA-DRA/HLA-DRB1	3

Human Diseases	Immune disease	hsa05322	Systemic lupus erythematosus	7/192	139/8846	0.0315298532590767	0.119714911593057	0.0917892109680358	C1QA/C1QB/C1QC/FCGR3A/HLA-DRA/HLA-DRB1/SNRPB	7

Human Diseases	Cancer: overview	hsa05203	Viral carcinogenesis	9/192	205/8846	0.0344441331384452	0.128768066963726	0.098730551668094	CCNA1/CCNA2/CCND1/CDK6/CREB3L1/EGR2/EIF2AK2/YWHAG/YWHAQ	9

Cellular Processes	Cellular community - eukaryotes	hsa04550	Signaling pathways regulating pluripotency of stem cells	7/192	144/8846	0.0371446179492382	0.136759729722195	0.104858012392586	DLX5/FZD1/FZD2/FZD6/FZD7/INHBA/WNT7B	7

Organismal Systems	Immune system	hsa04062	Chemokine signaling pathway	8/192	193/8846	0.0590098165727575	0.210894324299574	0.161699351964586	CCL18/CCL5/CXCL10/CXCL13/CXCL14/CXCL8/CXCL9/STAT1	8

Environmental Information Processing	Signaling molecules and interaction	hsa04060	Cytokine-cytokine receptor interaction	11/192	298/8846	0.059684216186024	0.210894324299574	0.161699351964586	ACKR3/CCL18/CCL5/CXCL10/CXCL13/CXCL14/CXCL8/CXCL9/IL27RA/INHBA/TNFRSF12A	11

Human Diseases	Cancer: specific types	hsa05221	Acute myeloid leukemia	4/192	68/8846	0.0598835735665457	0.210894324299574	0.161699351964586	CCNA1/CCNA2/CCND1/CD14	4

Human Diseases	Cardiovascular disease	hsa05416	Viral myocarditis	4/192	69/8846	0.0625376755427345	0.217052606181377	0.166421101555571	CCND1/HLA-DRA/HLA-DRB1/ICAM1	4

Human Diseases	Infectious disease: viral	hsa05167	Kaposi sarcoma-associated herpesvirus infection	8/192	196/8846	0.063418662711431	0.217052606181377	0.166421101555571	CCND1/CDK6/CXCL8/EIF2AK2/HIF1A/ICAM1/ITPR3/STAT1	8

Organismal Systems	Endocrine system	hsa04917	Prolactin signaling pathway	4/192	71/8846	0.0680291025882537	0.223392863904671	0.171282377782659	CCND1/SOCS1/SOCS3/STAT1	4

Human Diseases	Infectious disease: bacterial	hsa05120	Epithelial cell signaling in Helicobacter pylori infection	4/192	71/8846	0.0680291025882537	0.223392863904671	0.171282377782659	CCL5/CXCL8/EGFR/MET	4

Human Diseases	Cancer: overview	hsa05230	Central carbon metabolism in cancer	4/192	71/8846	0.0680291025882537	0.223392863904671	0.171282377782659	EGFR/HIF1A/MET/SLC7A5	4

Human Diseases	Cancer: specific types	hsa05218	Melanoma	4/192	73/8846	0.0737619942521574	0.234146937955132	0.179527866658256	CCND1/CDK6/EGFR/MET	4

Human Diseases	Cancer: specific types	hsa05223	Non-small cell lung cancer	4/192	73/8846	0.0737619942521574	0.234146937955132	0.179527866658256	CCND1/CDK6/EGFR/MET	4

Human Diseases	Infectious disease: bacterial	hsa05130	Pathogenic Escherichia coli infection	8/192	203/8846	0.0744895928742312	0.234146937955132	0.179527866658256	CLDN1/CXCL8/F2R/GNA12/GNA13/MYH9/PAK2/TUBB	8

Metabolism	Glycan biosynthesis and metabolism	hsa00532	Glycosaminoglycan biosynthesis - chondroitin sulfate / dermatan sulfate	2/192	21/8846	0.0751582763806597	0.234146937955132	0.179527866658256	CHPF/CHST11	2

Environmental Information Processing	Signal transduction	hsa04064	NF-kappa B signaling pathway	5/192	105/8846	0.0779491013297909	0.239767488900496	0.183837320924357	CD14/CXCL8/ICAM1/PLAU/TNFAIP3	5

Organismal Systems	Endocrine system	hsa04918	Thyroid hormone synthesis	4/192	75/8846	0.0797324664843859	0.241540339917106	0.185196621898755	CREB3L1/HSPA5/ITPR3/PDIA4	4

Human Diseases	Neurodegenerative disease	hsa05020	Prion disease	10/192	278/8846	0.0806977184275985	0.241540339917106	0.185196621898755	C1QA/C1QB/C1QC/CCL5/CREB3L1/GRIN2D/HSPA5/ITPR3/PRNP/TUBB	10

Human Diseases	Endocrine and metabolic disease	hsa04930	Type II diabetes mellitus	3/192	47/8846	0.0815074398074183	0.241540339917106	0.185196621898755	IRS2/SOCS1/SOCS3	3

Human Diseases	Cancer: specific types	hsa05212	Pancreatic cancer	4/192	77/8846	0.0859361149736049	0.251596095645614	0.192906688019379	CCND1/CDK6/EGFR/STAT1	4

Human Diseases	Substance dependence	hsa05030	Cocaine addiction	3/192	49/8846	0.0898080790010171	0.259801942824371	0.199198370716542	CREB3L1/FOSB/GRIN2D	3

Human Diseases	Endocrine and metabolic disease	hsa04936	Alcoholic liver disease	6/192	144/8846	0.0931528298310764	0.266307501752371	0.204186388608056	C1QA/C1QB/C1QC/CCND1/CD14/CXCL8	6

Human Diseases	Infectious disease: bacterial	hsa05152	Tuberculosis	7/192	180/8846	0.0967617215008297	0.27340812005467	0.209630657351859	CD14/CEBPB/FCER1G/FCGR3A/HLA-DRA/HLA-DRB1/STAT1	7

Environmental Information Processing	Signal transduction	hsa04072	Phospholipase D signaling pathway	6/192	149/8846	0.105302015442464	0.294119422442745	0.225510668279687	CXCL8/EGFR/F2R/FCER1G/GNA12/GNA13	6

Environmental Information Processing	Signal transduction	hsa04152	AMPK signaling pathway	5/192	122/8846	0.125897132828999	0.34764776451644	0.266552541994412	CCNA1/CCNA2/CCND1/CREB3L1/IRS2	5

Environmental Information Processing	Signal transduction	hsa04150	mTOR signaling pathway	6/192	158/8846	0.129129195807529	0.352566231249771	0.270323686078447	FZD1/FZD2/FZD6/FZD7/SLC7A5/WNT7B	6

Cellular Processes	Cell growth and death	hsa04217	Necroptosis	6/192	159/8846	0.131926385481937	0.356201240801231	0.27311076292752	EIF2AK2/FTL/HSP90AA1/HSP90AB1/STAT1/TNFAIP3	6

Cellular Processes	Cellular community - eukaryotes	hsa04540	Gap junction	4/192	92/8846	0.139335974850279	0.368028716180629	0.282179262568644	EGFR/GJA1/ITPR3/TUBB	4

Organismal Systems	Immune system	hsa04658	Th1 and Th2 cell differentiation	4/192	92/8846	0.139335974850279	0.368028716180629	0.282179262568644	HLA-DRA/HLA-DRB1/RUNX3/STAT1	4

Organismal Systems	Nervous system	hsa04730	Long-term depression	3/192	60/8846	0.141059629070097	0.368575159828317	0.282598238204947	GNA12/GNA13/ITPR3	3

Human Diseases	Neurodegenerative disease	hsa05010	Alzheimer disease	12/192	391/8846	0.142940375104021	0.369516076066778	0.283319668459258	APP/EIF2AK2/FZD1/FZD2/FZD6/FZD7/GRIN2D/IRS2/ITPR3/SLC39A6/TUBB/WNT7B	12

Environmental Information Processing	Signal transduction	hsa04630	JAK-STAT signaling pathway	6/192	168/8846	0.158369427041093	0.405092323905111	0.310597103449013	CCND1/EGFR/IL27RA/SOCS1/SOCS3/STAT1	6

Organismal Systems	Endocrine system	hsa04927	Cortisol synthesis and secretion	3/192	65/8846	0.166977818497315	0.416310576437289	0.319198492654972	CREB3L1/ITPR3/NCEH1	3

Human Diseases	Immune disease	hsa05321	Inflammatory bowel disease	3/192	65/8846	0.166977818497315	0.416310576437289	0.319198492654972	HLA-DRA/HLA-DRB1/STAT1	3

Human Diseases	Cardiovascular disease	hsa05410	Hypertrophic cardiomyopathy	4/192	99/8846	0.167894800373886	0.416310576437289	0.319198492654972	ITGA2/ITGA6/ITGAV/ITGB4	4

Genetic Information Processing	Replication and repair	hsa03030	DNA replication	2/192	36/8846	0.183468640794836	0.440940229099	0.338082826729578	MCM2/PCNA	2

Human Diseases	Cancer: overview	hsa05207	Chemical carcinogenesis - receptor activation	7/192	215/8846	0.186593736226757	0.440940229099	0.338082826729578	AHR/CCND1/CREB3L1/EGFR/HSP90AA1/HSP90AB1/KPNA2	7

Environmental Information Processing	Signal transduction	hsa04371	Apelin signaling pathway	5/192	140/8846	0.188114485612912	0.440940229099	0.338082826729578	CCND1/GNA13/ITPR3/SERPINE1/SPP1	5

Human Diseases	Substance dependence	hsa05031	Amphetamine addiction	3/192	69/8846	0.188634328522574	0.440940229099	0.338082826729578	CREB3L1/FOSB/GRIN2D	3

Human Diseases	Infectious disease: parasitic	hsa05143	African trypanosomiasis	2/192	37/8846	0.191346979304165	0.440940229099	0.338082826729578	F2RL1/ICAM1	2

Human Diseases	Cardiovascular disease	hsa05418	Fluid shear stress and atherosclerosis	5/192	141/8846	0.19185953354998	0.440940229099	0.338082826729578	HSP90AA1/HSP90AB1/ICAM1/ITGAV/MMP9	5

Organismal Systems	Immune system	hsa04625	C-type lectin receptor signaling pathway	4/192	105/8846	0.193874863728899	0.440940229099	0.338082826729578	EGR2/FCER1G/ITPR3/STAT1	4

Human Diseases	Cardiovascular disease	hsa05414	Dilated cardiomyopathy	4/192	105/8846	0.193874863728899	0.440940229099	0.338082826729578	ITGA2/ITGA6/ITGAV/ITGB4	4

Human Diseases	Cancer: specific types	hsa05211	Renal cell carcinoma	3/192	70/8846	0.194158866311083	0.440940229099	0.338082826729578	HIF1A/MET/PAK2	3

Human Diseases	Immune disease	hsa05330	Allograft rejection	2/192	38/8846	0.199269513661832	0.448356405739122	0.343769044036494	HLA-DRA/HLA-DRB1	2

Organismal Systems	Immune system	hsa04622	RIG-I-like receptor signaling pathway	3/192	72/8846	0.205325928590875	0.457744960069566	0.350967545732351	CXCL10/CXCL8/ISG15	3

Environmental Information Processing	Signal transduction	hsa04066	HIF-1 signaling pathway	4/192	109/8846	0.211850649842908	0.467997344652969	0.358828373418131	EGFR/HIF1A/SERPINE1/TFRC	4

Organismal Systems	Endocrine system	hsa04914	Progesterone-mediated oocyte maturation	4/192	111/8846	0.221010788833617	0.48383442960873	0.370971167601235	CCNA1/CCNA2/HSP90AA1/HSP90AB1	4

Human Diseases	Cancer: specific types	hsa05214	Glioma	3/192	76/8846	0.22807546313232	0.494842299474587	0.379411249759593	CCND1/CDK6/EGFR	3

Human Diseases	Endocrine and metabolic disease	hsa04940	Type I diabetes mellitus	2/192	43/8846	0.239356960638845	0.514723375533091	0.39465469988892	HLA-DRA/HLA-DRB1	2

Organismal Systems	Immune system	hsa04670	Leukocyte transendothelial migration	4/192	116/8846	0.24434817310721	0.520847421623264	0.399350199815109	CLDN1/ICAM1/MMP9/MSN	4

Human Diseases	Immune disease	hsa05332	Graft-versus-host disease	2/192	44/8846	0.247434696591579	0.522840271928292	0.400878181205578	HLA-DRA/HLA-DRB1	2

Human Diseases	Neurodegenerative disease	hsa05022	Pathways of neurodegeneration - multiple diseases	13/192	483/8846	0.250036222982682	0.5237827774551	0.401600830017556	APP/FZD1/FZD2/FZD6/FZD7/GRIN2D/HSPA5/ITPR3/NEFL/PRNP/TUBB/UCHL1/WNT7B	13

Cellular Processes	Cell motility	hsa04814	Motor proteins	6/192	197/8846	0.256581560048432	0.532900163177513	0.408591418160796	KIF11/KIF14/KIF20A/KIF4A/MYH9/TUBB	6

Metabolism	Glycan biosynthesis and metabolism	hsa00533	Glycosaminoglycan biosynthesis - keratan sulfate	1/192	14/8846	0.264673598924791	0.545048174056985	0.417905682512828	CHST2	1

Organismal Systems	Endocrine system	hsa04919	Thyroid hormone signaling pathway	4/192	122/8846	0.27303335463668	0.557538698963977	0.427482563208247	CCND1/HIF1A/ITGAV/STAT1	4

Metabolism	Global and overview maps	hsa01232	Nucleotide metabolism	3/192	85/8846	0.280723886128853	0.568465869410928	0.435860770568483	HPRT1/NT5E/TK1	3

Organismal Systems	Immune system	hsa04672	Intestinal immune network for IgA production	2/192	49/8846	0.287883648905018	0.578005915979117	0.443175426156828	HLA-DRA/HLA-DRB1	2

Environmental Information Processing	Signal transduction	hsa04022	cGMP-PKG signaling pathway	5/192	166/8846	0.29251060502167	0.578005915979117	0.443175426156828	CREB3L1/GNA12/GNA13/IRS2/ITPR3	5

Metabolism	Amino acid metabolism	hsa00330	Arginine and proline metabolism	2/192	50/8846	0.295958543898405	0.578005915979117	0.443175426156828	ODC1/P4HA1	2

Human Diseases	Infectious disease: bacterial	hsa05132	Salmonella infection	7/192	251/8846	0.303513462371637	0.578005915979117	0.443175426156828	CD14/CSE1L/CXCL8/HSP90AA1/HSP90AB1/PFN2/TUBB	7

Organismal Systems	Digestive system	hsa04979	Cholesterol metabolism	2/192	51/8846	0.304020249394536	0.578005915979117	0.443175426156828	APOC1/NCEH1	2

Human Diseases	Infectious disease: bacterial	hsa05110	Vibrio cholerae infection	2/192	51/8846	0.304020249394536	0.578005915979117	0.443175426156828	KDELR3/PDIA4	2

Genetic Information Processing	Folding, sorting and degradation	hsa04141	Protein processing in endoplasmic reticulum	5/192	170/8846	0.309507630449298	0.578005915979117	0.443175426156828	HSP90AA1/HSP90AB1/HSPA5/PDIA4/RPN2	5

Cellular Processes	Cellular community - eukaryotes	hsa04530	Tight junction	5/192	170/8846	0.309507630449298	0.578005915979117	0.443175426156828	CCND1/CLDN1/MSN/MYH9/PCNA	5

Organismal Systems	Aging	hsa04211	Longevity regulating pathway	3/192	90/8846	0.31047132560918	0.578005915979117	0.443175426156828	CREB3L1/IRS2/SESN3	3

Human Diseases	Cancer: overview	hsa05235	PD-L1 expression and PD-1 checkpoint pathway in cancer	3/192	90/8846	0.31047132560918	0.578005915979117	0.443175426156828	EGFR/HIF1A/STAT1	3

Metabolism	Energy metabolism	hsa00910	Nitrogen metabolism	1/192	17/8846	0.311599897091623	0.578005915979117	0.443175426156828	CA9	1

Human Diseases	Immune disease	hsa05320	Autoimmune thyroid disease	2/192	53/8846	0.320091364075415	0.589259102047923	0.451803600010753	HLA-DRA/HLA-DRB1	2

Cellular Processes	Cellular community - eukaryotes	hsa04520	Adherens junction	3/192	93/8846	0.328379117311515	0.599970868471415	0.460016650289974	EGFR/MET/SNAI2	3

Organismal Systems	Circulatory system	hsa04270	Vascular smooth muscle contraction	4/192	134/8846	0.331827204241948	0.601746347990996	0.461377966620777	GNA12/GNA13/ITPR3/MYH9	4

Environmental Information Processing	Signal transduction	hsa04340	Hedgehog signaling pathway	2/192	56/8846	0.344023260049765	0.614688619059507	0.471301215393254	CCND1/GLI2	2

Human Diseases	Infectious disease: bacterial	hsa05134	Legionellosis	2/192	56/8846	0.344023260049765	0.614688619059507	0.471301215393254	CD14/CXCL8	2

Organismal Systems	Immune system	hsa04666	Fc gamma R-mediated phagocytosis	3/192	98/8846	0.358196711204233	0.627566708921961	0.481175254404103	FCGR3A/MARCKS/MARCKSL1	3

Metabolism	Nucleotide metabolism	hsa00240	Pyrimidine metabolism	2/192	58/8846	0.359830184033218	0.627566708921961	0.481175254404103	NT5E/TK1	2

Metabolism	Metabolism of other amino acids	hsa00480	Glutathione metabolism	2/192	58/8846	0.359830184033218	0.627566708921961	0.481175254404103	GCLM/ODC1	2

Human Diseases	Drug resistance: antineoplastic	hsa01522	Endocrine resistance	3/192	99/8846	0.364143645917681	0.627566708921961	0.481175254404103	CCND1/EGFR/MMP9	3

Human Diseases	Cancer: overview	hsa05231	Choline metabolism in cancer	3/192	99/8846	0.364143645917681	0.627566708921961	0.481175254404103	EGFR/HIF1A/SLC44A1	3

Human Diseases	Cancer: specific types	hsa05213	Endometrial cancer	2/192	59/8846	0.367682081480005	0.628468218980766	0.481866470693505	CCND1/EGFR	2

Organismal Systems	Development and regeneration	hsa04360	Axon guidance	5/192	184/8846	0.369839322280862	0.628468218980766	0.481866470693505	EFNB1/MET/PAK2/PLXNA1/SEMA3C	5

Environmental Information Processing	Signal transduction	hsa04330	Notch signaling pathway	2/192	62/8846	0.391005594484392	0.659821940692411	0.50590636128463	DTX3L/NRARP	2

Metabolism	Glycan biosynthesis and metabolism	hsa00515	Mannose type O-glycan biosynthesis	1/192	23/8846	0.39670054339099	0.66026186331514	0.506243663880355	POMK	1

Genetic Information Processing	Replication and repair	hsa03430	Mismatch repair	1/192	23/8846	0.39670054339099	0.66026186331514	0.506243663880355	PCNA	1

Organismal Systems	Endocrine system	hsa04929	GnRH secretion	2/192	65/8846	0.413940521790046	0.684269025816199	0.524650715050757	ITPR3/SPP1	2

Environmental Information Processing	Signal transduction	hsa04350	TGF-beta signaling pathway	3/192	108/8846	0.417141541677352	0.684901315051327	0.525135511215443	FST/INHBA/TFRC	3

Human Diseases	Endocrine and metabolic disease	hsa04931	Insulin resistance	3/192	109/8846	0.422951661257571	0.689780226077784	0.52887632668732	CREB3L1/IRS2/SOCS3	3

Organismal Systems	Nervous system	hsa04720	Long-term potentiation	2/192	67/8846	0.428991963985168	0.694966981655972	0.532853176318419	GRIN2D/ITPR3	2

Metabolism	Glycan biosynthesis and metabolism	hsa00563	Glycosylphosphatidylinositol (GPI)-anchor biosynthesis	1/192	26/8846	0.43523969939689	0.70041885399632	0.537033299360401	PIGT	1

Metabolism	Lipid metabolism	hsa00062	Fatty acid elongation	1/192	27/8846	0.447533801178726	0.715465221621253	0.548569825544561	PPT1	1

Organismal Systems	Endocrine system	hsa04920	Adipocytokine signaling pathway	2/192	70/8846	0.451182428175993	0.716583856514812	0.549427518315451	IRS2/SOCS3	2

Human Diseases	Infectious disease: bacterial	hsa05131	Shigellosis	6/192	249/8846	0.456356599811425	0.720095154247897	0.552119741398648	CCL5/CD14/CXCL8/EGFR/ITPR3/PFN2	6

Environmental Information Processing	Signal transduction	hsa04015	Rap1 signaling pathway	5/192	212/8846	0.489598421530843	0.767563976980612	0.588515589887669	EGFR/F2R/MET/PFN2/SIPA1L3	5

Genetic Information Processing	Folding, sorting and degradation	hsa03060	Protein export	1/192	31/8846	0.494104337231372	0.768318876815556	0.589094395479114	HSPA5	1

Environmental Information Processing	Signal transduction	hsa04071	Sphingolipid signaling pathway	3/192	122/8846	0.496403554156552	0.768318876815556	0.589094395479114	FCER1G/GNA12/GNA13	3

Human Diseases	Cancer: specific types	hsa05220	Chronic myeloid leukemia	2/192	77/8846	0.500995142978119	0.770517846479006	0.590780415104111	CCND1/CDK6	2

Human Diseases	Drug resistance: antineoplastic	hsa01521	EGFR tyrosine kinase inhibitor resistance	2/192	80/8846	0.521443971425495	0.796923805386134	0.611026699386379	EGFR/MET	2

Organismal Systems	Environmental adaptation	hsa04710	Circadian rhythm	1/192	34/8846	0.526449965228659	0.797123700560485	0.611179965342022	BHLHE41	1

Metabolism	Xenobiotics biodegradation and metabolism	hsa00983	Drug metabolism - other enzymes	2/192	81/8846	0.528135455926906	0.797123700560485	0.611179965342022	HPRT1/TK1	2

Organismal Systems	Immune system	hsa04623	Cytosolic DNA-sensing pathway	2/192	83/8846	0.541328339727332	0.810395905130229	0.621356184570286	CCL5/CXCL10	2

Cellular Processes	Transport and catabolism	hsa04142	Lysosome	3/192	132/8846	0.549621594880488	0.810395905130229	0.621356184570286	LAMP3/LAPTM4B/PPT1	3

Environmental Information Processing	Signal transduction	hsa04068	FoxO signaling pathway	3/192	133/8846	0.554761689825742	0.810395905130229	0.621356184570286	CCND1/EGFR/IRS2	3

Human Diseases	Cancer: specific types	hsa05216	Thyroid cancer	1/192	37/8846	0.556737582218757	0.810395905130229	0.621356184570286	CCND1	1

Environmental Information Processing	Signal transduction	hsa04012	ErbB signaling pathway	2/192	86/8846	0.560636856846186	0.810395905130229	0.621356184570286	EGFR/PAK2	2

Organismal Systems	Endocrine system	hsa04911	Insulin secretion	2/192	86/8846	0.560636856846186	0.810395905130229	0.621356184570286	CREB3L1/ITPR3	2

Organismal Systems	Immune system	hsa04650	Natural killer cell mediated cytotoxicity	3/192	135/8846	0.564937348585005	0.810395905130229	0.621356184570286	FCER1G/FCGR3A/ICAM1	3

Metabolism	Metabolism of cofactors and vitamins	hsa00760	Nicotinate and nicotinamide metabolism	1/192	38/8846	0.566398881368944	0.810395905130229	0.621356184570286	NT5E	1

Human Diseases	Cancer: specific types	hsa05210	Colorectal cancer	2/192	87/8846	0.566943637333905	0.810395905130229	0.621356184570286	CCND1/EGFR	2

Organismal Systems	Endocrine system	hsa04910	Insulin signaling pathway	3/192	138/8846	0.579934453932256	0.824117381903732	0.631876875013907	IRS2/SOCS1/SOCS3	3

Cellular Processes	Cell growth and death	hsa04114	Oocyte meiosis	3/192	139/8846	0.584861235920826	0.826286513539307	0.633540016878741	ITPR3/YWHAG/YWHAQ	3

Human Diseases	Substance dependence	hsa05033	Nicotine addiction	1/192	41/8846	0.594143739036782	0.833122272092895	0.638781209272005	GRIN2D	1

Genetic Information Processing	Folding, sorting and degradation	hsa04120	Ubiquitin mediated proteolysis	3/192	142/8846	0.599421122715744	0.833122272092895	0.638781209272005	SOCS1/SOCS3/UBE2C	3

Metabolism	Glycan biosynthesis and metabolism	hsa00513	Various types of N-glycan biosynthesis	1/192	42/8846	0.602993756311619	0.833122272092895	0.638781209272005	RPN2	1

Organismal Systems	Endocrine system	hsa04912	GnRH signaling pathway	2/192	93/8846	0.603413662091973	0.833122272092895	0.638781209272005	EGFR/ITPR3	2

Genetic Information Processing	Replication and repair	hsa03410	Base excision repair	1/192	44/8846	0.620121939876316	0.843625045934607	0.64683401832543	PCNA	1

Organismal Systems	Excretory system	hsa04962	Vasopressin-regulated water reabsorption	1/192	44/8846	0.620121939876316	0.843625045934607	0.64683401832543	CREB3L1	1

Organismal Systems	Environmental adaptation	hsa04713	Circadian entrainment	2/192	97/8846	0.6264181584562	0.843625045934607	0.64683401832543	GRIN2D/ITPR3	2

Organismal Systems	Digestive system	hsa04970	Salivary secretion	2/192	97/8846	0.6264181584562	0.843625045934607	0.64683401832543	CST1/ITPR3	2

Organismal Systems	Endocrine system	hsa04925	Aldosterone synthesis and secretion	2/192	98/8846	0.632006105273949	0.843625045934607	0.64683401832543	CREB3L1/ITPR3	2

Metabolism	Metabolism of cofactors and vitamins	hsa00860	Porphyrin metabolism	1/192	46/8846	0.636514839158307	0.843625045934607	0.64683401832543	FTL	1

Organismal Systems	Sensory system	hsa04750	Inflammatory mediator regulation of TRP channels	2/192	99/8846	0.637529042580264	0.843625045934607	0.64683401832543	F2RL1/ITPR3	2

Environmental Information Processing	Signal transduction	hsa04010	MAPK signaling pathway	6/192	300/8846	0.638794273464887	0.843625045934607	0.64683401832543	CD14/EGFR/GNA12/JUND/MET/PAK2	6

Metabolism	Carbohydrate metabolism	hsa00620	Pyruvate metabolism	1/192	47/8846	0.644445424485762	0.84628259666824	0.648871646568241	GLO1	1

Environmental Information Processing	Signal transduction	hsa04020	Calcium signaling pathway	5/192	254/8846	0.650493327627151	0.84628259666824	0.648871646568241	EGFR/F2R/GRIN2D/ITPR3/MET	5

Organismal Systems	Endocrine system	hsa04921	Oxytocin signaling pathway	3/192	154/8846	0.654264065710218	0.84628259666824	0.648871646568241	CCND1/EGFR/ITPR3	3

Human Diseases	Cardiovascular disease	hsa05415	Diabetic cardiomyopathy	4/192	205/8846	0.654737152977897	0.84628259666824	0.648871646568241	COL1A1/COL1A2/COL3A1/MMP9	4

Human Diseases	Endocrine and metabolic disease	hsa04932	Non-alcoholic fatty liver disease	3/192	157/8846	0.667109847677284	0.853338080825026	0.654281309534458	CXCL8/IRS2/SOCS3	3

Metabolism	Lipid metabolism	hsa00565	Ether lipid metabolism	1/192	50/8846	0.667219075542202	0.853338080825026	0.654281309534458	PLA2G7	1

Organismal Systems	Endocrine system	hsa04922	Glucagon signaling pathway	2/192	107/8846	0.679397654667025	0.857196132067021	0.657239399505578	CREB3L1/ITPR3	2

Metabolism	Amino acid metabolism	hsa00270	Cysteine and methionine metabolism	1/192	52/8846	0.681589277648269	0.857196132067021	0.657239399505578	GCLM	1

Human Diseases	Infectious disease: viral	hsa05170	Human immunodeficiency virus 1 infection	4/192	213/8846	0.684112716935319	0.857196132067021	0.657239399505578	BST2/ITPR3/PAK2/TAPBP	4

Genetic Information Processing	Translation	hsa03013	Nucleocytoplasmic transport	2/192	108/8846	0.684345883214	0.857196132067021	0.657239399505578	CSE1L/KPNA2	2

Metabolism	Glycan biosynthesis and metabolism	hsa00510	N-Glycan biosynthesis	1/192	53/8846	0.688541160601593	0.858028215518909	0.657877384218526	RPN2	1

Environmental Information Processing	Signaling molecules and interaction	hsa04080	Neuroactive ligand-receptor interaction	7/192	368/8846	0.692919608445534	0.859078902307473	0.658682979027172	F2R/F2RL1/GAL/GRIN2D/NMB/NPW/NTS	7

Genetic Information Processing	Replication and repair	hsa03460	Fanconi anemia pathway	1/192	55/8846	0.701995167128184	0.862997379926148	0.661687399813421	UBE2T	1

Human Diseases	Neurodegenerative disease	hsa05012	Parkinson disease	5/192	271/8846	0.70576842213718	0.862997379926148	0.661687399813421	HSPA5/ITPR3/SLC39A6/TUBB/UCHL1	5

Metabolism	Global and overview maps	hsa01212	Fatty acid metabolism	1/192	57/8846	0.714870900900418	0.862997379926148	0.661687399813421	PPT1	1

Organismal Systems	Nervous system	hsa04725	Cholinergic synapse	2/192	115/8846	0.717258612016019	0.862997379926148	0.661687399813421	CREB3L1/ITPR3	2

Organismal Systems	Nervous system	hsa04726	Serotonergic synapse	2/192	115/8846	0.717258612016019	0.862997379926148	0.661687399813421	APP/ITPR3	2

Organismal Systems	Nervous system	hsa04724	Glutamatergic synapse	2/192	116/8846	0.721718794700723	0.862997379926148	0.661687399813421	GRIN2D/ITPR3	2

Organismal Systems	Endocrine system	hsa04923	Regulation of lipolysis in adipocytes	1/192	59/8846	0.727193092371116	0.862997379926148	0.661687399813421	IRS2	1

Environmental Information Processing	Signal transduction	hsa04024	cAMP signaling pathway	4/192	226/8846	0.728043057139343	0.862997379926148	0.661687399813421	CREB3L1/F2R/GRIN2D/SOX9	4

Human Diseases	Cancer: overview	hsa05208	Chemical carcinogenesis - reactive oxygen species	4/192	226/8846	0.728043057139343	0.862997379926148	0.661687399813421	AHR/EGFR/HIF1A/MET	4

Organismal Systems	Digestive system	hsa04978	Mineral absorption	1/192	61/8846	0.738985419697779	0.871715810614371	0.668372096507445	FTL	1

Organismal Systems	Aging	hsa04213	Longevity regulating pathway - multiple species	1/192	62/8846	0.74469000699636	0.874201312560944	0.670277809500919	IRS2	1

Genetic Information Processing	Replication and repair	hsa03420	Nucleotide excision repair	1/192	63/8846	0.750270553291521	0.876518002162691	0.672054088727729	PCNA	1

Genetic Information Processing	Information processing in viruses	hsa03250	Viral life cycle - HIV-1	1/192	64/8846	0.755729741925021	0.87867142242957	0.673705184189014	BST2	1

Genetic Information Processing	Translation	hsa00970	Aminoacyl-tRNA biosynthesis	1/192	66/8846	0.766294492065531	0.88304534399503	0.677058808261297	DARS2	1

Metabolism	Nucleotide metabolism	hsa00230	Purine metabolism	2/192	128/8846	0.770762761872398	0.88304534399503	0.677058808261297	HPRT1/NT5E	2

Metabolism	Metabolism of cofactors and vitamins	hsa00830	Retinol metabolism	1/192	68/8846	0.776404591243617	0.88304534399503	0.677058808261297	CYP2S1	1

Human Diseases	Substance dependence	hsa05034	Alcoholism	3/192	188/8846	0.779778846388648	0.88304534399503	0.677058808261297	CREB3L1/FOSB/GRIN2D	3

Organismal Systems	Immune system	hsa04664	Fc epsilon RI signaling pathway	1/192	69/8846	0.78129526320548	0.88304534399503	0.677058808261297	FCER1G	1

Organismal Systems	Endocrine system	hsa04924	Renin secretion	1/192	69/8846	0.78129526320548	0.88304534399503	0.677058808261297	ITPR3	1

Organismal Systems	Nervous system	hsa04728	Dopaminergic synapse	2/192	132/8846	0.785362580948606	0.883532903567182	0.677432635613564	CREB3L1/ITPR3	2

Human Diseases	Drug resistance: antineoplastic	hsa01524	Platinum drug resistance	1/192	75/8846	0.808483238301131	0.894666046675784	0.685968768731271	TOP2A	1

Organismal Systems	Endocrine system	hsa03320	PPAR signaling pathway	1/192	76/8846	0.812675601571702	0.894666046675784	0.685968768731271	MMP1	1

Organismal Systems	Digestive system	hsa04971	Gastric acid secretion	1/192	76/8846	0.812675601571702	0.894666046675784	0.685968768731271	ITPR3	1

Genetic Information Processing	Folding, sorting and degradation	hsa03018	RNA degradation	1/192	78/8846	0.820788392616745	0.894666046675784	0.685968768731271	XRN2	1

Human Diseases	Infectious disease: bacterial	hsa05100	Bacterial invasion of epithelial cells	1/192	78/8846	0.820788392616745	0.894666046675784	0.685968768731271	MET	1

Human Diseases	Neurodegenerative disease	hsa05014	Amyotrophic lateral sclerosis	6/192	371/8846	0.822340106721914	0.894666046675784	0.685968768731271	GRIN2D/HSPA5/ITPR3/NEFL/PFN2/TUBB	6

Human Diseases	Neurodegenerative disease	hsa05017	Spinocerebellar ataxia	2/192	144/8846	0.824381390115382	0.894666046675784	0.685968768731271	GRIN2D/ITPR3	2

Metabolism	Xenobiotics biodegradation and metabolism	hsa00980	Metabolism of xenobiotics by cytochrome P450	1/192	79/8846	0.824712734384262	0.894666046675784	0.685968768731271	CYP2S1	1

Organismal Systems	Sensory system	hsa04742	Taste transduction	1/192	86/8846	0.8498902446599	0.917881464232693	0.703768763999076	ITPR3	1

Genetic Information Processing	Transcription	hsa03040	Spliceosome	3/192	218/8846	0.857107008743315	0.921579659843476	0.706604287599286	RNU6-8/SF3B4/SNRPB	3

Organismal Systems	Digestive system	hsa04976	Bile secretion	1/192	90/8846	0.862626280861456	0.923428133256977	0.708021570658371	NCEH1	1

Genetic Information Processing	Translation	hsa03008	Ribosome biogenesis in eukaryotes	2/192	168/8846	0.883915637339784	0.936013179234806	0.717670923649819	GNL3L/XRN2	2

Environmental Information Processing	Signal transduction	hsa04070	Phosphatidylinositol signaling system	1/192	98/8846	0.884962417083593	0.936013179234806	0.717670923649819	ITPR3	1

Cellular Processes	Transport and catabolism	hsa04140	Autophagy - animal	2/192	169/8846	0.885938400098788	0.936013179234806	0.717670923649819	HIF1A/IRS2	2

Environmental Information Processing	Signal transduction	hsa04014	Ras signaling pathway	3/192	238/8846	0.894483780964532	0.940950470884767	0.721456501393129	EGFR/MET/PAK2	3

Cellular Processes	Transport and catabolism	hsa04137	Mitophagy - animal	1/192	105/8846	0.901519475725456	0.942467307640719	0.722619508132585	HIF1A	1

Organismal Systems	Digestive system	hsa04972	Pancreatic secretion	1/192	106/8846	0.903682644774845	0.942467307640719	0.722619508132585	ITPR3	1

Organismal Systems	Immune system	hsa04660	T cell receptor signaling pathway	1/192	122/8846	0.932513058225895	0.968378945080737	0.742486780503749	PAK2	1

Cellular Processes	Cell growth and death	hsa04210	Apoptosis	1/192	136/8846	0.950590511704729	0.981004337854081	0.752167068660049	ITPR3	1

Human Diseases	Infectious disease: bacterial	hsa05135	Yersinia infection	1/192	138/8846	0.952744953636062	0.981004337854081	0.752167068660049	CXCL8	1

Organismal Systems	Nervous system	hsa04723	Retrograde endocannabinoid signaling	1/192	149/8846	0.963027752116049	0.983116441768315	0.753786485566349	ITPR3	1

Metabolism	Global and overview maps	hsa01240	Biosynthesis of cofactors	1/192	153/8846	0.966186628037359	0.983116441768315	0.753786485566349	GCLM	1

Organismal Systems	Circulatory system	hsa04261	Adrenergic signaling in cardiomyocytes	1/192	154/8846	0.96693345507254	0.983116441768315	0.753786485566349	CREB3L1	1

Cellular Processes	Transport and catabolism	hsa04144	Endocytosis	2/192	252/8846	0.975275897555262	0.987466846274702	0.757122078365269	EGFR/TFRC	2

Organismal Systems	Immune system	hsa04613	Neutrophil extracellular trap formation	1/192	192/8846	0.985875828205373	0.994057370348156	0.762175241722433	FCGR3A	1

Human Diseases	Neurodegenerative disease	hsa05016	Huntington disease	2/192	311/8846	0.992168130226325	0.994630362645699	0.762614573048684	CREB3L1/TUBB	2

Organismal Systems	Environmental adaptation	hsa04714	Thermogenesis	1/192	235/8846	0.994630362645699	0.994630362645699	0.762614573048684	CREB3L1	1
