# Supplementary material for: Gene Expression Profiling and Clinical Validation Reveal CEBPB for Early Diagnosis of Esophageal Squamous Cell Carcinoma
Source: Cancer Rep (Hoboken). 2026 Nov 8;9(11):e70753. doi: 10.1002/cnr2.70753 (PMC13745642; doi:10.1002/cnr2.70753)
Supplement: Supplementary file 13 [file CNR2-9-e70753-s008.doc]

Doc. S3. GO enrichment analysis of the upregulated DEGs.

ONTOLOGY	ID	Description	GeneRatio	BgRatio	pvalue	p.adjust	qvalue	geneID	Count

BP	GO:0030198	extracellular matrix organization	31/349	321/18870	5.53350642198361e-14	8.79617293875295e-11	6.62482893678095e-11	APP/COL10A1/COL11A1/COL1A1/COL1A2/COL3A1/COL4A1/COL4A2/COL5A1/COL5A2/CREB3L1/FSCN1/LOX/LOXL2/LUM/MMP1/MMP10/MMP11/MMP12/MMP13/MMP3/MMP7/MMP9/OLFML2B/P4HA1/POSTN/PXDN/SERPINH1/SOX9/TGFBI/WDR72	31

BP	GO:0043062	extracellular structure organization	31/349	322/18870	6.02191605178706e-14	8.79617293875295e-11	6.62482893678095e-11	APP/COL10A1/COL11A1/COL1A1/COL1A2/COL3A1/COL4A1/COL4A2/COL5A1/COL5A2/CREB3L1/FSCN1/LOX/LOXL2/LUM/MMP1/MMP10/MMP11/MMP12/MMP13/MMP3/MMP7/MMP9/OLFML2B/P4HA1/POSTN/PXDN/SERPINH1/SOX9/TGFBI/WDR72	31

BP	GO:0045229	external encapsulating structure organization	31/349	323/18870	6.55127080840587e-14	8.79617293875295e-11	6.62482893678095e-11	APP/COL10A1/COL11A1/COL1A1/COL1A2/COL3A1/COL4A1/COL4A2/COL5A1/COL5A2/CREB3L1/FSCN1/LOX/LOXL2/LUM/MMP1/MMP10/MMP11/MMP12/MMP13/MMP3/MMP7/MMP9/OLFML2B/P4HA1/POSTN/PXDN/SERPINH1/SOX9/TGFBI/WDR72	31

BP	GO:0043588	skin development	30/349	317/18870	2.44508551437428e-13	2.4622011129749e-10	1.85440432958596e-10	CD109/CLDN1/CLIC4/COL1A1/COL1A2/COL3A1/COL5A1/COL5A2/EGFR/FOXE1/FOXQ1/FST/FZD6/GAL/GLI2/HOXC13/INHBA/ITGA2/ITGA6/ITGB4/KRT10/KRT14/KRT17/KRT75/MET/PLEC/S100A7/SOX9/SPRR2G/TP63	30

BP	GO:0001503	ossification	35/349	444/18870	5.20134850564866e-13	4.19020635615056e-10	3.1558497670062e-10	ASPN/CDK6/CEBPB/COL11A1/COL1A1/COL1A2/COL5A2/CREB3L1/CTHRC1/DDX21/DLX5/EGFR/EGR2/FZD1/GLI2/HIF1A/IGFBP3/ISG15/JUND/LOX/MMP13/PTHLH/RUNX3/SNAI2/SNX10/SOX9/SPP1/SRGN/STC1/TNC/TNFAIP6/TP63/TWSG1/VCAN/WNT7B	35

BP	GO:0032963	collagen metabolic process	18/349	106/18870	9.12842771601438e-13	6.12821780668432e-10	4.6154611715006e-10	COL1A1/COL1A2/COL5A1/CREB3L1/F2R/HIF1A/INHBA/ITGA2/MMP1/MMP10/MMP11/MMP12/MMP13/MMP3/MMP7/MMP9/SERPINH1/TRAM2	18

BP	GO:0008544	epidermis development	30/349	385/18870	3.4610713545582e-11	1.99159934516578e-08	1.49997107426116e-08	BNC1/CD109/CLDN1/CLIC4/EGFR/FOXE1/FOXQ1/FST/FZD6/GAL/GLI2/HLA-DRB1/HOXC13/INHBA/KRT10/KRT14/KRT17/KRT75/KRTDAP/LAMB3/LAMC2/MET/PLEC/PTHLH/S100A7/SFRP4/SOX9/SPRR2G/TP63/YBX1	30

BP	GO:0030199	collagen fibril organization	13/349	65/18870	1.63779081427064e-10	8.24627674985269e-08	6.21067516674736e-08	COL11A1/COL1A1/COL1A2/COL3A1/COL5A1/COL5A2/LOX/LOXL2/LUM/MMP11/P4HA1/PXDN/SERPINH1	13

BP	GO:0002460	adaptive immune response based on somatic recombination of immune receptors built from immunoglobulin superfamily domains	28/349	380/18870	5.77722395695384e-10	2.58562867762334e-07	1.94736367765392e-07	AHR/C1QA/C1QB/C1QC/CXCL13/FCER1G/FCGR3A/HLA-DRA/HLA-DRB1/HPRT1/ICAM1/IGHG2/IGHG3/IGHG4/IGHV1-18/IGHV3-15/IGHV3-21/IGHV3-23/IGHV3-48/IGHV4-34/IGHV4-39/IGHV5-51/IL27RA/RSAD2/SOCS3/TFRC/TNFAIP3/TREM2	28

BP	GO:0098773	skin epidermis development	16/349	128/18870	1.90258630854251e-09	7.66361765080922e-07	5.77184604338896e-07	CD109/CLDN1/EGFR/FOXE1/FOXQ1/FST/FZD6/GAL/GLI2/HOXC13/INHBA/KRT17/MET/PLEC/SOX9/TP63	16

BP	GO:0001649	osteoblast differentiation	22/349	257/18870	2.76768896145026e-09	1.0134773760656e-06	7.63299481999966e-07	CDK6/CEBPB/COL1A1/CREB3L1/CTHRC1/DDX21/DLX5/FZD1/GLI2/IGFBP3/JUND/LOX/PTHLH/SNAI2/SOX9/SPP1/TNC/TNFAIP6/TP63/TWSG1/VCAN/WNT7B	22

BP	GO:0002449	lymphocyte mediated immunity	26/349	368/18870	5.854659115838e-09	1.96521390988295e-06	1.48009890981097e-06	AHR/C1QA/C1QB/C1QC/FCER1G/FCGR3A/HLA-DRA/HLA-DRB1/HPRT1/ICAM1/IGHG2/IGHG3/IGHG4/IGHV1-18/IGHV3-15/IGHV3-21/IGHV3-23/IGHV3-48/IGHV4-34/IGHV4-39/IGHV5-51/IL27RA/RSAD2/TFRC/TREM2/TUBB	26

BP	GO:0016064	immunoglobulin mediated immune response	19/349	205/18870	9.34842555804286e-09	2.89657370367666e-06	2.18155161605502e-06	C1QA/C1QB/C1QC/FCER1G/FCGR3A/IGHG2/IGHG3/IGHG4/IGHV1-18/IGHV3-15/IGHV3-21/IGHV3-23/IGHV3-48/IGHV4-34/IGHV4-39/IGHV5-51/IL27RA/TFRC/TREM2	19

BP	GO:0019724	B cell mediated immunity	19/349	208/18870	1.18665840962987e-08	3.41418576713508e-06	2.57139062898743e-06	C1QA/C1QB/C1QC/FCER1G/FCGR3A/IGHG2/IGHG3/IGHG4/IGHV1-18/IGHV3-15/IGHV3-21/IGHV3-23/IGHV3-48/IGHV4-34/IGHV4-39/IGHV5-51/IL27RA/TFRC/TREM2	19

BP	GO:0002443	leukocyte mediated immunity	29/349	466/18870	1.32624741470708e-08	3.56141639096008e-06	2.68227722749881e-06	AHR/BST2/C1QA/C1QB/C1QC/DDX21/F2RL1/FCER1G/FCGR3A/HLA-DRA/HLA-DRB1/HPRT1/ICAM1/IGHG2/IGHG3/IGHG4/IGHV1-18/IGHV3-15/IGHV3-21/IGHV3-23/IGHV3-48/IGHV4-34/IGHV4-39/IGHV5-51/IL27RA/RSAD2/TFRC/TREM2/TUBB	29

BP	GO:0001942	hair follicle development	13/349	93/18870	1.62478573278408e-08	4.09039808228392e-06	3.08067926439718e-06	CD109/EGFR/FOXE1/FOXQ1/FST/FZD6/GAL/GLI2/HOXC13/INHBA/KRT17/SOX9/TP63	13

BP	GO:0071229	cellular response to acid chemical	13/349	94/18870	1.85446795542878e-08	4.39399819086303e-06	3.30933538547725e-06	CEBPB/COL1A1/COL1A2/COL3A1/COL4A1/COL5A2/EGFR/GCLM/PLEC/SESN3/SH3BP4/SLC7A5/SOCS1	13

BP	GO:0022404	molting cycle process	13/349	96/18870	2.40372181257822e-08	5.09589024266582e-06	3.83796468911381e-06	CD109/EGFR/FOXE1/FOXQ1/FST/FZD6/GAL/GLI2/HOXC13/INHBA/KRT17/SOX9/TP63	13

BP	GO:0022405	hair cycle process	13/349	96/18870	2.40372181257822e-08	5.09589024266582e-06	3.83796468911381e-06	CD109/EGFR/FOXE1/FOXQ1/FST/FZD6/GAL/GLI2/HOXC13/INHBA/KRT17/SOX9/TP63	13

BP	GO:0043589	skin morphogenesis	6/349	12/18870	3.22605448141921e-08	6.37724090484767e-06	4.80301267124105e-06	COL1A1/COL1A2/ITGA2/ITGA6/ITGB4/TP63	6

BP	GO:0042303	molting cycle	14/349	117/18870	3.48310079212137e-08	6.37724090484767e-06	4.80301267124105e-06	CD109/EGFR/FOXE1/FOXQ1/FST/FZD6/GAL/GLI2/HOXC13/INHBA/KRT14/KRT17/SOX9/TP63	14

BP	GO:0042633	hair cycle	14/349	117/18870	3.48310079212137e-08	6.37724090484767e-06	4.80301267124105e-06	CD109/EGFR/FOXE1/FOXQ1/FST/FZD6/GAL/GLI2/HOXC13/INHBA/KRT14/KRT17/SOX9/TP63	14

BP	GO:1904645	response to amyloid-beta	10/349	53/18870	3.96293182308413e-08	6.94029973190561e-06	5.22707986916635e-06	ABCC1/APP/GJA1/ICAM1/MMP12/MMP13/MMP3/MMP9/PRNP/TREM2	10

BP	GO:0071230	cellular response to amino acid stimulus	12/349	85/18870	5.20001854301762e-08	8.7273644546979e-06	6.57300589516525e-06	CEBPB/COL1A1/COL1A2/COL3A1/COL4A1/COL5A2/EGFR/GCLM/SESN3/SH3BP4/SLC7A5/SOCS1	12

BP	GO:0071559	response to transforming growth factor beta	21/349	293/18870	1.38684296398714e-07	2.23448138357609e-05	1.68289744093093e-05	ASPN/CD109/CHST11/CLDN1/COL1A1/COL1A2/COL3A1/COL4A2/HSP90AB1/HSPA5/HTRA3/LOX/MXRA5/PAK2/PMEPA1/POSTN/PTPRK/RUNX3/SOX9/TWSG1/ZNF703	21

BP	GO:0031214	biomineral tissue development	16/349	175/18870	1.69386322766955e-07	2.62418503117421e-05	1.97640235714318e-05	AMTN/ASPN/CEBPB/COL1A1/COL1A2/FAM83H/HIF1A/ISG15/LOX/MMP13/PTHLH/SNX10/SOX9/SPP1/SRGN/WDR72	16

BP	GO:0009615	response to virus	26/349	436/18870	1.77464960004741e-07	2.64751429221888e-05	1.99397276699284e-05	BST2/CCL5/CDK6/CXCL10/CXCL9/DDX21/DDX60/DTX3L/EIF2AK2/F2RL1/FOSL1/HIF1A/HSP90AA1/IFI6/IFIT3/ISG15/MMP12/NMB/OAS2/OAS3/ODC1/RSAD2/STAT1/TNFAIP3/TREM2/XPR1	26

BP	GO:0022617	extracellular matrix disassembly	10/349	62/18870	1.88534272322881e-07	2.71220017470202e-05	2.04269087531783e-05	FSCN1/MMP1/MMP10/MMP11/MMP12/MMP13/MMP3/MMP7/MMP9/WDR72	10

BP	GO:0048732	gland development	26/349	443/18870	2.41687449353546e-07	3.35695533102097e-05	2.52828758271114e-05	CCND1/CEBPB/CLDN1/EGFR/FOXE1/GLI2/HIF1A/HOXD13/IRS2/ITGA2/MET/MSN/MTCO2P12/OAS2/PCNA/PLXNA1/SEMA3C/SLC7A5/SNAI2/SOX9/TNC/TNFAIP3/TP63/TWSG1/WNT7B/ZNF703	26

BP	GO:0048736	appendage development	16/349	182/18870	2.90249762803845e-07	3.77137433733512e-05	2.84040684686139e-05	CHST11/COL3A1/DLX5/EN1/FZD6/GNA12/HOXC10/HOXC13/HOXD10/HOXD13/ITGA6/ITGB4/SEMA3C/SLC7A11/SOX9/TP63	16

BP	GO:0060173	limb development	16/349	182/18870	2.90249762803845e-07	3.77137433733512e-05	2.84040684686139e-05	CHST11/COL3A1/DLX5/EN1/FZD6/GNA12/HOXC10/HOXC13/HOXD10/HOXD13/ITGA6/ITGB4/SEMA3C/SLC7A11/SOX9/TP63	16

BP	GO:0051216	cartilage development	17/349	207/18870	3.27722549332375e-07	4.12520758972127e-05	3.10689601044705e-05	BGN/CHST11/COL11A1/COL1A1/COL3A1/HIF1A/LOXL2/MMP13/PTHLH/RUNX3/SERPINH1/SNAI2/SOX9/STC1/TGFBI/TWSG1/WNT7B	17

BP	GO:0001101	response to acid chemical	14/349	141/18870	3.67073917960808e-07	4.48052648953374e-05	3.37450408792041e-05	CEBPB/COL1A1/COL1A2/COL3A1/COL4A1/COL5A2/EGFR/GCLM/PCNA/PLEC/SESN3/SH3BP4/SLC7A5/SOCS1	14

BP	GO:0061138	morphogenesis of a branching epithelium	16/349	187/18870	4.1968953653303e-07	4.9720866269266e-05	3.74472211853929e-05	CLIC4/COL4A1/GLI2/GNA13/HOXB7/HOXD11/HOXD13/MET/NRARP/PLXNA1/SEMA3C/SNAI2/SOCS3/SOX9/TNC/TP63	16

BP	GO:0043200	response to amino acid	13/349	126/18870	6.17809033692899e-07	7.03982903439284e-05	5.30204026475727e-05	CEBPB/COL1A1/COL1A2/COL3A1/COL4A1/COL5A2/EGFR/GCLM/PCNA/SESN3/SH3BP4/SLC7A5/SOCS1	13

BP	GO:0019058	viral life cycle	21/349	321/18870	6.2918035064087e-07	7.03982903439284e-05	5.30204026475727e-05	BST2/CCL5/CLDN1/CXCL8/EGFR/EIF2AK2/HLA-DRB1/HSP90AB1/ICAM1/ISG15/ITGA2/ITGAV/KPNA2/LAMP3/NUCKS1/OAS2/OAS3/RSAD2/TFRC/TOP2A/XPR1	21

BP	GO:0001704	formation of primary germ layer	13/349	128/18870	7.41061320155291e-07	8.06755404752841e-05	6.07607034050512e-05	COL11A1/COL4A2/COL5A1/COL5A2/FZD7/GJA1/INHBA/ITGA2/ITGAV/ITGB4/LAMB3/MMP9/TWSG1	13

BP	GO:0001763	morphogenesis of a branching structure	16/349	203/18870	1.26050531110615e-06	0.000133613562977252	0.000100630922620718	CLIC4/COL4A1/GLI2/GNA13/HOXB7/HOXD11/HOXD13/MET/NRARP/PLXNA1/SEMA3C/SNAI2/SOCS3/SOX9/TNC/TP63	16

BP	GO:0030574	collagen catabolic process	8/349	45/18870	1.49961749895989e-06	0.000154883571431036	0.000116650408421117	MMP1/MMP10/MMP11/MMP12/MMP13/MMP3/MMP7/MMP9	8

BP	GO:0002062	chondrocyte differentiation	12/349	116/18870	1.62782880110413e-06	0.000160903825384244	0.000121184556723303	CHST11/COL11A1/COL3A1/HIF1A/LOXL2/PTHLH/RUNX3/SERPINH1/SNAI2/SOX9/TGFBI/TWSG1	12

BP	GO:0061448	connective tissue development	19/349	285/18870	1.63779961289821e-06	0.000160903825384244	0.000121184556723303	BGN/CHST11/COL11A1/COL1A1/COL3A1/COL5A1/HIF1A/LOX/LOXL2/MMP13/PTHLH/RUNX3/SERPINH1/SNAI2/SOX9/STC1/TGFBI/TWSG1/WNT7B	19

BP	GO:0071560	cellular response to transforming growth factor beta stimulus	19/349	287/18870	1.81680776347738e-06	0.000174240515983021	0.000131229072038642	ASPN/CD109/CHST11/CLDN1/COL1A1/COL1A2/COL3A1/COL4A2/HSP90AB1/HSPA5/HTRA3/LOX/PAK2/PMEPA1/POSTN/PTPRK/SOX9/TWSG1/ZNF703	19

BP	GO:0002573	myeloid leukocyte differentiation	17/349	235/18870	1.92565108629547e-06	0.000180384245944143	0.000135856216173893	APP/C1QC/CD109/CDK6/CEBPB/F2RL1/GLO1/HLA-DRB1/INHBA/MMP9/MYH9/SNX10/SOCS1/TFRC/TNFAIP6/TREM2/TRIB1	17

BP	GO:0006935	chemotaxis	25/349	468/18870	2.29772539288045e-06	0.000210346315511874	0.000158422119193336	ABCC1/ACKR3/CCL18/CCL5/CXCL10/CXCL13/CXCL14/CXCL8/CXCL9/F2RL1/FCER1G/FOSL1/GPNMB/ITGA2/ITGAV/LOX/MET/PLA2G7/PLAU/PLEC/S100A7/SEMA3C/SERPINE1/TNFAIP6/TREM2	25

BP	GO:0042330	taxis	25/349	470/18870	2.4787857424193e-06	0.000219042863701393	0.000164971915848904	ABCC1/ACKR3/CCL18/CCL5/CXCL10/CXCL13/CXCL14/CXCL8/CXCL9/F2RL1/FCER1G/FOSL1/GPNMB/ITGA2/ITGAV/LOX/MET/PLA2G7/PLAU/PLEC/S100A7/SEMA3C/SERPINE1/TNFAIP6/TREM2	25

BP	GO:0032964	collagen biosynthetic process	8/349	48/18870	2.50148255468324e-06	0.000219042863701393	0.000164971915848904	COL1A1/COL5A1/CREB3L1/F2R/INHBA/ITGA2/SERPINH1/TRAM2	8

BP	GO:0022612	gland morphogenesis	12/349	122/18870	2.78216616878935e-06	0.000238437560167734	0.000179579012283335	CEBPB/EGFR/HOXD13/MSN/PLXNA1/SEMA3C/SNAI2/SOX9/TNC/TNFAIP3/TP63/TWSG1	12

BP	GO:0035987	endodermal cell differentiation	8/349	49/18870	2.94185776318029e-06	0.000246870897293546	0.000185930571786965	COL11A1/COL4A2/COL5A1/COL5A2/INHBA/ITGAV/LAMB3/MMP9	8

BP	GO:0070141	response to UV-A	5/349	14/18870	3.67036961530928e-06	0.000301719363478893	0.000227239639770598	CCND1/EGFR/MMP1/MMP3/MMP9	5

BP	GO:0051607	defense response to virus	20/349	334/18870	4.50560245999807e-06	0.000362971334177445	0.000273371500836094	BST2/CXCL10/CXCL9/DDX21/DDX60/DTX3L/EIF2AK2/F2RL1/HSP90AA1/IFI6/IFIT3/ISG15/MMP12/NMB/OAS2/OAS3/RSAD2/STAT1/TNFAIP3/TREM2	20

BP	GO:0051402	neuron apoptotic process	18/349	278/18870	4.63706441994631e-06	0.000365000258810185	0.000274899583413723	APP/CCND1/CEBPB/EN1/F2R/FOXQ1/FZD1/GCLM/HIF1A/IL27RA/KIF14/MYBL2/NEFL/PIGT/PPT1/PRNP/TP63/TREM2	18

BP	GO:0140546	defense response to symbiont	20/349	335/18870	4.71201922992295e-06	0.000365000258810185	0.000274899583413723	BST2/CXCL10/CXCL9/DDX21/DDX60/DTX3L/EIF2AK2/F2RL1/HSP90AA1/IFI6/IFIT3/ISG15/MMP12/NMB/OAS2/OAS3/RSAD2/STAT1/TNFAIP3/TREM2	20

BP	GO:0050900	leukocyte migration	22/349	396/18870	5.0499248391452e-06	0.000383794287775035	0.000289054287714329	APP/CCL18/CCL5/CHST2/CXCL10/CXCL13/CXCL8/CXCL9/F2RL1/FCER1G/ICAM1/IL27RA/ITGA2/ITGA6/MSN/MYH9/PLA2G7/PLEC/S100A7/SERPINE1/TNFAIP6/TREM2	22

BP	GO:0033627	cell adhesion mediated by integrin	10/349	88/18870	5.1716950033859e-06	0.000385770138400711	0.000290542397656105	CCL5/CXCL13/ICAM1/ITGA2/ITGA6/ITGAV/ITGB4/PLAU/SERPINE1/SNAI2	10

BP	GO:0014910	regulation of smooth muscle cell migration	10/349	89/18870	5.73191054590584e-06	0.000413788584022203	0.000311644462225472	CCL5/GNA12/GNA13/IGFBP3/ITGA2/PLAU/PLXNA1/POSTN/SERPINE1/TRIB1	10

BP	GO:0030099	myeloid cell differentiation	23/349	430/18870	5.75277078084493e-06	0.000413788584022203	0.000311644462225472	APP/C1QC/CD109/CDK6/CEBPB/F2RL1/GLO1/HIF1A/HLA-DRB1/HOXB7/INHBA/ISG15/LOX/MFHAS1/MMP9/MYH9/SNX10/SOCS1/STAT1/TFRC/TNFAIP6/TREM2/TRIB1	23

BP	GO:0007162	negative regulation of cell adhesion	19/349	312/18870	6.14110193066433e-06	0.000433971203100279	0.000326844981794545	CEBPB/COL1A1/EPCAM/FZD7/GPNMB/HLA-DRB1/MMP12/NRARP/PLXNA1/POSTN/PRNP/RUNX3/SERPINE1/SNAI2/SOCS1/TGFBI/TNC/TWSG1/ZNF703	19

BP	GO:0042476	odontogenesis	12/349	132/18870	6.34177831815711e-06	0.000434963867657215	0.000327592606122431	AMTN/ASPN/COL1A1/COL1A2/FST/GLI2/INHBA/SERPINE1/SNX10/TNC/TP63/WDR72	12

BP	GO:0002685	regulation of leukocyte migration	16/349	230/18870	6.37111921344978e-06	0.000434963867657215	0.000327592606122431	APP/CCL5/CHST2/CXCL10/CXCL13/CXCL8/F2RL1/ICAM1/IL27RA/ITGA2/MSN/PLA2G7/S100A7/SERPINE1/TNFAIP6/TREM2	16

BP	GO:0001819	positive regulation of cytokine production	25/349	499/18870	7.07071356221813e-06	0.00047468057047691	0.000357505201514257	APP/CD14/CEBPB/DDX21/EIF2AK2/F2R/F2RL1/FCER1G/FCGR3A/HIF1A/HSP90AA1/IL27RA/ISG15/LUM/MMP12/NMB/OAS2/OAS3/PANX1/POSTN/RSAD2/SERPINE1/SLC7A5/STAT1/TREM2	25

BP	GO:0072678	T cell migration	9/349	73/18870	7.82514381366117e-06	0.000516716053793889	0.00038916418414101	APP/CCL5/CXCL10/CXCL13/ICAM1/IL27RA/MSN/PLEC/S100A7	9

BP	GO:0070661	leukocyte proliferation	20/349	350/18870	9.02526448791023e-06	0.000586351054150039	0.000441609715690276	AHR/BST2/CCL5/CEBPB/EFNB1/ELF4/F2RL1/FCGR3A/GAL/GPNMB/HLA-DRB1/HPRT1/IRS2/MSN/NMB/PRNP/TFRC/TNFAIP3/TREM2/TWSG1	20

BP	GO:0006958	complement activation, classical pathway	7/349	42/18870	1.07747224746278e-05	0.000680954377611665	0.000512860115056922	C1QA/C1QB/C1QC/IGHG2/IGHG3/IGHG4/TREM2	7

BP	GO:0001706	endoderm formation	8/349	58/18870	1.08195333086263e-05	0.000680954377611665	0.000512860115056922	COL11A1/COL4A2/COL5A1/COL5A2/INHBA/ITGAV/LAMB3/MMP9	8

BP	GO:0050673	epithelial cell proliferation	24/349	480/18870	1.11783585685019e-05	0.000692162901990393	0.000521301804091442	CCND1/CD109/CDK6/CEBPB/CLDN1/DLX5/EGFR/EPPK1/FST/FZD7/HIF1A/IGFBP3/IRS2/LOXL2/MMP12/NRARP/PTPRK/RUNX3/SNAI2/SOX9/STAT1/TNFAIP3/TP63/ZNF703	24

BP	GO:0014909	smooth muscle cell migration	10/349	96/18870	1.13412987913024e-05	0.000692162901990393	0.000521301804091442	CCL5/GNA12/GNA13/IGFBP3/ITGA2/PLAU/PLXNA1/POSTN/SERPINE1/TRIB1	10

BP	GO:0042060	wound healing	22/349	423/18870	1.42390375816473e-05	0.000852788109312696	0.000642276519897348	CD109/CLDN1/COL3A1/COL5A1/EPPK1/F2R/F2RL1/FCER1G/FZD6/FZD7/GNA12/GNA13/HIF1A/ITGA2/MMP12/MYH9/PLAU/PLEC/SERPINE1/SLC7A11/TNFAIP3/TNFRSF12A	22

BP	GO:1903900	regulation of viral life cycle	12/349	143/18870	1.43966215077615e-05	0.000852788109312696	0.000642276519897348	BST2/CCL5/CXCL8/EIF2AK2/HLA-DRB1/ISG15/KPNA2/LAMP3/OAS2/OAS3/RSAD2/TOP2A	12

BP	GO:1990868	response to chemokine	10/349	99/18870	1.491552218675e-05	0.000858281762403273	0.000646414059281407	ACKR3/CCL18/CCL5/CXCL10/CXCL13/CXCL8/CXCL9/HIF1A/LOX/TREM2	10

BP	GO:1990869	cellular response to chemokine	10/349	99/18870	1.491552218675e-05	0.000858281762403273	0.000646414059281407	ACKR3/CCL18/CCL5/CXCL10/CXCL13/CXCL8/CXCL9/HIF1A/LOX/TREM2	10

BP	GO:0050792	regulation of viral process	13/349	168/18870	1.52826826430212e-05	0.000867023178677317	0.000652997648290395	BST2/CCL5/CXCL8/EIF2AK2/HLA-DRB1/ISG15/KPNA2/LAMP3/OAS2/OAS3/RSAD2/STAT1/TOP2A	13

BP	GO:0035107	appendage morphogenesis	12/349	146/18870	1.77556711631799e-05	0.000979723882812171	0.000737878071986797	CHST11/COL3A1/DLX5/EN1/FZD6/GNA12/HOXC10/HOXD10/HOXD13/SEMA3C/SOX9/TP63	12

BP	GO:0035108	limb morphogenesis	12/349	146/18870	1.77556711631799e-05	0.000979723882812171	0.000737878071986797	CHST11/COL3A1/DLX5/EN1/FZD6/GNA12/HOXC10/HOXD10/HOXD13/SEMA3C/SOX9/TP63	12

BP	GO:0016032	viral process	22/349	432/18870	1.96867230526728e-05	0.00107159622238062	0.000807071633539161	BST2/CCL5/CLDN1/CXCL8/EGFR/EIF2AK2/HLA-DRB1/HSP90AB1/ICAM1/ISG15/ITGA2/ITGAV/KPNA2/LAMP3/NUCKS1/OAS2/OAS3/RSAD2/STAT1/TFRC/TOP2A/XPR1	22

BP	GO:0009410	response to xenobiotic stimulus	22/349	434/18870	2.11271419048574e-05	0.00113466836790354	0.000854574357470861	ABCC1/AHR/CA9/CCND1/COL1A1/CYP2S1/FOSB/FOSL1/FZD1/GAL/GCLM/GNA12/HSP90AA1/ITGA2/LOX/NCEH1/PCNA/PRNP/SEMA3C/STAT1/TFRC/UCHL1	22

BP	GO:0002687	positive regulation of leukocyte migration	12/349	149/18870	2.17790240201763e-05	0.00115428827306934	0.000869351069614239	APP/CCL5/CXCL10/CXCL13/CXCL8/F2RL1/ICAM1/ITGA2/PLA2G7/S100A7/SERPINE1/TREM2	12

BP	GO:0045069	regulation of viral genome replication	9/349	85/18870	2.7323017973512e-05	0.00142931319996502	0.0010764858209113	BST2/CCL5/CXCL8/EIF2AK2/ISG15/OAS2/OAS3/RSAD2/TOP2A	9

BP	GO:0031069	hair follicle morphogenesis	6/349	33/18870	2.78475400863275e-05	0.00143807553163753	0.00108308516233193	FOXE1/FOXQ1/FST/GLI2/KRT17/TP63	6

BP	GO:0043523	regulation of neuron apoptotic process	15/349	232/18870	2.93216473994551e-05	0.00148929471500971	0.0011216608395594	CCND1/CEBPB/EN1/F2R/FOXQ1/FZD1/GCLM/HIF1A/IL27RA/KIF14/MYBL2/NEFL/PPT1/PRNP/TREM2	15

BP	GO:0000302	response to reactive oxygen species	14/349	205/18870	2.95788424033707e-05	0.00148929471500971	0.0011216608395594	COL1A1/ECT2/EGFR/FOSL1/HIF1A/MET/MMP3/MMP9/PCNA/PTPRK/S100A7/SESN3/STAT1/TNFAIP3	14

BP	GO:0007584	response to nutrient	12/349	155/18870	3.22629854751569e-05	0.00160438648757941	0.0012083420940793	COL1A1/CXCL10/GCLM/ITGA2/POSTN/SLC16A1/SNAI2/SPP1/STAT1/STC1/TFRC/TNC	12

BP	GO:0009954	proximal/distal pattern formation	6/349	34/18870	3.32920897961973e-05	0.00162733552495148	0.00122562613884654	EN1/GLI2/HOXC10/HOXD10/IRX2/TP63	6

BP	GO:0090026	positive regulation of monocyte chemotaxis	5/349	21/18870	3.35324847494967e-05	0.00162733552495148	0.00122562613884654	CCL5/CXCL10/PLA2G7/S100A7/SERPINE1	5

BP	GO:0035878	nail development	4/349	11/18870	3.42491562163237e-05	0.00164232858618276	0.00123691814806322	FZD6/HOXC13/ITGA6/ITGB4	4

BP	GO:1901654	response to ketone	14/349	208/18870	3.47231302084341e-05	0.00164546786446556	0.00123928249239266	AHR/CA9/CLDN1/DSG2/FOSB/FOSL1/HOXD13/INHBA/MSN/NEFL/PCNA/POSTN/SPP1/TNC	14

BP	GO:0030879	mammary gland development	11/349	133/18870	3.74114794218885e-05	0.00173743798642556	0.00130854969865637	CCND1/CEBPB/GLI2/HIF1A/IRS2/ITGA2/MTCO2P12/OAS2/SOX9/WNT7B/ZNF703	11

BP	GO:0014812	muscle cell migration	10/349	110/18870	3.75265900742363e-05	0.00173743798642556	0.00130854969865637	CCL5/GNA12/GNA13/IGFBP3/ITGA2/PLAU/PLXNA1/POSTN/SERPINE1/TRIB1	10

BP	GO:0060326	cell chemotaxis	18/349	325/18870	3.81947158437941e-05	0.0017482763115773	0.00131671257250974	ABCC1/ACKR3/CCL18/CCL5/CXCL10/CXCL13/CXCL14/CXCL8/CXCL9/F2RL1/FCER1G/LOX/MET/PLA2G7/PLEC/S100A7/SERPINE1/TNFAIP6	18

BP	GO:0070098	chemokine-mediated signaling pathway	9/349	91/18870	4.71999849894035e-05	0.00212199752186813	0.00159818033189357	ACKR3/CCL18/CCL5/CXCL10/CXCL13/CXCL8/CXCL9/HIF1A/TREM2	9

BP	GO:0002064	epithelial cell development	14/349	214/18870	4.74130528719293e-05	0.00212199752186813	0.00159818033189357	CDK6/CLDN1/CLIC4/F2RL1/HIF1A/ICAM1/MET/MSN/PLEC/SIPA1L3/SOX9/STC1/TP63/WNT7B	14

BP	GO:0034135	regulation of toll-like receptor 2 signaling pathway	4/349	12/18870	5.06252188381395e-05	0.00224086133494534	0.00168770249498575	F2RL1/MFHAS1/TNFAIP3/TREM2	4

BP	GO:0043524	negative regulation of neuron apoptotic process	12/349	163/18870	5.28834043660178e-05	0.0023153733998513	0.00174382118287029	CCND1/CEBPB/EN1/F2R/FOXQ1/FZD1/GCLM/HIF1A/IL27RA/KIF14/NEFL/PPT1	12

BP	GO:0032481	positive regulation of type I interferon production	8/349	72/18870	5.39714465674532e-05	0.00233760200831937	0.00176056263732202	CD14/HSP90AA1/ISG15/MMP12/NMB/OAS2/OAS3/STAT1	8

BP	GO:0048730	epidermis morphogenesis	6/349	37/18870	5.49218700560558e-05	0.00235346055942333	0.00177250648937909	FOXE1/FOXQ1/FST/GLI2/KRT17/TP63	6

BP	GO:1902074	response to salt	19/349	366/18870	5.61573807213625e-05	0.00238107294258577	0.00179330272840961	AGRN/ASPN/CCNA2/CLDN1/CLIC4/COL1A1/ECT2/EN1/FOSB/GCLM/HSP90AA1/ITGA2/ITPR3/JUND/NEFL/NT5E/PANX1/PCNA/PLEC	19

BP	GO:0070374	positive regulation of ERK1 and ERK2 cascade	14/349	218/18870	5.79758364426508e-05	0.00243256947073955	0.00183208728758464	ACKR3/APP/CCL18/CCL5/EGFR/F2R/F2RL1/GPNMB/HLA-DRB1/ICAM1/INHBA/MFHAS1/S100A7/TREM2	14

BP	GO:0031099	regeneration	13/349	191/18870	5.87443251398985e-05	0.00243940352230424	0.0018372343467519	CCNA2/CCND1/CEBPB/CLDN1/EPPK1/FZD7/NEFL/PCNA/POSTN/PTGFRN/SLC7A5/SPP1/TNC	13

BP	GO:0006979	response to oxidative stress	20/349	400/18870	6.05484533909392e-05	0.00248866500263983	0.00187433558187634	ABCC1/APP/COL1A1/ECT2/EGFR/FOSL1/GCLM/HIF1A/MET/MMP3/MMP9/PCNA/PRNP/PTPRK/PXDN/S100A7/SESN3/SLC7A11/STAT1/TNFAIP3	20

BP	GO:0002455	humoral immune response mediated by circulating immunoglobulin	7/349	55/18870	6.59608352171329e-05	0.00265690244254611	0.00200104344311344	C1QA/C1QB/C1QC/IGHG2/IGHG3/IGHG4/TREM2	7

BP	GO:0045071	negative regulation of viral genome replication	7/349	55/18870	6.59608352171329e-05	0.00265690244254611	0.00200104344311344	BST2/CCL5/EIF2AK2/ISG15/OAS2/OAS3/RSAD2	7

BP	GO:0044060	regulation of endocrine process	6/349	39/18870	7.47163674238689e-05	0.00297977750478558	0.00224421647645222	F2R/F2RL1/GAL/GJA1/INHBA/SPP1	6

BP	GO:0050678	regulation of epithelial cell proliferation	20/349	407/18870	7.68364668938937e-05	0.00303428714361376	0.00228527035694738	CCND1/CD109/CDK6/CLDN1/DLX5/EGFR/EPPK1/FZD7/HIF1A/IRS2/MMP12/NRARP/PTPRK/RUNX3/SNAI2/SOX9/STAT1/TNFAIP3/TP63/ZNF703	20

BP	GO:0007369	gastrulation	13/349	197/18870	8.05772739636675e-05	0.0031511190245209	0.00237326217233817	COL11A1/COL4A2/COL5A1/COL5A2/FZD7/GJA1/INHBA/ITGA2/ITGAV/ITGB4/LAMB3/MMP9/TWSG1	13

BP	GO:0050921	positive regulation of chemotaxis	11/349	145/18870	8.24117153191383e-05	0.0031918691279374	0.00240395307236596	CCL5/CXCL10/CXCL13/CXCL8/F2RL1/ITGA2/MET/PLA2G7/S100A7/SERPINE1/TREM2	11

BP	GO:0007178	transmembrane receptor protein serine/threonine kinase signaling pathway	20/349	410/18870	8.49339330506077e-05	0.00325822745074141	0.00245393077746217	ASPN/CD109/CHST11/COL1A2/COL3A1/DLX5/FST/FZD1/HSP90AB1/HSPA5/HTRA3/INHBA/LOX/NCEH1/PMEPA1/PTPRK/SFRP4/TNFAIP6/TWSG1/ZNF703	20

BP	GO:0030282	bone mineralization	10/349	123/18870	9.71430857305781e-05	0.00369143725776197	0.00278020231455339	ASPN/COL1A2/HIF1A/ISG15/LOX/MMP13/PTHLH/SNX10/SOX9/SRGN	10

BP	GO:0006959	humoral immune response	15/349	258/18870	9.82226411950575e-05	0.0036975775582588	0.002784826875791	C1QA/C1QB/C1QC/CXCL10/CXCL13/CXCL14/CXCL8/CXCL9/HLA-DRB1/IGHG2/IGHG3/IGHG4/IGKV3-20/S100A7/TREM2	15

BP	GO:0098883	synapse pruning	4/349	14/18870	9.94165668239978e-05	0.00370786973302836	0.00279257841702497	C1QA/C1QB/C1QC/TREM2	4

BP	GO:0072676	lymphocyte migration	10/349	124/18870	0.000103959630335562	0.00384173753203343	0.00289340081725823	APP/CCL18/CCL5/CXCL10/CXCL13/ICAM1/IL27RA/MSN/PLEC/S100A7	10

BP	GO:1903706	regulation of hemopoiesis	20/349	417/18870	0.000106840538331798	0.00391230625818622	0.00294654958346644	C1QC/CDK6/CEBPB/GLI2/HIF1A/HLA-DRA/HLA-DRB1/INHBA/ISG15/LOX/NRARP/RUNX3/SNAI2/SOCS1/SOX12/SOX4/STAT1/TNFAIP6/TREM2/TRIB1	20

BP	GO:0010818	T cell chemotaxis	5/349	27/18870	0.000121439842613796	0.00436749719686044	0.00328937618809172	CCL5/CXCL10/CXCL13/PLEC/S100A7	5

BP	GO:0150146	cell junction disassembly	5/349	27/18870	0.000121439842613796	0.00436749719686044	0.00328937618809172	C1QA/C1QB/C1QC/SNAI2/TREM2	5

BP	GO:0090287	regulation of cellular response to growth factor stimulus	18/349	357/18870	0.000126773364629895	0.00451896559937361	0.00340345446542485	ASPN/CD109/CHST11/CREB3L1/CXCL13/FST/FZD1/HIF1A/HSP90AB1/HSPA5/HTRA3/LOX/PMEPA1/SFRP4/SHISA2/TNFAIP6/TWSG1/ZNF703	18

BP	GO:0060350	endochondral bone morphogenesis	7/349	61/18870	0.000128978087308587	0.0045488710769837	0.00342597774626745	COL1A1/COL3A1/DLX5/MMP13/SERPINH1/SOX9/STC1	7

BP	GO:0032506	cytokinetic process	6/349	43/18870	0.000131197267383331	0.0045488710769837	0.00342597774626745	ANLN/CEP55/ECT2/KIF20A/MYH9/PLEC	6

BP	GO:1904646	cellular response to amyloid-beta	6/349	43/18870	0.000131197267383331	0.0045488710769837	0.00342597774626745	ABCC1/APP/GJA1/ICAM1/PRNP/TREM2	6

BP	GO:0071216	cellular response to biotic stimulus	15/349	265/18870	0.000132129571997789	0.0045488710769837	0.00342597774626745	AHR/CD14/CXCL10/CXCL13/CXCL8/CXCL9/HSPA5/MMP3/MMP9/MTDH/SERPINE1/SLC7A5/TNFAIP3/TREM2/TRIB1	15

BP	GO:0038065	collagen-activated signaling pathway	4/349	15/18870	0.000133596338037984	0.00456039025099152	0.00343465340076244	COL1A1/COL4A1/COL4A2/ITGA2	4

BP	GO:0070663	regulation of leukocyte proliferation	15/349	267/18870	0.000143518390674028	0.00485791661878139	0.00365873508998274	AHR/BST2/CCL5/CEBPB/EFNB1/FCGR3A/GAL/GPNMB/HLA-DRB1/IRS2/NMB/PRNP/TFRC/TNFAIP3/TWSG1	15

BP	GO:0007492	endoderm development	8/349	83/18870	0.000149156093066553	0.00500667285726731	0.00377077070366497	COL11A1/COL4A2/COL5A1/COL5A2/INHBA/ITGAV/LAMB3/MMP9	8

BP	GO:0071219	cellular response to molecule of bacterial origin	14/349	239/18870	0.000154177509730027	0.00513245462142602	0.0038655031147624	AHR/CD14/CXCL10/CXCL13/CXCL8/CXCL9/MMP3/MMP9/MTDH/SERPINE1/SLC7A5/TNFAIP3/TREM2/TRIB1	14

BP	GO:0000768	syncytium formation by plasma membrane fusion	7/349	63/18870	0.000158483199692884	0.00519000266961736	0.00390884537026008	ADAM12/CD109/CXCL10/CXCL9/MYH9/PTGFRN/TREM2	7

BP	GO:0140253	cell-cell fusion	7/349	63/18870	0.000158483199692884	0.00519000266961736	0.00390884537026008	ADAM12/CD109/CXCL10/CXCL9/MYH9/PTGFRN/TREM2	7

BP	GO:0019079	viral genome replication	10/349	131/18870	0.000163985665771955	0.00531633293901576	0.00400399088753552	BST2/CCL5/CXCL8/EIF2AK2/ISG15/NUCKS1/OAS2/OAS3/RSAD2/TOP2A	10

BP	GO:2001233	regulation of apoptotic signaling pathway	19/349	398/18870	0.000168109123193692	0.00531633293901576	0.00400399088753552	ACKR3/CREB3L1/FZD1/GCLM/HIF1A/ICAM1/IFI6/INHBA/ITGA6/ITGAV/MAGEA3/MMP9/PAK2/SERPINE1/SNAI2/TNFAIP3/TNFRSF12A/TP63/TREM2	19

BP	GO:0000070	mitotic sister chromatid segregation	12/349	184/18870	0.000168252277577294	0.00531633293901576	0.00400399088753552	DLGAP5/KIF11/KIF14/KIF4A/MAPRE1/MISP/MYBL2/MZT1/NCAPD2/TPX2/TRIP13/UBE2C	12

BP	GO:0071604	transforming growth factor beta production	6/349	45/18870	0.00016992386887672	0.00531633293901576	0.00400399088753552	COL3A1/HIF1A/HSP90AB1/ITGAV/LAPTM4B/LUM	6

BP	GO:0090025	regulation of monocyte chemotaxis	5/349	29/18870	0.000173301419271246	0.00531633293901576	0.00400399088753552	CCL5/CXCL10/PLA2G7/S100A7/SERPINE1	5

BP	GO:0097421	liver regeneration	5/349	29/18870	0.000173301419271246	0.00531633293901576	0.00400399088753552	CCND1/CEBPB/CLDN1/PCNA/SLC7A5	5

BP	GO:0060562	epithelial tube morphogenesis	17/349	334/18870	0.000173833662222739	0.00531633293901576	0.00400399088753552	CLIC4/COL4A1/CTHRC1/CXCL10/FZD1/FZD2/FZD6/GLI2/GNA13/HIF1A/HOXB7/HOXD11/IRX2/MET/NRARP/SOX9/TNC	17

BP	GO:0001885	endothelial cell development	7/349	64/18870	0.000175159585189249	0.00531633293901576	0.00400399088753552	CLDN1/CLIC4/F2RL1/ICAM1/MET/MSN/STC1	7

BP	GO:0030225	macrophage differentiation	7/349	64/18870	0.000175159585189249	0.00531633293901576	0.00400399088753552	APP/C1QC/HLA-DRB1/INHBA/MMP9/SOCS1/TRIB1	7

BP	GO:0010819	regulation of T cell chemotaxis	4/349	16/18870	0.000175539295156181	0.00531633293901576	0.00400399088753552	CCL5/CXCL10/CXCL13/S100A7	4

BP	GO:2001236	regulation of extrinsic apoptotic signaling pathway	11/349	158/18870	0.000177031898853574	0.0053215260341955	0.00400790206202672	GCLM/ICAM1/IFI6/INHBA/ITGA6/ITGAV/PAK2/SERPINE1/SNAI2/TNFAIP3/TNFRSF12A	11

BP	GO:2001234	negative regulation of apoptotic signaling pathway	14/349	243/18870	0.000183244913724703	0.00546039952352209	0.00411249449296782	ACKR3/CREB3L1/FZD1/GCLM/HIF1A/ICAM1/IFI6/ITGA6/ITGAV/MAGEA3/MMP9/SERPINE1/SNAI2/TNFAIP3	14

BP	GO:0002244	hematopoietic progenitor cell differentiation	10/349	133/18870	0.00018571865310887	0.00546039952352209	0.00411249449296782	ANLN/CDK6/EIF2AK2/FST/INHBA/KRT75/MLF1/SIPA1L3/SOX4/TOP2A	10

BP	GO:0019730	antimicrobial humoral response	10/349	133/18870	0.00018571865310887	0.00546039952352209	0.00411249449296782	CXCL10/CXCL13/CXCL14/CXCL8/CXCL9/IGHG2/IGHG3/IGHG4/IGKV3-20/S100A7	10

BP	GO:0002237	response to molecule of bacterial origin	18/349	369/18870	0.000190884466007881	0.00553570975769448	0.00416921432124483	AHR/CD14/CEBPB/CLDN1/CXCL10/CXCL13/CXCL8/CXCL9/F2R/MMP3/MMP9/MTDH/S100A7/SERPINE1/SLC7A5/TNFAIP3/TREM2/TRIB1	18

BP	GO:0033273	response to vitamin	8/349	86/18870	0.000191259248352201	0.00553570975769448	0.00416921432124483	COL1A1/CXCL10/ITGA2/POSTN/SNAI2/SPP1/STC1/TNC	8

BP	GO:0035272	exocrine system development	6/349	46/18870	0.00019240302037667	0.00553570975769448	0.00416921432124483	EGFR/PLXNA1/SEMA3C/SNAI2/SOX9/TWSG1	6

BP	GO:0050679	positive regulation of epithelial cell proliferation	13/349	218/18870	0.000221273221648251	0.00630758922987712	0.00475055458122246	CCND1/CLDN1/DLX5/EGFR/FZD7/HIF1A/IRS2/MMP12/NRARP/SOX9/TNFAIP3/TP63/ZNF703	13

BP	GO:0007389	pattern specification process	21/349	475/18870	0.000222362877518011	0.00630758922987712	0.00475055458122246	BASP1/BHLHE41/C1QA/EFNB1/EGR2/EN1/FST/GLI2/HIF1A/HOXB7/HOXC10/HOXC13/HOXD10/HOXD11/HOXD13/IRX2/NRARP/SEMA3C/STC1/TP63/WNT7B	21

BP	GO:0034134	toll-like receptor 2 signaling pathway	4/349	17/18870	0.000226216788986551	0.00637203654571906	0.00479909301331792	F2RL1/MFHAS1/TNFAIP3/TREM2	4

BP	GO:0006949	syncytium formation	7/349	67/18870	0.000233874129241247	0.00649686201781891	0.00489310519399835	ADAM12/CD109/CXCL10/CXCL9/MYH9/PTGFRN/TREM2	7

BP	GO:0006956	complement activation	7/349	67/18870	0.000233874129241247	0.00649686201781891	0.00489310519399835	C1QA/C1QB/C1QC/IGHG2/IGHG3/IGHG4/TREM2	7

BP	GO:0034143	regulation of toll-like receptor 4 signaling pathway	5/349	31/18870	0.000240551024228862	0.00663657209310861	0.00499832769882899	CD14/F2RL1/MFHAS1/TNFAIP3/TREM2	5

BP	GO:0032102	negative regulation of response to external stimulus	21/349	481/18870	0.000263263805291439	0.00721378644703344	0.00543305611779396	AHR/CD109/CXCL13/ELF4/EPPK1/F2RL1/HLA-DRB1/ISG15/MFHAS1/MMP12/NT5E/OAS3/PLAU/SEMA3C/SERPINE1/SOCS3/SPP1/TNFAIP3/TNFAIP6/TREM2/TRIB1	21

BP	GO:2000404	regulation of T cell migration	6/349	49/18870	0.000274181363987363	0.00745851175350809	0.00561737073998074	APP/CCL5/CXCL10/CXCL13/IL27RA/S100A7	6

BP	GO:0007435	salivary gland morphogenesis	5/349	32/18870	0.000280811909428725	0.00745851175350809	0.00561737073998074	EGFR/PLXNA1/SEMA3C/SNAI2/TWSG1	5

BP	GO:0050918	positive chemotaxis	7/349	69/18870	0.000281142163896483	0.00745851175350809	0.00561737073998074	CCL5/CXCL10/CXCL8/F2RL1/GPNMB/ITGA2/MET	7

BP	GO:2000401	regulation of lymphocyte migration	7/349	69/18870	0.000281142163896483	0.00745851175350809	0.00561737073998074	APP/CCL5/CXCL10/CXCL13/IL27RA/MSN/S100A7	7

BP	GO:0032496	response to lipopolysaccharide	17/349	348/18870	0.000281453273717286	0.00745851175350809	0.00561737073998074	CD14/CEBPB/CLDN1/CXCL10/CXCL13/CXCL8/CXCL9/F2R/MMP3/MMP9/MTDH/S100A7/SERPINE1/SLC7A5/TNFAIP3/TREM2/TRIB1	17

BP	GO:0036003	positive regulation of transcription from RNA polymerase II promoter in response to stress	4/349	18/18870	0.000286627651785242	0.00754598811366638	0.00568325347399428	CEBPB/CREB3L1/HIF1A/HSPA5	4

BP	GO:0000819	sister chromatid segregation	13/349	225/18870	0.000300824462915405	0.00786831777028086	0.00592601573562678	DLGAP5/KIF11/KIF14/KIF4A/MAPRE1/MISP/MYBL2/MZT1/NCAPD2/TOP2A/TPX2/TRIP13/UBE2C	13

BP	GO:0051250	negative regulation of lymphocyte activation	11/349	169/18870	0.000317574621589646	0.00825284242427803	0.00621562009793793	CEBPB/GAL/GPNMB/HLA-DRB1/INHBA/NRARP/PRNP/RUNX3/SOCS1/TNFAIP3/TWSG1	11

BP	GO:0007159	leukocyte cell-cell adhesion	19/349	419/18870	0.000321516412856225	0.00830171866015944	0.00625243118658326	CCL5/CEBPB/CHST2/EFNB1/GLI2/GPNMB/HLA-DRA/HLA-DRB1/ICAM1/MSN/NRARP/NT5E/PRNP/RUNX3/SOCS1/SOX12/SOX4/TFRC/TWSG1	19

BP	GO:0002063	chondrocyte development	5/349	33/18870	0.000325984118239866	0.00836346514821772	0.00629893549290845	CHST11/COL11A1/PTHLH/SERPINH1/SOX9	5

BP	GO:0048525	negative regulation of viral process	8/349	93/18870	0.000328289486540587	0.00836930412522458	0.00630333311265804	BST2/CCL5/EIF2AK2/ISG15/OAS2/OAS3/RSAD2/STAT1	8

BP	GO:0031334	positive regulation of protein-containing complex assembly	12/349	198/18870	0.000331004349801315	0.00838544352829999	0.00631548848809925	CXCL13/FSCN1/GNL3L/HSP90AA1/ISG15/MAPRE1/MET/MMP1/MMP3/MSN/PFN2/TFRC	12

BP	GO:0003158	endothelium development	10/349	143/18870	0.000334179578381625	0.00841297088575742	0.00633622069010424	CLDN1/CLIC4/CXCL10/F2RL1/FZD1/FZD2/ICAM1/MET/MSN/STC1	10

BP	GO:1903707	negative regulation of hemopoiesis	9/349	118/18870	0.000348284435227473	0.0087136006527718	0.00656263970137677	C1QC/CDK6/INHBA/NRARP/RUNX3/SNAI2/SOCS1/TNFAIP6/TRIB1	9

BP	GO:0051897	positive regulation of phosphatidylinositol 3-kinase/protein kinase B signal transduction	11/349	171/18870	0.000351237389161844	0.00872695621452254	0.00657269842948151	CCL5/EGFR/F2R/F2RL1/HSP90AA1/HSP90AB1/MFHAS1/MTDH/NTS/SOX9/TREM2	11

BP	GO:0045104	intermediate filament cytoskeleton organization	8/349	94/18870	0.00035315140590049	0.00872695621452254	0.00657269842948151	EPPK1/FAM83H/KRT10/KRT14/KRT17/KRT75/NEFL/PLEC	8

BP	GO:0007179	transforming growth factor beta receptor signaling pathway	13/349	229/18870	0.000356394012056175	0.00875338463757482	0.0065926029701277	ASPN/CD109/CHST11/COL1A2/COL3A1/HSP90AB1/HSPA5/HTRA3/LOX/PMEPA1/PTPRK/TWSG1/ZNF703	13

BP	GO:0007431	salivary gland development	5/349	34/18870	0.000376448261074983	0.00913453973259055	0.0068796695524293	EGFR/PLXNA1/SEMA3C/SNAI2/TWSG1	5

BP	GO:0060351	cartilage development involved in endochondral bone morphogenesis	5/349	34/18870	0.000376448261074983	0.00913453973259055	0.0068796695524293	COL1A1/MMP13/SERPINH1/SOX9/STC1	5

BP	GO:0045103	intermediate filament-based process	8/349	95/18870	0.00037952585031203	0.00915407260513088	0.006894380716037	EPPK1/FAM83H/KRT10/KRT14/KRT17/KRT75/NEFL/PLEC	8

BP	GO:0030326	embryonic limb morphogenesis	9/349	120/18870	0.000394427612776974	0.00939052359818233	0.00707246354726428	CHST11/DLX5/EN1/FZD6/GNA12/HOXC10/HOXD10/HOXD13/TP63	9

BP	GO:0035113	embryonic appendage morphogenesis	9/349	120/18870	0.000394427612776974	0.00939052359818233	0.00707246354726428	CHST11/DLX5/EN1/FZD6/GNA12/HOXC10/HOXD10/HOXD13/TP63	9

BP	GO:0045109	intermediate filament organization	7/349	73/18870	0.00039865430369642	0.00939052359818233	0.00707246354726428	EPPK1/KRT10/KRT14/KRT17/KRT75/NEFL/PLEC	7

BP	GO:0071677	positive regulation of mononuclear cell migration	7/349	73/18870	0.00039865430369642	0.00939052359818233	0.00707246354726428	APP/CCL5/CXCL10/CXCL13/PLA2G7/S100A7/SERPINE1	7

BP	GO:0050920	regulation of chemotaxis	13/349	232/18870	0.000403602189198421	0.00943295404760998	0.00710441999822609	CCL5/CXCL10/CXCL13/CXCL8/F2RL1/ITGA2/MET/PLA2G7/S100A7/SEMA3C/SERPINE1/TNFAIP6/TREM2	13

BP	GO:0001667	ameboidal-type cell migration	21/349	497/18870	0.000406283789472089	0.00943295404760998	0.00710441999822609	ANLN/CXCL13/EFNB1/EPPK1/GJA1/GNA12/GNA13/HIF1A/IRS2/ITGA2/ITGB4/LOXL2/MET/MMP9/MYH9/PFN2/PLEC/SEMA3C/SNAI2/SOX9/STC1	21

BP	GO:0002690	positive regulation of leukocyte chemotaxis	8/349	96/18870	0.00040748113313906	0.00943295404760998	0.00710441999822609	CCL5/CXCL10/CXCL13/CXCL8/F2RL1/PLA2G7/S100A7/SERPINE1	8

BP	GO:2000406	positive regulation of T cell migration	5/349	35/18870	0.000432596451767338	0.00995713432982193	0.00749920585860733	APP/CCL5/CXCL10/CXCL13/S100A7	5

BP	GO:0090092	regulation of transmembrane receptor protein serine/threonine kinase signaling pathway	15/349	297/18870	0.000449277248750608	0.0102823224884514	0.00774412099820127	ASPN/CD109/CHST11/FST/FZD1/HSP90AB1/HSPA5/HTRA3/INHBA/LOX/PMEPA1/SFRP4/TNFAIP6/TWSG1/ZNF703	15

BP	GO:0060993	kidney morphogenesis	8/349	98/18870	0.000468416864556844	0.0106437160521885	0.00801630420279287	BASP1/HOXB7/HOXD11/IRX2/SOX4/SOX9/STAT1/WNT7B	8

BP	GO:0002065	columnar/cuboidal epithelial cell differentiation	9/349	123/18870	0.000472995326053063	0.0106437160521885	0.00801630420279287	CDK6/CEBPB/FST/HIF1A/SERPINE1/SOX4/SOX9/TP63/TUBB	9

BP	GO:0071774	response to fibroblast growth factor	9/349	123/18870	0.000472995326053063	0.0106437160521885	0.00801630420279287	CCL5/COL1A1/CREB3L1/CXCL13/CXCL8/GCLM/POSTN/SHISA2/TNC	9

BP	GO:0050878	regulation of body fluid levels	17/349	365/18870	0.000486251886930265	0.0108724676564083	0.00818858824694733	ANO1/CCND1/COL3A1/F2R/F2RL1/FCER1G/FZD6/GNA12/GNA13/HIF1A/ITGA2/MTCO2P12/MYH9/OAS2/PLAU/SERPINE1/SLC7A11	17

BP	GO:0045667	regulation of osteoblast differentiation	10/349	150/18870	0.000488559246725397	0.0108724676564083	0.00818858824694733	CDK6/CEBPB/CTHRC1/JUND/SNAI2/SOX9/TNFAIP6/TP63/TWSG1/WNT7B	10

BP	GO:0071674	mononuclear cell migration	12/349	207/18870	0.000494285000476967	0.0109394504501166	0.00823903627168664	APP/CCL18/CCL5/CXCL10/CXCL13/ICAM1/IL27RA/MSN/PLA2G7/PLEC/S100A7/SERPINE1	12

BP	GO:2001237	negative regulation of extrinsic apoptotic signaling pathway	8/349	99/18870	0.000501543350604726	0.0109867285905222	0.00827464375630717	GCLM/ICAM1/IFI6/ITGA6/ITGAV/SERPINE1/SNAI2/TNFAIP3	8

BP	GO:0045446	endothelial cell differentiation	9/349	124/18870	0.000501876380500518	0.0109867285905222	0.00827464375630717	CLDN1/CLIC4/F2RL1/FZD1/FZD2/ICAM1/MET/MSN/STC1	9

BP	GO:0001890	placenta development	10/349	151/18870	0.0005148005413936	0.0112087382742347	0.008441850129709	CEBPB/EGFR/FOSL1/HIF1A/HSP90AB1/PHLDA2/SOCS3/SPP1/STC1/WNT7B	10

BP	GO:0071675	regulation of mononuclear cell migration	9/349	125/18870	0.000532189284647347	0.0115250453685995	0.00868007650454812	APP/CCL5/CXCL10/CXCL13/IL27RA/MSN/PLA2G7/S100A7/SERPINE1	9

BP	GO:0060349	bone morphogenesis	8/349	100/18870	0.000536542755704859	0.0115571883421346	0.00870428495323054	COL1A1/COL3A1/DLX5/MMP13/SERPINH1/SFRP4/SOX9/STC1	8

BP	GO:0030595	leukocyte chemotaxis	13/349	240/18870	0.000556220418343014	0.0118835569997619	0.00895008918447548	CCL18/CCL5/CXCL10/CXCL13/CXCL8/CXCL9/F2RL1/FCER1G/PLA2G7/PLEC/S100A7/SERPINE1/TNFAIP6	13

BP	GO:0070371	ERK1 and ERK2 cascade	16/349	336/18870	0.000557594903911372	0.0118835569997619	0.00895008918447548	ACKR3/APP/CCL18/CCL5/EGFR/F2R/F2RL1/GPNMB/HLA-DRB1/ICAM1/INHBA/ITGAV/MFHAS1/S100A7/SOX9/TREM2	16

BP	GO:0033280	response to vitamin D	5/349	37/18870	0.000563567687919474	0.0118939343030486	0.00895790484016782	CXCL10/SNAI2/SPP1/STC1/TNC	5

BP	GO:0002688	regulation of leukocyte chemotaxis	9/349	126/18870	0.000563987450814868	0.0118939343030486	0.00895790484016782	CCL5/CXCL10/CXCL13/CXCL8/F2RL1/PLA2G7/S100A7/SERPINE1/TNFAIP6	9

BP	GO:0030857	negative regulation of epithelial cell differentiation	6/349	56/18870	0.000571218821301956	0.0119836948552306	0.00902550791114166	CCND1/FST/MMP9/SOX9/STAT1/TP63	6

BP	GO:0030111	regulation of Wnt signaling pathway	16/349	338/18870	0.000594422119795874	0.012405866831802	0.00934346631716231	APP/COL1A1/CTHRC1/DLX5/EGFR/FZD1/FZD6/FZD7/NRARP/SFRP4/SHISA2/SNAI2/SOX4/SOX9/TNFAIP3/ZNF703	16

BP	GO:0140014	mitotic nuclear division	14/349	274/18870	0.000616218777270636	0.0127944805919903	0.00963615038575134	ANLN/DLGAP5/KIF11/KIF14/KIF4A/MAPRE1/MISP/MYBL2/MZT1/NCAPD2/REEP3/TPX2/TRIP13/UBE2C	14

BP	GO:0046651	lymphocyte proliferation	15/349	307/18870	0.000633185398317462	0.0130700939620676	0.00984372832244781	AHR/CCL5/CEBPB/EFNB1/ELF4/FCGR3A/GAL/GPNMB/HLA-DRB1/HPRT1/IRS2/MSN/PRNP/TFRC/TWSG1	15

BP	GO:0045637	regulation of myeloid cell differentiation	12/349	213/18870	0.00063718532994926	0.0130700939620676	0.00984372832244781	C1QC/CDK6/CEBPB/HIF1A/HLA-DRB1/INHBA/ISG15/LOX/STAT1/TNFAIP6/TREM2/TRIB1	12

BP	GO:0014912	negative regulation of smooth muscle cell migration	5/349	38/18870	0.000639227534887615	0.0130700939620676	0.00984372832244781	GNA12/GNA13/IGFBP3/SERPINE1/TRIB1	5

BP	GO:1900118	negative regulation of execution phase of apoptosis	4/349	22/18870	0.000646316259838695	0.0131482923971225	0.00990262339635895	MTRNR2L12/MTRNR2L2/MTRNR2L8/PAK2	4

BP	GO:0048771	tissue remodeling	11/349	184/18870	0.000651450295012791	0.0131861396397564	0.00993112800966339	CSPG4/CTHRC1/F2R/GJA1/GPNMB/HIF1A/SEMA3C/SNX10/SPP1/TFRC/TNFAIP3	11

BP	GO:0048754	branching morphogenesis of an epithelial tube	10/349	156/18870	0.000664170040208463	0.0133629011917667	0.010064255797489	CLIC4/COL4A1/GLI2/GNA13/HOXB7/HOXD11/MET/NRARP/SOX9/TNC	10

BP	GO:0001886	endothelial cell morphogenesis	3/349	10/18870	0.000683405572369398	0.0133629011917667	0.010064255797489	CLIC4/MET/STC1	3

BP	GO:0038063	collagen-activated tyrosine kinase receptor signaling pathway	3/349	10/18870	0.000683405572369398	0.0133629011917667	0.010064255797489	COL1A1/COL4A1/COL4A2	3

BP	GO:0044837	actomyosin contractile ring organization	3/349	10/18870	0.000683405572369398	0.0133629011917667	0.010064255797489	ANLN/ECT2/PLEC	3

BP	GO:0060054	positive regulation of epithelial cell proliferation involved in wound healing	3/349	10/18870	0.000683405572369398	0.0133629011917667	0.010064255797489	CLDN1/FZD7/MMP12	3

BP	GO:0060536	cartilage morphogenesis	3/349	10/18870	0.000683405572369398	0.0133629011917667	0.010064255797489	SNAI2/STC1/WNT7B	3

BP	GO:0070099	regulation of chemokine-mediated signaling pathway	3/349	10/18870	0.000683405572369398	0.0133629011917667	0.010064255797489	CCL5/HIF1A/TREM2	3

BP	GO:0060348	bone development	12/349	215/18870	0.000691915564899729	0.0134639415237493	0.0101403542234479	BGN/COL1A1/COL3A1/DLX5/GJA1/LOX/MMP13/SERPINH1/SFRP4/SNX10/SOX9/STC1	12

BP	GO:0032642	regulation of chemokine production	8/349	104/18870	0.000696852711920263	0.0134948207866097	0.0101636109096872	APP/EIF2AK2/F2RL1/HIF1A/OAS3/POSTN/SNAI2/TREM2	8

BP	GO:0071621	granulocyte chemotaxis	9/349	130/18870	0.000707144368284469	0.0136286005523916	0.0102643670077856	CCL18/CCL5/CXCL10/CXCL13/CXCL8/CXCL9/FCER1G/S100A7/TNFAIP6	9

BP	GO:0050808	synapse organization	20/349	483/18870	0.000713882916417449	0.0136929542253785	0.0103128349128576	AGRN/APP/C1QA/C1QB/C1QC/CLSTN1/COL4A1/F2R/FZD1/NEFL/PFN2/PRNP/SEZ6L2/SLC7A11/SRGN/THBS2/TNC/TREM2/TUBB/WNT7B	20

BP	GO:0032602	chemokine production	8/349	105/18870	0.0007424205686813	0.014172843841935	0.0106742633022674	APP/EIF2AK2/F2RL1/HIF1A/OAS3/POSTN/SNAI2/TREM2	8

BP	GO:0043616	keratinocyte proliferation	6/349	59/18870	0.000756779545052546	0.0143788113559984	0.0108293875314868	CD109/EPPK1/FST/PTPRK/SNAI2/TP63	6

BP	GO:0045591	positive regulation of regulatory T cell differentiation	4/349	23/18870	0.000771059069234802	0.0145813423984872	0.0109819235855434	HLA-DRA/HLA-DRB1/SOCS1/SOX12	4

BP	GO:0002440	production of molecular mediator of immune response	15/349	314/18870	0.000797138704586983	0.0149343009398901	0.0112477539614183	BST2/DDX21/F2RL1/IGKV1-5/IGKV3-20/IGKV4-1/IGLV1-40/IGLV3-19/IGLV3-21/IGLV3-25/IL27RA/PANX1/RSAD2/SLC7A5/TFRC	15

BP	GO:0032943	mononuclear cell proliferation	15/349	314/18870	0.000797138704586983	0.0149343009398901	0.0112477539614183	AHR/CCL5/CEBPB/EFNB1/ELF4/FCGR3A/GAL/GPNMB/HLA-DRB1/HPRT1/IRS2/MSN/PRNP/TFRC/TWSG1	15

BP	GO:0048568	embryonic organ development	19/349	453/18870	0.000828597775028823	0.0154518140639634	0.0116375184582508	CEBPB/CHST11/COL11A1/CTHRC1/CXCL8/DLX5/EGFR/EN1/FOXE1/FZD2/FZD6/GLI2/HIF1A/HOXB7/HOXD10/PHLDA2/SOCS3/SOX9/WNT7B	19

BP	GO:0061041	regulation of wound healing	9/349	133/18870	0.000832909208491476	0.0154606372894178	0.011644163661763	CD109/CLDN1/EPPK1/F2R/F2RL1/PLAU/SERPINE1/TNFAIP3/TNFRSF12A	9

BP	GO:0030593	neutrophil chemotaxis	8/349	107/18870	0.000840748197406245	0.0155164822002422	0.0116862232010396	CCL18/CCL5/CXCL10/CXCL13/CXCL8/CXCL9/FCER1G/TNFAIP6	8

BP	GO:0046883	regulation of hormone secretion	13/349	251/18870	0.000843622046140281	0.0155164822002422	0.0116862232010396	ANO1/CCL5/F2RL1/GAL/GJA1/HIF1A/HLA-DRB1/INHBA/IRS2/NMB/SLC16A1/SOX4/SPP1	13

BP	GO:0060070	canonical Wnt signaling pathway	15/349	316/18870	0.000850122950675557	0.0155649783878234	0.011722748056684	COL1A1/CTHRC1/DLX5/EGFR/FZD1/FZD2/FZD6/FZD7/NRARP/SFRP4/SNAI2/SOX4/SOX9/WNT7B/ZNF703	15

BP	GO:0048565	digestive tract development	9/349	134/18870	0.000878661370236201	0.0160146968294634	0.0120614530555882	COL3A1/CXCL8/EGFR/FOXE1/GLI2/HIF1A/HOXD13/SOX9/TP63	9

BP	GO:0010839	negative regulation of keratinocyte proliferation	4/349	24/18870	0.000911887327226194	0.0164712204218256	0.0124052833470186	CD109/EPPK1/PTPRK/SNAI2	4

BP	GO:0060571	morphogenesis of an epithelial fold	4/349	24/18870	0.000911887327226194	0.0164712204218256	0.0124052833470186	EGFR/HIF1A/HOXD13/TP63	4

BP	GO:0071492	cellular response to UV-A	3/349	11/18870	0.000926839228426801	0.0165190637703679	0.0124413165175875	MMP1/MMP3/MMP9	3

BP	GO:0071895	odontoblast differentiation	3/349	11/18870	0.000926839228426801	0.0165190637703679	0.0124413165175875	CEBPB/SERPINE1/TUBB	3

BP	GO:1990440	positive regulation of transcription from RNA polymerase II promoter in response to endoplasmic reticulum stress	3/349	11/18870	0.000926839228426801	0.0165190637703679	0.0124413165175875	CEBPB/CREB3L1/HSPA5	3

BP	GO:0050866	negative regulation of cell activation	12/349	223/18870	0.000951853246085115	0.0168901536353782	0.0127208024818794	CEBPB/GAL/GPNMB/HLA-DRB1/INHBA/NRARP/PRNP/RUNX3/SOCS1/TNFAIP3/TREM2/TWSG1	12

BP	GO:0016055	Wnt signaling pathway	19/349	459/18870	0.000967576064282541	0.0170938438023249	0.0128742115293734	APP/CCND1/COL1A1/CTHRC1/DLX5/EGFR/FZD1/FZD2/FZD6/FZD7/NRARP/SFRP4/SHISA2/SNAI2/SOX4/SOX9/TNFAIP3/WNT7B/ZNF703	19

BP	GO:0007596	blood coagulation	12/349	224/18870	0.000989421355625735	0.0174034463775566	0.0131073884022678	COL3A1/F2R/F2RL1/FCER1G/FZD6/GNA12/GNA13/ITGA2/MYH9/PLAU/SERPINE1/SLC7A11	12

BP	GO:1902105	regulation of leukocyte differentiation	15/349	321/18870	0.00099580425016349	0.0174395631289502	0.0131345896978086	C1QC/CDK6/CEBPB/GLI2/HLA-DRA/HLA-DRB1/INHBA/NRARP/RUNX3/SOCS1/SOX12/SOX4/TNFAIP6/TREM2/TRIB1	15

BP	GO:0050727	regulation of inflammatory response	18/349	425/18870	0.00101052602350911	0.0174812477422228	0.013165984423009	ABCC1/AHR/APP/CCL5/CEBPB/ELF4/HLA-DRB1/MFHAS1/MMP3/MMP9/NT5E/PLA2G7/SERPINE1/SOCS3/TNC/TNFAIP3/TNFAIP6/TREM2	18

BP	GO:0016049	cell growth	20/349	497/18870	0.00101216727721174	0.0174812477422228	0.013165984423009	ALCAM/APP/BST2/CLSTN1/EGFR/GJA1/HSP90AA1/HSP90AB1/IGFBP3/INHBA/KIF14/KRT17/PLXNA1/POSTN/PPT1/SEMA3C/SH3BP4/SOX9/SPP1/TNC	20

BP	GO:0198738	cell-cell signaling by wnt	19/349	461/18870	0.00101813829854647	0.0174812477422228	0.013165984423009	APP/CCND1/COL1A1/CTHRC1/DLX5/EGFR/FZD1/FZD2/FZD6/FZD7/NRARP/SFRP4/SHISA2/SNAI2/SOX4/SOX9/TNFAIP3/WNT7B/ZNF703	19

BP	GO:0071634	regulation of transforming growth factor beta production	5/349	42/18870	0.00101988411604329	0.0174812477422228	0.013165984423009	HIF1A/HSP90AB1/ITGAV/LAPTM4B/LUM	5

BP	GO:2000403	positive regulation of lymphocyte migration	5/349	42/18870	0.00101988411604329	0.0174812477422228	0.013165984423009	APP/CCL5/CXCL10/CXCL13/S100A7	5

BP	GO:0043491	phosphatidylinositol 3-kinase/protein kinase B signal transduction	14/349	290/18870	0.0010677395091307	0.0180875490311177	0.0136226196382379	CCL5/EGFR/F2R/F2RL1/FCGR3A/HSP90AA1/HSP90AB1/LOX/MFHAS1/MTDH/NTS/SESN3/SOX9/TREM2	14

BP	GO:0071222	cellular response to lipopolysaccharide	12/349	226/18870	0.00106826660190861	0.0180875490311177	0.0136226196382379	CD14/CXCL10/CXCL13/CXCL8/CXCL9/MMP3/MMP9/MTDH/SERPINE1/SLC7A5/TNFAIP3/TRIB1	12

BP	GO:1902106	negative regulation of leukocyte differentiation	8/349	111/18870	0.00106872807085551	0.0180875490311177	0.0136226196382379	C1QC/CDK6/INHBA/NRARP/RUNX3/SOCS1/TNFAIP6/TRIB1	8

BP	GO:0003002	regionalization	18/349	430/18870	0.00115271903713571	0.0194274154041115	0.0146317386700071	BASP1/BHLHE41/C1QA/EGR2/EN1/GLI2/HIF1A/HOXB7/HOXC10/HOXC13/HOXD10/HOXD11/HOXD13/IRX2/NRARP/SEMA3C/TP63/WNT7B	18

BP	GO:0031100	animal organ regeneration	6/349	64/18870	0.00116573051288168	0.0194836618501552	0.0146741006251365	CCNA2/CCND1/CEBPB/CLDN1/PCNA/SLC7A5	6

BP	GO:0048247	lymphocyte chemotaxis	6/349	64/18870	0.00116573051288168	0.0194836618501552	0.0146741006251365	CCL18/CCL5/CXCL10/CXCL13/PLEC/S100A7	6

BP	GO:0050817	coagulation	12/349	229/18870	0.00119627620222704	0.0199115724899608	0.0149963810996882	COL3A1/F2R/F2RL1/FCER1G/FZD6/GNA12/GNA13/ITGA2/MYH9/PLAU/SERPINE1/SLC7A11	12

BP	GO:0000101	sulfur amino acid transport	3/349	12/18870	0.00121891056495635	0.0201220153919844	0.0151548759629172	SLC1A4/SLC7A11/SLC7A5	3

BP	GO:0051798	positive regulation of hair follicle development	3/349	12/18870	0.00121891056495635	0.0201220153919844	0.0151548759629172	FST/GAL/KRT17	3

BP	GO:0048705	skeletal system morphogenesis	12/349	230/18870	0.00124167244659777	0.0201340969763132	0.0151639751961884	CHST11/COL11A1/COL1A1/COL3A1/DLX5/HOXB7/HOXD10/MMP13/SERPINH1/SFRP4/SOX9/STC1	12

BP	GO:0032967	positive regulation of collagen biosynthetic process	4/349	26/18870	0.00124614632558814	0.0201340969763132	0.0151639751961884	CREB3L1/F2R/INHBA/ITGA2	4

BP	GO:0060740	prostate gland epithelium morphogenesis	4/349	26/18870	0.00124614632558814	0.0201340969763132	0.0151639751961884	HOXD13/SOX9/TNC/TP63	4

BP	GO:1901623	regulation of lymphocyte chemotaxis	4/349	26/18870	0.00124614632558814	0.0201340969763132	0.0151639751961884	CCL5/CXCL10/CXCL13/S100A7	4

BP	GO:0060828	regulation of canonical Wnt signaling pathway	13/349	262/18870	0.00124662512440609	0.0201340969763132	0.0151639751961884	COL1A1/CTHRC1/DLX5/EGFR/FZD1/FZD6/FZD7/NRARP/SFRP4/SNAI2/SOX4/SOX9/ZNF703	13

BP	GO:0046887	positive regulation of hormone secretion	9/349	141/18870	0.00125931477172512	0.0201340969763132	0.0151639751961884	ANO1/GAL/HIF1A/HLA-DRB1/INHBA/IRS2/NMB/SOX4/SPP1	9

BP	GO:0050691	regulation of defense response to virus by host	5/349	44/18870	0.00126338647090916	0.0201340969763132	0.0151639751961884	DTX3L/HSP90AA1/MMP12/STAT1/TNFAIP3	5

BP	GO:1905330	regulation of morphogenesis of an epithelium	6/349	65/18870	0.00126462922914777	0.0201340969763132	0.0151639751961884	CXCL10/GJA1/HOXB7/HOXD13/SNAI2/SOX9	6

BP	GO:2000117	negative regulation of cysteine-type endopeptidase activity	6/349	65/18870	0.00126462922914777	0.0201340969763132	0.0151639751961884	CST1/IFI6/LAMP3/MAGEA3/MMP9/PAK2	6

BP	GO:0007599	hemostasis	12/349	231/18870	0.00128848690658098	0.0204331703138118	0.0153892219841127	COL3A1/F2R/F2RL1/FCER1G/FZD6/GNA12/GNA13/ITGA2/MYH9/PLAU/SERPINE1/SLC7A11	12

BP	GO:0001960	negative regulation of cytokine-mediated signaling pathway	7/349	89/18870	0.00131118648613892	0.0207116045732062	0.0155989244708044	CCL5/F2RL1/ISG15/MMP12/OAS3/PXDN/TREM2	7

BP	GO:0001889	liver development	9/349	142/18870	0.00132315533595177	0.0208190222391162	0.0156798259794943	CCND1/CEBPB/CLDN1/ITGA2/MET/PCNA/SLC7A5/SOX9/TNFAIP3	9

BP	GO:0030856	regulation of epithelial cell differentiation	10/349	171/18870	0.0013400018122316	0.0208960166113513	0.0157378142146852	CCND1/CD109/CEBPB/FST/MMP9/SERPINE1/SFRP4/SOX9/STAT1/TP63	10

BP	GO:0048660	regulation of smooth muscle cell proliferation	10/349	171/18870	0.0013400018122316	0.0208960166113513	0.0157378142146852	CCL5/GJA1/GNA12/GNA13/IGFBP3/ITGA2/MMP9/STAT1/TNFAIP3/TRIB1	10

BP	GO:0044344	cellular response to fibroblast growth factor stimulus	8/349	115/18870	0.00134361179303376	0.0208960166113513	0.0157378142146852	CCL5/COL1A1/CREB3L1/CXCL13/CXCL8/GCLM/POSTN/SHISA2	8

BP	GO:0002695	negative regulation of leukocyte activation	11/349	202/18870	0.00139504479472574	0.0216124632044433	0.0162774052566784	CEBPB/GAL/GPNMB/HLA-DRB1/INHBA/NRARP/PRNP/RUNX3/SOCS1/TNFAIP3/TWSG1	11

BP	GO:0043410	positive regulation of MAPK cascade	19/349	474/18870	0.00140524973451622	0.0216871491595071	0.0163336549097631	ACKR3/APP/CCL18/CCL5/CSPG4/EGFR/EIF2AK2/F2R/F2RL1/FZD7/GPNMB/HLA-DRB1/ICAM1/IGFBP3/INHBA/MFHAS1/S100A7/TREM2/WNT7B	19

BP	GO:0010714	positive regulation of collagen metabolic process	4/349	27/18870	0.00144174745306194	0.0221654913776088	0.0166939178775593	CREB3L1/F2R/INHBA/ITGA2	4

BP	GO:1903034	regulation of response to wounding	10/349	173/18870	0.00146202087141417	0.0223917112929896	0.0168642951827722	CD109/CLDN1/EPPK1/F2R/F2RL1/PLAU/SERPINE1/SPP1/TNFAIP3/TNFRSF12A	10

BP	GO:0060021	roof of mouth development	7/349	91/18870	0.0014927301823038	0.0227754438421201	0.0171533029720876	DLX5/FOXE1/FZD1/FZD2/INHBA/MSC/SNAI2	7

BP	GO:0061008	hepaticobiliary system development	9/349	145/18870	0.00153047975317999	0.0232310898947418	0.0174964723453316	CCND1/CEBPB/CLDN1/ITGA2/MET/PCNA/SLC7A5/SOX9/TNFAIP3	9

BP	GO:0045646	regulation of erythrocyte differentiation	5/349	46/18870	0.00154734567125914	0.0232310898947418	0.0174964723453316	CDK6/HIF1A/INHBA/ISG15/STAT1	5

BP	GO:0085029	extracellular matrix assembly	5/349	46/18870	0.00154734567125914	0.0232310898947418	0.0174964723453316	COL1A2/COL3A1/LOX/PXDN/SOX9	5

BP	GO:0060100	positive regulation of phagocytosis, engulfment	3/349	13/18870	0.00156296558129966	0.0232310898947418	0.0174964723453316	F2RL1/ITGA2/TREM2	3

BP	GO:0070493	thrombin-activated receptor signaling pathway	3/349	13/18870	0.00156296558129966	0.0232310898947418	0.0174964723453316	F2R/F2RL1/MET	3

BP	GO:0090177	establishment of planar polarity involved in neural tube closure	3/349	13/18870	0.00156296558129966	0.0232310898947418	0.0174964723453316	CTHRC1/FZD1/FZD2	3

BP	GO:1905155	positive regulation of membrane invagination	3/349	13/18870	0.00156296558129966	0.0232310898947418	0.0174964723453316	F2RL1/ITGA2/TREM2	3

BP	GO:0001738	morphogenesis of a polarized epithelium	7/349	92/18870	0.00159055366136916	0.0235028666708647	0.0177011607550912	CTHRC1/FZD1/FZD2/FZD6/FZD7/MSN/TP63	7

BP	GO:0048659	smooth muscle cell proliferation	10/349	175/18870	0.00159292020882474	0.0235028666708647	0.0177011607550912	CCL5/GJA1/GNA12/GNA13/IGFBP3/ITGA2/MMP9/STAT1/TNFAIP3/TRIB1	10

BP	GO:0035567	non-canonical Wnt signaling pathway	6/349	68/18870	0.0016000871991079	0.0235105052644739	0.0177069137543025	CTHRC1/FZD1/FZD2/FZD6/FZD7/SFRP4	6

BP	GO:0055123	digestive system development	9/349	146/18870	0.00160511145673543	0.0235105052644739	0.0177069137543025	COL3A1/CXCL8/EGFR/FOXE1/GLI2/HIF1A/HOXD13/SOX9/TP63	9

BP	GO:0031349	positive regulation of defense response	19/349	480/18870	0.00162242853605441	0.0236780512435767	0.0178331008425203	ABCC1/APP/CCL5/CD14/CEBPB/DDX60/EIF2AK2/F2RL1/FOSL1/HSP90AA1/MFHAS1/MMP12/OAS3/PAK2/PLA2G7/RSAD2/SERPINE1/TNFAIP3/TREM2	19

BP	GO:0014850	response to muscle activity	4/349	28/18870	0.00165776976177136	0.0237973937129291	0.0179229835051121	HIF1A/ITGA2/POSTN/SLC7A5	4

BP	GO:0060512	prostate gland morphogenesis	4/349	28/18870	0.00165776976177136	0.0237973937129291	0.0179229835051121	HOXD13/SOX9/TNC/TP63	4

BP	GO:0050670	regulation of lymphocyte proliferation	12/349	238/18870	0.00165879714570251	0.0237973937129291	0.0179229835051121	AHR/CCL5/CEBPB/EFNB1/FCGR3A/GAL/GPNMB/HLA-DRB1/IRS2/PRNP/TFRC/TWSG1	12

BP	GO:1903131	mononuclear cell differentiation	19/349	481/18870	0.00166126025238804	0.0237973937129291	0.0179229835051121	CDK6/CEBPB/F2RL1/FCER1G/FZD7/GLI2/HLA-DRA/HLA-DRB1/INHBA/MYH9/NRARP/PRR7/RSAD2/RUNX3/SOCS1/SOCS3/SOX12/SOX4/TREM2	19

BP	GO:0030336	negative regulation of cell migration	16/349	373/18870	0.00166654928572812	0.0237973937129291	0.0179229835051121	BST2/CLIC4/COL3A1/CXCL13/EPPK1/GJA1/GNA12/GNA13/IGFBP3/IL27RA/PFN2/PTPRK/SERPINE1/STC1/TNFAIP6/TRIB1	16

BP	GO:0032609	type II interferon production	8/349	119/18870	0.00167196187208514	0.0237973937129291	0.0179229835051121	CD14/F2RL1/HLA-DRB1/IL27RA/INHBA/ISG15/PRNP/SLC7A5	8

BP	GO:0032649	regulation of type II interferon production	8/349	119/18870	0.00167196187208514	0.0237973937129291	0.0179229835051121	CD14/F2RL1/HLA-DRB1/IL27RA/INHBA/ISG15/PRNP/SLC7A5	8

BP	GO:0042475	odontogenesis of dentin-containing tooth	7/349	93/18870	0.00169329609420405	0.0240161854487814	0.0180877662842701	AMTN/FST/GLI2/SERPINE1/TNC/TP63/WDR72	7

BP	GO:0010837	regulation of keratinocyte proliferation	5/349	47/18870	0.00170570063470694	0.024022944603495	0.0180928569349481	CD109/EPPK1/PTPRK/SNAI2/TP63	5

BP	GO:0090102	cochlea development	5/349	47/18870	0.00170570063470694	0.024022944603495	0.0180928569349481	CCNA2/CTHRC1/FZD2/MCM2/SOX9	5

BP	GO:1901653	cellular response to peptide	16/349	374/18870	0.00171245825433414	0.0240340830956722	0.0181012458792995	ABCC1/ANO1/APP/CCNA2/GJA1/ICAM1/INHBA/IRS2/NUCKS1/PRNP/SESN3/SOCS1/SOCS3/STAT1/TREM2/YWHAG	16

BP	GO:0030216	keratinocyte differentiation	10/349	177/18870	0.00173316960174019	0.0242403026243385	0.0182565599130674	CD109/CLIC4/KRT10/KRT14/KRT17/KRT75/PLEC/S100A7/SPRR2G/TP63	10

BP	GO:0060761	negative regulation of response to cytokine stimulus	7/349	94/18870	0.00180112382774572	0.0251035528656047	0.0189067159772834	CCL5/F2RL1/ISG15/MMP12/OAS3/PXDN/TREM2	7

BP	GO:0030217	T cell differentiation	14/349	307/18870	0.00182422887558302	0.0253379100374083	0.0190832218491117	CDK6/FCER1G/FZD7/GLI2/HLA-DRA/HLA-DRB1/NRARP/PRR7/RSAD2/RUNX3/SOCS1/SOCS3/SOX12/SOX4	14

BP	GO:0001736	establishment of planar polarity	6/349	70/18870	0.00185865770586793	0.0256392919151918	0.019310207311865	CTHRC1/FZD1/FZD2/FZD6/FZD7/TP63	6

BP	GO:0007164	establishment of tissue polarity	6/349	70/18870	0.00185865770586793	0.0256392919151918	0.019310207311865	CTHRC1/FZD1/FZD2/FZD6/FZD7/TP63	6

BP	GO:0090101	negative regulation of transmembrane receptor protein serine/threonine kinase signaling pathway	10/349	179/18870	0.00188325240414367	0.0258898999450194	0.0194989525013186	ASPN/CD109/CHST11/FST/FZD1/HSPA5/HTRA3/PMEPA1/TNFAIP6/TWSG1	10

BP	GO:0032944	regulation of mononuclear cell proliferation	12/349	242/18870	0.00190706105081292	0.0260394641107608	0.0196115966046131	AHR/CCL5/CEBPB/EFNB1/FCGR3A/GAL/GPNMB/HLA-DRB1/IRS2/PRNP/TFRC/TWSG1	12

BP	GO:0097529	myeloid leukocyte migration	12/349	242/18870	0.00190706105081292	0.0260394641107608	0.0196115966046131	CCL18/CCL5/CXCL10/CXCL13/CXCL8/CXCL9/FCER1G/PLA2G7/S100A7/SERPINE1/TNFAIP6/TREM2	12

BP	GO:0072091	regulation of stem cell proliferation	7/349	95/18870	0.00191420548504158	0.0260487151815793	0.0196185640394375	CD109/EIF2AK2/EPCAM/GJA1/SNAI2/SOX9/TP63	7

BP	GO:0021782	glial cell development	8/349	122/18870	0.00195750548718946	0.0265214781168852	0.019974624975922	ANO1/APP/C1QA/CDK6/ITGB4/PLEC/SOX4/TREM2	8

BP	GO:0051412	response to corticosterone	3/349	14/18870	0.0019621153125203	0.0265214781168852	0.019974624975922	FOSB/FOSL1/NEFL	3

BP	GO:0002548	monocyte chemotaxis	6/349	71/18870	0.00199922948895268	0.0267538085764166	0.0201496044314098	CCL18/CCL5/CXCL10/PLA2G7/S100A7/SERPINE1	6

BP	GO:0090307	mitotic spindle assembly	6/349	71/18870	0.00199922948895268	0.0267538085764166	0.0201496044314098	KIF11/KIF4A/MISP/MYBL2/MZT1/TPX2	6

BP	GO:1903317	regulation of protein maturation	6/349	71/18870	0.00199922948895268	0.0267538085764166	0.0201496044314098	MAGEA3/MYH9/PLAU/PRNP/SERPINE1/SOX4	6

BP	GO:0070664	negative regulation of leukocyte proliferation	7/349	96/18870	0.00203271189474178	0.026825137164302	0.0202033254867293	CEBPB/GAL/GPNMB/HLA-DRB1/PRNP/TNFAIP3/TWSG1	7

BP	GO:0060560	developmental growth involved in morphogenesis	12/349	244/18870	0.00204217525009609	0.026825137164302	0.0202033254867293	ALCAM/APP/HOXD13/HSP90AA1/HSP90AB1/PLXNA1/POSTN/SEMA3C/SOX9/SPP1/TNC/WNT7B	12

BP	GO:0007520	myoblast fusion	5/349	49/18870	0.00205760507432746	0.026825137164302	0.0202033254867293	ADAM12/CXCL10/CXCL9/MYH9/PTGFRN	5

BP	GO:0033628	regulation of cell adhesion mediated by integrin	5/349	49/18870	0.00205760507432746	0.026825137164302	0.0202033254867293	CCL5/CXCL13/PLAU/SERPINE1/SNAI2	5

BP	GO:0048546	digestive tract morphogenesis	5/349	49/18870	0.00205760507432746	0.026825137164302	0.0202033254867293	EGFR/GLI2/HIF1A/HOXD13/TP63	5

BP	GO:0060071	Wnt signaling pathway, planar cell polarity pathway	5/349	49/18870	0.00205760507432746	0.026825137164302	0.0202033254867293	CTHRC1/FZD1/FZD2/FZD6/FZD7	5

BP	GO:0032479	regulation of type I interferon production	8/349	123/18870	0.00206077575926852	0.026825137164302	0.0202033254867293	CD14/HSP90AA1/ISG15/MMP12/NMB/OAS2/OAS3/STAT1	8

BP	GO:0032606	type I interferon production	8/349	123/18870	0.00206077575926852	0.026825137164302	0.0202033254867293	CD14/HSP90AA1/ISG15/MMP12/NMB/OAS2/OAS3/STAT1	8

BP	GO:0001818	negative regulation of cytokine production	16/349	381/18870	0.0020644966536578	0.026825137164302	0.0202033254867293	BST2/ELF4/F2RL1/GPNMB/HLA-DRB1/HSP90AB1/IL27RA/INHBA/LAPTM4B/NMB/OAS3/PRNP/SRGN/TNFAIP3/TREM2/TWSG1	16

BP	GO:0016331	morphogenesis of embryonic epithelium	9/349	152/18870	0.00211649872385059	0.0269421292716569	0.0202914379765105	CTHRC1/FZD1/FZD2/FZD6/HIF1A/IRX2/SOX9/TP63/WNT7B	9

BP	GO:0098813	nuclear chromosome segregation	14/349	312/18870	0.00211701329321694	0.0269421292716569	0.0202914379765105	DLGAP5/ECT2/KIF11/KIF14/KIF4A/MAPRE1/MISP/MYBL2/MZT1/NCAPD2/TOP2A/TPX2/TRIP13/UBE2C	14

BP	GO:1903037	regulation of leukocyte cell-cell adhesion	16/349	382/18870	0.00211940527954502	0.0269421292716569	0.0202914379765105	CCL5/CEBPB/CHST2/EFNB1/GLI2/GPNMB/HLA-DRA/HLA-DRB1/NRARP/PRNP/RUNX3/SOCS1/SOX12/SOX4/TFRC/TWSG1	16

BP	GO:0009612	response to mechanical stimulus	11/349	213/18870	0.0021216066397393	0.0269421292716569	0.0202914379765105	COL11A1/COL1A1/CXCL10/FOSB/FOSL1/ITGA2/PLEC/POSTN/SOX9/STAT1/TNC	11

BP	GO:0014823	response to activity	6/349	72/18870	0.00214769152096877	0.0269421292716569	0.0202914379765105	COL4A2/GCLM/HIF1A/ITGA2/POSTN/SLC7A5	6

BP	GO:0032722	positive regulation of chemokine production	6/349	72/18870	0.00214769152096877	0.0269421292716569	0.0202914379765105	APP/EIF2AK2/F2RL1/HIF1A/OAS3/POSTN	6

BP	GO:0010955	negative regulation of protein processing	4/349	30/18870	0.00215535420698397	0.0269421292716569	0.0202914379765105	MAGEA3/PLAU/PRNP/SERPINE1	4

BP	GO:0070168	negative regulation of biomineral tissue development	4/349	30/18870	0.00215535420698397	0.0269421292716569	0.0202914379765105	ASPN/HIF1A/SOX9/SRGN	4

BP	GO:0071295	cellular response to vitamin	4/349	30/18870	0.00215535420698397	0.0269421292716569	0.0202914379765105	COL1A1/POSTN/SNAI2/TNC	4

BP	GO:1900117	regulation of execution phase of apoptosis	4/349	30/18870	0.00215535420698397	0.0269421292716569	0.0202914379765105	MTRNR2L12/MTRNR2L2/MTRNR2L8/PAK2	4

BP	GO:1903318	negative regulation of protein maturation	4/349	30/18870	0.00215535420698397	0.0269421292716569	0.0202914379765105	MAGEA3/PLAU/PRNP/SERPINE1	4

BP	GO:0050688	regulation of defense response to virus	7/349	97/18870	0.00215681601852114	0.0269421292716569	0.0202914379765105	DTX3L/F2RL1/HSP90AA1/MMP12/STAT1/TNFAIP3/TREM2	7

BP	GO:0002761	regulation of myeloid leukocyte differentiation	8/349	124/18870	0.00216828606125251	0.0269421292716569	0.0202914379765105	C1QC/CDK6/CEBPB/HLA-DRB1/INHBA/TNFAIP6/TREM2/TRIB1	8

BP	GO:0043254	regulation of protein-containing complex assembly	17/349	419/18870	0.00217567693852048	0.0269421292716569	0.0202914379765105	CXCL13/EIF2AK2/FSCN1/GNL3L/HSP90AA1/HSPA5/ISG15/KIF14/MAPRE1/MET/MMP1/MMP3/MSN/PFN2/SOX9/TFRC/TREM2	17

BP	GO:0070372	regulation of ERK1 and ERK2 cascade	14/349	313/18870	0.00218000382066394	0.0269421292716569	0.0202914379765105	ACKR3/APP/CCL18/CCL5/EGFR/F2R/F2RL1/GPNMB/HLA-DRB1/ICAM1/INHBA/MFHAS1/S100A7/TREM2	14

BP	GO:0048863	stem cell differentiation	12/349	246/18870	0.00218503461023241	0.0269421292716569	0.0202914379765105	CDK6/EFNB1/EIF2AK2/EPCAM/FZD1/HIF1A/KRT14/SEMA3C/SNAI2/SOX9/TP63/ZNF281	12

BP	GO:0022407	regulation of cell-cell adhesion	19/349	493/18870	0.00219222699410417	0.0269421292716569	0.0202914379765105	CCL5/CEBPB/CHST2/CXCL13/EFNB1/EPCAM/GLI2/GPNMB/HLA-DRA/HLA-DRB1/NRARP/PRNP/RUNX3/SOCS1/SOX12/SOX4/TFRC/TWSG1/ZNF703	19

BP	GO:0045216	cell-cell junction organization	11/349	214/18870	0.00220058603038111	0.0269421292716569	0.0202914379765105	CLDN1/DSG2/ECT2/F2R/F2RL1/FSCN1/GJA1/PAK2/PLEC/SNAI2/ZNF703	11

BP	GO:0060541	respiratory system development	11/349	214/18870	0.00220058603038111	0.0269421292716569	0.0202914379765105	BASP1/COL3A1/DLX5/GLI2/LOX/MMP12/MSC/SLC7A11/SOX9/TNC/WNT7B	11

BP	GO:0072089	stem cell proliferation	8/349	125/18870	0.00228015592631953	0.0278317214279245	0.0209614334279199	CD109/EIF2AK2/EPCAM/GJA1/SNAI2/SOX9/TP63/WNT7B	8

BP	GO:0031667	response to nutrient levels	19/349	495/18870	0.00229332268937811	0.02790786644355	0.0210187819710215	COL1A1/CXCL10/EIF2AK2/GCLM/HSPA5/ITGA2/PLEC/POSTN/SESN3/SLC16A1/SLC7A5/SNAI2/SPP1/STAT1/STC1/TFRC/TNC/XPR1/YWHAG	19

BP	GO:0051896	regulation of phosphatidylinositol 3-kinase/protein kinase B signal transduction	12/349	248/18870	0.00233596663435519	0.0283411855517552	0.0213451358281917	CCL5/EGFR/F2R/F2RL1/HSP90AA1/HSP90AB1/MFHAS1/MTDH/NTS/SESN3/SOX9/TREM2	12

BP	GO:2000027	regulation of animal organ morphogenesis	8/349	126/18870	0.00239650633143849	0.0287454840298981	0.0216496328265736	CTHRC1/FZD1/FZD2/FZD6/FZD7/HOXB7/SNAI2/SOX9	8

BP	GO:0022411	cellular component disassembly	19/349	497/18870	0.00239831684647438	0.0287454840298981	0.0216496328265736	C1QA/C1QB/C1QC/F2RL1/FSCN1/HIF1A/MMP1/MMP10/MMP11/MMP12/MMP13/MMP3/MMP7/MMP9/SNAI2/TOP2A/TPX2/TREM2/WDR72	19

BP	GO:0040013	negative regulation of locomotion	17/349	423/18870	0.00240060312400217	0.0287454840298981	0.0216496328265736	BST2/CLIC4/COL3A1/CXCL13/EPPK1/GJA1/GNA12/GNA13/IGFBP3/IL27RA/PFN2/PTPRK/SEMA3C/SERPINE1/STC1/TNFAIP6/TRIB1	17

BP	GO:0010820	positive regulation of T cell chemotaxis	3/349	15/18870	0.00241924505614088	0.0287454840298981	0.0216496328265736	CCL5/CXCL13/S100A7	3

BP	GO:0034144	negative regulation of toll-like receptor 4 signaling pathway	3/349	15/18870	0.00241924505614088	0.0287454840298981	0.0216496328265736	MFHAS1/TNFAIP3/TREM2	3

BP	GO:0060099	regulation of phagocytosis, engulfment	3/349	15/18870	0.00241924505614088	0.0287454840298981	0.0216496328265736	F2RL1/ITGA2/TREM2	3

BP	GO:1905153	regulation of membrane invagination	3/349	15/18870	0.00241924505614088	0.0287454840298981	0.0216496328265736	F2RL1/ITGA2/TREM2	3

BP	GO:0003338	metanephros morphogenesis	4/349	31/18870	0.00243902875257732	0.0288105801037579	0.0216986598701276	BASP1/SOX9/STAT1/WNT7B	4

BP	GO:0034505	tooth mineralization	4/349	31/18870	0.00243902875257732	0.0288105801037579	0.0216986598701276	AMTN/ASPN/COL1A1/WDR72	4

BP	GO:0050890	cognition	14/349	317/18870	0.00244768545950486	0.0288282954119461	0.0217120021369437	APP/EGFR/EGR2/EN1/FOSL1/HIF1A/HLA-DRA/ITPR3/POMK/PPT1/PRNP/SLC1A4/SLC7A11/TREM2	14

BP	GO:2000146	negative regulation of cell motility	16/349	388/18870	0.00247510002950737	0.0289656910002231	0.021815481488173	BST2/CLIC4/COL3A1/CXCL13/EPPK1/GJA1/GNA12/GNA13/IGFBP3/IL27RA/PFN2/PTPRK/SERPINE1/STC1/TNFAIP6/TRIB1	16

BP	GO:0007565	female pregnancy	10/349	186/18870	0.0024932706269643	0.0289656910002231	0.021815481488173	DSG2/FOSB/FOSL1/GJA1/ITGA2/ITGB4/MMP9/PTHLH/SPP1/STC1	10

BP	GO:0032640	tumor necrosis factor production	10/349	186/18870	0.0024932706269643	0.0289656910002231	0.021815481488173	APP/CD14/ELF4/FCGR3A/GPNMB/IL27RA/OAS2/OAS3/TNFAIP3/TREM2	10

BP	GO:0032680	regulation of tumor necrosis factor production	10/349	186/18870	0.0024932706269643	0.0289656910002231	0.021815481488173	APP/CD14/ELF4/FCGR3A/GPNMB/IL27RA/OAS2/OAS3/TNFAIP3/TREM2	10

BP	GO:0050803	regulation of synapse structure or activity	12/349	250/18870	0.00249530654843034	0.0289656910002231	0.021815481488173	AGRN/APP/CLSTN1/FZD1/NEFL/PPT1/PRNP/SLC7A11/SRGN/THBS2/TREM2/TUBB	12

BP	GO:0046683	response to organophosphorus	8/349	127/18870	0.00251745966269202	0.0290493297396248	0.0218784739219147	AHR/COL1A1/FOSB/FOSL1/NT5E/PANX1/STAT1/STC1	8

BP	GO:0030010	establishment of cell polarity	9/349	156/18870	0.00252414732097038	0.0290493297396248	0.0218784739219147	FSCN1/GJA1/HSP90AA1/HSP90AB1/MAPRE1/MISP/MSN/MYH9/SIPA1L3	9

BP	GO:0097530	granulocyte migration	9/349	156/18870	0.00252414732097038	0.0290493297396248	0.0218784739219147	CCL18/CCL5/CXCL10/CXCL13/CXCL8/CXCL9/FCER1G/S100A7/TNFAIP6	9

BP	GO:0002764	immune response-regulating signaling pathway	19/349	500/18870	0.00256337378598462	0.0294167225354588	0.0221551754422182	CD14/DDX60/EIF2AK2/F2RL1/FCER1G/FCGR3A/FOSL1/HLA-DRB1/IGHG2/IGHG3/IGHG4/MFHAS1/OAS3/PAK2/PRNP/RSAD2/SLC39A6/TNFAIP3/TREM2	19

BP	GO:0009914	hormone transport	14/349	319/18870	0.00259136635093634	0.0296534763112829	0.0223334863139908	ANO1/CCL5/F2RL1/GAL/GJA1/HIF1A/HLA-DRB1/INHBA/IRS2/NMB/SLC16A1/SLC7A5/SOX4/SPP1	14

BP	GO:0001678	intracellular glucose homeostasis	9/349	157/18870	0.00263519910002959	0.0300696373227172	0.022646917567572	ANO1/GCLM/HIF1A/HLA-DRB1/ICAM1/IRS2/NUCKS1/SOX4/TREM2	9

BP	GO:0010951	negative regulation of endopeptidase activity	8/349	128/18870	0.00264313967950693	0.0300750469747285	0.0226509918416264	CST1/IFI6/LAMP3/MAGEA3/MMP9/PAK2/SERPINE1/SERPINH1	8

BP	GO:0007528	neuromuscular junction development	5/349	52/18870	0.00268086603896504	0.0303329449577281	0.0228452274520912	AGRN/APP/COL4A1/F2R/TNC	5

BP	GO:0043154	negative regulation of cysteine-type endopeptidase activity involved in apoptotic process	5/349	52/18870	0.00268086603896504	0.0303329449577281	0.0228452274520912	IFI6/LAMP3/MAGEA3/MMP9/PAK2	5

BP	GO:1990266	neutrophil migration	8/349	129/18870	0.00277367147782114	0.0312756447558445	0.0235552208713594	CCL18/CCL5/CXCL10/CXCL13/CXCL8/CXCL9/FCER1G/TNFAIP6	8

BP	GO:0010721	negative regulation of cell development	13/349	287/18870	0.00277971222010733	0.0312756447558445	0.0235552208713594	C1QC/CDK6/INHBA/NRARP/PTHLH/RUNX3/SEMA3C/SNAI2/SOCS1/SPP1/TNFAIP6/TREM2/TRIB1	13

BP	GO:0070167	regulation of biomineral tissue development	7/349	102/18870	0.00286749576841648	0.0321734622707008	0.0242314112434432	AMTN/ASPN/CEBPB/HIF1A/ISG15/SOX9/SRGN	7

BP	GO:0002762	negative regulation of myeloid leukocyte differentiation	5/349	53/18870	0.00291605184215998	0.0325369995019955	0.0245052089491327	C1QC/CDK6/INHBA/TNFAIP6/TRIB1	5

BP	GO:0090175	regulation of establishment of planar polarity	5/349	53/18870	0.00291605184215998	0.0325369995019955	0.0245052089491327	CTHRC1/FZD1/FZD2/FZD6/FZD7	5

BP	GO:0042249	establishment of planar polarity of embryonic epithelium	3/349	16/18870	0.00293702330867581	0.0325904404610087	0.0245454579544836	CTHRC1/FZD1/FZD2	3

BP	GO:0048532	anatomical structure arrangement	3/349	16/18870	0.00293702330867581	0.0325904404610087	0.0245454579544836	EGR2/HSPA5/KIF14	3

BP	GO:0150063	visual system development	16/349	395/18870	0.00295151841927718	0.0326613082221112	0.024598831938568	C1QA/CLIC4/COL4A1/COL5A1/COL5A2/EGFR/FJX1/HIF1A/INHBA/PXDN/SIPA1L3/SLC7A11/SOX9/TWSG1/WNT7B/XRN2	16

BP	GO:0017015	regulation of transforming growth factor beta receptor signaling pathway	9/349	160/18870	0.00299174231433052	0.0330157206633517	0.0248657573176657	ASPN/CD109/CHST11/HSP90AB1/HSPA5/HTRA3/LOX/PMEPA1/ZNF703	9

BP	GO:0071706	tumor necrosis factor superfamily cytokine production	10/349	191/18870	0.00301897897662009	0.0331347338360374	0.0249553919707991	APP/CD14/ELF4/FCGR3A/GPNMB/IL27RA/OAS2/OAS3/TNFAIP3/TREM2	10

BP	GO:1903555	regulation of tumor necrosis factor superfamily cytokine production	10/349	191/18870	0.00301897897662009	0.0331347338360374	0.0249553919707991	APP/CD14/ELF4/FCGR3A/GPNMB/IL27RA/OAS2/OAS3/TNFAIP3/TREM2	10

BP	GO:0045639	positive regulation of myeloid cell differentiation	7/349	103/18870	0.00302892819543997	0.033153594487044	0.0249695968514245	HIF1A/HLA-DRB1/INHBA/ISG15/STAT1/TREM2/TRIB1	7

BP	GO:0003382	epithelial cell morphogenesis	4/349	33/18870	0.00308132631703717	0.0333644688307143	0.0251284166545023	CLIC4/MET/SIPA1L3/STC1	4

BP	GO:0043618	regulation of transcription from RNA polymerase II promoter in response to stress	4/349	33/18870	0.00308132631703717	0.0333644688307143	0.0251284166545023	CEBPB/CREB3L1/HIF1A/HSPA5	4

BP	GO:0045648	positive regulation of erythrocyte differentiation	4/349	33/18870	0.00308132631703717	0.0333644688307143	0.0251284166545023	HIF1A/INHBA/ISG15/STAT1	4

BP	GO:0061436	establishment of skin barrier	4/349	33/18870	0.00308132631703717	0.0333644688307143	0.0251284166545023	CLDN1/MET/PLEC/TP63	4

BP	GO:0061326	renal tubule development	7/349	104/18870	0.00319722230249809	0.0344342551723591	0.0259341251781579	COL4A1/HOXB7/HOXD11/IRX2/SOX9/STAT1/WNT7B	7

BP	GO:1990830	cellular response to leukemia inhibitory factor	7/349	104/18870	0.00319722230249809	0.0344342551723591	0.0259341251781579	GCLM/ICAM1/MSC/MYBL2/SNX10/SOCS3/TFRC	7

BP	GO:0002757	immune response-activating signaling pathway	18/349	473/18870	0.00324313091260293	0.034814989995177	0.02622087523287	CD14/DDX60/EIF2AK2/F2RL1/FCER1G/FOSL1/HLA-DRB1/IGHG2/IGHG3/IGHG4/MFHAS1/OAS3/PAK2/PRNP/RSAD2/SLC39A6/TNFAIP3/TREM2	18

BP	GO:0021915	neural tube development	9/349	162/18870	0.00324986003927174	0.034814989995177	0.02622087523287	CTHRC1/EN1/FZD1/FZD2/FZD6/GLI2/HIF1A/MARCKS/SEMA3C	9

BP	GO:0050868	negative regulation of T cell activation	8/349	133/18870	0.00334686303432994	0.0356644558261402	0.0268606495821189	CEBPB/GPNMB/HLA-DRB1/NRARP/PRNP/RUNX3/SOCS1/TWSG1	8

BP	GO:0051225	spindle assembly	8/349	133/18870	0.00334686303432994	0.0356644558261402	0.0268606495821189	KIF11/KIF4A/MAPRE1/MISP/MYBL2/MZT1/TPX2/TUBB	8

BP	GO:0044409	entry into host	9/349	163/18870	0.00338533016821408	0.0357903147442686	0.0269554401016521	CLDN1/CXCL8/EGFR/HLA-DRB1/ICAM1/ITGA2/ITGAV/TFRC/XPR1	9

BP	GO:1902850	microtubule cytoskeleton organization involved in mitosis	9/349	163/18870	0.00338533016821408	0.0357903147442686	0.0269554401016521	DLGAP5/GJA1/KIF11/KIF4A/MAPRE1/MISP/MYBL2/MZT1/TPX2	9

BP	GO:1903844	regulation of cellular response to transforming growth factor beta stimulus	9/349	163/18870	0.00338533016821408	0.0357903147442686	0.0269554401016521	ASPN/CD109/CHST11/HSP90AB1/HSPA5/HTRA3/LOX/PMEPA1/ZNF703	9

BP	GO:0048880	sensory system development	16/349	401/18870	0.00341813973352024	0.0358250187242407	0.0269815773697961	C1QA/CLIC4/COL4A1/COL5A1/COL5A2/EGFR/FJX1/HIF1A/INHBA/PXDN/SIPA1L3/SLC7A11/SOX9/TWSG1/WNT7B/XRN2	16

BP	GO:0034142	toll-like receptor 4 signaling pathway	5/349	55/18870	0.00343056790247408	0.0358250187242407	0.0269815773697961	CD14/F2RL1/MFHAS1/TNFAIP3/TREM2	5

BP	GO:0035025	positive regulation of Rho protein signal transduction	4/349	34/18870	0.0034419767244988	0.0358250187242407	0.0269815773697961	COL3A1/F2R/F2RL1/PRAG1	4

BP	GO:0048333	mesodermal cell differentiation	4/349	34/18870	0.0034419767244988	0.0358250187242407	0.0269815773697961	GJA1/INHBA/ITGA2/ITGB4	4

BP	GO:0048665	neuron fate specification	4/349	34/18870	0.0034419767244988	0.0358250187242407	0.0269815773697961	GLI2/HOXC10/HOXD10/SOX9	4

BP	GO:0090183	regulation of kidney development	4/349	34/18870	0.0034419767244988	0.0358250187242407	0.0269815773697961	BASP1/HOXB7/SOX9/STAT1	4

BP	GO:0042742	defense response to bacterium	14/349	330/18870	0.00351125772071725	0.0359648303855641	0.0270868763840474	CEBPB/CXCL13/F2RL1/FCER1G/IGHG2/IGHG3/IGHG4/IGKV3-20/IL27RA/ISG15/OAS2/OAS3/SERPINE1/TREM2	14

BP	GO:0048545	response to steroid hormone	14/349	330/18870	0.00351125772071725	0.0359648303855641	0.0270868763840474	CLDN1/COL1A1/DSG2/FOSB/FOSL1/INHBA/LOX/NEFL/PCNA/PMEPA1/SPP1/STC1/TP63/WNT7B	14

BP	GO:0002076	osteoblast development	3/349	17/18870	0.0035179104200378	0.0359648303855641	0.0270868763840474	GLI2/JUND/PTHLH	3

BP	GO:0009163	nucleoside biosynthetic process	3/349	17/18870	0.0035179104200378	0.0359648303855641	0.0270868763840474	HPRT1/NT5E/TK1	3

BP	GO:0032905	transforming growth factor beta1 production	3/349	17/18870	0.0035179104200378	0.0359648303855641	0.0270868763840474	COL3A1/LAPTM4B/LUM	3

BP	GO:0034404	nucleobase-containing small molecule biosynthetic process	3/349	17/18870	0.0035179104200378	0.0359648303855641	0.0270868763840474	HPRT1/NT5E/TK1	3

BP	GO:0060644	mammary gland epithelial cell differentiation	3/349	17/18870	0.0035179104200378	0.0359648303855641	0.0270868763840474	CEBPB/HIF1A/ZNF703	3

BP	GO:0030316	osteoclast differentiation	7/349	106/18870	0.00355514573363845	0.0361619369068073	0.0272353269653004	CD109/CEBPB/GLO1/SNX10/TFRC/TNFAIP6/TREM2	7

BP	GO:1990823	response to leukemia inhibitory factor	7/349	106/18870	0.00355514573363845	0.0361619369068073	0.0272353269653004	GCLM/ICAM1/MSC/MYBL2/SNX10/SOCS3/TFRC	7

BP	GO:0007163	establishment or maintenance of cell polarity	11/349	228/18870	0.00357965703614153	0.0363195429258894	0.0273540277824735	CLIC4/FSCN1/GJA1/HSP90AA1/HSP90AB1/MAPRE1/MISP/MSN/MYH9/SIPA1L3/WNT7B	11

BP	GO:0000280	nuclear division	17/349	441/18870	0.00366758081777428	0.0371181294823989	0.0279554824565603	ANLN/CCNA1/CKS2/DLGAP5/KIF11/KIF14/KIF4A/MAPRE1/MISP/MYBL2/MZT1/NCAPD2/REEP3/TOP2A/TPX2/TRIP13/UBE2C	17

BP	GO:0097191	extrinsic apoptotic signaling pathway	11/349	229/18870	0.00369991937179777	0.0373515669914822	0.0281312956853216	GCLM/ICAM1/IFI6/INHBA/ITGA6/ITGAV/PAK2/SERPINE1/SNAI2/TNFAIP3/TNFRSF12A	11

BP	GO:0002833	positive regulation of response to biotic stimulus	15/349	369/18870	0.0038121366884099	0.0382262656377872	0.0287900741044538	CCL5/CD14/DDX60/EIF2AK2/F2RL1/FOSL1/HLA-DRB1/HSP90AA1/MFHAS1/MMP12/OAS3/PAK2/RSAD2/TNFAIP3/TREM2	15

BP	GO:0002495	antigen processing and presentation of peptide antigen via MHC class II	4/349	35/18870	0.00383028385646415	0.0382262656377872	0.0287900741044538	FCER1G/HLA-DRA/HLA-DRB1/TREM2	4

BP	GO:0032633	interleukin-4 production	4/349	35/18870	0.00383028385646415	0.0382262656377872	0.0287900741044538	CEBPB/FCER1G/HLA-DRB1/SLC7A5	4

BP	GO:0032673	regulation of interleukin-4 production	4/349	35/18870	0.00383028385646415	0.0382262656377872	0.0287900741044538	CEBPB/FCER1G/HLA-DRB1/SLC7A5	4

BP	GO:0071333	cellular response to glucose stimulus	8/349	136/18870	0.00383401472633219	0.0382262656377872	0.0287900741044538	ANO1/GCLM/HIF1A/HLA-DRB1/ICAM1/IRS2/SOX4/TREM2	8

BP	GO:0007422	peripheral nervous system development	6/349	81/18870	0.00388552074288842	0.038548959488558	0.0290331106585544	CLDN1/EGR2/HOXD10/ITGB4/PLEC/RUNX3	6

BP	GO:0008344	adult locomotory behavior	6/349	81/18870	0.00388552074288842	0.038548959488558	0.0290331106585544	APP/EN1/GRIN2D/HOXD10/PPT1/UCHL1	6

BP	GO:0030900	forebrain development	16/349	407/18870	0.0039440173103828	0.039033173774501	0.0293977961684294	APP/CDK6/COL3A1/DLX5/EGFR/GLI2/HIF1A/HPRT1/INHBA/KIF14/NEFL/PLXNA1/SLC7A11/TWSG1/WNT7B/XRN2	16

BP	GO:0043122	regulation of canonical NF-kappaB signal transduction	12/349	265/18870	0.00399418346938593	0.0393762707140262	0.0296561992886175	BST2/DDX21/ECT2/F2R/F2RL1/GJA1/HLA-DRB1/MTDH/STAT1/TFRC/TNFAIP3/TREM2	12

BP	GO:0007266	Rho protein signal transduction	8/349	137/18870	0.00400801166652203	0.0393762707140262	0.0296561992886175	COL1A2/COL3A1/F2R/F2RL1/GNA12/GNA13/MET/PRAG1	8

BP	GO:0010466	negative regulation of peptidase activity	8/349	137/18870	0.00400801166652203	0.0393762707140262	0.0296561992886175	CST1/IFI6/LAMP3/MAGEA3/MMP9/PAK2/SERPINE1/SERPINH1	8

BP	GO:1902075	cellular response to salt	10/349	199/18870	0.00404052747751335	0.0395991354730505	0.0298240496611435	AGRN/CCNA2/CLDN1/CLIC4/COL1A1/ECT2/FOSB/GCLM/JUND/PLEC	10

BP	GO:0034329	cell junction assembly	17/349	446/18870	0.00410416701593167	0.040125205680031	0.0302202589164923	AGRN/APP/CLDN1/CLSTN1/ECT2/FSCN1/FZD1/GJA1/ITGA2/ITGA6/ITGB4/PAK2/PLEC/PTPRK/SNAI2/THBS2/ZNF703	17

BP	GO:0019896	axonal transport of mitochondrion	3/349	18/18870	0.0041641669736883	0.0403203475240781	0.030367229803061	HIF1A/NEFL/UCHL1	3

BP	GO:0035455	response to interferon-alpha	3/349	18/18870	0.0041641669736883	0.0403203475240781	0.030367229803061	BST2/EIF2AK2/LAMP3	3

BP	GO:0071371	cellular response to gonadotropin stimulus	3/349	18/18870	0.0041641669736883	0.0403203475240781	0.030367229803061	CCNA2/GCLM/INHBA	3

BP	GO:1904031	positive regulation of cyclin-dependent protein kinase activity	3/349	18/18870	0.0041641669736883	0.0403203475240781	0.030367229803061	CCND1/EGFR/HSP90AB1	3

BP	GO:0071331	cellular response to hexose stimulus	8/349	138/18870	0.00418803982997861	0.0404542552401771	0.030468082266814	ANO1/GCLM/HIF1A/HLA-DRB1/ICAM1/IRS2/SOX4/TREM2	8

BP	GO:0045589	regulation of regulatory T cell differentiation	4/349	36/18870	0.00424721536769885	0.0408300322221741	0.0307510983286222	HLA-DRA/HLA-DRB1/SOCS1/SOX12	4

BP	GO:0071711	basement membrane organization	4/349	36/18870	0.00424721536769885	0.0408300322221741	0.0307510983286222	COL3A1/COL4A1/MMP11/PXDN	4

BP	GO:0042063	gliogenesis	14/349	338/18870	0.00433534992843451	0.0415780702660338	0.0313144824404718	ANO1/APP/C1QA/CDK6/COL3A1/CSPG4/EGR2/ITGB4/PLEC/SLC7A5/SOX4/SOX9/TREM2/VCAN	14

BP	GO:0061844	antimicrobial humoral immune response mediated by antimicrobial peptide	6/349	83/18870	0.00438258941889046	0.0419312830862013	0.0315805043261465	CXCL10/CXCL13/CXCL14/CXCL8/CXCL9/S100A7	6

BP	GO:0007051	spindle organization	10/349	202/18870	0.0044874187318201	0.0427659651860232	0.0322091443229287	DLGAP5/KIF11/KIF4A/MAPRE1/MISP/MYBL2/MYH9/MZT1/TPX2/TUBB	10

BP	GO:0045861	negative regulation of proteolysis	11/349	235/18870	0.00449106337479837	0.0427659651860232	0.0322091443229287	CST1/HSP90AB1/IFI6/LAMP3/MAGEA3/MMP9/PAK2/PLAU/PRNP/SERPINE1/SERPINH1	11

BP	GO:0000086	G2/M transition of mitotic cell cycle	8/349	140/18870	0.00456673558960197	0.0431344001088934	0.0324866307201774	APP/CCNA2/CCND1/CDK6/CLSPN/DTL/FOXM1/KIF14	8

BP	GO:0071326	cellular response to monosaccharide stimulus	8/349	140/18870	0.00456673558960197	0.0431344001088934	0.0324866307201774	ANO1/GCLM/HIF1A/HLA-DRB1/ICAM1/IRS2/SOX4/TREM2	8

BP	GO:0007229	integrin-mediated signaling pathway	7/349	111/18870	0.00458329772755869	0.0431344001088934	0.0324866307201774	COL3A1/ISG15/ITGA2/ITGA6/ITGAV/ITGB4/MYH9	7

BP	GO:0032611	interleukin-1 beta production	7/349	111/18870	0.00458329772755869	0.0431344001088934	0.0324866307201774	APP/ELF4/F2R/F2RL1/PANX1/TNFAIP3/TREM2	7

BP	GO:0032651	regulation of interleukin-1 beta production	7/349	111/18870	0.00458329772755869	0.0431344001088934	0.0324866307201774	APP/ELF4/F2R/F2RL1/PANX1/TNFAIP3/TREM2	7

BP	GO:0001959	regulation of cytokine-mediated signaling pathway	9/349	171/18870	0.00463588322246413	0.0435275935200128	0.0327827639483293	CCL5/F2RL1/HIF1A/ISG15/MMP12/OAS3/PXDN/TNFAIP3/TREM2	9

BP	GO:0002504	antigen processing and presentation of peptide or polysaccharide antigen via MHC class II	4/349	37/18870	0.00469371811676301	0.043866117341813	0.0330377228294321	FCER1G/HLA-DRA/HLA-DRB1/TREM2	4

BP	GO:0051385	response to mineralocorticoid	4/349	37/18870	0.00469371811676301	0.043866117341813	0.0330377228294321	FOSB/FOSL1/INHBA/NEFL	4

BP	GO:0002544	chronic inflammatory response	3/349	19/18870	0.00487786190019946	0.0449611618627081	0.0338624545257735	CCL5/CXCL13/TNFAIP3	3

BP	GO:0051797	regulation of hair follicle development	3/349	19/18870	0.00487786190019946	0.0449611618627081	0.0338624545257735	FST/GAL/KRT17	3

BP	GO:0060231	mesenchymal to epithelial transition	3/349	19/18870	0.00487786190019946	0.0449611618627081	0.0338624545257735	BASP1/FZD7/STAT1	3

BP	GO:0060602	branch elongation of an epithelium	3/349	19/18870	0.00487786190019946	0.0449611618627081	0.0338624545257735	HOXD13/SOX9/TNC	3

BP	GO:0061298	retina vasculature development in camera-type eye	3/349	19/18870	0.00487786190019946	0.0449611618627081	0.0338624545257735	CLIC4/COL4A1/HIF1A	3

BP	GO:1903376	regulation of oxidative stress-induced neuron intrinsic apoptotic signaling pathway	3/349	19/18870	0.00487786190019946	0.0449611618627081	0.0338624545257735	FZD1/HIF1A/TREM2	3

BP	GO:0043087	regulation of GTPase activity	13/349	307/18870	0.00490109119185237	0.0449899530436921	0.0338841385752158	AGRN/CCL18/CCL5/CXCL13/ECT2/F2R/F2RL1/FGD6/ITGA6/MET/PLXNA1/SH3BP4/SIPA1L3	13

BP	GO:0051591	response to cAMP	6/349	85/18870	0.00492505921474892	0.0449899530436921	0.0338841385752158	AHR/COL1A1/FOSB/FOSL1/STAT1/STC1	6

BP	GO:0045089	positive regulation of innate immune response	14/349	343/18870	0.00492566268427712	0.0449899530436921	0.0338841385752158	CCL5/CD14/DDX60/EIF2AK2/F2RL1/FOSL1/HSP90AA1/MFHAS1/MMP12/OAS3/PAK2/RSAD2/TNFAIP3/TREM2	14

BP	GO:0070482	response to oxygen levels	14/349	343/18870	0.00492566268427712	0.0449899530436921	0.0338841385752158	CA9/CCNA2/COL1A1/HIF1A/ITGA2/LOXL2/MALAT1/PLAU/POSTN/SLC7A5/SOX4/STC1/TFRC/TREM2	14

BP	GO:0022408	negative regulation of cell-cell adhesion	10/349	205/18870	0.00497237605045899	0.0453138704326896	0.03412809663592	CEBPB/EPCAM/GPNMB/HLA-DRB1/NRARP/PRNP/RUNX3/SOCS1/TWSG1/ZNF703	10

BP	GO:0007566	embryo implantation	5/349	60/18870	0.0049975924851157	0.0454408634989752	0.0342237413380146	GJA1/ITGB4/MMP9/SPP1/STC1	5

BP	GO:0046879	hormone secretion	13/349	308/18870	0.0050342274749273	0.0456708744797459	0.034396973880371	ANO1/CCL5/F2RL1/GAL/GJA1/HIF1A/HLA-DRB1/INHBA/IRS2/NMB/SLC16A1/SOX4/SPP1	13

BP	GO:0032355	response to estradiol	7/349	113/18870	0.00505195300811453	0.0457286892509783	0.0344405170180629	CCNA2/COL1A1/EGFR/ITGA2/PCNA/POSTN/ZNF703	7

BP	GO:0007160	cell-matrix adhesion	11/349	239/18870	0.00508930091322282	0.0459634620593308	0.0346173359261463	CDK6/COL3A1/ITGA2/ITGA6/ITGAV/ITGB4/MMP12/PLAU/POSTN/PTPRK/SERPINE1	11

BP	GO:0050863	regulation of T cell activation	15/349	381/18870	0.00510403433039099	0.0459934010801228	0.0346398844700031	CCL5/CEBPB/EFNB1/GLI2/GPNMB/HLA-DRA/HLA-DRB1/NRARP/PRNP/RUNX3/SOCS1/SOX12/SOX4/TFRC/TWSG1	15

BP	GO:0044703	multi-organism reproductive process	10/349	206/18870	0.00514286007267463	0.0461810379406616	0.0347812029856757	DSG2/FOSB/FOSL1/GJA1/ITGA2/ITGB4/MMP9/PTHLH/SPP1/STC1	10

BP	GO:0032965	regulation of collagen biosynthetic process	4/349	38/18870	0.00517071700874836	0.0461810379406616	0.0347812029856757	CREB3L1/F2R/INHBA/ITGA2	4

BP	GO:0060563	neuroepithelial cell differentiation	4/349	38/18870	0.00517071700874836	0.0461810379406616	0.0347812029856757	CEBPB/SERPINE1/SOX4/TUBB	4

BP	GO:0071392	cellular response to estradiol stimulus	4/349	38/18870	0.00517071700874836	0.0461810379406616	0.0347812029856757	CCNA2/EGFR/ITGA2/ZNF703	4

BP	GO:0014074	response to purine-containing compound	8/349	143/18870	0.0051834462660301	0.0461923043353302	0.0347896882596617	AHR/COL1A1/FOSB/FOSL1/NT5E/PANX1/STAT1/STC1	8

BP	GO:0007611	learning or memory	12/349	274/18870	0.00519531907296768	0.0461959055759687	0.0347924005304818	APP/EGFR/EGR2/EN1/FOSL1/HIF1A/ITPR3/POMK/PPT1/PRNP/SLC7A11/TREM2	12

BP	GO:2001243	negative regulation of intrinsic apoptotic signaling pathway	7/349	114/18870	0.0052993611818925	0.0469046274732171	0.0353261737254512	ACKR3/CREB3L1/FZD1/HIF1A/MAGEA3/MMP9/SNAI2	7

BP	GO:0002768	immune response-regulating cell surface receptor signaling pathway	14/349	346/18870	0.00530995782715665	0.0469046274732171	0.0353261737254512	CD14/F2RL1/FCER1G/FCGR3A/HLA-DRB1/IGHG2/IGHG3/IGHG4/MFHAS1/PAK2/PRNP/SLC39A6/TNFAIP3/TREM2	14

BP	GO:0071496	cellular response to external stimulus	14/349	346/18870	0.00530995782715665	0.0469046274732171	0.0353261737254512	COL1A1/EIF2AK2/FOSL1/HSPA5/ITGA2/PLEC/POSTN/SESN3/SLC7A5/SNAI2/SOX9/TNC/XPR1/YWHAG	14

BP	GO:0002218	activation of innate immune response	12/349	276/18870	0.00549778370085036	0.048400002090204	0.036452413637163	CD14/DDX60/EIF2AK2/F2RL1/FOSL1/HSP90AA1/MFHAS1/OAS3/PAK2/RSAD2/TNFAIP3/TREM2	12

BP	GO:0010232	vascular transport	6/349	87/18870	0.00551529318753814	0.048400002090204	0.036452413637163	ABCC1/SLC16A1/SLC1A4/SLC44A1/SLC7A5/TFRC	6

BP	GO:0150104	transport across blood-brain barrier	6/349	87/18870	0.00551529318753814	0.048400002090204	0.036452413637163	ABCC1/SLC16A1/SLC1A4/SLC44A1/SLC7A5/TFRC	6

BP	GO:0062207	regulation of pattern recognition receptor signaling pathway	8/349	145/18870	0.00562865760970726	0.0489313862147535	0.0368526250642658	CD14/DDX60/EIF2AK2/F2RL1/MFHAS1/RSAD2/TNFAIP3/TREM2	8

BP	GO:0002396	MHC protein complex assembly	3/349	20/18870	0.00566088033169691	0.0489313862147535	0.0368526250642658	HLA-DRA/HLA-DRB1/TAPBP	3

BP	GO:0002501	peptide antigen assembly with MHC protein complex	3/349	20/18870	0.00566088033169691	0.0489313862147535	0.0368526250642658	HLA-DRA/HLA-DRB1/TAPBP	3

BP	GO:0003417	growth plate cartilage development	3/349	20/18870	0.00566088033169691	0.0489313862147535	0.0368526250642658	MMP13/SOX9/STC1	3

BP	GO:0072567	chemokine (C-X-C motif) ligand 2 production	3/349	20/18870	0.00566088033169691	0.0489313862147535	0.0368526250642658	F2RL1/OAS3/POSTN	3

BP	GO:0140131	positive regulation of lymphocyte chemotaxis	3/349	20/18870	0.00566088033169691	0.0489313862147535	0.0368526250642658	CCL5/CXCL13/S100A7	3

BP	GO:2000341	regulation of chemokine (C-X-C motif) ligand 2 production	3/349	20/18870	0.00566088033169691	0.0489313862147535	0.0368526250642658	F2RL1/OAS3/POSTN	3

BP	GO:0043620	regulation of DNA-templated transcription in response to stress	4/349	39/18870	0.00567911393740028	0.048983877815521	0.0368921590613943	CEBPB/CREB3L1/HIF1A/HSPA5	4

BP	GO:0032635	interleukin-6 production	9/349	177/18870	0.00578963250490702	0.0495347131456215	0.0373070201446928	APP/CEBPB/ELF4/F2R/F2RL1/IL27RA/NMB/TNFAIP3/TREM2	9

BP	GO:0032675	regulation of interleukin-6 production	9/349	177/18870	0.00578963250490702	0.0495347131456215	0.0373070201446928	APP/CEBPB/ELF4/F2R/F2RL1/IL27RA/NMB/TNFAIP3/TREM2	9

BP	GO:0019882	antigen processing and presentation	7/349	116/18870	0.00582132409957775	0.0495347131456215	0.0373070201446928	FCER1G/HLA-DRA/HLA-DRB1/ICAM1/RAB32/TAPBP/TREM2	7

BP	GO:0045582	positive regulation of T cell differentiation	7/349	116/18870	0.00582132409957775	0.0495347131456215	0.0373070201446928	GLI2/HLA-DRA/HLA-DRB1/RUNX3/SOCS1/SOX12/SOX4	7

BP	GO:0001892	embryonic placenta development	6/349	88/18870	0.00582906008714613	0.0495347131456215	0.0373070201446928	CEBPB/EGFR/HIF1A/PHLDA2/SOCS3/WNT7B	6

BP	GO:0050672	negative regulation of lymphocyte proliferation	6/349	88/18870	0.00582906008714613	0.0495347131456215	0.0373070201446928	CEBPB/GAL/GPNMB/HLA-DRB1/PRNP/TWSG1	6

BP	GO:0098586	cellular response to virus	6/349	88/18870	0.00582906008714613	0.0495347131456215	0.0373070201446928	CCL5/CXCL10/HIF1A/HSP90AA1/IFI6/MMP12	6

BP	GO:0050807	regulation of synapse organization	11/349	244/18870	0.0059239465024857	0.0502350663410788	0.0378344904601968	AGRN/APP/CLSTN1/FZD1/NEFL/PRNP/SLC7A11/SRGN/THBS2/TREM2/TUBB	11

BP	GO:0051701	biological process involved in interaction with host	10/349	211/18870	0.00606517880031242	0.0513246643018035	0.0386551200851402	CLDN1/CXCL8/EGFR/HLA-DRB1/HSP90AB1/ICAM1/ITGA2/ITGAV/TFRC/XPR1	10

BP	GO:0009913	epidermal cell differentiation	11/349	245/18870	0.00610311158816704	0.0515373867445217	0.0388153317821856	CD109/CLIC4/KRT10/KRT14/KRT17/KRT75/PLEC/S100A7/SFRP4/SPRR2G/TP63	11

BP	GO:0140895	cell surface toll-like receptor signaling pathway	5/349	63/18870	0.00614999042512332	0.0516561911085387	0.038904809171277	CD14/F2RL1/MFHAS1/TNFAIP3/TREM2	5

BP	GO:0032945	negative regulation of mononuclear cell proliferation	6/349	89/18870	0.00615565335950809	0.0516561911085387	0.038904809171277	CEBPB/GAL/GPNMB/HLA-DRB1/PRNP/TWSG1	6

BP	GO:0046849	bone remodeling	6/349	89/18870	0.00615565335950809	0.0516561911085387	0.038904809171277	CTHRC1/GJA1/SNX10/SPP1/TFRC/TNFAIP3	6

BP	GO:0032728	positive regulation of interferon-beta production	4/349	40/18870	0.00621978682209294	0.0520858655288781	0.0392284180353909	HSP90AA1/ISG15/OAS2/OAS3	4

BP	GO:0031668	cellular response to extracellular stimulus	12/349	281/18870	0.00631547429178979	0.0527774490608491	0.0397492834875261	COL1A1/EIF2AK2/FOSL1/HSPA5/PLEC/POSTN/SESN3/SLC7A5/SNAI2/TNC/XPR1/YWHAG	12

BP	GO:0009314	response to radiation	16/349	428/18870	0.00633258710438884	0.0528108920423981	0.0397744710359565	APP/CCND1/COL3A1/CXCL10/DTL/ECT2/EGFR/HIF1A/MMP1/MMP3/MMP9/NUCKS1/PCNA/PTPRK/SLC7A11/SNAI2	16

BP	GO:0001654	eye development	15/349	391/18870	0.00643376901767443	0.053543846287588	0.0403264947997776	CLIC4/COL4A1/COL5A1/COL5A2/EGFR/FJX1/HIF1A/INHBA/PXDN/SIPA1L3/SLC7A11/SOX9/TWSG1/WNT7B/XRN2	15

BP	GO:0042098	T cell proliferation	10/349	213/18870	0.00646826885568129	0.0537199730941943	0.0404591445297309	CCL5/CEBPB/EFNB1/ELF4/GPNMB/HLA-DRB1/MSN/PRNP/TFRC/TWSG1	10

BP	GO:0030502	negative regulation of bone mineralization	3/349	21/18870	0.00651493120446017	0.0537748829745196	0.0405004998517131	HIF1A/SOX9/SRGN	3

BP	GO:0036480	neuron intrinsic apoptotic signaling pathway in response to oxidative stress	3/349	21/18870	0.00651493120446017	0.0537748829745196	0.0405004998517131	FZD1/HIF1A/TREM2	3

BP	GO:0060445	branching involved in salivary gland morphogenesis	3/349	21/18870	0.00651493120446017	0.0537748829745196	0.0405004998517131	PLXNA1/SEMA3C/SNAI2	3

BP	GO:0002429	immune response-activating cell surface receptor signaling pathway	13/349	318/18870	0.00653193989103578	0.0538050181617426	0.0405231961381232	CD14/F2RL1/FCER1G/HLA-DRB1/IGHG2/IGHG3/IGHG4/MFHAS1/PAK2/PRNP/SLC39A6/TNFAIP3/TREM2	13

BP	GO:0062208	positive regulation of pattern recognition receptor signaling pathway	5/349	64/18870	0.0065723853329404	0.0540276900430285	0.0406909012449715	CD14/DDX60/F2RL1/MFHAS1/RSAD2	5

BP	GO:0031960	response to corticosteroid	8/349	149/18870	0.00660593058404926	0.0541928480500008	0.0408152898343444	CLDN1/FOSB/FOSL1/INHBA/NEFL/PCNA/STC1/WNT7B	8

BP	GO:0007219	Notch signaling pathway	9/349	181/18870	0.00667409288772338	0.05442777173739	0.0409922223768248	APP/CDK6/DTX3L/NRARP/POSTN/PRAG1/SNAI2/SOX9/TP63	9

BP	GO:0046631	alpha-beta T cell activation	9/349	181/18870	0.00667409288772338	0.05442777173739	0.0409922223768248	ELF4/HLA-DRA/HLA-DRB1/PRR7/RSAD2/RUNX3/SOCS1/SOCS3/TWSG1	9

BP	GO:0035710	CD4-positive, alpha-beta T cell activation	7/349	119/18870	0.00667510408100066	0.05442777173739	0.0409922223768248	HLA-DRA/HLA-DRB1/RSAD2/RUNX3/SOCS1/SOCS3/TWSG1	7

BP	GO:0031589	cell-substrate adhesion	14/349	356/18870	0.00676882259934832	0.0550804392528789	0.0414837782696903	CDK6/COL1A1/COL3A1/FZD7/ITGA2/ITGA6/ITGAV/ITGB4/KIF14/MMP12/PLAU/POSTN/PTPRK/SERPINE1	14

BP	GO:0045066	regulatory T cell differentiation	4/349	41/18870	0.00679358873528747	0.05511758898496	0.0415117575536128	HLA-DRA/HLA-DRB1/SOCS1/SOX12	4

BP	GO:0044772	mitotic cell cycle phase transition	17/349	470/18870	0.00683672126203392	0.05511758898496	0.0415117575536128	ANLN/APP/CCNA1/CCNA2/CCND1/CDK6/CKS2/CLSPN/DLGAP5/DTL/EGFR/FOXM1/GPNMB/INHBA/KIF14/TRIP13/UBE2C	17

BP	GO:0002220	innate immune response activating cell surface receptor signaling pathway	6/349	91/18870	0.00684849436122079	0.05511758898496	0.0415117575536128	CD14/F2RL1/MFHAS1/PAK2/TNFAIP3/TREM2	6

BP	GO:0030901	midbrain development	6/349	91/18870	0.00684849436122079	0.05511758898496	0.0415117575536128	BASP1/EN1/FZD1/FZD6/HSPA5/YWHAQ	6

BP	GO:0002703	regulation of leukocyte mediated immunity	11/349	249/18870	0.0068627903968527	0.05511758898496	0.0415117575536128	AHR/BST2/DDX21/F2RL1/HLA-DRA/HLA-DRB1/ICAM1/IL27RA/RSAD2/TFRC/TREM2	11

BP	GO:0002832	negative regulation of response to biotic stimulus	8/349	150/18870	0.00686917320517624	0.05511758898496	0.0415117575536128	AHR/F2RL1/ISG15/MMP12/OAS3/TNFAIP3/TREM2/TRIB1	8

BP	GO:0072073	kidney epithelium development	8/349	150/18870	0.00686917320517624	0.05511758898496	0.0415117575536128	BASP1/EPCAM/HOXB7/HOXD11/IRX2/SOX9/STAT1/WNT7B	8

BP	GO:0072009	nephron epithelium development	7/349	120/18870	0.00697938043792768	0.0558905455347369	0.0420939090135138	BASP1/HOXB7/HOXD11/IRX2/SOX9/STAT1/WNT7B	7

BP	GO:0010812	negative regulation of cell-substrate adhesion	5/349	65/18870	0.0070146983415898	0.0560619145236582	0.0422229753977899	COL1A1/FZD7/MMP12/POSTN/SERPINE1	5

BP	GO:0031348	negative regulation of defense response	13/349	321/18870	0.00704439623355657	0.0561877782747839	0.0423177695572903	AHR/ELF4/F2RL1/HLA-DRB1/ISG15/MFHAS1/MMP12/NT5E/OAS3/SOCS3/TNFAIP3/TNFAIP6/TREM2	13

BP	GO:0044706	multi-multicellular organism process	10/349	216/18870	0.00711160257252462	0.0566117295694252	0.0426370680549531	DSG2/FOSB/FOSL1/GJA1/ITGA2/ITGB4/MMP9/PTHLH/SPP1/STC1	10

BP	GO:0071322	cellular response to carbohydrate stimulus	8/349	151/18870	0.00714027192165088	0.0567208994195155	0.0427192892194229	ANO1/GCLM/HIF1A/HLA-DRB1/ICAM1/IRS2/SOX4/TREM2	8

BP	GO:0060759	regulation of response to cytokine stimulus	9/349	183/18870	0.00715347986720801	0.0567208994195155	0.0427192892194229	CCL5/F2RL1/HIF1A/ISG15/MMP12/OAS3/PXDN/TNFAIP3/TREM2	9

BP	GO:0050886	endocrine process	6/349	92/18870	0.00721532743330346	0.0570406459065469	0.0429601059694424	F2R/F2RL1/GAL/GJA1/INHBA/SPP1	6

BP	GO:0044403	biological process involved in symbiotic interaction	13/349	322/18870	0.00722212746085872	0.0570406459065469	0.0429601059694424	CCL5/CLDN1/CXCL8/EGFR/F2RL1/HLA-DRB1/HSP90AB1/ICAM1/ITGA2/ITGAV/NUCKS1/TFRC/XPR1	13

BP	GO:0010038	response to metal ion	14/349	359/18870	0.00726392282191349	0.0571687466426485	0.0430565849119618	CD14/CLDN1/CLIC4/ECT2/EGFR/FOSB/HIF1A/ITPR3/JUND/LOXL2/MMP9/PCNA/PRNP/TFRC	14

BP	GO:0042593	glucose homeostasis	11/349	251/18870	0.00726939283040734	0.0571687466426485	0.0430565849119618	ANO1/GCLM/HIF1A/HLA-DRB1/ICAM1/IRS2/NUCKS1/SESN3/SLC16A1/SOX4/TREM2	11

BP	GO:0007595	lactation	4/349	42/18870	0.00740134711626054	0.0571687466426485	0.0430565849119618	CCND1/HIF1A/MTCO2P12/OAS2	4

BP	GO:0042119	neutrophil activation	4/349	42/18870	0.00740134711626054	0.0571687466426485	0.0430565849119618	CCL5/CXCL8/F2RL1/FCER1G	4

BP	GO:0071364	cellular response to epidermal growth factor stimulus	4/349	42/18870	0.00740134711626054	0.0571687466426485	0.0430565849119618	COL1A1/EGFR/SNAI2/SOX9	4

BP	GO:0071542	dopaminergic neuron differentiation	4/349	42/18870	0.00740134711626054	0.0571687466426485	0.0430565849119618	EN1/FZD1/HIF1A/HPRT1	4

BP	GO:0009411	response to UV	8/349	152/18870	0.00741937043263976	0.0571687466426485	0.0430565849119618	CCND1/DTL/EGFR/MMP1/MMP3/MMP9/PCNA/PTPRK	8

BP	GO:1903038	negative regulation of leukocyte cell-cell adhesion	8/349	152/18870	0.00741937043263976	0.0571687466426485	0.0430565849119618	CEBPB/GPNMB/HLA-DRB1/NRARP/PRNP/RUNX3/SOCS1/TWSG1	8

BP	GO:0021756	striatum development	3/349	22/18870	0.00744155461676882	0.0571687466426485	0.0430565849119618	HPRT1/INHBA/SLC7A11	3

BP	GO:0032069	regulation of nuclease activity	3/349	22/18870	0.00744155461676882	0.0571687466426485	0.0430565849119618	OAS2/OAS3/PCNA	3

BP	GO:0060339	negative regulation of type I interferon-mediated signaling pathway	3/349	22/18870	0.00744155461676882	0.0571687466426485	0.0430565849119618	ISG15/MMP12/OAS3	3

BP	GO:0072234	metanephric nephron tubule development	3/349	22/18870	0.00744155461676882	0.0571687466426485	0.0430565849119618	SOX9/STAT1/WNT7B	3

BP	GO:0098801	regulation of renal system process	3/349	22/18870	0.00744155461676882	0.0571687466426485	0.0430565849119618	F2R/F2RL1/STC1	3

BP	GO:0034605	cellular response to heat	5/349	66/18870	0.00747739367412164	0.0571687466426485	0.0430565849119618	ANO1/CXCL10/HSP90AA1/HSP90AB1/HSP90AB3P	5

BP	GO:0061180	mammary gland epithelium development	5/349	66/18870	0.00747739367412164	0.0571687466426485	0.0430565849119618	CCND1/CEBPB/HIF1A/WNT7B/ZNF703	5

BP	GO:0010001	glial cell differentiation	11/349	252/18870	0.00747962499520252	0.0571687466426485	0.0430565849119618	ANO1/APP/C1QA/CDK6/EGR2/ITGB4/PLEC/SOX4/SOX9/TREM2/VCAN	11

BP	GO:0033500	carbohydrate homeostasis	11/349	252/18870	0.00747962499520252	0.0571687466426485	0.0430565849119618	ANO1/GCLM/HIF1A/HLA-DRB1/ICAM1/IRS2/NUCKS1/SESN3/SLC16A1/SOX4/TREM2	11

BP	GO:0051346	negative regulation of hydrolase activity	10/349	218/18870	0.00756728201905202	0.0576220736805525	0.043398007721568	APOC1/CST1/IFI6/LAMP3/MAGEA3/MMP9/PAK2/SERPINE1/SERPINH1/SH3BP4	10

BP	GO:0072001	renal system development	13/349	324/18870	0.00758824488787715	0.0576220736805525	0.043398007721568	BASP1/COL4A1/EPCAM/GLI2/HOXB7/HOXD11/IRX2/MMP9/ODC1/SOX4/SOX9/STAT1/WNT7B	13

BP	GO:0039531	regulation of viral-induced cytoplasmic pattern recognition receptor signaling pathway	6/349	93/18870	0.00759615718082755	0.0576220736805525	0.043398007721568	DDX60/EIF2AK2/F2RL1/RSAD2/TNFAIP3/TREM2	6

BP	GO:0043367	CD4-positive, alpha-beta T cell differentiation	6/349	93/18870	0.00759615718082755	0.0576220736805525	0.043398007721568	HLA-DRA/HLA-DRB1/RSAD2/RUNX3/SOCS1/SOCS3	6

BP	GO:1902107	positive regulation of leukocyte differentiation	9/349	185/18870	0.00765879515653264	0.0578792249352974	0.0435916809343874	GLI2/HLA-DRA/HLA-DRB1/RUNX3/SOCS1/SOX12/SOX4/TREM2/TRIB1	9

BP	GO:1903708	positive regulation of hemopoiesis	9/349	185/18870	0.00765879515653264	0.0578792249352974	0.0435916809343874	GLI2/HLA-DRA/HLA-DRB1/RUNX3/SOCS1/SOX12/SOX4/TREM2/TRIB1	9

BP	GO:1903829	positive regulation of protein localization	17/349	476/18870	0.00771092723262016	0.0581640728333221	0.0438062138466613	ANO1/DTX3L/ECT2/EGFR/F2RL1/GNL3L/HIF1A/HLA-DRB1/HSP90AA1/HSP90AB1/IRS2/MSN/PRNP/SOX4/TFRC/TNFAIP6/TREM2	17

BP	GO:0009952	anterior/posterior pattern specification	10/349	219/18870	0.0078034156712529	0.0587516978015078	0.0442487830094459	BASP1/BHLHE41/EN1/HOXB7/HOXC10/HOXC13/HOXD10/HOXD13/NRARP/SEMA3C	10

BP	GO:0032872	regulation of stress-activated MAPK cascade	9/349	186/18870	0.00792144894617172	0.0594928102985336	0.0448069511525568	APP/EGFR/EIF2AK2/F2RL1/FOXM1/FZD7/MFHAS1/TREM2/WNT7B	9

BP	GO:0051057	positive regulation of small GTPase mediated signal transduction	5/349	67/18870	0.00796092967996763	0.0594928102985336	0.0448069511525568	COL3A1/F2R/F2RL1/ITGAV/PRAG1	5

BP	GO:0070613	regulation of protein processing	5/349	67/18870	0.00796092967996763	0.0594928102985336	0.0448069511525568	MAGEA3/MYH9/PLAU/PRNP/SERPINE1	5

BP	GO:0071300	cellular response to retinoic acid	5/349	67/18870	0.00796092967996763	0.0594928102985336	0.0448069511525568	COL1A1/FZD7/SOX9/TNC/WNT7B	5

BP	GO:0034614	cellular response to reactive oxygen species	8/349	154/18870	0.00800214266870968	0.0596900567954863	0.0449555071563768	ECT2/EGFR/MET/MMP3/MMP9/PCNA/PTPRK/TNFAIP3	8

BP	GO:0010712	regulation of collagen metabolic process	4/349	43/18870	0.00804386306704316	0.0597798531993539	0.0450231372290122	CREB3L1/F2R/INHBA/ITGA2	4

BP	GO:0014002	astrocyte development	4/349	43/18870	0.00804386306704316	0.0597798531993539	0.0450231372290122	APP/C1QA/CDK6/TREM2	4

BP	GO:0002758	innate immune response-activating signaling pathway	11/349	255/18870	0.00813887740045609	0.0601458928834195	0.0452988196545275	CD14/DDX60/EIF2AK2/F2RL1/FOSL1/MFHAS1/OAS3/PAK2/RSAD2/TNFAIP3/TREM2	11

BP	GO:0034599	cellular response to oxidative stress	11/349	255/18870	0.00813887740045609	0.0601458928834195	0.0452988196545275	ABCC1/ECT2/EGFR/HIF1A/MET/MMP3/MMP9/PCNA/PTPRK/SLC7A11/TNFAIP3	11

BP	GO:0051259	protein complex oligomerization	11/349	255/18870	0.00813887740045609	0.0601458928834195	0.0452988196545275	CLDN1/COL1A2/ECT2/HLA-DRB1/HPRT1/ISG15/KRT10/PRNP/PXDN/TK1/TP63	11

BP	GO:0042176	regulation of protein catabolic process	14/349	364/18870	0.00815284446731555	0.0601458928834195	0.0452988196545275	DTL/EGFR/GNA12/HSP90AA1/HSP90AB1/LAMP3/LAPTM4B/MSN/ODC1/SOX9/TMTC3/TNFAIP3/TREM2/TRIB1	14

BP	GO:0046632	alpha-beta T cell differentiation	7/349	124/18870	0.00830019392834711	0.060941693262349	0.0458981759217295	HLA-DRA/HLA-DRB1/PRR7/RSAD2/RUNX3/SOCS1/SOCS3	7

BP	GO:0044839	cell cycle G2/M phase transition	8/349	155/18870	0.00830610466758432	0.060941693262349	0.0458981759217295	APP/CCNA2/CCND1/CDK6/CLSPN/DTL/FOXM1/KIF14	8

BP	GO:0045807	positive regulation of endocytosis	8/349	155/18870	0.00830610466758432	0.060941693262349	0.0458981759217295	CD14/F2RL1/FCER1G/ITGA2/PPT1/SERPINE1/SFRP4/TREM2	8

BP	GO:1901659	glycosyl compound biosynthetic process	3/349	23/18870	0.00844212894889894	0.0618270825566635	0.0465650059918215	HPRT1/NT5E/TK1	3

BP	GO:0044000	movement in host	9/349	188/18870	0.00846729711745436	0.0618988616862181	0.046619066372153	CLDN1/CXCL8/EGFR/HLA-DRB1/ICAM1/ITGA2/ITGAV/TFRC/XPR1	9

BP	GO:0046718	viral entry into host cell	8/349	156/18870	0.00861864290253642	0.0628911116148853	0.0473663784231693	CLDN1/EGFR/HLA-DRB1/ICAM1/ITGA2/ITGAV/TFRC/XPR1	8

BP	GO:0001838	embryonic epithelial tube formation	7/349	125/18870	0.00865731482677249	0.0630590671288238	0.047492873952143	CTHRC1/FZD1/FZD2/FZD6/HIF1A/IRX2/SOX9	7

BP	GO:0097178	ruffle assembly	4/349	44/18870	0.00872191072665655	0.0634149032616834	0.0477608715831734	CSPG4/ICAM1/PFN2/SNX10	4

BP	GO:0070302	regulation of stress-activated protein kinase signaling cascade	9/349	189/18870	0.00875071143726593	0.0635096678726255	0.0478322434560463	APP/EGFR/EIF2AK2/F2RL1/FOXM1/FZD7/MFHAS1/TREM2/WNT7B	9

BP	GO:0021700	developmental maturation	13/349	330/18870	0.00877531931578151	0.0635737161941869	0.0478804813859945	AGRN/APP/BNC1/C1QA/CLSTN1/GAL/HIF1A/KIF14/NEFL/RAB32/SEZ6L2/SNX10/TRIP13	13

BP	GO:0010720	positive regulation of cell development	16/349	444/18870	0.0088452481439287	0.0639652774214449	0.0481753853364878	BNC1/EGR2/GLI2/HIF1A/HLA-DRA/HLA-DRB1/NEFL/PLXNA1/RUNX3/SLC7A5/SOCS1/SOX12/SOX4/TREM2/TRIB1/VCAN	16

CC	GO:0062023	collagen-containing extracellular matrix	38/357	429/19886	4.63992410105309e-16	1.76781108250123e-13	1.43105027537743e-13	AGRN/AMTN/ASPN/BGN/C1QA/C1QB/C1QC/COL10A1/COL11A1/COL1A1/COL1A2/COL3A1/COL4A1/COL4A2/COL5A1/COL5A2/CSPG4/CTHRC1/HSP90AA1/ICAM1/ITGB4/LAMB3/LAMC2/LGALS3BP/LOX/LOXL2/LUM/MMP9/MXRA5/POSTN/PXDN/S100A7/SERPINE1/SERPINH1/TGFBI/THBS2/TNC/VCAN	38

CC	GO:0098644	complex of collagen trimers	10/357	21/19886	9.07793103671206e-13	1.72934586249365e-10	1.39991252302981e-10	COL10A1/COL11A1/COL1A1/COL1A2/COL3A1/COL4A1/COL4A2/COL5A1/COL5A2/LUM	10

CC	GO:0005581	collagen trimer	15/357	86/19886	3.32283453244394e-11	4.2199998562038e-09	3.41610708072307e-09	C1QA/C1QB/C1QC/COL10A1/COL11A1/COL1A1/COL1A2/COL3A1/COL4A1/COL4A2/COL5A1/COL5A2/CTHRC1/LOX/LUM	15

CC	GO:0005583	fibrillar collagen trimer	7/357	12/19886	4.15553164689357e-10	3.1665151149329e-08	2.56330688955751e-08	COL11A1/COL1A1/COL1A2/COL3A1/COL5A1/COL5A2/LUM	7

CC	GO:0098643	banded collagen fibril	7/357	12/19886	4.15553164689357e-10	3.1665151149329e-08	2.56330688955751e-08	COL11A1/COL1A1/COL1A2/COL3A1/COL5A1/COL5A2/LUM	7

CC	GO:0019814	immunoglobulin complex	18/357	165/19886	1.09302574934349e-09	6.94071350833117e-08	5.61853586943234e-08	IGHG2/IGHG3/IGHG4/IGHV1-18/IGHV3-15/IGHV3-21/IGHV3-23/IGHV3-48/IGHV4-34/IGHV4-39/IGHV5-51/IGKV1-5/IGKV3-20/IGKV4-1/IGLV1-40/IGLV3-19/IGLV3-21/IGLV3-25	18

CC	GO:0030055	cell-substrate junction	28/357	431/19886	5.08523141091494e-09	2.76781881079799e-07	2.24056060661365e-07	ALCAM/CSPG4/EGFR/EPPK1/FZD1/FZD2/GJA1/GNA12/GNA13/HSPA5/ICAM1/ITGA2/ITGA6/ITGAV/ITGB4/MAPRE1/MARCKS/MISP/MSN/MYH9/PLAU/PLEC/PRAG1/S100A7/TNC/TNS4/YWHAG/YWHAQ	28

CC	GO:0005604	basement membrane	13/357	90/19886	7.65568659514237e-09	3.64602074093655e-07	2.9514686478641e-07	AGRN/AMTN/COL4A1/COL4A2/COL5A1/ITGB4/LAMB3/LAMC2/LOXL2/PXDN/TGFBI/THBS2/TNC	13

CC	GO:0005925	focal adhesion	27/357	421/19886	1.26591494697283e-08	5.35903994218499e-07	4.33816467208234e-07	ALCAM/CSPG4/EGFR/FZD1/FZD2/GJA1/GNA12/GNA13/HSPA5/ICAM1/ITGA2/ITGA6/ITGAV/ITGB4/MAPRE1/MARCKS/MISP/MSN/MYH9/PLAU/PLEC/PRAG1/S100A7/TNC/TNS4/YWHAG/YWHAQ	27

CC	GO:0005788	endoplasmic reticulum lumen	21/357	313/19886	2.59415256174466e-07	9.88372126024717e-06	8.00091263780196e-06	AMTN/APP/COL10A1/COL11A1/COL1A1/COL1A2/COL3A1/COL4A1/COL4A2/COL5A1/COL5A2/FOXRED2/HSPA5/IGFBP3/P4HA1/PDIA4/SERPINH1/SPP1/TNC/VCAN/WNT7B	21

CC	GO:0072562	blood microparticle	14/357	144/19886	3.35570900435992e-07	1.1622955733283e-05	9.40883003136322e-06	C1QB/C1QC/IGHG2/IGHG3/IGHG4/IGHV3-23/IGKV1-5/IGKV3-20/IGKV4-1/IGLV3-21/IGLV3-25/LGALS3BP/MSN/TFRC	14

CC	GO:0045177	apical part of cell	22/357	469/19886	4.39714843705887e-05	0.0013221283681723	0.00107026830190491	ABCC1/ANO1/APP/BST2/CLDN1/CLIC4/CSPG4/DSG2/EPCAM/FZD6/GJA1/HSP90AA1/ITPR3/MSN/MTDH/SIPA1L3/SLC16A1/SLC39A6/SLC7A11/SLC7A5/SNX10/STC1	22

CC	GO:0042571	immunoglobulin complex, circulating	4/357	12/19886	4.5111991564934e-05	0.0013221283681723	0.00107026830190491	IGHG2/IGHG3/IGHG4/IGKV3-20	4

CC	GO:0001533	cornified envelope	7/357	60/19886	9.66241298346201e-05	0.00262955667621359	0.00212863684522885	DSC3/DSG2/KRT10/KRT14/KRT17/KRT75/SPRR2G	7

CC	GO:0005796	Golgi lumen	9/357	106/19886	0.000124526377120597	0.00316296997886316	0.0025604370874621	AGRN/APP/BGN/CSPG4/GOLIM4/LUM/MMP11/VCAN/WNT7B	9

CC	GO:0042470	melanosome	9/357	112/19886	0.000189670393143127	0.00425084822279597	0.0034410789591911	GNA13/GPNMB/HSP90AA1/HSP90AB1/HSPA5/PDIA4/RAB32/SLC1A4/TFRC	9

CC	GO:0048770	pigment granule	9/357	112/19886	0.000189670393143127	0.00425084822279597	0.0034410789591911	GNA13/GPNMB/HSP90AA1/HSP90AB1/HSPA5/PDIA4/RAB32/SLC1A4/TFRC	9

CC	GO:0045121	membrane raft	15/357	286/19886	0.000221160303913371	0.00456276601558517	0.00369357768356529	APP/BST2/CD14/EFHD2/EFNB1/EGFR/F2R/GJA1/ICAM1/PPT1/PRNP/SERPINH1/SLC39A6/TREM2/TUBB	15

CC	GO:0098857	membrane microdomain	15/357	287/19886	0.000229630046299207	0.00456276601558517	0.00369357768356529	APP/BST2/CD14/EFHD2/EFNB1/EGFR/F2R/GJA1/ICAM1/PPT1/PRNP/SERPINH1/SLC39A6/TREM2/TUBB	15

CC	GO:0009897	external side of plasma membrane	18/357	387/19886	0.000239515276408671	0.00456276601558517	0.00369357768356529	ACKR3/ALCAM/CD14/CXCL10/CXCL9/FCER1G/FCGR3A/HLA-DRB1/ICAM1/IL27RA/ITGA2/ITGA6/ITGAV/NT5E/PLAU/PRNP/SLC7A5/TFRC	18

CC	GO:0031528	microvillus membrane	5/357	33/19886	0.000284571000291245	0.00516293100528401	0.00417941368848795	CA9/ITGAV/MSN/SLC7A11/SLC7A5	5

CC	GO:0005775	vacuolar lumen	11/357	176/19886	0.000350838218301036	0.0060758800533043	0.00491844966326332	AGRN/BGN/CSPG4/FTL/HSP90AA1/LUM/PLBD2/PPT1/S100A7/TUBB/VCAN	11

CC	GO:0045178	basal part of cell	15/357	301/19886	0.000381161331210283	0.0061105196236326	0.00494649053660548	ABCC1/CA9/CLDN1/EGFR/EPCAM/EPPK1/HSP90AA1/ITGA2/ITGB4/KRT14/MET/MSN/SLC16A1/SLC7A5/TFRC	15

CC	GO:0043202	lysosomal lumen	8/357	98/19886	0.000385539356041986	0.0061105196236326	0.00494649053660548	AGRN/BGN/CSPG4/HSP90AA1/LUM/PLBD2/PPT1/VCAN	8

CC	GO:0000307	cyclin-dependent protein kinase holoenzyme complex	6/357	54/19886	0.000400952731209489	0.0061105196236326	0.00494649053660548	CCNA1/CCNA2/CCND1/CDK6/CKS2/PCNA	6

CC	GO:0098636	protein complex involved in cell adhesion	6/357	57/19886	0.000538529470612565	0.00789152801166874	0.0063882240845944	ITGA2/ITGA6/ITGAV/ITGB4/PLAU/TNC	6

CC	GO:0016324	apical plasma membrane	17/357	403/19886	0.00105538624384433	0.0148926725520256	0.0120556791207169	ABCC1/ANO1/BST2/CLDN1/CSPG4/DSG2/EPCAM/FZD6/GJA1/HSP90AA1/MSN/MTDH/SIPA1L3/SLC16A1/SLC39A6/SLC7A5/STC1	17

CC	GO:0001772	immunological synapse	5/357	45/19886	0.00122860713843498	0.0167178328479903	0.0135331538180996	ALCAM/HLA-DRA/HLA-DRB1/ICAM1/MYH9	5

CC	GO:0016328	lateral plasma membrane	6/357	67/19886	0.00127483978238138	0.0167487571409416	0.0135581871592648	ABCC1/CLDN1/DSG2/EPCAM/GNA12/SLC16A1	6

CC	GO:0071735	IgG immunoglobulin complex	3/357	13/19886	0.00143543446277457	0.0182300176772371	0.0147572735997526	IGHG2/IGHG3/IGHG4	3

CC	GO:0005902	microvillus	7/357	97/19886	0.00182681363433403	0.0220417941600038	0.0178429221961075	CA9/CLIC4/FSCN1/ITGAV/MSN/SLC7A11/SLC7A5	7

CC	GO:0009925	basal plasma membrane	13/357	282/19886	0.00185127929952788	0.0220417941600038	0.0178429221961075	ABCC1/CA9/CLDN1/EGFR/EPCAM/EPPK1/HSP90AA1/ITGB4/MET/MSN/SLC16A1/SLC7A5/TFRC	13

CC	GO:0008305	integrin complex	4/357	31/19886	0.00219096224190292	0.0252956549746974	0.020476935785568	ITGA2/ITGA6/ITGAV/ITGB4	4

CC	GO:0070160	tight junction	8/357	131/19886	0.00254790364090924	0.0285515084466594	0.0231125624392077	CCND1/CLDN1/ECT2/EPCAM/EPPK1/GJA1/MTDH/SIPA1L3	8

CC	GO:0005903	brush border	7/357	108/19886	0.00335584996544778	0.0365308239095886	0.0295718508233443	GNA12/GNA13/HSP90AA1/ITPR3/MYH9/PLEC/SLC7A11	7

CC	GO:0005882	intermediate filament	10/357	214/19886	0.00546739696195264	0.0571288704753908	0.0462460534584597	EPPK1/FAM83H/HLA-DRB1/KRT10/KRT14/KRT17/KRT75/NEFL/PLEC/SLC1A4	10

CC	GO:0016323	basolateral plasma membrane	11/357	249/19886	0.0055479480514159	0.0571288704753908	0.0462460534584597	ABCC1/CA9/CLDN1/EGFR/EPCAM/EPPK1/HSP90AA1/MSN/SLC16A1/SLC7A5/TFRC	11

CC	GO:0045111	intermediate filament cytoskeleton	11/357	250/19886	0.00571302901203212	0.0572806329890589	0.0463689058317288	DDX60/EPPK1/FAM83H/HLA-DRB1/KRT10/KRT14/KRT17/KRT75/NEFL/PLEC/SLC1A4	11

MF	GO:0005201	extracellular matrix structural constituent	23/332	166/18496	2.89363555800572e-14	1.65805317473728e-11	1.37676133917746e-11	AGRN/ASPN/BGN/COL10A1/COL11A1/COL1A1/COL1A2/COL3A1/COL4A1/COL4A2/COL5A1/COL5A2/CTHRC1/LAMB3/LAMC2/LUM/MXRA5/POSTN/PXDN/TGFBI/THBS2/TNC/VCAN	23

MF	GO:0003823	antigen binding	22/332	178/18496	1.14467100367055e-12	3.27948242551612e-10	2.72311207188993e-10	HLA-DRA/HLA-DRB1/IGHG2/IGHG3/IGHG4/IGHV1-18/IGHV3-15/IGHV3-21/IGHV3-23/IGHV3-48/IGHV4-34/IGHV4-39/IGHV5-51/IGKV1-5/IGKV3-20/IGKV4-1/IGLV1-40/IGLV3-19/IGLV3-21/IGLV3-25/SLC7A5/TAPBP	22

MF	GO:0050840	extracellular matrix binding	10/332	55/18496	4.33514808986222e-08	8.28013285163684e-06	6.87539276006219e-06	AGRN/BGN/COL11A1/ITGA2/ITGA6/ITGAV/OLFML2B/PXDN/SPP1/TGFBI	10

MF	GO:0001664	G protein-coupled receptor binding	21/332	291/18496	7.40012911321988e-08	9.98385518483763e-06	8.29007539918547e-06	CCL18/CCL5/CTHRC1/CXCL10/CXCL13/CXCL14/CXCL8/CXCL9/FZD1/FZD7/GAL/GNA12/GNA13/ITGB4/NMB/NPW/NTS/PRNP/STAT1/UCHL1/WNT7B	21

MF	GO:0030020	extracellular matrix structural constituent conferring tensile strength	9/332	45/18496	8.71191551905552e-08	9.98385518483763e-06	8.29007539918547e-06	COL10A1/COL11A1/COL1A1/COL1A2/COL3A1/COL4A1/COL4A2/COL5A1/COL5A2	9

MF	GO:0019838	growth factor binding	14/332	135/18496	1.48000379275066e-07	1.41340362207688e-05	1.17361704267246e-05	CD109/COL1A1/COL1A2/COL3A1/COL4A1/COL5A1/CXCL13/EGFR/IGFBP3/ITGA6/ITGAV/ITGB4/PXDN/TWSG1	14

MF	GO:0005539	glycosaminoglycan binding	18/332	240/18496	3.646118471846e-07	2.98460840623966e-05	2.47826398387127e-05	AGRN/APP/BGN/COL11A1/COL5A1/CXCL10/CXCL13/CXCL8/GPNMB/LAMC2/NELL2/POSTN/PRNP/THBS2/TNFAIP6/TREM2/TWSG1/VCAN	18

MF	GO:0048407	platelet-derived growth factor binding	5/332	11/18496	7.64518677747981e-07	5.47586502936992e-05	4.54687424134326e-05	COL1A1/COL1A2/COL3A1/COL4A1/COL5A1	5

MF	GO:0044389	ubiquitin-like protein ligase binding	20/332	327/18496	2.06616061588125e-06	0.000131545559211106	0.000109228607997465	CEBPB/DTX3L/EGFR/EGR2/FZD6/HIF1A/HLTF/HSP90AA1/HSP90AB1/HSPA5/ISG15/LAPTM4B/MFHAS1/PRR7/STAT1/TRIB1/TUBB/UBE2C/UBE2T/UCHL1	20

MF	GO:0003725	double-stranded RNA binding	9/332	73/18496	6.14031208574029e-06	0.000351839882512918	0.000292149585553117	DDX21/DDX60/EIF2AK2/HSP90AB1/MSN/MTDH/OAS2/OAS3/TFRC	9

MF	GO:1901681	sulfur compound binding	17/332	275/18496	1.05445114263767e-05	0.000549273186119441	0.000456087958346628	AGRN/APP/COL11A1/COL5A1/CXCL10/CXCL13/CXCL8/FST/FTL/GPNMB/ITGA2/LAMC2/NELL2/POSTN/PRNP/THBS2/TWSG1	17

MF	GO:0048019	receptor antagonist activity	6/332	31/18496	1.6115842201014e-05	0.000769531465098418	0.000638979006566519	CCL5/FST/MTRNR2L12/MTRNR2L2/MTRNR2L8/PXDN	6

MF	GO:0017147	Wnt-protein binding	6/332	32/18496	1.95372618904969e-05	0.000861142389481132	0.000715047965546931	CTHRC1/FZD1/FZD2/FZD6/FZD7/SFRP4	6

MF	GO:0005178	integrin binding	12/332	154/18496	2.25152968164553e-05	0.000921518933987778	0.000765181515867504	COL3A1/COL5A1/GPNMB/ICAM1/ISG15/ITGA2/ITGA6/ITGAV/ITGB4/MYH9/SPP1/TGFBI	12

MF	GO:0008009	chemokine activity	7/332	49/18496	2.53369829307464e-05	0.000967872747954511	0.000803671318224376	CCL18/CCL5/CXCL10/CXCL13/CXCL14/CXCL8/CXCL9	7

MF	GO:0005518	collagen binding	8/332	68/18496	2.86967144011492e-05	0.00102770108449116	0.000853349665086806	ITGA2/LOX/LUM/MMP12/MMP13/MMP9/SERPINH1/TGFBI	8

MF	GO:0043394	proteoglycan binding	6/332	36/18496	3.95314021130123e-05	0.001332440788868	0.001106389706197	AGRN/COL5A1/FST/GPNMB/ITGA2/TNC	6

MF	GO:0042379	chemokine receptor binding	8/332	74/18496	5.33021350415909e-05	0.00169678463215731	0.00140892193209352	CCL18/CCL5/CXCL10/CXCL13/CXCL14/CXCL8/CXCL9/STAT1	8

MF	GO:0031994	insulin-like growth factor I binding	4/332	13/18496	6.41322945399601e-05	0.00193409498796827	0.00160597214028044	IGFBP3/ITGA6/ITGAV/ITGB4	4

MF	GO:0008201	heparin binding	12/332	174/18496	7.45998467415877e-05	0.00213728560914649	0.00177469109090514	APP/COL11A1/COL5A1/CXCL10/CXCL13/CXCL8/GPNMB/LAMC2/NELL2/POSTN/THBS2/TWSG1	12

MF	GO:0030547	signaling receptor inhibitor activity	6/332	42/18496	9.72534463715222e-05	0.00265362975099439	0.00220343647919439	CCL5/FST/MTRNR2L12/MTRNR2L2/MTRNR2L8/PXDN	6

MF	GO:0042813	Wnt receptor activity	4/332	16/18496	0.000156432731887343	0.00407436160779306	0.00338313850780282	FZD1/FZD2/FZD6/FZD7	4

MF	GO:0004222	metalloendopeptidase activity	9/332	110/18496	0.000164420754798184	0.00409622141301562	0.00340128975600819	ADAM12/MMP1/MMP10/MMP11/MMP12/MMP13/MMP3/MMP7/MMP9	9

MF	GO:0019207	kinase regulator activity	14/332	256/18496	0.000230157926707489	0.00538192774756733	0.00446887359584905	CCL5/CCNA1/CCNA2/CCND1/CKS2/CXCL10/EGFR/HSP90AB1/PAK2/SOCS1/SOCS3/TREM2/TRIB1/YWHAG	14

MF	GO:0019887	protein kinase regulator activity	13/332	226/18496	0.000234813601551803	0.00538192774756733	0.00446887359584905	CCL5/CCNA1/CCNA2/CCND1/CKS2/CXCL10/EGFR/HSP90AB1/PAK2/SOCS1/SOCS3/TRIB1/YWHAG	13

MF	GO:0002020	protease binding	10/332	142/18496	0.000248634854367893	0.00542554707359092	0.00450509282127877	COL1A1/COL1A2/COL3A1/F2RL1/ITGAV/MAGEA3/PANX1/PRNP/SERPINE1/TNFAIP3	10

MF	GO:0045236	CXCR chemokine receptor binding	4/332	18/18496	0.000255654050588054	0.00542554707359092	0.00450509282127877	CXCL10/CXCL13/CXCL8/CXCL9	4

MF	GO:0005520	insulin-like growth factor binding	4/332	19/18496	0.000319269891485587	0.00653363027933004	0.00542518762975508	IGFBP3/ITGA6/ITGAV/ITGB4	4

MF	GO:0001216	DNA-binding transcription activator activity	20/332	475/18496	0.000395739833956998	0.00781927327094345	0.00649271887290611	BNC1/CEBPB/CREB3L1/DLX5/EGR2/ELF4/FOSB/FOSL1/HIF1A/HOXB7/HOXC10/HOXC13/HOXD10/HOXD13/JUND/MYBL2/SOX12/SOX4/SOX9/TP63	20

MF	GO:0031625	ubiquitin protein ligase binding	15/332	308/18496	0.000479510107598454	0.00880497351563293	0.00731119321955742	EGFR/EGR2/FZD6/HIF1A/HLTF/HSP90AA1/HSP90AB1/HSPA5/ISG15/LAPTM4B/MFHAS1/TRIB1/TUBB/UBE2T/UCHL1	15

MF	GO:0042826	histone deacetylase binding	9/332	127/18496	0.000481283199327683	0.00880497351563293	0.00731119321955742	BHLHE41/CCND1/CEBPB/HIF1A/HSP90AA1/HSP90AB1/KPNA2/MAGEA3/MAGEA6	9

MF	GO:0023023	MHC protein complex binding	5/332	37/18496	0.000491726269633951	0.00880497351563293	0.00731119321955742	HLA-DRA/HLA-DRB1/HSP90AA1/HSP90AB1/TAPBP	5

MF	GO:0001618	virus receptor activity	7/332	79/18496	0.000540217925572142	0.00938014761675265	0.00778878795402259	CLDN1/EGFR/ICAM1/ITGA2/ITGAV/TFRC/XPR1	7

MF	GO:0030021	extracellular matrix structural constituent conferring compression resistance	4/332	22/18496	0.000577483089782419	0.00954557567404864	0.0079261508306604	ASPN/BGN/LUM/VCAN	4

MF	GO:0140272	exogenous protein binding	7/332	80/18496	0.000583063086547474	0.00954557567404864	0.0079261508306604	CLDN1/EGFR/ICAM1/ITGA2/ITGAV/TFRC/XPR1	7

MF	GO:0140662	ATP-dependent protein folding chaperone	5/332	40/18496	0.000710373867140096	0.0113067840519799	0.00938856689904454	HSP90AA1/HSP90AB1/HSP90AB3P/HSPA13/HSPA5	5

MF	GO:0019215	intermediate filament binding	3/332	11/18496	0.000849714611253763	0.0129326988169201	0.0107386421700154	EPPK1/FAM83H/KRT14	3

MF	GO:0017171	serine hydrolase activity	11/332	196/18496	0.000857665890127335	0.0129326988169201	0.0107386421700154	HTRA3/MMP1/MMP10/MMP11/MMP12/MMP13/MMP3/MMP7/MMP9/NCEH1/PLAU	11

MF	GO:0001228	DNA-binding transcription activator activity, RNA polymerase II-specific	19/332	471/18496	0.000917510352354887	0.0134803444076756	0.0111933786576089	CEBPB/CREB3L1/DLX5/EGR2/ELF4/FOSB/FOSL1/HIF1A/HOXB7/HOXC10/HOXC13/HOXD10/HOXD13/JUND/MYBL2/SOX12/SOX4/SOX9/TP63	19

MF	GO:0004252	serine-type endopeptidase activity	10/332	174/18496	0.00122023793355854	0.0174799083982261	0.0145144091044332	HTRA3/MMP1/MMP10/MMP11/MMP12/MMP13/MMP3/MMP7/MMP9/PLAU	10

MF	GO:0023026	MHC class II protein complex binding	4/332	27/18496	0.00129100255528954	0.0180425479068513	0.0149815957635654	HLA-DRA/HLA-DRB1/HSP90AA1/HSP90AB1	4

MF	GO:0043236	laminin binding	4/332	28/18496	0.00148508080336542	0.0202607452459139	0.0168234717574228	AGRN/ITGA2/ITGA6/PXDN	4

MF	GO:0008237	metallopeptidase activity	10/332	185/18496	0.00192692305579495	0.0256773700225699	0.0213211559662012	ADAM12/CPXM1/MMP1/MMP10/MMP11/MMP12/MMP13/MMP3/MMP7/MMP9	10

MF	GO:0005080	protein kinase C binding	5/332	51/18496	0.00215969908237212	0.0281251721408915	0.0233536838572296	ITGAV/MARCKS/NELL2/TOP2A/YWHAG	5

MF	GO:0015562	efflux transmembrane transporter activity	3/332	15/18496	0.0022215695100239	0.0282879850943043	0.0234888752872703	ABCC1/GJA1/XPR1	3

MF	GO:0042277	peptide binding	14/332	325/18496	0.00233477528368508	0.0290831790772077	0.0241491631172919	C1QA/CLSTN1/HLA-DRA/HLA-DRB1/HSP90AB1/INHBA/ITGA2/KDELR3/KPNA2/PRNP/SLC7A5/TAPBP/TMEM158/TREM2	14

MF	GO:0008236	serine-type peptidase activity	10/332	192/18496	0.00252731984378064	0.0308117929890703	0.0255845144320011	HTRA3/MMP1/MMP10/MMP11/MMP12/MMP13/MMP3/MMP7/MMP9/PLAU	10

MF	GO:0034987	immunoglobulin receptor binding	3/332	16/18496	0.00269813953574772	0.0322090407079884	0.0267447164508327	IGHG2/IGHG3/IGHG4	3

MF	GO:0033218	amide binding	16/332	405/18496	0.00280470234099545	0.0327978457426611	0.0272336296053693	C1QA/CLSTN1/GRIN2D/HLA-DRA/HLA-DRB1/HSP90AB1/INHBA/ITGA2/KDELR3/KPNA2/LAPTM4B/PRNP/SLC7A5/TAPBP/TMEM158/TREM2	16

MF	GO:0043395	heparan sulfate proteoglycan binding	3/332	18/18496	0.00382858264665075	0.0438755571306176	0.0364319864481293	AGRN/FST/ITGA2	3

MF	GO:0005200	structural constituent of cytoskeleton	7/332	112/18496	0.00409146587892714	0.0459688225220637	0.0381701254339539	AGRN/HLA-DRB1/KRT14/MSN/NEFL/PLEC/TUBB	7

MF	GO:0005109	frizzled binding	4/332	37/18496	0.00422106968181398	0.0465129409169118	0.038621933121051	CTHRC1/FZD1/FZD7/WNT7B	4
