# Supplementary material for: Gene Expression Profiling and Clinical Validation Reveal CEBPB for Early Diagnosis of Esophageal Squamous Cell Carcinoma
Source: Cancer Rep (Hoboken). 2026 Nov 8;9(11):e70753. doi: 10.1002/cnr2.70753 (PMC13745642; doi:10.1002/cnr2.70753)
Supplement: Supplementary file 14 [file CNR2-9-e70753-s011.doc]

Doc. S8. KEGG pathway enrichment analysis of the seven key genes.

category	subcategory	ID	Description	GeneRatio	BgRatio	pvalue	p.adjust	qvalue	geneID	Count

Organismal Systems	Immune system	hsa04657	IL-17 signaling pathway	2/4	95/8846	0.000675226971160052	0.0197824266615607	0.0101804300948382	CEBPB/CXCL8	2

Environmental Information Processing	Signal transduction	hsa04668	TNF signaling pathway	2/4	119/8846	0.00105790064874804	0.0197824266615607	0.0101804300948382	CEBPB/SOCS3	2

Genetic Information Processing	Folding, sorting and degradation	hsa04120	Ubiquitin mediated proteolysis	2/4	142/8846	0.00150315765866313	0.0197824266615607	0.0101804300948382	UBE2C/SOCS3	2

Human Diseases	Endocrine and metabolic disease	hsa04932	Non-alcoholic fatty liver disease	2/4	157/8846	0.00183454991243339	0.0197824266615607	0.0101804300948382	CXCL8/SOCS3	2

Human Diseases	Infectious disease: viral	hsa05164	Influenza A	2/4	172/8846	0.00219804740684007	0.0197824266615607	0.0101804300948382	CXCL8/SOCS3	2

Human Diseases	Cancer: overview	hsa05202	Transcriptional misregulation in cancer	2/4	193/8846	0.00276044739412384	0.0207033554559288	0.010654358363285	CEBPB/CXCL8	2

Human Diseases	Cancer: specific types	hsa05219	Bladder cancer	1/4	41/8846	0.0184140599266044	0.0946806758182044	0.0487245583158011	CXCL8	1

Human Diseases	Endocrine and metabolic disease	hsa04930	Type II diabetes mellitus	1/4	47/8846	0.0210873137447253	0.0946806758182044	0.0487245583158011	SOCS3	1

Human Diseases	Infectious disease: parasitic	hsa05144	Malaria	1/4	50/8846	0.0224218911950561	0.0946806758182044	0.0487245583158011	CXCL8	1

Human Diseases	Infectious disease: bacterial	hsa05134	Legionellosis	1/4	56/8846	0.0250869518355873	0.0946806758182044	0.0487245583158011	CXCL8	1

Organismal Systems	Endocrine system	hsa04920	Adipocytokine signaling pathway	1/4	70/8846	0.0312842341646622	0.0946806758182044	0.0487245583158011	SOCS3	1

Organismal Systems	Endocrine system	hsa04917	Prolactin signaling pathway	1/4	71/8846	0.0317257636841859	0.0946806758182044	0.0487245583158011	SOCS3	1

Human Diseases	Infectious disease: bacterial	hsa05120	Epithelial cell signaling in Helicobacter pylori infection	1/4	71/8846	0.0317257636841859	0.0946806758182044	0.0487245583158011	CXCL8	1

Organismal Systems	Immune system	hsa04622	RIG-I-like receptor signaling pathway	1/4	72/8846	0.0321671422534465	0.0946806758182044	0.0487245583158011	CXCL8	1

Human Diseases	Infectious disease: bacterial	hsa05133	Pertussis	1/4	78/8846	0.0348122456400902	0.0946806758182044	0.0487245583158011	CXCL8	1

Human Diseases	Immune disease	hsa05323	Rheumatoid arthritis	1/4	94/8846	0.0418393499618308	0.0946806758182044	0.0487245583158011	CXCL8	1

Environmental Information Processing	Signaling molecules and interaction	hsa04061	Viral protein interaction with cytokine and cytokine receptor	1/4	100/8846	0.0444645959771212	0.0946806758182044	0.0487245583158011	CXCL8	1

Human Diseases	Endocrine and metabolic disease	hsa04933	AGE-RAGE signaling pathway in diabetic complications	1/4	101/8846	0.0449016119405435	0.0946806758182044	0.0487245583158011	CXCL8	1

Human Diseases	Infectious disease: parasitic	hsa05142	Chagas disease	1/4	103/8846	0.0457751941425402	0.0946806758182044	0.0487245583158011	CXCL8	1

Human Diseases	Infectious disease: parasitic	hsa05146	Amoebiasis	1/4	103/8846	0.0457751941425402	0.0946806758182044	0.0487245583158011	CXCL8	1

Environmental Information Processing	Signal transduction	hsa04064	NF-kappa B signaling pathway	1/4	105/8846	0.0466481769399919	0.0946806758182044	0.0487245583158011	CXCL8	1

Organismal Systems	Immune system	hsa04620	Toll-like receptor signaling pathway	1/4	109/8846	0.0483923454181934	0.0946806758182044	0.0487245583158011	CXCL8	1

Human Diseases	Endocrine and metabolic disease	hsa04931	Insulin resistance	1/4	109/8846	0.0483923454181934	0.0946806758182044	0.0487245583158011	SOCS3	1

Organismal Systems	Endocrine system	hsa04935	Growth hormone synthesis, secretion and action	1/4	122/8846	0.0540443684979668	0.0977318996176128	0.0502947787505844	SOCS3	1

Organismal Systems	Endocrine system	hsa04910	Insulin signaling pathway	1/4	138/8846	0.0609660980172499	0.0977318996176128	0.0502947787505844	SOCS3	1

Human Diseases	Infectious disease: bacterial	hsa05135	Yersinia infection	1/4	138/8846	0.0609660980172499	0.0977318996176128	0.0502947787505844	CXCL8	1

Organismal Systems	Development and regeneration	hsa04380	Osteoclast differentiation	1/4	142/8846	0.0626905787882219	0.0977318996176128	0.0502947787505844	SOCS3	1

Human Diseases	Endocrine and metabolic disease	hsa04936	Alcoholic liver disease	1/4	144/8846	0.0635519279351391	0.0977318996176128	0.0502947787505844	CXCL8	1

Environmental Information Processing	Signal transduction	hsa04072	Phospholipase D signaling pathway	1/4	149/8846	0.0657027035464303	0.0977318996176128	0.0502947787505844	CXCL8	1

NA	NA	hsa04148	Efferocytosis	1/4	157/8846	0.069136235832016	0.0977318996176128	0.0502947787505844	CEBPB	1

Cellular Processes	Cell growth and death	hsa04218	Cellular senescence	1/4	157/8846	0.069136235832016	0.0977318996176128	0.0502947787505844	CXCL8	1

Human Diseases	Infectious disease: viral	hsa05160	Hepatitis C	1/4	159/8846	0.0699931381671424	0.0977318996176128	0.0502947787505844	SOCS3	1

Human Diseases	Infectious disease: viral	hsa05161	Hepatitis B	1/4	163/8846	0.0717051678619701	0.0977318996176128	0.0502947787505844	CXCL8	1

Environmental Information Processing	Signal transduction	hsa04630	JAK-STAT signaling pathway	1/4	168/8846	0.0738418797110852	0.0977318996176128	0.0502947787505844	SOCS3	1

Human Diseases	Infectious disease: bacterial	hsa05152	Tuberculosis	1/4	180/8846	0.0789549378931206	0.101513491576869	0.0522408611623655	CEBPB	1

Organismal Systems	Immune system	hsa04621	NOD-like receptor signaling pathway	1/4	189/8846	0.0827758157583963	0.101533477530509	0.0522511463314899	CXCL8	1

Organismal Systems	Immune system	hsa04062	Chemokine signaling pathway	1/4	193/8846	0.0844701623815719	0.101533477530509	0.0522511463314899	CXCL8	1

Human Diseases	Infectious disease: viral	hsa05167	Kaposi sarcoma-associated herpesvirus infection	1/4	196/8846	0.085739381025763	0.101533477530509	0.0522511463314899	CXCL8	1

Human Diseases	Infectious disease: bacterial	hsa05130	Pathogenic Escherichia coli infection	1/4	203/8846	0.0886957589923587	0.102341260375799	0.0526668474448554	CXCL8	1

Human Diseases	Cardiovascular disease	hsa05417	Lipid and atherosclerosis	1/4	216/8846	0.0941671465307884	0.105938039847137	0.0545178216757196	CXCL8	1

Human Diseases	Infectious disease: viral	hsa05163	Human cytomegalovirus infection	1/4	226/8846	0.0983591120520155	0.107955122983919	0.0555558527636545	CXCL8	1

Human Diseases	Infectious disease: viral	hsa05171	Coronavirus disease - COVID-19	1/4	238/8846	0.103370246031854	0.110753835034129	0.0569961256315988	CXCL8	1

Human Diseases	Infectious disease: bacterial	hsa05131	Shigellosis	1/4	249/8846	0.107945408896814	0.111247539381783	0.0572501957052452	CXCL8	1

Human Diseases	Infectious disease: bacterial	hsa05132	Salmonella infection	1/4	251/8846	0.108775371839966	0.111247539381783	0.0572501957052452	CXCL8	1

Environmental Information Processing	Signaling molecules and interaction	hsa04060	Cytokine-cytokine receptor interaction	1/4	298/8846	0.128113322240875	0.128113322240875	0.0659296629075848	CXCL8	1
