# Supplementary material for: Gene Expression Profiling and Clinical Validation Reveal CEBPB for Early Diagnosis of Esophageal Squamous Cell Carcinoma
Source: Cancer Rep (Hoboken). 2026 Nov 8;9(11):e70753. doi: 10.1002/cnr2.70753 (PMC13745642; doi:10.1002/cnr2.70753)
Supplement: Supplementary file 15 [file CNR2-9-e70753-s015.doc]

Doc. S7. GO enrichment analysis of the seven key genes.

ONTOLOGY	ID	Description	GeneRatio	BgRatio	pvalue	p.adjust	qvalue	geneID	Count

BP	GO:0071624	positive regulation of granulocyte chemotaxis	2/7	28/18870	4.43838363279034e-05	0.0155343427147662	0.00672765519075589	S100A7/CXCL8	2

BP	GO:0071622	regulation of granulocyte chemotaxis	2/7	53/18870	0.000161087809004186	0.0194253143310588	0.00841276771029313	S100A7/CXCL8	2

BP	GO:0032496	response to lipopolysaccharide	3/7	348/18870	0.000205991392127841	0.0194253143310588	0.00841276771029313	S100A7/CEBPB/CXCL8	3

BP	GO:0002237	response to molecule of bacterial origin	3/7	369/18870	0.0002448712137382	0.0194253143310588	0.00841276771029313	S100A7/CEBPB/CXCL8	3

BP	GO:0008544	epidermis development	3/7	385/18870	0.000277504490443697	0.0194253143310588	0.00841276771029313	S100A7/SPRR2G/KRTDAP	3

BP	GO:0061844	antimicrobial humoral immune response mediated by antimicrobial peptide	2/7	83/18870	0.000395704324173373	0.0195844700684171	0.00848169530782577	S100A7/CXCL8	2

BP	GO:0001892	embryonic placenta development	2/7	88/18870	0.000444730133359416	0.0195844700684171	0.00848169530782577	CEBPB/SOCS3	2

BP	GO:0048568	embryonic organ development	3/7	453/18870	0.000447645030135249	0.0195844700684171	0.00848169530782577	CEBPB/CXCL8/SOCS3	3

BP	GO:0002690	positive regulation of leukocyte chemotaxis	2/7	96/18870	0.000529023116021599	0.0205731211786177	0.00890986300667957	S100A7/CXCL8	2

BP	GO:0002688	regulation of leukocyte chemotaxis	2/7	126/18870	0.000908770090056411	0.0295085260214835	0.0127796323220861	S100A7/CXCL8	2

BP	GO:0071621	granulocyte chemotaxis	2/7	130/18870	0.000966938662529878	0.0295085260214835	0.0127796323220861	S100A7/CXCL8	2

BP	GO:0019730	antimicrobial humoral response	2/7	133/18870	0.00101172089216515	0.0295085260214835	0.0127796323220861	S100A7/CXCL8	2

BP	GO:0050921	positive regulation of chemotaxis	2/7	145/18870	0.00120072251041457	0.0303577985766361	0.0131474375790544	S100A7/CXCL8	2

BP	GO:0002687	positive regulation of leukocyte migration	2/7	149/18870	0.00126722121985456	0.0303577985766361	0.0131474375790544	S100A7/CXCL8	2

BP	GO:0001890	placenta development	2/7	151/18870	0.00130112433163713	0.0303577985766361	0.0131474375790544	CEBPB/SOCS3	2

BP	GO:0097530	granulocyte migration	2/7	156/18870	0.00138778507778908	0.0303577985766361	0.0131474375790544	S100A7/CXCL8	2

BP	GO:0030216	keratinocyte differentiation	2/7	177/18870	0.00178129500744839	0.0366737207415846	0.0158827542459795	S100A7/SPRR2G	2

BP	GO:0002685	regulation of leukocyte migration	2/7	230/18870	0.0029835419359833	0.0459355943589298	0.0198939115419124	S100A7/CXCL8	2

BP	GO:0050920	regulation of chemotaxis	2/7	232/18870	0.00303469267650523	0.0459355943589298	0.0198939115419124	S100A7/CXCL8	2

BP	GO:0030595	leukocyte chemotaxis	2/7	240/18870	0.00324345245395228	0.0459355943589298	0.0198939115419124	S100A7/CXCL8	2

BP	GO:0097529	myeloid leukocyte migration	2/7	242/18870	0.00329667928336776	0.0459355943589298	0.0198939115419124	S100A7/CXCL8	2

BP	GO:0009913	epidermal cell differentiation	2/7	245/18870	0.00337729530957135	0.0459355943589298	0.0198939115419124	S100A7/SPRR2G	2

BP	GO:0006959	humoral immune response	2/7	258/18870	0.00373735288238222	0.0459355943589298	0.0198939115419124	S100A7/CXCL8	2

BP	GO:0034976	response to endoplasmic reticulum stress	2/7	266/18870	0.00396754767036042	0.0459355943589298	0.0198939115419124	CEBPB/CXCL8	2

BP	GO:0071895	odontoblast differentiation	1/7	11/18870	0.004074068588382	0.0459355943589298	0.0198939115419124	CEBPB	1

BP	GO:1990440	positive regulation of transcription from RNA polymerase II promoter in response to endoplasmic reticulum stress	1/7	11/18870	0.004074068588382	0.0459355943589298	0.0198939115419124	CEBPB	1

BP	GO:0042532	negative regulation of tyrosine phosphorylation of STAT protein	1/7	12/18870	0.00444373195971048	0.0459355943589298	0.0198939115419124	SOCS3	1

BP	GO:0044341	sodium-dependent phosphate transport	1/7	12/18870	0.00444373195971048	0.0459355943589298	0.0198939115419124	CEBPB	1

BP	GO:0010992	ubiquitin recycling	1/7	13/18870	0.00481327771622098	0.0459355943589298	0.0198939115419124	UBE2C	1

BP	GO:0060670	branching involved in labyrinthine layer morphogenesis	1/7	13/18870	0.00481327771622098	0.0459355943589298	0.0198939115419124	SOCS3	1

BP	GO:2001198	regulation of dendritic cell differentiation	1/7	13/18870	0.00481327771622098	0.0459355943589298	0.0198939115419124	CEBPB	1

BP	GO:0045842	positive regulation of mitotic metaphase/anaphase transition	1/7	14/18870	0.00518270588910053	0.0459355943589298	0.0198939115419124	UBE2C	1

BP	GO:0050930	induction of positive chemotaxis	1/7	14/18870	0.00518270588910053	0.0459355943589298	0.0198939115419124	CXCL8	1

BP	GO:0060354	negative regulation of cell adhesion molecule production	1/7	14/18870	0.00518270588910053	0.0459355943589298	0.0198939115419124	CXCL8	1

BP	GO:0010820	positive regulation of T cell chemotaxis	1/7	15/18870	0.00555201650952775	0.0459355943589298	0.0198939115419124	S100A7	1

BP	GO:1902101	positive regulation of metaphase/anaphase transition of cell cycle	1/7	15/18870	0.00555201650952775	0.0459355943589298	0.0198939115419124	UBE2C	1

BP	GO:0043588	skin development	2/7	317/18870	0.00558737683541437	0.0459355943589298	0.0198939115419124	S100A7/SPRR2G	2

BP	GO:0060326	cell chemotaxis	2/7	325/18870	0.0058650637043314	0.0459355943589298	0.0198939115419124	S100A7/CXCL8	2

BP	GO:0010819	regulation of T cell chemotaxis	1/7	16/18870	0.00592120960867581	0.0459355943589298	0.0198939115419124	S100A7	1

BP	GO:0072540	T-helper 17 cell lineage commitment	1/7	16/18870	0.00592120960867581	0.0459355943589298	0.0198939115419124	SOCS3	1

BP	GO:0060644	mammary gland epithelial cell differentiation	1/7	17/18870	0.00629028521771036	0.0459355943589298	0.0198939115419124	CEBPB	1

BP	GO:0097396	response to interleukin-17	1/7	17/18870	0.00629028521771036	0.0459355943589298	0.0198939115419124	SOCS3	1

BP	GO:0097398	cellular response to interleukin-17	1/7	17/18870	0.00629028521771036	0.0459355943589298	0.0198939115419124	SOCS3	1

BP	GO:0007096	regulation of exit from mitosis	1/7	18/18870	0.00665924336779111	0.0459355943589298	0.0198939115419124	UBE2C	1

BP	GO:0036003	positive regulation of transcription from RNA polymerase II promoter in response to stress	1/7	18/18870	0.00665924336779111	0.0459355943589298	0.0198939115419124	CEBPB	1

BP	GO:1901970	positive regulation of mitotic sister chromatid separation	1/7	18/18870	0.00665924336779111	0.0459355943589298	0.0198939115419124	UBE2C	1

BP	GO:1904666	regulation of ubiquitin protein ligase activity	1/7	18/18870	0.00665924336779111	0.0459355943589298	0.0198939115419124	UBE2C	1

BP	GO:0045091	regulation of single stranded viral RNA replication via double stranded DNA intermediate	1/7	19/18870	0.0070280840900715	0.0459355943589298	0.0198939115419124	CXCL8	1

BP	GO:0002295	T-helper cell lineage commitment	1/7	20/18870	0.00739680741569693	0.0459355943589298	0.0198939115419124	SOCS3	1

BP	GO:0039692	single stranded viral RNA replication via double stranded DNA intermediate	1/7	20/18870	0.00739680741569693	0.0459355943589298	0.0198939115419124	CXCL8	1

BP	GO:0060353	regulation of cell adhesion molecule production	1/7	20/18870	0.00739680741569693	0.0459355943589298	0.0198939115419124	CXCL8	1

BP	GO:0140131	positive regulation of lymphocyte chemotaxis	1/7	20/18870	0.00739680741569693	0.0459355943589298	0.0198939115419124	S100A7	1

BP	GO:0090026	positive regulation of monocyte chemotaxis	1/7	21/18870	0.00776541337580794	0.0459355943589298	0.0198939115419124	S100A7	1

BP	GO:0051443	positive regulation of ubiquitin-protein transferase activity	1/7	22/18870	0.0081339020015373	0.0459355943589298	0.0198939115419124	UBE2C	1

BP	GO:0060713	labyrinthine layer morphogenesis	1/7	22/18870	0.0081339020015373	0.0459355943589298	0.0198939115419124	SOCS3	1

BP	GO:0001701	in utero embryonic development	2/7	392/18870	0.00843595742233237	0.0459355943589298	0.0198939115419124	CEBPB/SOCS3	2

BP	GO:0043373	CD4-positive, alpha-beta T cell lineage commitment	1/7	23/18870	0.00850227332401088	0.0459355943589298	0.0198939115419124	SOCS3	1

BP	GO:0060352	cell adhesion molecule production	1/7	23/18870	0.00850227332401088	0.0459355943589298	0.0198939115419124	CXCL8	1

BP	GO:0072574	hepatocyte proliferation	1/7	23/18870	0.00850227332401088	0.0459355943589298	0.0198939115419124	CEBPB	1

BP	GO:0072575	epithelial cell proliferation involved in liver morphogenesis	1/7	23/18870	0.00850227332401088	0.0459355943589298	0.0198939115419124	CEBPB	1

BP	GO:0050900	leukocyte migration	2/7	396/18870	0.00860309713456911	0.0459355943589298	0.0198939115419124	S100A7/CXCL8	2

BP	GO:0031145	anaphase-promoting complex-dependent catabolic process	1/7	24/18870	0.00887052737434957	0.0459355943589298	0.0198939115419124	UBE2C	1

BP	GO:0046426	negative regulation of receptor signaling pathway via JAK-STAT	1/7	24/18870	0.00887052737434957	0.0459355943589298	0.0198939115419124	SOCS3	1

BP	GO:0002363	alpha-beta T cell lineage commitment	1/7	25/18870	0.00923866418366626	0.0459355943589298	0.0198939115419124	SOCS3	1

BP	GO:0050927	positive regulation of positive chemotaxis	1/7	25/18870	0.00923866418366626	0.0459355943589298	0.0198939115419124	CXCL8	1

BP	GO:0072576	liver morphogenesis	1/7	25/18870	0.00923866418366626	0.0459355943589298	0.0198939115419124	CEBPB	1

BP	GO:0090023	positive regulation of neutrophil chemotaxis	1/7	25/18870	0.00923866418366626	0.0459355943589298	0.0198939115419124	CXCL8	1

BP	GO:0033598	mammary gland epithelial cell proliferation	1/7	26/18870	0.00960668378306728	0.0459355943589298	0.0198939115419124	CEBPB	1

BP	GO:0043369	CD4-positive or CD8-positive, alpha-beta T cell lineage commitment	1/7	26/18870	0.00960668378306728	0.0459355943589298	0.0198939115419124	SOCS3	1

BP	GO:0050926	regulation of positive chemotaxis	1/7	26/18870	0.00960668378306728	0.0459355943589298	0.0198939115419124	CXCL8	1

BP	GO:1901623	regulation of lymphocyte chemotaxis	1/7	26/18870	0.00960668378306728	0.0459355943589298	0.0198939115419124	S100A7	1

BP	GO:0050727	regulation of inflammatory response	2/7	425/18870	0.00985998801745271	0.0459355943589298	0.0198939115419124	CEBPB/SOCS3	2

BP	GO:0006817	phosphate ion transport	1/7	27/18870	0.00997458620365332	0.0459355943589298	0.0198939115419124	CEBPB	1

BP	GO:0010818	T cell chemotaxis	1/7	27/18870	0.00997458620365332	0.0459355943589298	0.0198939115419124	S100A7	1

BP	GO:0032753	positive regulation of interleukin-4 production	1/7	27/18870	0.00997458620365332	0.0459355943589298	0.0198939115419124	CEBPB	1

BP	GO:0060669	embryonic placenta morphogenesis	1/7	27/18870	0.00997458620365332	0.0459355943589298	0.0198939115419124	SOCS3	1

BP	GO:0090025	regulation of monocyte chemotaxis	1/7	29/18870	0.010710039632746	0.0470808000133933	0.0203898803065523	S100A7	1

BP	GO:0097421	liver regeneration	1/7	29/18870	0.010710039632746	0.0470808000133933	0.0203898803065523	CEBPB	1

BP	GO:1905820	positive regulation of chromosome separation	1/7	29/18870	0.010710039632746	0.0470808000133933	0.0203898803065523	UBE2C	1

BP	GO:0039694	viral RNA genome replication	1/7	30/18870	0.0110775907034202	0.0470808000133933	0.0203898803065523	CXCL8	1

BP	GO:0070979	protein K11-linked ubiquitination	1/7	30/18870	0.0110775907034202	0.0470808000133933	0.0203898803065523	UBE2C	1

BP	GO:0060674	placenta blood vessel development	1/7	31/18870	0.0114450247196128	0.0470808000133933	0.0203898803065523	SOCS3	1

BP	GO:0002360	T cell lineage commitment	1/7	32/18870	0.011812341712392	0.0470808000133933	0.0203898803065523	SOCS3	1

BP	GO:0010458	exit from mitosis	1/7	32/18870	0.011812341712392	0.0470808000133933	0.0203898803065523	UBE2C	1

BP	GO:0006935	chemotaxis	2/7	468/18870	0.0118675083293545	0.0470808000133933	0.0203898803065523	S100A7/CXCL8	2

BP	GO:0042330	taxis	2/7	470/18870	0.0119650030213653	0.0470808000133933	0.0203898803065523	S100A7/CXCL8	2

BP	GO:0043618	regulation of transcription from RNA polymerase II promoter in response to stress	1/7	33/18870	0.0121795417128173	0.0470808000133933	0.0203898803065523	CEBPB	1

BP	GO:0048566	embryonic digestive tract development	1/7	33/18870	0.0121795417128173	0.0470808000133933	0.0203898803065523	CXCL8	1

BP	GO:0072539	T-helper 17 cell differentiation	1/7	33/18870	0.0121795417128173	0.0470808000133933	0.0203898803065523	SOCS3	1

BP	GO:1904893	negative regulation of receptor signaling pathway via STAT	1/7	33/18870	0.0121795417128173	0.0470808000133933	0.0203898803065523	SOCS3	1

BP	GO:1903131	mononuclear cell differentiation	2/7	481/18870	0.0125077008498054	0.0470808000133933	0.0203898803065523	CEBPB/SOCS3	2

BP	GO:0090022	regulation of neutrophil chemotaxis	1/7	34/18870	0.0125466247519431	0.0470808000133933	0.0203898803065523	CXCL8	1

BP	GO:1902624	positive regulation of neutrophil migration	1/7	34/18870	0.0125466247519431	0.0470808000133933	0.0203898803065523	CXCL8	1

BP	GO:0032633	interleukin-4 production	1/7	35/18870	0.0129135908608164	0.0470808000133933	0.0203898803065523	CEBPB	1

BP	GO:0032673	regulation of interleukin-4 production	1/7	35/18870	0.0129135908608164	0.0470808000133933	0.0203898803065523	CEBPB	1

BP	GO:2000406	positive regulation of T cell migration	1/7	35/18870	0.0129135908608164	0.0470808000133933	0.0203898803065523	S100A7	1

BP	GO:0060563	neuroepithelial cell differentiation	1/7	38/18870	0.0140137879162955	0.0505652141309631	0.0218989198040863	CEBPB	1

BP	GO:0043620	regulation of DNA-templated transcription in response to stress	1/7	39/18870	0.0143802866144996	0.0508393971219684	0.0220176637159803	CEBPB	1

BP	GO:0051438	regulation of ubiquitin-protein transferase activity	1/7	39/18870	0.0143802866144996	0.0508393971219684	0.0220176637159803	UBE2C	1

BP	GO:0043368	positive T cell selection	1/7	40/18870	0.0147466685375891	0.0515969406081596	0.0223457426994736	SOCS3	1

BP	GO:0046627	negative regulation of insulin receptor signaling pathway	1/7	41/18870	0.0151129337165715	0.0515969406081596	0.0223457426994736	SOCS3	1

BP	GO:0042119	neutrophil activation	1/7	42/18870	0.0154790821824479	0.0515969406081596	0.0223457426994736	CXCL8	1

BP	GO:0140467	integrated stress response signaling	1/7	42/18870	0.0154790821824479	0.0515969406081596	0.0223457426994736	CEBPB	1

BP	GO:1900077	negative regulation of cellular response to insulin stimulus	1/7	42/18870	0.0154790821824479	0.0515969406081596	0.0223457426994736	SOCS3	1

BP	GO:2000403	positive regulation of lymphocyte migration	1/7	42/18870	0.0154790821824479	0.0515969406081596	0.0223457426994736	S100A7	1

BP	GO:0045840	positive regulation of mitotic nuclear division	1/7	44/18870	0.016211029098854	0.0535269828735744	0.023181610627954	UBE2C	1

BP	GO:0060711	labyrinthine layer development	1/7	46/18870	0.0169425095346847	0.0544025535517398	0.0235608051472196	SOCS3	1

BP	GO:0097028	dendritic cell differentiation	1/7	46/18870	0.0169425095346847	0.0544025535517398	0.0235608051472196	CEBPB	1

BP	GO:1902622	regulation of neutrophil migration	1/7	46/18870	0.0169425095346847	0.0544025535517398	0.0235608051472196	CXCL8	1

BP	GO:0072538	T-helper 17 type immune response	1/7	47/18870	0.0173080748998173	0.0550711474085097	0.0238503615844373	SOCS3	1

BP	GO:0006953	acute-phase response	1/7	48/18870	0.0176735237377124	0.0553824528751212	0.0239851825985487	CEBPB	1

BP	GO:0036230	granulocyte activation	1/7	49/18870	0.0180388560793252	0.0553824528751212	0.0239851825985487	CXCL8	1

BP	GO:0050732	negative regulation of peptidyl-tyrosine phosphorylation	1/7	49/18870	0.0180388560793252	0.0553824528751212	0.0239851825985487	SOCS3	1

BP	GO:2000404	regulation of T cell migration	1/7	49/18870	0.0180388560793252	0.0553824528751212	0.0239851825985487	S100A7	1

BP	GO:0045058	T cell selection	1/7	53/18870	0.0194990211018237	0.0589179660944236	0.0255163522333744	SOCS3	1

BP	GO:0045744	negative regulation of G protein-coupled receptor signaling pathway	1/7	54/18870	0.0198637714261199	0.0589179660944236	0.0255163522333744	CXCL8	1

BP	GO:0050873	brown fat cell differentiation	1/7	54/18870	0.0198637714261199	0.0589179660944236	0.0255163522333744	CEBPB	1

BP	GO:0070169	positive regulation of biomineral tissue development	1/7	54/18870	0.0198637714261199	0.0589179660944236	0.0255163522333744	CEBPB	1

BP	GO:0001541	ovarian follicle development	1/7	56/18870	0.0205929231735514	0.060062692589525	0.0260121134823808	CEBPB	1

BP	GO:0052372	modulation by symbiont of entry into host	1/7	56/18870	0.0205929231735514	0.060062692589525	0.0260121134823808	CXCL8	1

BP	GO:0010965	regulation of mitotic sister chromatid separation	1/7	59/18870	0.0216857790053325	0.06272745993278	0.0271661781363017	UBE2C	1

BP	GO:0051785	positive regulation of nuclear division	1/7	60/18870	0.022049831928992	0.0632577145503869	0.0273958222413706	UBE2C	1

BP	GO:0051306	mitotic sister chromatid separation	1/7	62/18870	0.0227775894311775	0.0648142788692042	0.0280699433298208	UBE2C	1

BP	GO:0031100	animal organ regeneration	1/7	64/18870	0.0235048826789493	0.0652392243976648	0.028253979889515	CEBPB	1

BP	GO:0048247	lymphocyte chemotaxis	1/7	64/18870	0.0235048826789493	0.0652392243976648	0.028253979889515	S100A7	1

BP	GO:0043903	regulation of biological process involved in symbiotic interaction	1/7	65/18870	0.0238683552845671	0.0652392243976648	0.028253979889515	CXCL8	1

BP	GO:0070059	intrinsic apoptotic signaling pathway in response to endoplasmic reticulum stress	1/7	65/18870	0.0238683552845671	0.0652392243976648	0.028253979889515	CEBPB	1

BP	GO:0070936	protein K48-linked ubiquitination	1/7	65/18870	0.0238683552845671	0.0652392243976648	0.028253979889515	UBE2C	1

BP	GO:0046854	phosphatidylinositol phosphate biosynthetic process	1/7	66/18870	0.0242317119191326	0.0652392243976648	0.028253979889515	SOCS3	1

BP	GO:0061180	mammary gland epithelium development	1/7	66/18870	0.0242317119191326	0.0652392243976648	0.028253979889515	CEBPB	1

BP	GO:0045670	regulation of osteoclast differentiation	1/7	69/18870	0.0253210863048527	0.0661371657216303	0.0286428627486158	CEBPB	1

BP	GO:0046626	regulation of insulin receptor signaling pathway	1/7	69/18870	0.0253210863048527	0.0661371657216303	0.0286428627486158	SOCS3	1

BP	GO:0050918	positive chemotaxis	1/7	69/18870	0.0253210863048527	0.0661371657216303	0.0286428627486158	CXCL8	1

BP	GO:2000401	regulation of lymphocyte migration	1/7	69/18870	0.0253210863048527	0.0661371657216303	0.0286428627486158	S100A7	1

BP	GO:0002548	monocyte chemotaxis	1/7	71/18870	0.0260467566052389	0.0664128518034152	0.0287622576231332	S100A7	1

BP	GO:0045600	positive regulation of fat cell differentiation	1/7	71/18870	0.0260467566052389	0.0664128518034152	0.0287622576231332	CEBPB	1

BP	GO:1900076	regulation of cellular response to insulin stimulus	1/7	71/18870	0.0260467566052389	0.0664128518034152	0.0287622576231332	SOCS3	1

BP	GO:0042130	negative regulation of T cell proliferation	1/7	72/18870	0.0264094180608357	0.0664128518034152	0.0287622576231332	CEBPB	1

BP	GO:0042093	T-helper cell differentiation	1/7	73/18870	0.0267719637611004	0.0664128518034152	0.0287622576231332	SOCS3	1

BP	GO:0071677	positive regulation of mononuclear cell migration	1/7	73/18870	0.0267719637611004	0.0664128518034152	0.0287622576231332	S100A7	1

BP	GO:0072678	T cell migration	1/7	73/18870	0.0267719637611004	0.0664128518034152	0.0287622576231332	S100A7	1

BP	GO:0045669	positive regulation of osteoblast differentiation	1/7	74/18870	0.0271343937368239	0.0664128518034152	0.0287622576231332	CEBPB	1

BP	GO:1905818	regulation of chromosome separation	1/7	74/18870	0.0271343937368239	0.0664128518034152	0.0287622576231332	UBE2C	1

BP	GO:0002294	CD4-positive, alpha-beta T cell differentiation involved in immune response	1/7	75/18870	0.0274967080187905	0.0667850501590561	0.0289234502944484	SOCS3	1

BP	GO:0002287	alpha-beta T cell activation involved in immune response	1/7	76/18870	0.0278589066377777	0.0667850501590561	0.0289234502944484	SOCS3	1

BP	GO:0002293	alpha-beta T cell differentiation involved in immune response	1/7	76/18870	0.0278589066377777	0.0667850501590561	0.0289234502944484	SOCS3	1

BP	GO:0042509	regulation of tyrosine phosphorylation of STAT protein	1/7	78/18870	0.0285829570098939	0.0680546595473664	0.0294732961648745	SOCS3	1

BP	GO:0051304	chromosome separation	1/7	81/18870	0.0296681658647933	0.0701612030586327	0.030385603730656	UBE2C	1

BP	GO:0007260	tyrosine phosphorylation of STAT protein	1/7	82/18870	0.0300296711518732	0.0705394959943329	0.0305494358591998	SOCS3	1

BP	GO:0031424	keratinization	1/7	83/18870	0.0303910609912356	0.0707124873856849	0.0306243554392139	SPRR2G	1

BP	GO:0002292	T cell differentiation involved in immune response	1/7	84/18870	0.0307523354136052	0.0707124873856849	0.0306243554392139	SOCS3	1

BP	GO:0045069	regulation of viral genome replication	1/7	85/18870	0.0311134944497014	0.0707124873856849	0.0306243554392139	CXCL8	1

BP	GO:0061515	myeloid cell development	1/7	85/18870	0.0311134944497014	0.0707124873856849	0.0306243554392139	CEBPB	1

BP	GO:0071230	cellular response to amino acid stimulus	1/7	85/18870	0.0311134944497014	0.0707124873856849	0.0306243554392139	CEBPB	1

BP	GO:0050672	negative regulation of lymphocyte proliferation	1/7	88/18870	0.0321962795474399	0.0727012763974449	0.0314856655676152	CEBPB	1

BP	GO:0032945	negative regulation of mononuclear cell proliferation	1/7	89/18870	0.0325569773455002	0.0730445004546479	0.0316343099713363	CEBPB	1

BP	GO:0030071	regulation of mitotic metaphase/anaphase transition	1/7	90/18870	0.0329175599107835	0.073383095342511	0.0317809495618694	UBE2C	1

BP	GO:0070098	chemokine-mediated signaling pathway	1/7	91/18870	0.0332780272739689	0.0737171490246146	0.0319256224347203	CXCL8	1

BP	GO:0043367	CD4-positive, alpha-beta T cell differentiation	1/7	93/18870	0.0339986165167334	0.0742318423467505	0.0321485272118258	SOCS3	1

BP	GO:1902099	regulation of metaphase/anaphase transition of cell cycle	1/7	93/18870	0.0339986165167334	0.0742318423467505	0.0321485272118258	UBE2C	1

BP	GO:0007091	metaphase/anaphase transition of mitotic cell cycle	1/7	94/18870	0.0343587384576388	0.0742318423467505	0.0321485272118258	UBE2C	1

BP	GO:0071229	cellular response to acid chemical	1/7	94/18870	0.0343587384576388	0.0742318423467505	0.0321485272118258	CEBPB	1

BP	GO:0070664	negative regulation of leukocyte proliferation	1/7	96/18870	0.0350786371317643	0.075322226970046	0.0326207539358996	CEBPB	1

BP	GO:0044784	metaphase/anaphase transition of cell cycle	1/7	97/18870	0.0354384139262712	0.0756307614280179	0.0327543748740889	UBE2C	1

BP	GO:1901992	positive regulation of mitotic cell cycle phase transition	1/7	98/18870	0.0357980757332558	0.0757794485279674	0.0328187686857964	UBE2C	1

BP	GO:1990868	response to chemokine	1/7	99/18870	0.0361576225833444	0.0757794485279674	0.0328187686857964	CXCL8	1

BP	GO:1990869	cellular response to chemokine	1/7	99/18870	0.0361576225833444	0.0757794485279674	0.0328187686857964	CXCL8	1

BP	GO:0008585	female gonad development	1/7	101/18870	0.0368763715353125	0.0763711836530141	0.0330750389354407	CEBPB	1

BP	GO:0120162	positive regulation of cold-induced thermogenesis	1/7	101/18870	0.0368763715353125	0.0763711836530141	0.0330750389354407	CEBPB	1

BP	GO:0070167	regulation of biomineral tissue development	1/7	102/18870	0.0372355736984139	0.0766614752614404	0.0332007592109697	CEBPB	1

BP	GO:0033045	regulation of sister chromatid segregation	1/7	104/18870	0.0379536335518585	0.0767441118715366	0.0332365476977482	UBE2C	1

BP	GO:1990830	cellular response to leukemia inhibitory factor	1/7	104/18870	0.0379536335518585	0.0767441118715366	0.0332365476977482	SOCS3	1

BP	GO:0046545	development of primary female sexual characteristics	1/7	105/18870	0.0383124913033845	0.0767441118715366	0.0332365476977482	CEBPB	1

BP	GO:0030316	osteoclast differentiation	1/7	106/18870	0.0386712343122242	0.0767441118715366	0.0332365476977482	CEBPB	1

BP	GO:0046425	regulation of receptor signaling pathway via JAK-STAT	1/7	106/18870	0.0386712343122242	0.0767441118715366	0.0332365476977482	SOCS3	1

BP	GO:1990823	response to leukemia inhibitory factor	1/7	106/18870	0.0386712343122242	0.0767441118715366	0.0332365476977482	SOCS3	1

BP	GO:0002526	acute inflammatory response	1/7	107/18870	0.0390298626089529	0.0767441118715366	0.0332365476977482	CEBPB	1

BP	GO:0030593	neutrophil chemotaxis	1/7	107/18870	0.0390298626089529	0.0767441118715366	0.0332365476977482	CXCL8	1

BP	GO:0031398	positive regulation of protein ubiquitination	1/7	110/18870	0.040105059532126	0.0784177141689614	0.0339613559107682	UBE2C	1

BP	GO:0071347	cellular response to interleukin-1	1/7	111/18870	0.0404632292860321	0.0786785013895068	0.0340742983461323	CXCL8	1

BP	GO:0044344	cellular response to fibroblast growth factor stimulus	1/7	115/18870	0.0418947630136569	0.0810119726783421	0.0350848844080639	CXCL8	1

BP	GO:0007088	regulation of mitotic nuclear division	1/7	118/18870	0.0429672116116235	0.0816049069155854	0.035341673972464	UBE2C	1

BP	GO:0035710	CD4-positive, alpha-beta T cell activation	1/7	119/18870	0.0433244657455139	0.0816049069155854	0.035341673972464	SOCS3	1

BP	GO:1901989	positive regulation of cell cycle phase transition	1/7	119/18870	0.0433244657455139	0.0816049069155854	0.035341673972464	UBE2C	1

BP	GO:1904892	regulation of receptor signaling pathway via STAT	1/7	119/18870	0.0433244657455139	0.0816049069155854	0.035341673972464	SOCS3	1

BP	GO:0046660	female sex differentiation	1/7	120/18870	0.0436816055641787	0.0816049069155854	0.035341673972464	CEBPB	1

BP	GO:0022612	gland morphogenesis	1/7	122/18870	0.0443955423777597	0.0816049069155854	0.035341673972464	CEBPB	1

BP	GO:0002065	columnar/cuboidal epithelial cell differentiation	1/7	123/18870	0.0447523394336244	0.0816049069155854	0.035341673972464	CEBPB	1

BP	GO:0002286	T cell activation involved in immune response	1/7	123/18870	0.0447523394336244	0.0816049069155854	0.035341673972464	SOCS3	1

BP	GO:0071774	response to fibroblast growth factor	1/7	123/18870	0.0447523394336244	0.0816049069155854	0.035341673972464	CXCL8	1

BP	GO:0002761	regulation of myeloid leukocyte differentiation	1/7	124/18870	0.0451090222961607	0.0816049069155854	0.035341673972464	CEBPB	1

BP	GO:0046632	alpha-beta T cell differentiation	1/7	124/18870	0.0451090222961607	0.0816049069155854	0.035341673972464	SOCS3	1

BP	GO:0072676	lymphocyte migration	1/7	124/18870	0.0451090222961607	0.0816049069155854	0.035341673972464	S100A7	1

BP	GO:0008286	insulin receptor signaling pathway	1/7	125/18870	0.0454655909958261	0.0816049069155854	0.035341673972464	SOCS3	1

BP	GO:0071675	regulation of mononuclear cell migration	1/7	125/18870	0.0454655909958261	0.0816049069155854	0.035341673972464	S100A7	1

BP	GO:0043200	response to amino acid	1/7	126/18870	0.0458220455630721	0.0818250813626288	0.0354370277179505	CEBPB	1

BP	GO:0031623	receptor internalization	1/7	128/18870	0.0465346124220807	0.0822581532713547	0.0356245836724063	CXCL8	1

BP	GO:0045931	positive regulation of mitotic cell cycle	1/7	128/18870	0.0465346124220807	0.0822581532713547	0.0356245836724063	UBE2C	1

BP	GO:1990266	neutrophil migration	1/7	129/18870	0.0468907247747137	0.0824711239756271	0.0357168176014746	CXCL8	1

BP	GO:0006661	phosphatidylinositol biosynthetic process	1/7	131/18870	0.0476026074783644	0.0824797654328096	0.0357205600671416	SOCS3	1

BP	GO:0019079	viral genome replication	1/7	131/18870	0.0476026074783644	0.0824797654328096	0.0357205600671416	CXCL8	1

BP	GO:0051983	regulation of chromosome segregation	1/7	131/18870	0.0476026074783644	0.0824797654328096	0.0357205600671416	UBE2C	1

BP	GO:1903322	positive regulation of protein modification by small protein conjugation or removal	1/7	132/18870	0.0479583778902142	0.0824873757752112	0.0357238559748283	UBE2C	1

BP	GO:0030879	mammary gland development	1/7	133/18870	0.0483140343826237	0.0824873757752112	0.0357238559748283	CEBPB	1

BP	GO:0050868	negative regulation of T cell activation	1/7	133/18870	0.0483140343826237	0.0824873757752112	0.0357238559748283	CEBPB	1

BP	GO:0048565	digestive tract development	1/7	134/18870	0.0486695769859925	0.082691028859696	0.0358120546038984	CXCL8	1

MF	GO:0050786	RAGE receptor binding	1/6	10/18496	0.00324000050750728	0.0485924456108936	0.0230174742367391	S100A7	1

MF	GO:0044389	ubiquitin-like protein ligase binding	2/6	327/18496	0.00445961101004077	0.0485924456108936	0.0230174742367391	CEBPB/UBE2C	2

MF	GO:0046935	1-phosphatidylinositol-3-kinase regulator activity	1/6	16/18496	0.00517979828930559	0.0485924456108936	0.0230174742367391	SOCS3	1

MF	GO:0045236	CXCR chemokine receptor binding	1/6	18/18496	0.00582569803850885	0.0485924456108936	0.0230174742367391	CXCL8	1

MF	GO:0000979	RNA polymerase II core promoter sequence-specific DNA binding	1/6	19/18496	0.00614851683988149	0.0485924456108936	0.0230174742367391	CEBPB	1

MF	GO:0035035	histone acetyltransferase binding	1/6	24/18496	0.00776130087099158	0.0485924456108936	0.0230174742367391	CEBPB	1

MF	GO:0061631	ubiquitin conjugating enzyme activity	1/6	34/18496	0.010980326614226	0.0485924456108936	0.0230174742367391	UBE2C	1

MF	GO:0061650	ubiquitin-like protein conjugating enzyme activity	1/6	40/18496	0.0129075613205676	0.0485924456108936	0.0230174742367391	UBE2C	1

MF	GO:0140313	molecular sequestering activity	1/6	40/18496	0.0129075613205676	0.0485924456108936	0.0230174742367391	S100A7	1

MF	GO:0001046	core promoter sequence-specific DNA binding	1/6	41/18496	0.0132284626335323	0.0485924456108936	0.0230174742367391	CEBPB	1

MF	GO:0035198	miRNA binding	1/6	42/18496	0.013549277004933	0.0485924456108936	0.0230174742367391	SOCS3	1

MF	GO:0001784	phosphotyrosine residue binding	1/6	47/18496	0.0151520453979442	0.0485924456108936	0.0230174742367391	SOCS3	1

MF	GO:0008009	chemokine activity	1/6	49/18496	0.0157925448235404	0.0485924456108936	0.0230174742367391	CXCL8	1

MF	GO:0061980	regulatory RNA binding	1/6	56/18496	0.0180315598879595	0.0506395666890895	0.0239871631685161	SOCS3	1

MF	GO:0045309	protein phosphorylated amino acid binding	1/6	59/18496	0.0189898375084085	0.0506395666890895	0.0239871631685161	SOCS3	1

MF	GO:0004860	protein kinase inhibitor activity	1/6	72/18496	0.0231333736809248	0.0533862332635361	0.0252882157564119	SOCS3	1

MF	GO:0042379	chemokine receptor binding	1/6	74/18496	0.0237695443706185	0.0533862332635361	0.0252882157564119	CXCL8	1

MF	GO:0019210	kinase inhibitor activity	1/6	76/18496	0.0244053697751341	0.0533862332635361	0.0252882157564119	SOCS3	1

MF	GO:0048306	calcium-dependent protein binding	1/6	79/18496	0.0253584608001797	0.0533862332635361	0.0252882157564119	S100A7	1

MF	GO:0051219	phosphoprotein binding	1/6	93/18496	0.0297959659999945	0.0595919319999889	0.0282277572631526	SOCS3	1

MF	GO:0031490	chromatin DNA binding	1/6	120/18496	0.0383064744857217	0.0729647133061365	0.0345622326186962	CEBPB	1

MF	GO:0042826	histone deacetylase binding	1/6	127/18496	0.0405027178294098	0.0736413051443815	0.0348827234894439	CEBPB	1
